# Supplementary material for: Genetic diversity patterns and domestication origin of soybean
Source: Theor Appl Genet. 2018 Dec 26;132(4):1179–93. doi: 10.1007/s00122-018-3271-7 (PMC6449312; doi:10.1007/s00122-018-3271-7)
Supplement: Supplementary file 1 — Supplementary material 1 (PDF 1948 kb) [file 122_2018_3271_MOESM1_ESM.pdf]

# Supplementary Figs S1 to S12 for
# Genetic diversity patterns and domestication origin of soybean

**Jeong et al., 2018**

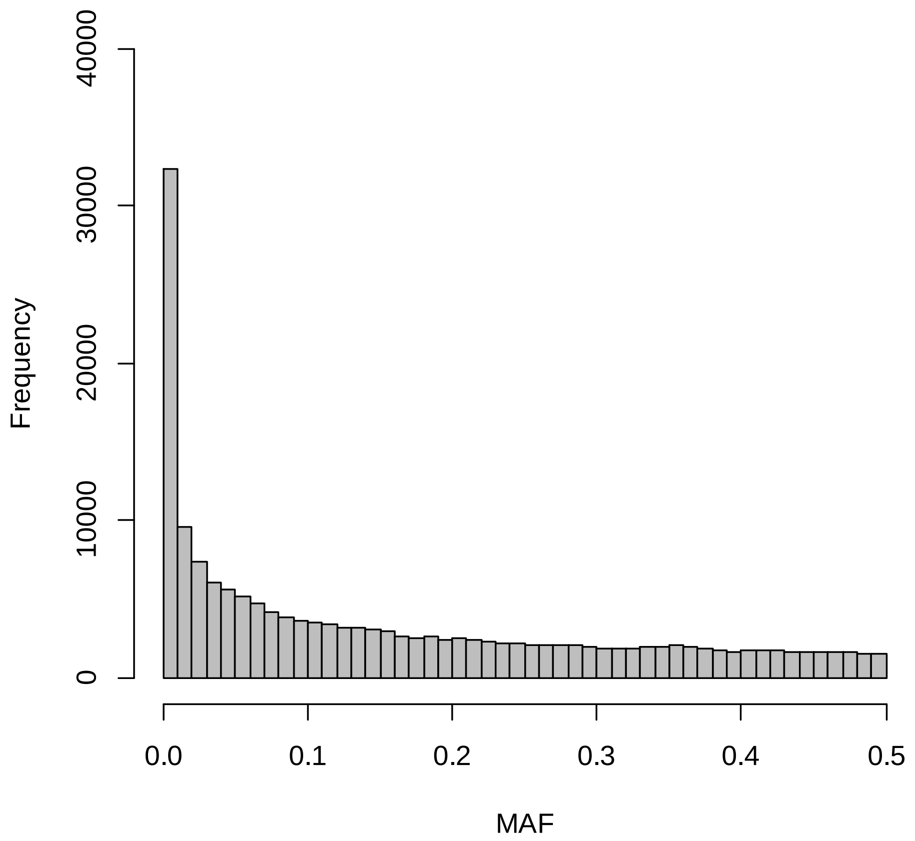


**Fig. S1.** Histogram of minor allele frequencies (MAF) of single-nucleotide polymorphisms in the 3,036 non-redundant soybean set.

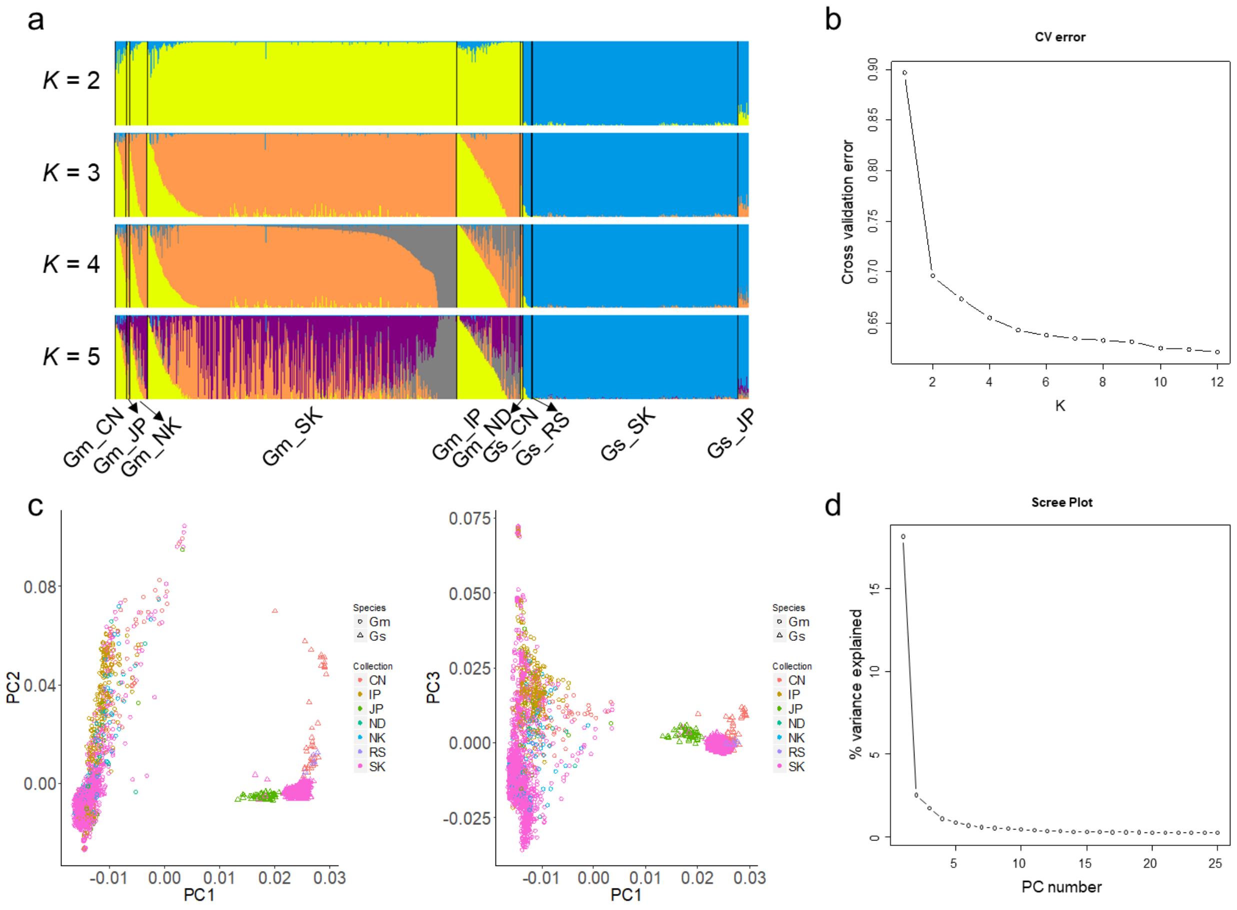


**Fig. S2.** Population structure of the 3,036 non-redundant soybean set. Before showing ancestral genomic fractions of the domesticated (*Glycine max*, Gm) and wild (*G. soja*, Gs) soybean accessions, they were grouped on the basis of countries of origin and improvement status represented by two-letter codes —CN, China; JP, Japan; NK, North Korea; RS, Russia; SK, South Korea; IP, improved breeding line; and ND, not determined. **a** ADMIXTURE plots. **b** Estimated cross-validation (CV) error plot from ADMIXTURE. **c** Principal components (PC) of SNP variation in the soybean accession set. The plots show the first three principal components. **d** Scree plot of the PC number and their contribution to variance from principal component analysis.

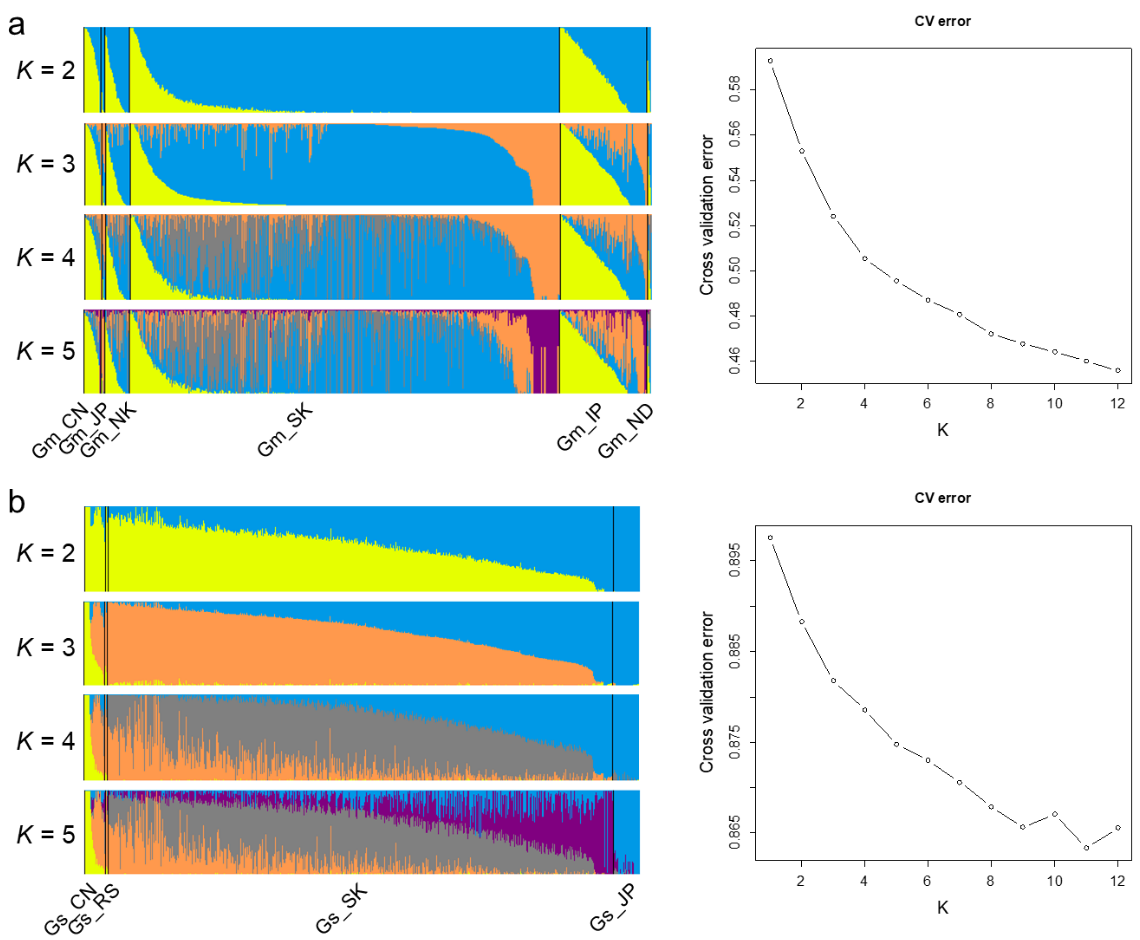


**Fig. S3.** ADMIXTURE plots and estimated cross-validation (CV) error plots of 1,957 domesticated (**a**) and 1,079 wild (**b**) soybean accessions in the 3,036 non-redundant soybean accession set. Each color represents one population. Before showing ancestral genomic fractions of the domesticated (*Glycine max*, Gm) and wild (*G. soja*, Gs) soybean accessions, they were grouped on the basis of countries of origin and improvement status represented by two-letter codes —CN, China; JP, Japan; NK, North Korea; RS, Russia; SK, South Korea; IP, improved breeding line; and ND, not determined.

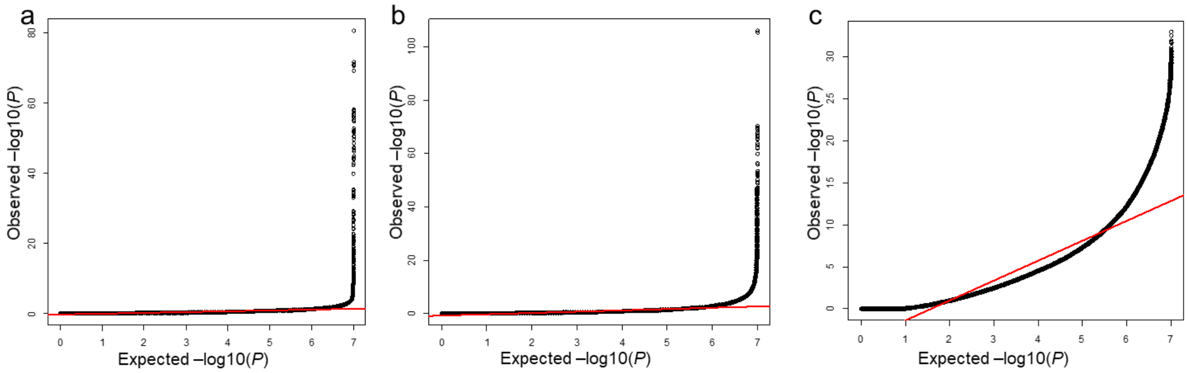


**Fig. S4.** Quantile-quantile plots for flower color (**a**), seed-coat color (**b**), and domestication (**c**) in genome-wide association analyses of soybean traits.

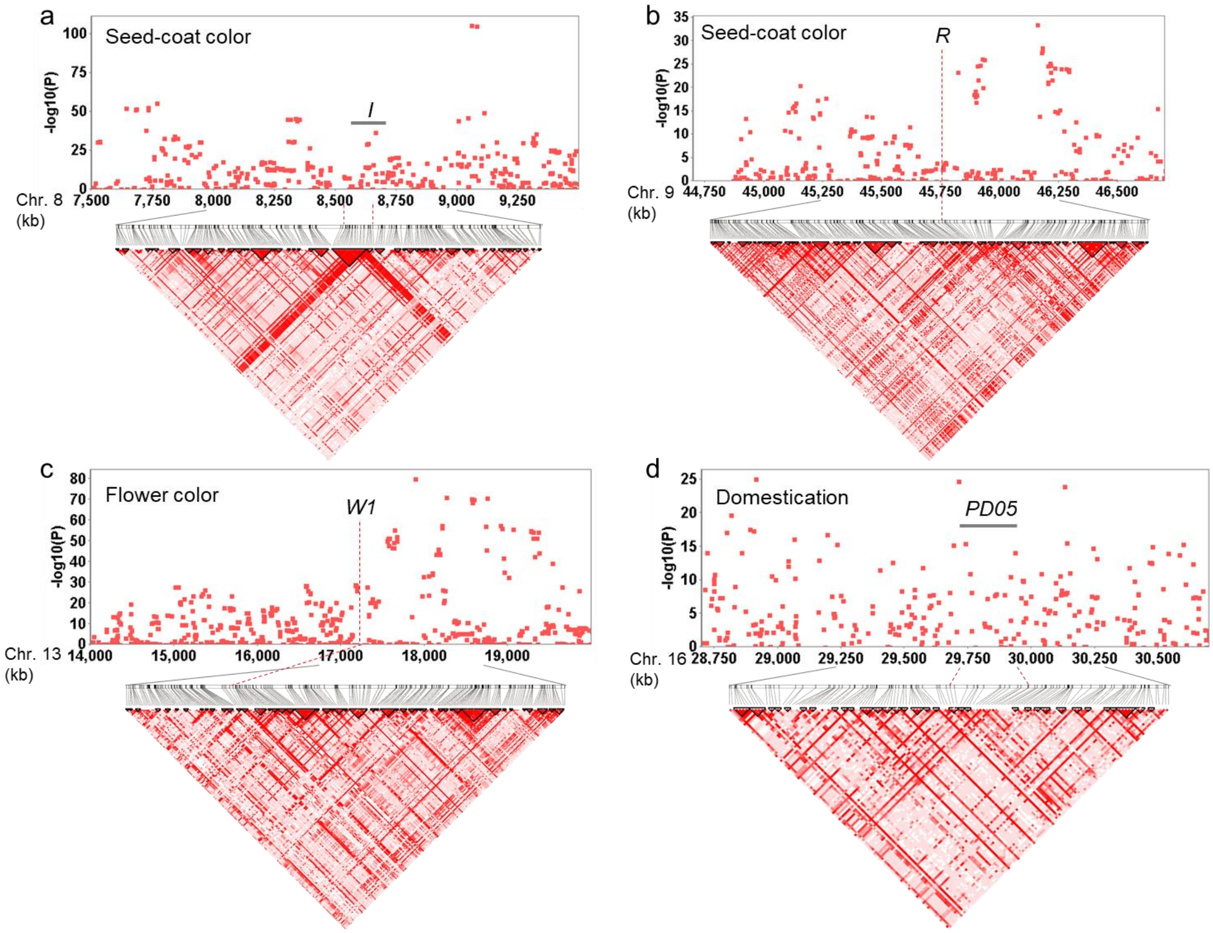


**Fig. S5.** Genome-wide association scans for 3,036 soybean accessions for flower color, seed-coat color, and domestication. **a** Local Manhattan plot (top) and LD heatmap (bottom) surrounding the *I* locus on chromosome 8. Bar and dashed lines indicate the region of the *I* locus. **b** Local Manhattan plot (top) and LD heatmap (bottom) surrounding the *R* locus on chromosome 9. A dashed line indicate the region of the *R* locus. **c** Local Manhattan plot (top) and LD heatmap (bottom) surrounding the *W1* locus on chromosome 13. Dashed lines indicate the region of the *W1* locus. **d** Local Manhattan plot (top) and LD heatmap (bottom) surrounding the *PD05* locus on chromosome 16. Bar and dashed lines indicate the region of the *R* locus.

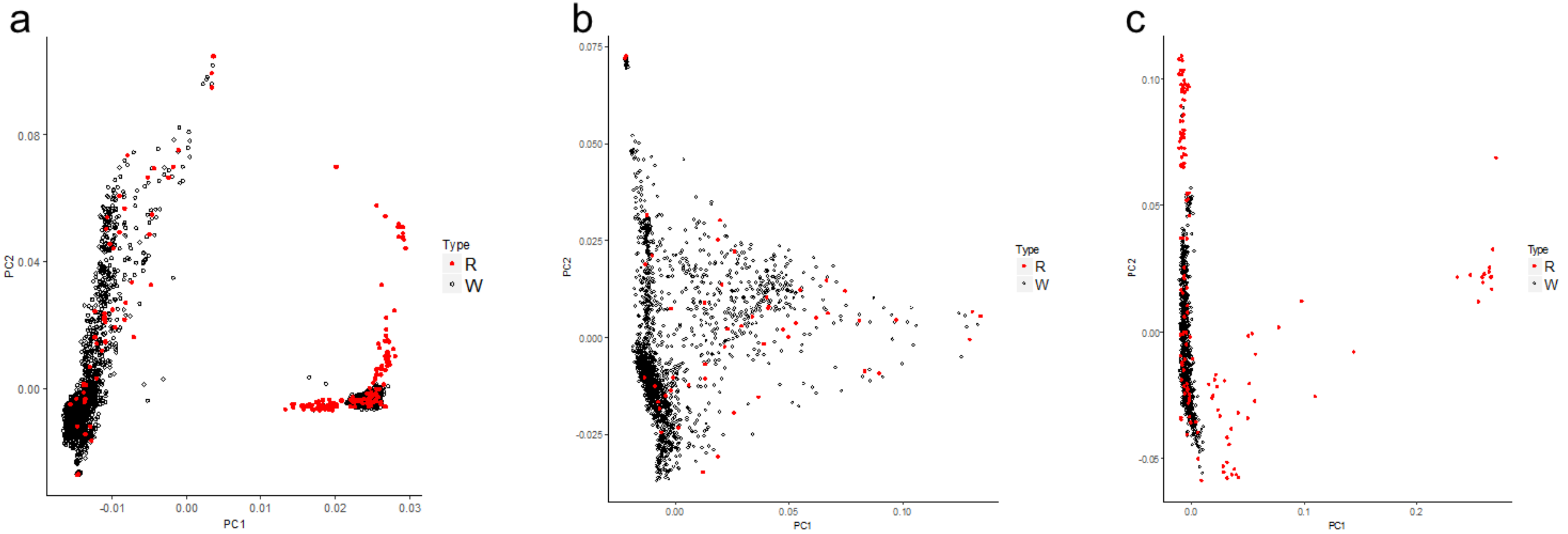


**Fig. S6.** Principal component analysis (PCA) plots of 1,957 domesticated and 1,079 wild soybean accessions in the 3,036 non-redundant soybean accession set that show distribution of the 194 representative set. Selected representative accessions were indicated by filled red circles (R) and unselected accessions by black circles (W). (A) PCA plot of the 3,036 non-redundant soybean accession set. (B) PCA plot of the 1,957 domesticated soybean accession set. (C) PCA plot of the 1,079 wild soybean accession set.

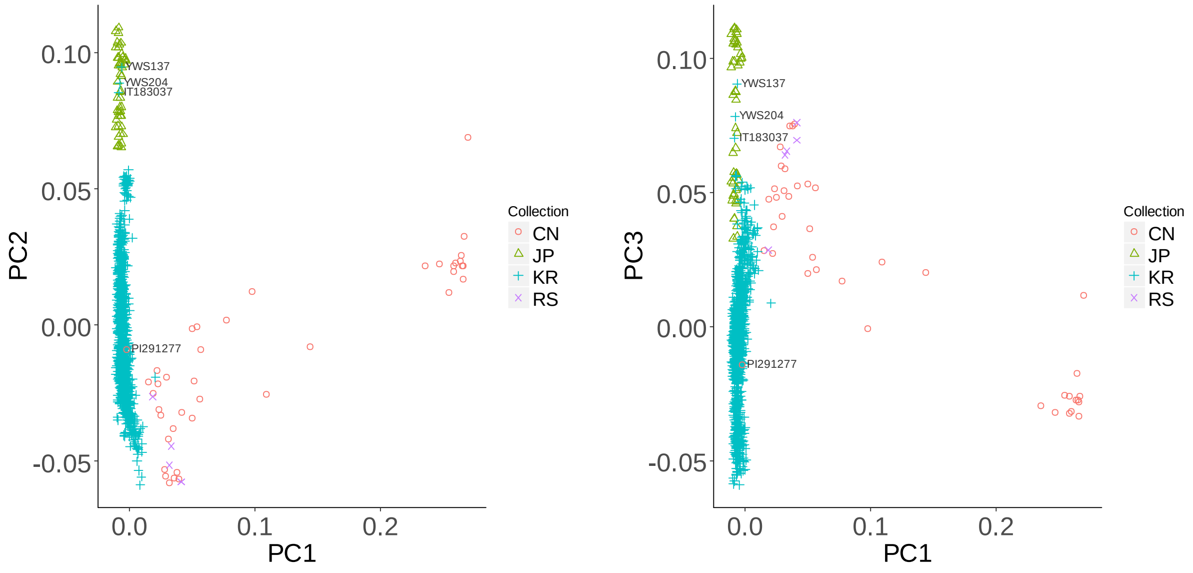


**Fig. S7.** Locations of four anomalies in the principal components analysis plots of the wild soybean accessions. Three Korean accessions YWS137, YWS204, and IT183037 were grouped with Japanese accessions. One Chinese accession PI 291277 was grouped with Korean accessions.

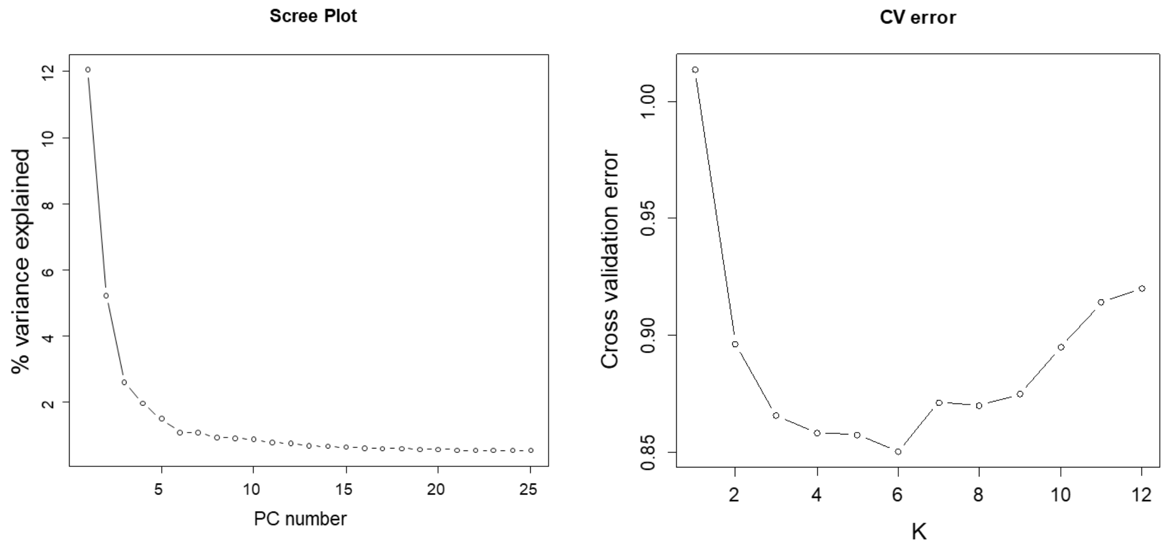


**Fig. S8.** Scree plot of the principal component (PC) number and their contribution to variance from principal component analysis and estimated cross-validation (CV) error plot from ADMIXTURE analysis for the representative 194 soybean accessions comprising 50 *G. max* and 144 *G. soja* accessions.

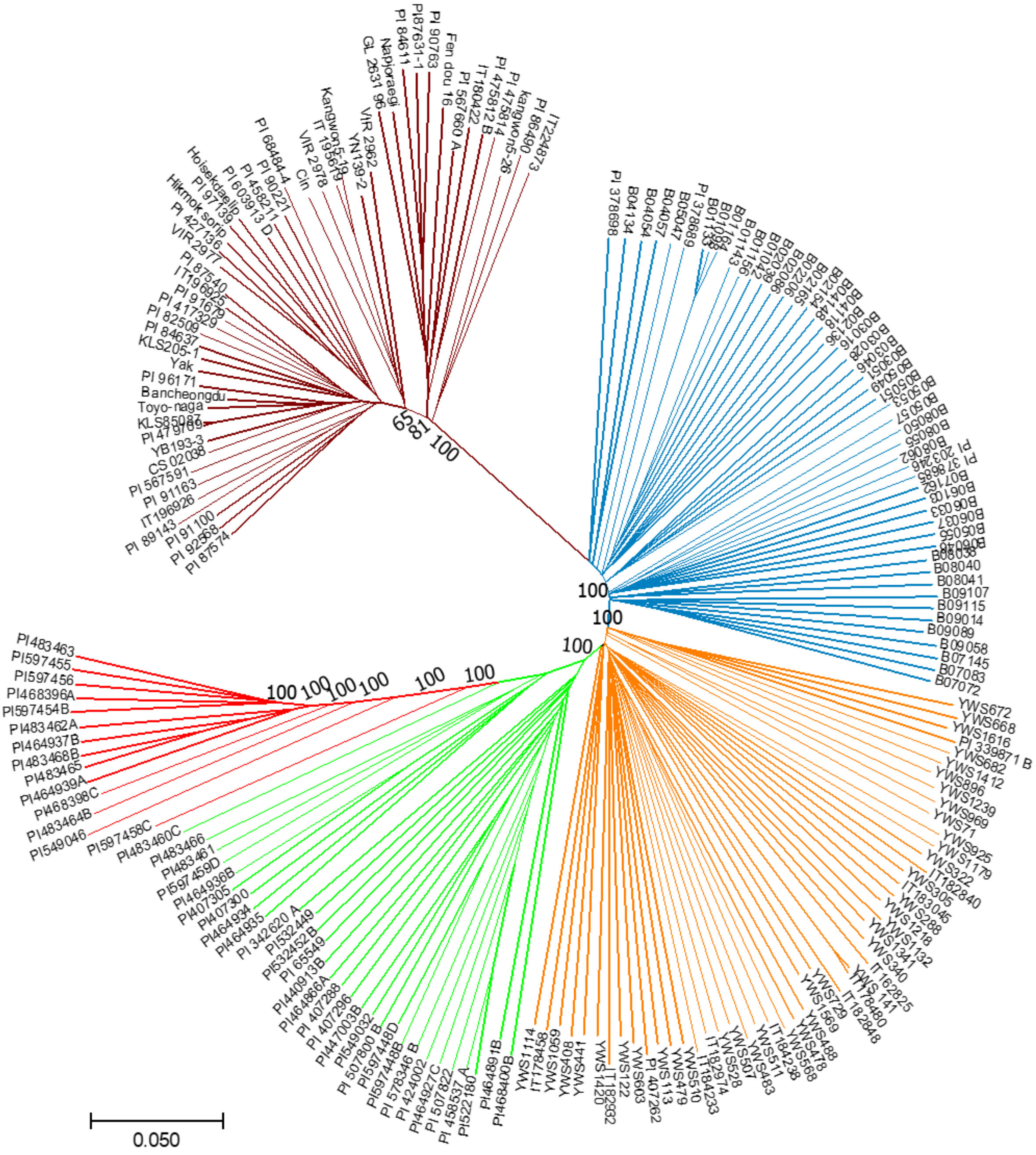


**Fig. S9.** Neighbor-joining phylogenetic tree of 194 soybean accessions based on the SNPs genotyped by the 180K AXIOM SoyaSNP array, with the evolutionary distances measured by the *p*-distance. Taxa in the neighbor-joining tree and >65 bootstrap values from 1,000 bootstrap replications at basal branches are denoted. Branches in the neighbor-joining tree are represented by different colors that correspond with soybean subgroups identified: brown (*Glycine max*, Gm), red (*G. soja*, Gs-I), green (*G. soja*, Gs-II), orange (*G. soja*, Gs-III) and blue (*G. soja*, Gs-IV).

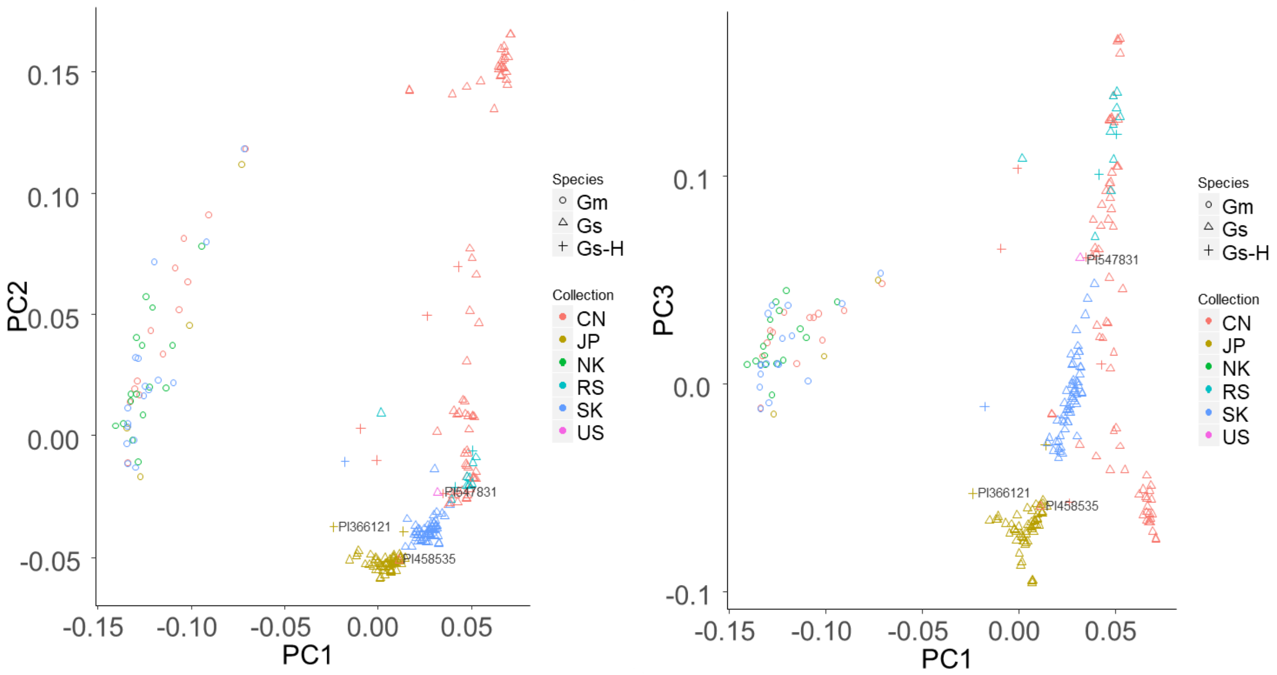


**Fig. S10.** Principal components of SNP variation in the expanded set that contained the representative set selected in this study and 62 wild soybean accessions reported by Zhou et al. (2015). Each of PC1, PC2, and PC3 explained 11.5%, 6.2%, and 3.2% of variance in the data. Countries of collection of the soybean accessions and species names are represented by two-letter codes —CN, China; JP, Japan; NK, North Korea; RS, Russia; SK, South Korea; US, United States of America; Gm, *G. max*; Gs, *G. soja*; and Gs-H, *G. soja* with heterozygous SNP calls > 20%. One Chinese accession PI 458535, which was considered an anomaly, was grouped with Japanese accessions. PI 457831 from US is a hybrid. PI 366121 had 20.7% heterozygous SNPs and was detected to be a hybrid in our SNP arrary data analysis. In PCA plots, accessions with the high level of heterozygous SNPs tended to be positioned between *G. max* and *G. soja* groups.

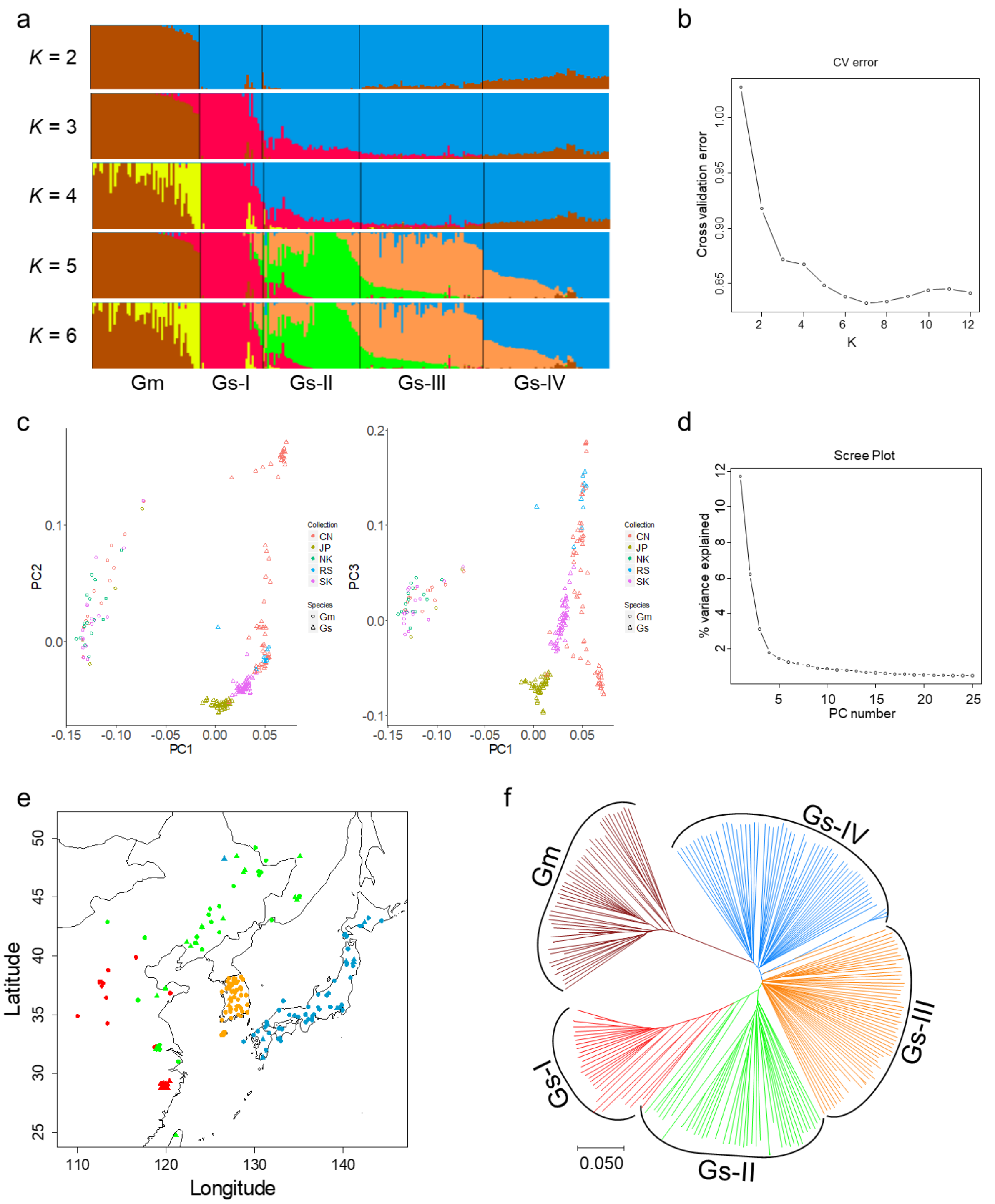


**Fig. S11.** Population structure of the expanded set that contained the representative set selected in this study and 45 wild soybean accessions reported by Zhou et al. (2015). **a** ADMIXTURE plots. Each color represents one population. **b** Estimated cross-validation (CV) error plot from ADMIXTURE. **c** Principal components (PC) of SNP variation in the soybean accession set. Each of PC1, PC2, and PC3 explained 11.7%, 6.2%, and 3.1% of variance in the data. Countries of collection of the soybean accessions and species names are represented by two-letter codes —CN, China; JP, Japan; NK, North

Korea; RS, Russia; SK, South Korea; Gm, *G. max*; and Gs, *G. soja*. **d** Scree plot of the PC number and their contribution to variance from principal component analysis. **e** Geographic distribution of the four *G. soja* subgroups. Gs-I is red, Gs-II green, Gs-III orange, and Gs-IV blue. **f** Neighbor-joining phylogenetic tree of 239 soybean accessions, with evolutionary distances measured by the *p*-distance. The taxa used in the neighbor-joining tree and bootstrap values from 1000 bootstrap replications at branches are described in Fig. S11.

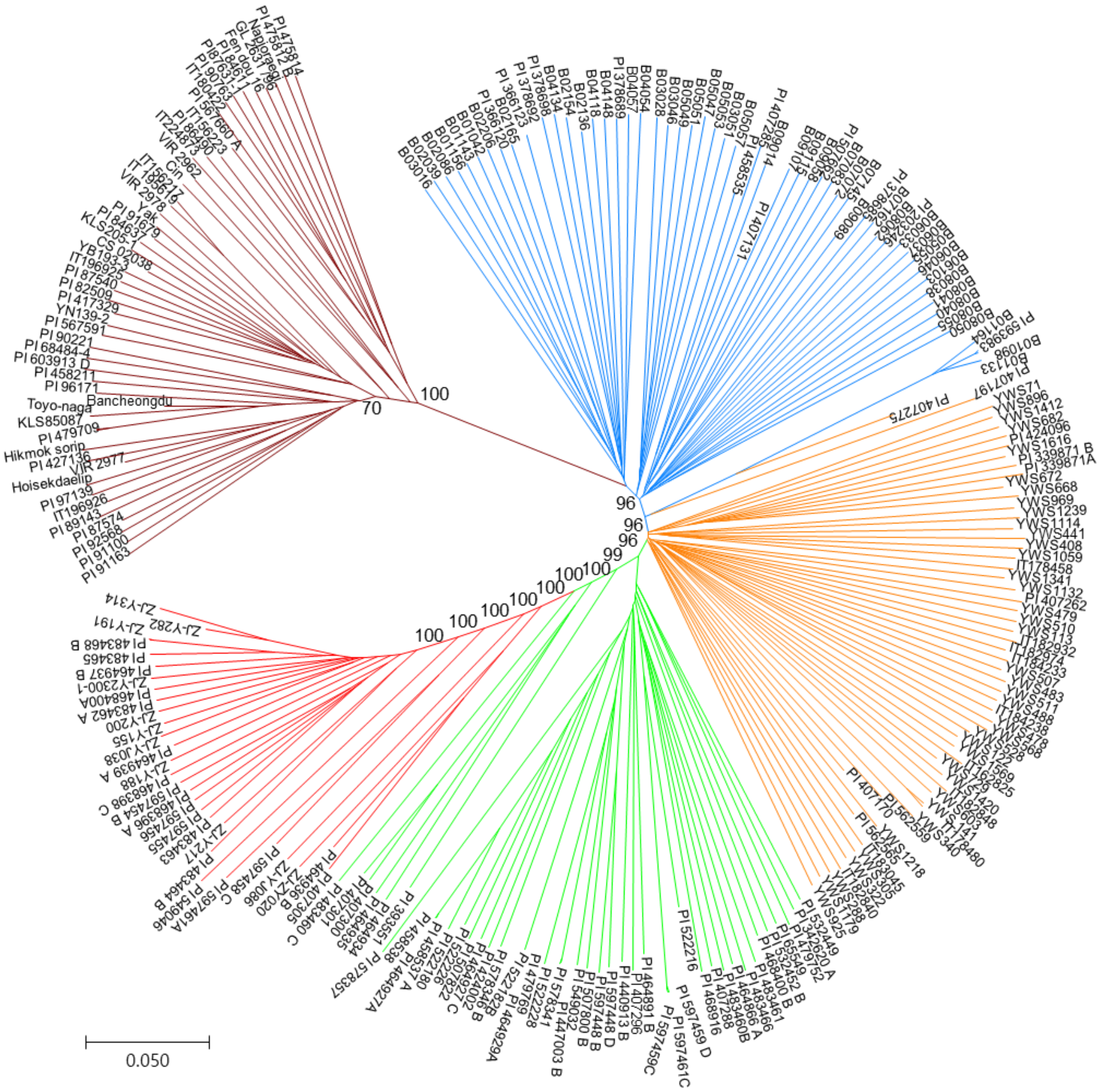


**Fig. S12.** Neighbor-joining phylogenetic tree of 239 soybean accessions of the expanded set that contained the representative set selected in this study and 45 wild soybean accessions reported by Zhou et al. (2015), with the evolutionary distances measured by the *p*-distance. Taxa in the neighbor-joining tree and >65 bootstrap values from 1,000 bootstrap replications at basal branches are denoted. Branches in the neighbor-joining tree are represented by different colors that correspond with soybean subgroups identified: brown (*Glycine max*, Gm), red (*G. soja*, Gs-I), green (*G. soja*, Gs-II), orange (*G. soja*, Gs-III) and blue (*G. soja*, Gs-IV).
