# Supplementary material for: Genetic diversity patterns and domestication origin of soybean
Source: Theor Appl Genet. 2018 Dec 26;132(4):1179–93. doi: 10.1007/s00122-018-3271-7 (PMC6449312; doi:10.1007/s00122-018-3271-7)
Supplement: Supplementary file 2 — Supplementary material 2 (PDF 2792 kb) [file 122_2018_3271_MOESM2_ESM.pdf]

Table S1. List of 4,234 soybean accessions

Nonredundant sample group index	Species	Name or accession code (IT number for Korea, PI number for US, and B number for Japan) used in this study	Other name or accession code	DNA ID for SNParray	Collected country (*G. max* and *G. soja*) and province (*G. soja*)	Improvement status of *G. max* or collection province of G. soja	Flower color	Seed-coat color	Remarks
1	G. max	PI 96983		96983	North Korea	Landrace	White	Yellow	
	G. max	PI 96983		PI96983	North Korea	Landrace			
2	G. max	SS0404-T5-76		1000Al	South Korea	Improved line	White	Yellow	
3	G. max	Cheonsang		CD-1166	South Korea	Improved line	White	Yellow	
4	G. max	Seonyu		CD-1168	South Korea	Improved line	Purple	Yellow	
5	G. max	Nampung		CD-1171	South Korea	Improved line	White	Yellow	
6	G. max	Jinpum2		CD-1173	South Korea	Improved line	Purple	Yellow	
7	G. max	Singi		CD-1174	South Korea	Improved line	Purple	Yellow	
8	G. max	Daeyang		CD-1175	South Korea	Improved line	Purple	Yellow	
9	G. max	Daeha		CD-1176	South Korea	Improved line	White	Yellow	
10	G. max	Jangyeop		CD-1177	South Korea	Improved line	Purple	Yellow	
11	G. max	Saeal		CD-1179	South Korea	Improved line	Purple	Yellow	
12	G. max	Bogwang		CD-1180	South Korea	Improved line	Purple	Yellow	
13	G. max	Muhan		CD-1182	South Korea	Improved line	Purple	Yellow	
14	G. max	Jangsu		CD-1183	South Korea	Improved line	Purple	Yellow	
15	G. max	Danwon		CD-1184	South Korea	Improved line	White	Yellow	
16	G. max	Samnam		CD-1186	South Korea	Improved line	White	Yellow	
17	G. max	Danbaek		CD-1188	South Korea	Improved line	Purple	Yellow	
	G. max	Danbaek		Danbaek	South Korea	Improved line			
18	G. max	Geumgang		CD-1191	South Korea	Improved line	White	Yellow	
	G. max	Dangyung		CD-1181	South Korea	Improved line			
	G. max	Duyu		CD-1189	South Korea	Improved line			
19	G. max	Alchan		CD-1192	South Korea	Improved line	Purple	Yellow	
20	G. max	Dajang		CD-1193	South Korea	Improved line	White	Yellow	
21	G. max	Jangmi		CD-1194	South Korea	Improved line	Purple	Yellow	
22	G. max	Sodam		CD-1195	South Korea	Improved line	Purple	Yellow	
23	G. max	Songhak		CD-1196	South Korea	Improved line	White	Yellow	
24	G. max	Ilmi		CD-1197	South Korea	Improved line	Purple	Yellow	
25	G. max	Jangwon		CD-1199	South Korea	Improved line	Purple	Yellow	
26	G. max	Jinmi		CD-1200	South Korea	Improved line	Purple	Yellow	
	G. max	Mansu		CD-1204	South Korea	Improved line			
27	G. max	Hojang		CD-1201	South Korea	Improved line	White	Yellow	
28	G. max	Daemang2		CD-1203	South Korea	Improved line	White	Green yellow	
29	G. max	Jungmo3003		CD-1205	South Korea	Improved line	Purple	Yellow	
30	G. max	Jungmo3004		CD-1206	South Korea	Improved line	White	Yellow	
31	G. max	Jungmo3005		CD-1209	South Korea	Improved line	White	Green	
32	G. max	Jangyeon		CD-1212	South Korea	Improved line	White	Yellow	
33	G. max	Manpung		CD-1213	South Korea	Improved line	White	Yellow	
34	G. max	Soyang		CD-1215	South Korea	Improved line	White	Yellow	
35	G. max	Cheonga		CD-1216	South Korea	Improved line	White	Yellow	
36	G. max	Hoban		CD-1217	South Korea	Improved line	Purple	Yellow	
37	G. max	Daewang		CD-1218	South Korea	Improved line	Purple	Yellow	
38	G. max	Milyang216		CD-1220	South Korea	Improved line	Purple	Yellow	
39	G. max	Milyang232		CD-1221	South Korea	Improved line	White	Yellow	
40	G. max	Miyang242		CD-1222	South Korea	Improved line	White	Yellow	
41	G. max	Wongwang		CD-1226	South Korea	Improved line	Purple	Yellow	
42	G. max	Wonheuk		CD-1227	South Korea	Improved line	Purple	Black	
43	G. max	Joyang		CD-1228	South Korea	Improved line	Purple	Yellow	
44	G. max	Eunha		CD-1231	South Korea	Improved line	Purple	Yellow	
45	G. max	Namhae		CD-1232	South Korea	Improved line	White	Yellow	
46	G. max	Bukwang		CD-1233	South Korea	Improved line	White	Yellow	
47	G. max	Gwangan		CD-1234	South Korea	Improved line	Purple	Yellow	
48	G. max	Pureun-3		CD-1235	South Korea	Improved line	White	Green	
	G. max	Pureun		poolnkong2	South Korea	Improved line			
	G. max	Pureun		CD-1343	South Korea	Improved line			
49	G. max	Myeongjunamul		CD-1237	South Korea	Improved line	Purple	Yellow	
50	G. max	Iksannamul		CD-1238	South Korea	Improved line	White	Yellow	
51	G. max	Sobaeknamul		CD-1239	South Korea	Improved line	Purple	Yellow	
52	G. max	Dawon		CD-1240	South Korea	Improved line	Purple	Black	
53	G. max	Paldonamul		CD-1242	South Korea	Improved line	Purple	Yellow	

54	G. max	Doremi		CD-1244	South Korea	Improved line	White	Yellow	
55	G. max	Bangsa		CD-1245	South Korea	Improved line	White	Yellow	
	G. max	Bangsa		Dooyou	South Korea	Improved line			DNA mislabelled
	G. max	Bangsa		Bangsa	South Korea	Improved line			
56	G. max	Saebyeol		CD-1246	South Korea	Improved line	Purple	Yellow	
57	G. max	Sorok		CD-1247	South Korea	Improved line	Purple	Green yellow	
58	G. max	Anpyeong		CD-1248	South Korea	Improved line	Purple	Yellow	
59	G. max	Sunam		CD-1249	South Korea	Improved line	Purple	Yellow	
60	G. max	Dagi		CD-1250	South Korea	Improved line	Purple	Yellow	
61	G. max	Dachae		CD-1251	South Korea	Improved line	White	Yellow	
62	G. max	Sojin		CD-1252	South Korea	Improved line	Purple	Yellow	
63	G. max	Bosug		CD-1253	South Korea	Improved line	Purple	Yellow	
64	G. max	Sogang		CD-1254	South Korea	Improved line	White	Yellow	
65	G. max	Nokchae		CD-1255	South Korea	Improved line	White	Green	
66	G. max	Wonhwang		CD-1256	South Korea	Improved line	Purple	Yellow	
	G. max	CS90		EE-1274	China	Improved line			
67	G. max	Janggi		CD-1257	South Korea	Improved line	Purple	Yellow	
68	G. max	Jonam		CD-1258	South Korea	Improved line	Purple	Yellow	
69	G. max	Hoseo		CD-1259	South Korea	Improved line	Purple	Yellow	
70	G. max	Galchae		CD-1261	South Korea	Improved line	Purple	Brown	
	G. max	Galchae		Galchae	South Korea	Improved line			
71	G. max	Sohwang		CD-1262	South Korea	Improved line	Purple	Yellow	
72	G. max	Socheong		CD-1264	South Korea	Improved line	White	Black	
73	G. max	Cheongja		CD-1265	South Korea	Improved line	Purple	Black	
74	G. max	Cheongja3		CD-1266	South Korea	Improved line	Purple	Black	
75	G. max	Cheongdu1		CD-1267	South Korea	Improved line	White	Green	
76	G. max	Heukseong		CD-1269	South Korea	Improved line	Purple	Black	
77	G. max	Geomjeong5		CD-1270	South Korea	Improved line	Purple	Black	
78	G. max	Cheongyeop1		CD-1271	South Korea	Improved line	White	Black	
79	G. max	Geomjeong1		CD-1272	South Korea	Improved line	White	Black	
80	G. max	Geomjeong2		CD-1273	South Korea	Improved line	Purple	Black	
81	G. max	Jinyul		CD-1275	South Korea	Improved line	Purple	Brown	
82	G. max	Geomjeong3		CD-1276	South Korea	Improved line	Purple	Black	
83	G. max	Geomjeong4		CD-1277	South Korea	Improved line	Purple	Black	
84	G. max	Cheongja2		CD-1278	South Korea	Improved line	Purple	Black	
85	G. max	Ilpumgeomjeong2		CD-1279	South Korea	Improved line	Purple	Black	
	G. max	Hwangkeumol		CD-1287	South Korea	Improved line			
86	G. max	Heukmi		CD-1280	South Korea	Improved line	Purple	Black	
87	G. max	Seonheuk		CD-1281	South Korea	Improved line	Purple	Black	
88	G. max	Danmi		CD-1283	South Korea	Improved line	Purple	Green	
89	G. max	Nogwon		CD-1284	South Korea	Improved line	White	Green	
90	G. max	Seaol		CD-1285	South Korea	Improved line	White	Yellow	
91	G. max	Hanol		CD-1286	South Korea	Improved line	White	Yellow	
92	G. max	Daol		CD-1288	South Korea	Improved line	White	Yellow	
93	G. max	Geomjeongsaeol		CD-1289	South Korea	Improved line	Purple	Black	
94	G. max	Shillog		CD-1292	South Korea	Improved line	Purple	Yellow	
95	G. max	Seonrok		CD-1293	South Korea	Improved line	White	Green yellow	
96	G. max	Dajin		CD-1294	South Korea	Improved line	White	Green yellow	
97	G. max	Mirang		CD-1295	South Korea	Improved line	White	Black	
98	G. max	Danmi2		CD-1296	South Korea	Improved line	White	Green	
99	G. max	Chamol		CD-1297	South Korea	Improved line	White	Yellow	
	G. max	Milyang210	IT237832	DD-1386	South Korea	Improved line			
100	G. max	Maple presto	PI 548594	CD-1298	United States	Improved line	Purple	Yellow	
101	G. max	Fiskeby 840-7-3	PI 438477	CD-1299	Sweden	Improved line	Purple	Yellow	
102	G. max	OT94-49	PI 591431	CD-1306	Canada	Improved line	Purple	Yellow	
103	G. max	Blackhawk	PI 548516	CD-1310	United States	Improved line	White	Yellow	
104	G. max	L67-153	PI 547729	CD-1311	United States	Improved line	Purple	Yellow	
105	G. max	L62-667	PI 547716	CD-1312	United States	Improved line	Purple	Yellow	
	G. max	OT94-51	PI 591432	CD-1307	Canada	Improved line			
	G. max	Harosoy	PI 548573	DD-1427	Canada	Improved line			
	G. max	OT89-05	PI 546043	CD-1300	Canada	Improved line			
	G. max	OT89-06	PI 546044	CD-1301	Canada	Improved line			
	G. max	OT93-26	PI 591429	CD-1302	Canada	Improved line			
	G. max	OT93-28	PI 591430	CD-1303	Canada	Improved line			
	G. max	OT94-37	PI 591433	CD-1304	Canada	Improved line			
	G. max	OT94-39	PI 591434	CD-1305	Canada	Improved line			
106	G. max	Adelphia	PI 548503	CD-1345	United States	Improved line	White	Yellow	

107	G. max	Bancheongdu	IT230769	CD-1346	South Korea	Landrace	Purple	Brown	Representative
108	G. max	Babmit	IT021574	CD-1347	South Korea	Landrace	Purple	Yellow	
109	G. max	Boone	PI 548309	CD-1349	China	Improved line	White	Yellow	
110	G. max	Jogyemyeongtae	IT021585	CD-1350	South Korea	Landrace	Purple	Green	
	G. max	Leekamkwn	IT230697	DD-1335	South Korea	Landrace			
111	G. max	Danchwondamrok	IT021607	CD-1353	South Korea	Landrace	Purple	Yellow	
112	G. max	KLS 119-1	PI 398876	CD-1355	South Korea	Landrace	Purple	Yellow	
113	G. max	Ikdonjaerae	IT021662	CD-1356	South Korea	Landrace	Purple	Yellow	
	G. max	Ichunjaelae	IT230735	EE-1178	South Korea	Landrace			
114	G. max	K.L.S 739-2	IT021695	CD-1358	South Korea	Landrace	White	Yellow	
	G. max	KAS230-15	IT115426	DD-0289	South Korea	Landrace			
	G. max	YN131-4	IT023870	EE-0075	South Korea	Landrace			
	G. max	Suweon 27	IT230764	EE-1191	South Korea	Improved line			
	G. max	Heuin	IT021646	CD-1354	South Korea	Landrace			
115	G. max	K.L.S 803-2	IT021696	CD-1359	South Korea	Landrace	Purple	Yellow	
116	G. max	Gyeongnam1	IT021704	CD-1360	South Korea	Landrace	White	Yellow	
117	G. max	Mack	PI 559370	CD-1361	United States	Improved line	Purple	Green yellow	
118	G. max	Nongrim5	IT021727	CD-1362	Japan	Improved line	White	Yellow	
119	G. max	Oealkong	IT021728	CD-1363	South Korea	Landrace	Purple	Yellow	
120	G. max	Perry	PI 548603	CD-1364	United States	Improved line	Purple	Yellow	
121	G. max	Pomana	IT021734	CD-1365	United States	Improved line	Purple	Yellow	
122	G. max	PI 68430		CD-1366	China	Not determined	Purple	Yellow	
123	G. max	Suwon116	IT021748	CD-1368	South Korea	Improved line	White	Yellow	
124	G. max	PI 196168		CD-1370	South Korea	Landrace	Purple	Yellow	
125	G. max	Sugae29	IT021772	CD-1373	South Korea	Improved line	White	Yellow	
126	G. max	Kwanggyo	IT021773	CD-1374	South Korea	Improved line	Purple	Yellow	
127	G. max	Sugae34	IT021776	CD-1375	South Korea	Improved line	White	Yellow	
		Sugae34	IT023251	EE-0059	South Korea	Improved line			
128	G. max	Sugae35	IT021777	CD-1376	South Korea	Improved line	White	Yellow	
129	G. max	Sugae47	IT021783	CD-1380	South Korea	Improved line	Purple	Yellow	
	G. max	Ham-nam	IT022285	DD-0007	South Korea	Landrace			
	G. max	KLS740	PI 399010	DD-0041	South Korea	Landrace			
	G. max	KLS827	IT022682	DD-0046	South Korea	Landrace			
	G. max	KAS624-24	IT141964	DD-0451	South Korea	Landrace			
	G. max	KAS601-1	IT154392	EE-0501	South Korea	Landrace			
	G. max	PI 458294		EE-1047	South Korea	Landrace			
	G. max	Haman-dailip	IT022286	EE-0010	South Korea	Landrace			
	G. max	Gyungdu	IT162656	EE-0678	South Korea	Landrace			
	G. max	KLS 739	IT230828	EE-1210	South Korea	Landrace			
	G. max	Ham ahn	PI 406708	EE-0990	South Korea	Landrace			
	G. max	Sugae47	IT023259	EE-0060	South Korea	Improved line			
	G. max	Heamnam	IT230698	EE-1165	South Korea	Landrace			
	G. max	PI 95801		DD-0759	South Korea	Landrace			
	G. max	YJ143-1	IT024053	DD-0106	South Korea	Landrace			
	G. max	KAS633-3	PI 408089	DD-0595	South Korea	Landrace			
	G. max	Kyongdu	IT230757	DD-1346	South Korea	Landrace			
	G. max	Crawford	PI 548541	CD-1351	United States	Improved line			
	G. max	PI 157410		CD-1369	South Korea	Landrace			
130	G. max	Sugae51	IT021785	CD-1381	South Korea	Improved line	Purple	Yellow	
	G. max	Sugae51	IT021791	CD-1387	South Korea	Improved line			
131	G. max	Sugae52	IT021786	CD-1382	South Korea	Improved line	Purple	Yellow	
	G. max	Sugae52	IT023262	EE-0062	South Korea	Improved line			
132	G. max	Sugae53	IT021787	CD-1383	South Korea	Improved line	Purple	Yellow	
	G. max	Sugae53	IT023263	EE-0063	South Korea	Improved line			
133	G. max	Sugae55	IT021788	CD-1384	South Korea	Improved line	Purple	Yellow	
	G. max	Sugae55	IT023264	EE-0064	South Korea	Improved line			
134	G. max	Sugae56	IT021789	CD-1385	South Korea	Improved line	Purple	Yellow	
135	G. max	Sugae57	IT021790	CD-1386	South Korea	Improved line	Purple	Yellow	
136	G. max	Dusan6	IT021799	CD-1388	South Korea	Improved line	White	Yellow	
137	G. max	Dusan7	IT021800	CD-1389	South Korea	Improved line	Purple	Yellow	
138	G. max	Dusan61	IT021801	CD-1390	South Korea	Improved line	Purple	Yellow	
139	G. max	Dusan69	IT021802	CD-1391	South Korea	Improved line	Purple	Yellow	
	G. max	Mokpo1	IT024717	DD-0154	South Korea	Improved line			
140	G. max	Dusan73	IT021804	CD-1393	South Korea	Improved line	White	Yellow	
141	G. max	Dusan74	IT021805	CD-1394	South Korea	Improved line	White	Yellow	
	G. max	Dusan74	IT021672	CD-1357	South Korea	Improved line			
	G. max	Dusan70	IT021803	CD-1392	South Korea	Improved line			

142	G. max	Dusan75	IT021806	CD-1395	South Korea	Improved line	Purple	Yellow	
143	G. max	Dusan83	IT021807	CD-1396	South Korea	Improved line	Purple	Green yellow	
144	G. max	Wabash	PI 548626	CD-1397	United States	Improved line	White	Yellow	
145	G. max	Woodsworth	IT021831	CD-1398	United States	Improved line	White	Yellow	
146	G. max	Ulsantae	IT021832	CD-1399	South Korea	Landrace	White	Yellow	
147	G. max	Yugwu16	IT021836	CD-1400	Japan	Improved line	Purple	Yellow	
	G. max	Donggil	IT025356	EE-0191	South Korea	Landrace			
	G. max	Yuku25	IT021837	CD-1401	Japan	Improved line			
148	G. max	Cheongja		Chungja	South Korea	Improved line	Purple	Black	
149	G. max	Cheongja3		Chungja3	South Korea	Improved line	Purple	Black	
150	G. max	Daepung		Daepoong	South Korea	Improved line	White	Yellow	
	G. max	Daepung		CD-1167	South Korea	Improved line			
151	G. max	Daewon		DaeWon	South Korea	Improved line	White	Yellow	
	G. max	Daewon		CD-1164	South Korea	Improved line			
152	G. max	SLSN293-2	IT022016	DD-0003	South Korea	Landrace	Purple	Yellow	
	G. max	KAS631-38	IT115777	EE-0291	South Korea	Landrace			
153	G. max	KLS 901-2	IT022067	DD-0004	South Korea	Landrace	Purple	Yellow	
154	G. max	Davis	PI 553039	DD-0006	United States	Improved line	-	Yellow	
155	G. max	Kantou #44	IT022368	DD-0008	Japan	Improved line	Purple	Yellow	
156	G. max	KLS101-2	PI 398866	DD-0009	South Korea	Landrace	Purple	Yellow	
157	G. max	KLS105	IT022396	DD-0010	South Korea	Landrace	Purple	Yellow	
	G. max	KLS87115	IT153732	DD-0548	South Korea	Landrace			
158	G. max	KLS117	PI 398875	DD-0011	South Korea	Landrace	Purple	Brown	
	G. max	KLS124-1	IT022434	EE-0013	South Korea	Landrace			
159	G. max	KLS119-2	IT022413	DD-0012	South Korea	Landrace	Purple	Yellow	
160	G. max	KLS123-1	PI 398879	DD-0013	South Korea	Landrace	Purple	Brown	
	G. max	KAS 134-6	PI 424233	EE-1134	South Korea	Landrace			
161	G. max	KLS125-1	PI 398880	DD-0014	South Korea	Landrace	White	Yellow	
162	G. max	KLS127	PI 398882	DD-0016	South Korea	Landrace	White	Yellow	
163	G. max	KLS205-1	PI 398892	DD-0018	South Korea	Landrace	Purple	Yellow	Representative
	G. max	KLS204	IT022445	EE-0017	South Korea	Landrace			
164	G. max	KLS215	IT022461	DD-0020	South Korea	Landrace	White	Yellow	
165	G. max	KLS411-1	PI 398926	DD-0021	South Korea	Landrace	Purple	Black	
166	G. max	KLS419	IT022505	DD-0023	South Korea	Landrace	Purple	Yellow	
	G. max	KLS87180	IT153785	EE-0464	South Korea	Landrace			
	G. max	Jangdanbaekmok	IT230740	DD-1131	South Korea	Landrace			
	G. max	KAS 351-25	IT226834	DD-1175	South Korea	Landrace			
	G. max	PI 458072 A		DD-1185	South Korea	Landrace			
	G. max	Buseuk	IT230806	DD-1357	South Korea	Landrace			
	G. max	KLS713-1	IT022592	DD-0034	South Korea	Landrace			
	G. max	KAS544-31	PI 458194	DD-0426	South Korea	Landrace			
	G. max	KAS624-30	PI 424541	DD-0452	South Korea	Landrace			
	G. max	KAS630-35	IT142033	DD-0454	South Korea	Landrace			
	G. max	KLS86089	IT142858	DD-0464	South Korea	Landrace			
	G. max	KLS87047	IT153673	EE-0454	South Korea	Landrace			
	G. max	YJ55-4	IT024629	EE-0141	South Korea	Landrace			
	G. max	Baekjung 47	IT230733	EE-1177	South Korea	Landrace			
	G. max	Packjung #48	IT230813	EE-1206	South Korea	Not determined			
	G. max	IT115188		EE-0251	South Korea	Landrace			
	G. max	KAS181-7	PI 398325	EE-0815	South Korea	Landrace			
	G. max	Booseuk	IT230716	EE-1171	South Korea	Landrace			
	G. max	Juinunkong	IT230744 (Dealip)	EE-1182	South Korea	Landrace			
	G. max	KAS581-28	IT141846	EE-0362	South Korea	Landrace			
	G. max	KAS 540-18	PI 424424	DD-1177	South Korea	Landrace			
	G. max	KAS 102-1-1	IT229951	DD-1309	South Korea	Landrace			
	G. max	PI 399093		DD-1318	South Korea	Landrace			
	G. max	Baekjung 42	IT230732	DD-1341	South Korea	Landrace			
	G. max	KAS631-8	IT154458	DD-0589	South Korea	Landrace			
	G. max	Dancheon	IT021606	CD-1352	South Korea	Landrace			
	G. max	Sugae2	IT021767	CD-1372	South Korea	Improved line			
	G. max	YB192-3	IT024424	DD-0125	South Korea	Landrace			
	G. max	IT103631		DD-0230	South Korea	Landrace			
	G. max	KAS524-19	IT115635	DD-0312	South Korea	Landrace			
	G. max	KAS580-11	IT115740	DD-0329	South Korea	Landrace			
	G. max	KAS651-33	IT115858	DD-0340	South Korea	Landrace			
	G. max	KAS679-27	IT141690	DD-0418	South Korea	Landrace			

	G. max	KAS523-10	IT141695	DD-0420	South Korea	Landrace			
	G. max	KAS607-3	IT141896	DD-0439	South Korea	Landrace			
	G. max	KLS409	IT022491	EE-0020	South Korea	Landrace			
	G. max	Suwon101	IT024225	EE-0101	South Korea	Improved line			
	G. max	PI 458163		EE-1031	South Korea	Landrace			
	G. max	Juinun-Kong	IT022353	EE-0012	South Korea	Landrace			
	G. max	YJ9	IT023982	EE-0084	South Korea	Landrace			
	G. max	KLS418	IT022504	EE-0022	South Korea	Landrace			
	G. max	KAS634-18	IT154524	EE-0510	South Korea	Landrace			
	G. max	KAS631-7	IT142039	EE-0380	South Korea	Landrace			
	G. max	KAS640-44	IT115821	EE-0296	South Korea	Landrace			
	G. max	Suweon 2	IT230760	EE-1188	South Korea	Improved line			
167	G. max	KLS505-3	IT022511	DD-0024	South Korea	Landrace	Purple	Yellow	
168	G. max	KLS 116-1	PI 398874	DD-0025	South Korea	Landrace	White	Black	
	G. max	Yuwoltae)	IT226762	DD-1165	South Korea	Landrace			
	G. max	Suwon36	IT142813	DD-0458	South Korea	Improved line			
169	G. max	KLS607-3	PI 398946	DD-0027	South Korea	Landrace	White	Yellow	
170	G. max	KLS632-1	IT022572	DD-0029	South Korea	Landrace	Purple	Black	
171	G. max	KLS701-2	IT022575	DD-0030	South Korea	Landrace	Purple	Yellow	
172	G. max	KLS702-1	PI 398977	DD-0031	South Korea	Landrace	Purple	Yellow	
173	G. max	KLS706	IT022585	DD-0032	South Korea	Landrace	Purple	Green	
174	G. max	KLS711-1	PI 398983	DD-0033	South Korea	Landrace	Purple	Yellow	
175	G. max	KLS714-2	IT022595	DD-0035	South Korea	Landrace	Purple	Yellow	
176	G. max	KLS723-1	PI 398993	DD-0037	South Korea	Landrace	White	Yellow	
177	G. max	KLS724-1	PI 398994	DD-0038	South Korea	Landrace	Purple	Yellow	
178	G. max	KLS736-3	IT022625	DD-0040	South Korea	Landrace	Purple	Yellow	
179	G. max	KLS741	PI 399011	DD-0042	South Korea	Landrace	Purple	Yellow	
	G. max	KAS 680-4	IT226823	EE-0999	South Korea	Landrace			
180	G. max	KLS804-1	IT022645	DD-0044	South Korea	Landrace	White	Yellow	
181	G. max	KLS806-1	IT022650	DD-0045	South Korea	Landrace	Purple	Yellow	
182	G. max	Lindarin #63	PI 548590	DD-0047	United States	Improved line	Purple	Yellow	
183	G. max	Century 84	PI 548529	DD-0048	United States	Improved line	Purple	Yellow	
184	G. max	Norchief	PI 548601	DD-0049	United States	Improved line	Purple	Yellow	
	G. max	KAS590-1	IT141849	DD-0433	South Korea	Landrace			
	G. max	KLS904-2	IT022687	EE-0050	South Korea	Landrace			
	G. max	PI 399045		DD-1073	South Korea	Landrace			
	G. max	KAS548-10	PI 408017	DD-1082	South Korea	Landrace			
185	G. max	PI 70213		DD-0051	China	Not determined	Purple	Yellow	
186	G. max	PI 79628		DD-0053	China	Not determined	Purple	Yellow	
187	G. max	PI 91163		DD-0056	China	Not determined	White	Yellow	Representative
188	G. max	PI 200503		DD-0057	Japan	Not determined	White	Yellow	
189	G. max	PI 243517		DD-0058	Japan	Not determined	White	Brown	
190	G. max	YN4-2	IT023798	DD-0064	South Korea	Landrace	Purple	Yellow	
191	G. max	YN23-4	IT023802	DD-0065	South Korea	Landrace	Purple	Yellow	
192	G. max	YN48	IT023813	DD-0067	South Korea	Landrace	Purple	Brown	
193	G. max	YN69-2	IT023822	DD-0069	South Korea	Landrace	Purple	Yellow	
194	G. max	YN85-3	IT023836	DD-0071	South Korea	Landrace	Purple	Green yellow	
195	G. max	YJ41	IT023839	DD-0072	South Korea	Landrace	Purple	Yellow	
196	G. max	YN108-4	IT023847	DD-0073	South Korea	Landrace	Purple	Green	
197	G. max	YN109-1	IT023848	DD-0074	South Korea	Landrace	White	Green	
198	G. max	YN122-4	IT023864	DD-0075	South Korea	Landrace	Purple	Yellow	
199	G. max	YN152-1	IT023885	DD-0077	South Korea	Landrace	White	Brown	
200	G. max	YN154	IT023887	DD-0078	South Korea	Landrace	Purple	Yellow	
201	G. max	YN162	IT023896	DD-0080	South Korea	Landrace	Purple	Yellow	
202	G. max	YN174	IT023906	DD-0081	South Korea	Landrace	Purple	Green	
203	G. max	YN175-3	IT023908	DD-0082	South Korea	Landrace	Purple	Yellow	
204	G. max	YN178-3	IT023913	DD-0085	South Korea	Landrace	Purple	Yellow	
205	G. max	YN180-3	IT023918	DD-0086	South Korea	Landrace	Purple	Green	
206	G. max	YN188-3	IT023925	DD-0087	South Korea	Landrace	Purple	Green	
207	G. max	YN191-4	IT023932	DD-0088	South Korea	Landrace	White	Green	
208	G. max	YN201-4	IT023939	DD-0089	South Korea	Landrace	White	Yellow	
209	G. max	YJ73-1	IT023945	DD-0091	South Korea	Landrace	Purple	Yellow	
210	G. max	YN213-2	IT023953	DD-0092	South Korea	Landrace	Purple	Green	
211	G. max	YN220-3	IT023963	DD-0093	South Korea	Landrace	White	Green	
212	G. max	YN228-1	IT023971	DD-0094	South Korea	Landrace	Purple	Yellow	
213	G. max	YJ16-4	IT023990	DD-0096	South Korea	Landrace	White	Yellow	
214	G. max	YJ19-1	IT023991	DD-0097	South Korea	Landrace	Purple	Yellow	

215	G. max	YJ22-1	IT023993	DD-0098	South Korea	Landrace	Purple	Yellow
216	G. max	YJ88	IT024016	DD-0100	South Korea	Landrace	Purple	Yellow
217	G. max	YJ107-3	IT024027	DD-0102	South Korea	Landrace	Purple	Yellow
	G. max	YJ77-1	IT024640	EE-0143	South Korea	Landrace		
218	G. max	YJ108-1	IT024029	DD-0103	South Korea	Landrace	White	Green yellow
219	G. max	YJ113-1	IT024035	DD-0104	South Korea	Landrace	White	Brown
220	G. max	YJ138-4	IT024051	DD-0105	South Korea	Landrace	Purple	Yellow
	G. max	KAS633-9	IT154502	DD-0596	South Korea	Landrace		
	G. max	YN55-4	IT023815	DD-0068	South Korea	Landrace		
221	G. max	YJ153-1	IT024056	DD-0107	South Korea	Landrace	White	Yellow
222	G. max	YJ190-4	IT024085	DD-0108	South Korea	Landrace	White	Yellow
223	G. max	YJ196-1	IT024088	DD-0109	South Korea	Landrace	Purple	Yellow
224	G. max	YJ225-1	IT024122	DD-0112	South Korea	Landrace	Purple	Yellow
225	G. max	YJ229-3	IT024128	DD-0113	South Korea	Landrace	Purple	Yellow
226	G. max	YN48	IT023813	DD-0114	South Korea	Landrace	Purple	Black
227	G. max	YJ60-3	IT024154	DD-0115	South Korea	Landrace	Purple	Yellow
228	G. max	YJ85-2	IT024163	DD-0116	South Korea	Landrace	White	Yellow
229	G. max	YJ91-3	IT024169	DD-0117	South Korea	Landrace	Purple	Yellow
230	G. max	YJ179-1	IT024179	DD-0118	South Korea	Landrace	White	Yellow
231	G. max	Suwon82	IT024208	DD-0119	South Korea	Improved line	White	Green yellow
232	G. max	Suwon98	IT024222	DD-0120	South Korea	Improved line	Purple	Yellow
233	G. max	Changdan-baekmok	IT022171	DD-0121	South Korea	Landrace	White	Yellow
234	G. max	YB297-2	IT024391	DD-0122	South Korea	Landrace	Purple	Black
235	G. max	YB316-3	IT024393	DD-0123	South Korea	Landrace	Purple	Black
	G. max	KAS631-12	IT142042	EE-0381	South Korea	Landrace		
236	G. max	YJ85-1	IT024399	DD-0124	South Korea	Landrace	White	Yellow
237	G. max	YN197-1	IT024431	DD-0127	South Korea	Landrace	Purple	Brown
238	G. max	KAS370-26	PI 458155	DD-0128	South Korea	Landrace	Purple	Yellow
239	G. max	Suwon103	IT024470	DD-0129	South Korea	Improved line	White	Yellow
240	G. max	Suwon59-2	IT024497	DD-0131	South Korea	Improved line	Purple	Yellow
241	G. max	YN75-2	IT024514	DD-0132	South Korea	Landrace	Purple	Yellow
242	G. max	YN89-2	IT024524	DD-0134	South Korea	Landrace	Purple	Green
243	G. max	Milyang1	IT024533	DD-0136	South Korea	Improved line	Purple	Green yellow
244	G. max	YN148-3	IT024562	DD-0139	South Korea	Landrace	Purple	Yellow
245	G. max	YN168-4	IT024574	DD-0140	South Korea	Landrace	Purple	Green
246	G. max	YN210-4	IT024595	DD-0141	South Korea	Landrace	Purple	Brown
247	G. max	YN221-2	IT024601	DD-0142	South Korea	Landrace	Purple	Green yellow
248	G. max	Milyang6	IT024603	DD-0143	South Korea	Improved line	Purple	Yellow
249	G. max	Milyang10	IT024622	DD-0144	South Korea	Improved line	White	Yellow
250	G. max	YJ93-3	IT024648	DD-0147	South Korea	Landrace	Purple	Yellow
	G. max	YJ93-4	IT024649	DD-0148	South Korea	Landrace		
251	G. max	YJ98-4	IT024652	DD-0149	South Korea	Landrace	Purple	Yellow
252	G. max	YJ120-3	IT024668	DD-0150	South Korea	Landrace	Purple	Yellow
253	G. max	Hourai	PI 416940	DD-0151	Japan	Improved line	Purple	Yellow
254	G. max	YJ165-4	IT024698	DD-0152	South Korea	Landrace	Purple	Yellow
255	G. max	YJ177-3	IT024711	DD-0153	South Korea	Landrace	Purple	Yellow
256	G. max	YJ207-1	IT024738	DD-0155	South Korea	Landrace	Purple	Yellow
257	G. max	YJ223-3	IT024746	DD-0156	South Korea	Landrace	Purple	Yellow
258	G. max	YJ232-2	IT024757	DD-0158	South Korea	Landrace	Purple	Yellow
259	G. max	KAS700-42	IT024777	DD-0160	South Korea	Landrace	Purple	Yellow
260	G. max	YJ256-1	IT024790	DD-0162	South Korea	Landrace	White	Yellow
261	G. max	YJ258-4	IT024794	DD-0163	South Korea	Landrace	White	Green yellow
262	G. max	YJ268	IT024805	DD-0164	South Korea	Landrace	Purple	Green yellow
263	G. max	YJ284-1	IT024826	DD-0165	South Korea	Landrace	Purple	Black
264	G. max	YJ296-2	IT024841	DD-0167	South Korea	Landrace	Purple	Yellow
265	G. max	YJ302-1	IT024848	DD-0168	South Korea	Landrace	Purple	Brown
	G. max	YB25-3	IT024872	DD-0172	South Korea	Landrace		
266	G. max	YB19-3	IT024864	DD-0170	South Korea	Landrace	Purple	Black
267	G. max	YB27	IT024874	DD-0173	South Korea	Landrace	White	Black
268	G. max	YB31-3	IT024878	DD-0174	South Korea	Landrace	Purple	Yellow
269	G. max	YB44	IT024890	DD-0175	South Korea	Landrace	Purple	Brown
270	G. max	YB77	IT024923	DD-0176	South Korea	Landrace	Purple	Brown
271	G. max	YB132-4	IT024958	DD-0177	South Korea	Landrace	Purple	Black
	G. max	Geomeunbabmit	IT160624	EE-0585	South Korea	Landrace		
272	G. max	YB154	IT024983	DD-0178	South Korea	Landrace	Purple	Brown
273	G. max	YB182-4	IT025015	DD-0179	South Korea	Landrace	Purple	Black

274	G. max	YB193-3	IT025031	DD-0180	South Korea	Landrace	Purple	Black	Representative
275	G. max	YB199	IT025040	DD-0181	South Korea	Landrace	Purple	Black	
	G. max	NA 56704	PI 508298	EE-1118	South Korea	Landrace			
276	G. max	YB201-2	IT025042	DD-0182	South Korea	Landrace	Purple	Black	
277	G. max	YB213-4	IT025057	DD-0184	South Korea	Landrace	Purple	Green	
278	G. max	YB233-3	IT025082	DD-0186	South Korea	Landrace	Purple	Green	
279	G. max	YB244	IT025089	DD-0188	South Korea	Landrace	Purple	Green	
280	G. max	YB262-4	IT025099	DD-0189	South Korea	Landrace	Purple	Black	
281	G. max	YB293-2	IT025123	DD-0191	South Korea	Landrace	Purple	Green	
282	G. max	YB302	IT025131	DD-0192	South Korea	Landrace	Purple	Brown	
283	G. max	YB303	IT025132	DD-0193	South Korea	Landrace	Purple	Green	
284	G. max	YB331-3	IT025158	DD-0195	South Korea	Landrace	Purple	Black	
	G. max	KLS77134-2	IT025376	DD-0214	South Korea	Landrace			
285	G. max	KLS77016	IT025219	DD-0199	South Korea	Landrace	Purple	Brown	
286	G. max	KLS77042	IT025246	DD-0202	South Korea	Landrace	Purple	Green	
287	G. max	KLS77048	IT025253	DD-0203	South Korea	Landrace	Purple	Yellow	
	G. max	KLS414	IT022497	DD-0022	South Korea	Landrace			
	G. max	YJ212-2	IT024105	DD-0111	South Korea	Landrace			
	G. max	YJ234-4	IT024763	DD-0159	South Korea	Landrace			
	G. max	IT113394		EE-0248	South Korea	Landrace			
	G. max	KLS87106	IT153723	DD-0545	South Korea	Landrace			
	G. max	KAS511-11	IT154252	DD-0574	South Korea	Landrace			
	G. max	Hongseonggun	IT162682	DD-0774	South Korea	Landrace			
	G. max	SLS90-194	IT167832	DD-0819	South Korea	Landrace			
	G. max	KAS 351-26	PI 424358	DD-1176	South Korea	Landrace			
	G. max	PI 458123 A		DD-1193	South Korea	Landrace			
288	G. max	KLS77056	IT025261	DD-0204	South Korea	Landrace	White	Green	
289	G. max	KLS77064	IT025269	DD-0205	South Korea	Landrace	Purple	Brown	
	G. max	KLS77005-1	IT024756	DD-0157	South Korea	Landrace			
	G. max	KLS77003-4	IT025201	DD-0198	South Korea	Landrace			
290	G. max	KLS77069	IT025273	DD-0206	South Korea	Landrace	Purple	Black	
291	G. max	KLS77098-1	IT025309	DD-0208	South Korea	Landrace	White	Yellow	
292	G. max	KLS77109-3	IT025324	DD-0209	South Korea	Landrace	Purple	Black	
293	G. max	KLS77109-4	IT025325	DD-0210	South Korea	Landrace	White	Yellow	
294	G. max	KLS77114-1	IT025335	DD-0211	South Korea	Landrace	Purple	Black	
295	G. max	KLS77125-1	IT025353	DD-0212	South Korea	Landrace	Purple	Yellow	
296	G. max	KLS77131-2	IT025370	DD-0213	South Korea	Landrace	White	Yellow	
297	G. max	KLS140-3	IT025386	DD-0215	South Korea	Landrace	Purple	Green	
298	G. max	KLS141	IT025388	DD-0216	South Korea	Landrace	Purple	Black	
	G. max	SLSB-B-19	IT134471	DD-0386	South Korea	Landrace			
299	G. max	KLS77191-1	IT025391	DD-0217	South Korea	Landrace	Purple	Yellow	
300	G. max	KLS77213-4	IT025398	DD-0218	South Korea	Landrace	Purple	Black	
301	G. max	KLS77214-3	IT025407	DD-0219	South Korea	Landrace	White	Yellow	
302	G. max	KLS77170	IT025435	DD-0220	South Korea	Landrace	Purple	Yellow	
303	G. max	KLS77207-1	IT025500	DD-0221	South Korea	Landrace	White	Yellow	
304	G. max	Yuwol	IT101020	DD-0222	South Korea	Landrace	White	Yellow	
305	G. max	Jaeraeul	IT101025	DD-0223	South Korea	Landrace	White	Yellow	
306	G. max	Yuwol	IT101061	DD-0224	South Korea	Landrace	White	Yellow	
307	G. max	Yeoreum	IT101110	DD-0225	South Korea	Landrace	White	Yellow	
308	G. max	Pureun	IT102743	DD-0226	South Korea	Landrace	Purple	Green	
309	G. max	Geomjeong	IT103343	DD-0227	South Korea	Landrace	Purple	Black	
310	G. max	IT103415		DD-0228	South Korea	Landrace	Purple	Black	
311	G. max	Meju	IT104038	DD-0235	South Korea	Landrace	White	Yellow	
312	G. max	Nondureong	IT104075	DD-0236	South Korea	Landrace	Purple	Brown	
313	G. max	IT104334		DD-0237	South Korea	Landrace	White	Yellow	
314	G. max	Kongnamul	IT104356	DD-0238	South Korea	Landrace	White	Green	
315	G. max	IT104441		DD-0239	South Korea	Landrace	Purple	Brown	
316	G. max	Jilgeum	IT104553	DD-0240	South Korea	Landrace	Purple	Green	
	G. max	Hwangsaekjilgeum	IT104620	EE-0228	South Korea	Landrace			
317	G. max	IT104704		DD-0241	South Korea	Landrace	Purple	Yellow	
318	G. max	Daedu	IT104865	DD-0242	South Korea	Landrace	White	Green yellow	
319	G. max	IT104887		DD-0243	South Korea	Landrace	Purple	Green	
320	G. max	IT105237		DD-0244	South Korea	Landrace	Purple	Yellow	
321	G. max	IT105267		DD-0245	South Korea	Landrace	Purple	Green	
322	G. max	Meju	IT105278	DD-0247	South Korea	Landrace	Purple	Yellow	
323	G. max	Heuk	IT105442	DD-0248	South Korea	Landrace	Purple	Black	
324	G. max	Bul	IT105490	DD-0249	South Korea	Landrace	Purple	Yellow	

	G. max	kwangwon sujib1-16	IT156130	EE-0546	South Korea	Landrace		
325	G. max	IT105754		DD-0254	South Korea	Landrace	Purple	Black
326	G. max	Kongnamul	IT105773	DD-0255	South Korea	Landrace	White	Green
327	G. max	Gulgeun	IT105794	DD-0256	South Korea	Landrace	Purple	Yellow
328	G. max	Cheongsaeknamul	IT108679	DD-0257	South Korea	Landrace	Purple	Green
329	G. max	Meju	IT108810	DD-0258	South Korea	Landrace	Purple	Yellow
330	G. max	Geomeun	IT109118	DD-0260	South Korea	Landrace	Purple	Green
331	G. max	Ol	IT109170	DD-0261	South Korea	Landrace	White	Yellow
332	G. max	Cheong	IT109174	DD-0262	South Korea	Landrace	Purple	Green
333	G. max	Kongnamul	IT110990	DD-0264	South Korea	Landrace	Purple	Yellow
334	G. max	Kongnamul	IT111149	DD-0267	South Korea	Landrace	Purple	Brown
335	G. max	Bam	IT112768	DD-0268	South Korea	Landrace	Purple	Black
336	G. max	Yutae	IT112787	DD-0269	South Korea	Landrace	Purple	Yellow
337	G. max	IT112859		DD-0270	South Korea	Landrace	Purple	Black
	G. max	KLS87317	IT153894	EE-0480	South Korea	Landrace		
338	G. max	IT112926		DD-0271	South Korea	Landrace	Purple	Brown
	G. max	IT180333		EE-0799	South Korea	Landrace		
	G. max	IT186016		EE-0830	South Korea	Landrace		
	G. max	KLS087073	IT153386	DD-0535	South Korea	Landrace		
	G. max	Geomjeong	IT178220	DD-0924	South Korea	Landrace		
339	G. max	Heuksaek	IT113027	DD-0272	South Korea	Landrace	White	Black
340	G. max	Kongnamul	IT113082	DD-0273	South Korea	Landrace	Purple	Green
341	G. max	Cheongtae	IT113097	DD-0274	South Korea	Landrace	Purple	Green
342	G. max	IT113118		DD-0275	South Korea	Landrace	Purple	Brown
343	G. max	Ajukaribam	IT113138	DD-0276	South Korea	Landrace	Purple	Brown
344	G. max	Kongnamul	IT113421	DD-0277	South Korea	Landrace	Purple	Green
345	G. max	IT113573		DD-0278	South Korea	Landrace	Purple	Brown
346	G. max	IT113574		DD-0279	South Korea	Landrace	Purple	Green
347	G. max	Kongnamul	IT115171	DD-0280	South Korea	Landrace	Purple	Green yellow
348	G. max	KAS150-17-1	PI 424235	DD-0281	South Korea	Landrace	Purple	Brown
349	G. max	KAS150-22	IT115352	DD-0282	South Korea	Landrace	Purple	Green
350	G. max	KAS150-30	IT115353	DD-0283	South Korea	Landrace	White	Yellow
351	G. max	KAS170-2	IT115360	DD-0284	South Korea	Landrace	Purple	Brown
352	G. max	KAS171-7	IT115366	DD-0286	South Korea	Landrace	Purple	Green
	G. max	SLSB-B-64	IT134497	EE-0330	South Korea	Landrace		
353	G. max	KAS174-7	IT115383	DD-0287	South Korea	Landrace	White	Yellow
354	G. max	KAS174-9	IT115384	DD-0288	South Korea	Landrace	Purple	Green
355	G. max	KAS234-8	PI 458069	DD-0291	South Korea	Landrace	White	Black
356	G. max	KAS243-2	PI 458098	DD-0292	South Korea	Landrace	Purple	Brown
	G. max	PI 458098	IT226874	EE-1019	South Korea	Landrace		
357	G. max	KAS243-6	PI 458102	DD-0293	South Korea	Landrace	Purple	Green
358	G. max	KAS245-5	IT115481	DD-0294	South Korea	Landrace	Purple	Yellow
	G. max	YJ22-2	IT024610	EE-0140	South Korea	Landrace		
359	G. max	KAS245-10	IT115485	DD-0295	South Korea	Landrace	White	Yellow
360	G. max	KAS246-10	IT115496	DD-0296	South Korea	Landrace	Purple	Green
361	G. max	KAS301-21	IT115511	DD-0297	South Korea	Landrace	Purple	Brown
362	G. max	KAS302-5	PI 398675	DD-0298	South Korea	Landrace	Purple	Black
363	G. max	KAS331-12	IT115548	DD-0301	South Korea	Landrace	Purple	Brown
364	G. max	KAS351-4	PI 424336	DD-0302	South Korea	Landrace	Purple	Yellow
365	G. max	KAS351-20	PI 424352	DD-0303	South Korea	Landrace	Purple	Green
366	G. max	KAS352-9	PI 398780	DD-0304	South Korea	Landrace	Purple	Black
367	G. max	KAS360-7	IT115579	DD-0305	South Korea	Landrace	Purple	Black
368	G. max	KAS380-14	PI 398582	DD-0307	South Korea	Landrace	White	Yellow
369	G. max	KAS505-1	IT115608	DD-0309	South Korea	Landrace	Purple	Black
370	G. max	KAS510-6	IT115611	DD-0310	South Korea	Landrace	Purple	Green
371	G. max	KAS524-29	IT115638	DD-0313	South Korea	Landrace	Purple	Black
	G. max	KAS524-30	IT115639	DD-0314	South Korea	Landrace		
372	G. max	KAS530-20	IT115648	DD-0315	South Korea	Landrace	White	Yellow
373	G. max	KAS530-24	IT115650	DD-0316	South Korea	Landrace	Purple	Yellow
374	G. max	KAS530-31	IT115656	DD-0317	South Korea	Landrace	Purple	Brown
375	G. max	KAS530-39	IT115664	DD-0318	South Korea	Landrace	Purple	Yellow
376	G. max	KAS531-5	IT115665	DD-0319	South Korea	Landrace	Purple	Yellow
	G. max	KAS610-6	IT141908	EE-0371	South Korea	Landrace		
	G. max	YB88-3	IT024928	EE-0164	South Korea	Landrace		
	G. max	KAS612-2	IT141913	DD-0442	South Korea	Landrace		
377	G. max	KAS531-7	IT115667	DD-0320	South Korea	Landrace	Purple	Green

378	G. max	KAS551-10	IT115705	DD-0322	South Korea	Landrace	Purple	Yellow
	G. max	SLS B-3	IT158118	EE-0567	South Korea	Landrace		
379	G. max	KAS551-17	IT115709	DD-0323	South Korea	Landrace	White	Yellow
	G. max	SLSN144-2	IT134508	DD-0397	South Korea	Landrace		
380	G. max	KAS552-10	IT115713	DD-0324	South Korea	Landrace	Purple	Black
381	G. max	KAS560-12	PI 458205	DD-0325	South Korea	Landrace	White	Yellow
382	G. max	KAS560-14	PI 458207	DD-0326	South Korea	Landrace	Purple	Yellow
383	G. max	KAS571-23	IT115727	DD-0327	South Korea	Landrace	White	Green
384	G. max	KAS574-7	PI 458227	DD-0328	South Korea	Landrace	Purple	Yellow
	G. max	PI 458227		EE-1039	South Korea	Landrace		
385	G. max	KAS604-9	IT115754	DD-0330	South Korea	Landrace	Purple	Black
386	G. max	KAS625-19	IT115766	DD-0331	South Korea	Landrace	White	Green
387	G. max	KAS640-12	PI 424567	DD-0332	South Korea	Landrace	White	Yellow
388	G. max	KAS640-55	IT115823	DD-0335	South Korea	Landrace	Purple	Black
389	G. max	KAS642-11	IT115830	DD-0336	South Korea	Landrace	White	Green
390	G. max	KAS643-21	IT115831	DD-0337	South Korea	Landrace	White	Yellow
391	G. max	KAS646-23	IT115840	DD-0338	South Korea	Landrace	White	Yellow
392	G. max	KAS649-3	IT115845	DD-0339	South Korea	Landrace	Purple	Green
	G. max	YJ293-2	IT024836	EE-0161	South Korea	Landrace		
393	G. max	IT115870		DD-0341	South Korea	Landrace	Purple	Black
	G. max	YN160-2	IT023895	DD-0079	South Korea	Landrace		
394	G. max	KAS660-21	IT115872	DD-0342	South Korea	Landrace	Purple	Yellow
	G. max	Kongnamul	IT104096	EE-0221	South Korea	Landrace		
	G. max	KLS87087	IT153709	EE-0459	South Korea	Landrace		
	G. max	Daegu sujib	IT195632	EE-0859	South Korea	Landrace		
	G. max	YJ73-2	IT024637	EE-0142	South Korea	Landrace		
	G. max	Heuingaedari	IT180512	EE-0805	South Korea	Landrace		
	G. max	KAS362-6	IT219786	EE-0912	South Korea	Landrace		
	G. max	KAS660-23	IT218321	EE-0889	South Korea	Landrace		
	G. max	KLS087037	IT153369	DD-0531	South Korea	Landrace		
	G. max	KLS87080	IT153702	DD-0543	South Korea	Landrace		
	G. max	KAS504-4	IT154227	DD-0572	South Korea	Landrace		
	G. max	KAS660-2	IT154744	DD-0622	South Korea	Landrace		
	G. max	KAS549-3	IT218311	DD-1054	South Korea	Landrace		
	G. max	KAS551-4	PI 407924	DD-1055	South Korea	Landrace		
	G. max	KAS576-17	IT218314	DD-1056	South Korea	Landrace		
	G. max	KAS606-11	PI 424509	DD-1057	South Korea	Landrace		
	G. max	KAS624-46	IT218319	DD-1058	South Korea	Landrace		
	G. max	ORD 8182	IT226793	DD-1168	South Korea	Landrace		
	G. max	Suwon No. 64	PI 407826	DD-1267	South Korea	Improved line		
	G. max	PI 458283		DD-1291	South Korea	Landrace		
	G. max	KAS573-12	PI 458221	DD-0579	South Korea	Landrace		
	G. max	KLS743-2	IT022635	DD-0043	South Korea	Landrace		
	G. max	YN44	IT023811	DD-0066	South Korea	Landrace		
	G. max	YN69-4	IT023823	DD-0070	South Korea	Landrace		
	G. max	YN137-2	IT023873	DD-0076	South Korea	Landrace		
	G. max	YN176-2	IT023910	DD-0083	South Korea	Landrace		
	G. max	YN176-3	IT023911	DD-0084	South Korea	Landrace		
	G. max	Suwon45-3	IT024486	DD-0130	South Korea	Improved line		
	G. max	YN83	IT024520	DD-0133	South Korea	Landrace		
	G. max	YN118	IT024538	DD-0138	South Korea	Landrace		
	G. max	IT104009		DD-0233	South Korea	Landrace		
	G. max	SLSN54-1	IT134506	DD-0396	South Korea	Landrace		
	G. max	PI 399118		EE-1086	South Korea	Landrace		
	G. max	KLS605-1	PI 398944	EE-0024	South Korea	Landrace		
	G. max	SLS '81-00857	IT158131	EE-0570	South Korea	Landrace		
	G. max	KAS640-27	IT115814	EE-0295	South Korea	Landrace		
	G. max	NA 56703	PI 508297	EE-1117	South Korea	Landrace		
	G. max	PI 458296		EE-1048	South Korea	Landrace		
	G. max	KLS805-1	IT022647	EE-0042	South Korea	Landrace		
	G. max	Suwon64	IT024262	EE-0102	South Korea	Improved line		
	G. max	YN39	IT023809	EE-0070	South Korea	Landrace		
	G. max	Kongnamul	IT110949	EE-0241	South Korea	Landrace		
	G. max	PI 407847		EE-0997	South Korea	Landrace		
	G. max	KLS812	IT022658	EE-0044	South Korea	Landrace		
	G. max	KLS606-2	IT022527	EE-0025	South Korea	Landrace		
	G. max	YN115-2	IT023858	EE-0073	South Korea	Landrace		

	G. max	Yutae	IT231351	EE-1219	South Korea	Landrace		
	G. max	PI 407818 B		EE-0995	South Korea	Landrace		
	G. max	ORD 8168	IT228512	EE-1096	South Korea	Landrace		
	G. max	KAS552-7	IT218313	EE-0888	South Korea	Landrace		
	G. max	KLS087042	IT153371	EE-0445	South Korea	Landrace		
	G. max	KLS87042	IT153668	EE-0453	South Korea	Landrace		
	G. max	YN196-2	IT024450	EE-0115	South Korea	Landrace		
	G. max	YN195-3	IT023937	EE-0079	South Korea	Landrace		
	G. max	KLS743-1	PI 399012	EE-0040	South Korea	Landrace		
	G. max	Jilgeum	IT231357	EE-1221	South Korea	Landrace		
395	G. max	KAS663-14	IT115881	DD-0343	South Korea	Landrace	Purple	Brown
396	G. max	KAS671-10	IT115886	DD-0344	South Korea	Landrace	Purple	Brown
397	G. max	KAS681-3	IT115904	DD-0345	South Korea	Landrace	Purple	Black
398	G. max	KAS684-5	IT115935	DD-0347	South Korea	Landrace	Purple	Green
399	G. max	IT119895		DD-0348	South Korea	Landrace	Purple	Green
400	G. max	Kongnamul	IT119896	DD-0349	South Korea	Landrace	White	Yellow
401	G. max	KLS84006	IT120724	DD-0351	South Korea	Landrace	Purple	Green
402	G. max	KLS84073	IT120788	DD-0352	South Korea	Landrace	Purple	Black
403	G. max	Kongnamul	IT121464	DD-0353	South Korea	Landrace	Purple	Yellow
404	G. max	Bul	IT121465	DD-0354	South Korea	Landrace	Purple	Black
405	G. max	IT121504		DD-0355	South Korea	Landrace	Purple	Black
406	G. max	Heuinkongnamul	IT121516	DD-0356	South Korea	Landrace	Purple	Yellow
	G. max	KAS241-9	IT115467	EE-0259	South Korea	Landrace		
	G. max	SLS B-1	IT158117	EE-0566	South Korea	Landrace		
	G. max	SLSN-B-1	IT134324	EE-0322	South Korea	Landrace		
407	G. max	SLSJ42-3	IT134214	DD-0357	South Korea	Landrace	Purple	Yellow
408	G. max	SLSJ101-1	IT134220	DD-0359	South Korea	Landrace	White	Yellow
409	G. max	SLSJ175-2	IT134245	DD-0361	South Korea	Landrace	Purple	Yellow
410	G. max	SLSJ264-3	IT134257	DD-0362	South Korea	Landrace	Purple	Yellow
411	G. max	SLSJ-B-23	IT134290	DD-0363	South Korea	Landrace	White	Yellow
412	G. max	Forrest	PI 548655	DD-0364	United States	Improved line	White	Green yellow
413	G. max	SLSN-B-5	IT134328	DD-0367	South Korea	Landrace	Purple	Yellow
	G. max	KAS604-8	PI 408232	DD-1110	South Korea	Landrace		
	G. max	SLS B-5	IT158119	DD-0655	South Korea	Landrace		
414	G. max	Baegbong	IT134332	DD-0368	South Korea	Landrace	Purple	Yellow
415	G. max	SLSB67-1	IT134378	DD-0370	South Korea	Landrace	White	Brown
416	G. max	SLSB211-2	IT134396	DD-0373	South Korea	Landrace	Purple	Black
417	G. max	SLSB244-2	IT134400	DD-0374	South Korea	Landrace	Purple	Green
	G. max	KAS310-13	IT141539	DD-0410	South Korea	Landrace		
418	G. max	SLSB46-1	IT134402	DD-0375	South Korea	Landrace	Purple	Green
	G. max	Seonbi	IT195155	DD-1018	South Korea	Landrace		
	G. max	YB241	IT025086	DD-0187	South Korea	Landrace		
419	G. max	SLSB322-3	IT134413	DD-0377	South Korea	Landrace	Purple	Black
420	G. max	SLSB406-2	IT134433	DD-0378	South Korea	Landrace	White	Black
421	G. max	SLSB519-2	IT134446	DD-0380	South Korea	Landrace	Purple	Green
422	G. max	SLSB397-1	IT134454	DD-0381	South Korea	Landrace	White	Brown
423	G. max	SLSB398-1	IT134458	DD-0382	South Korea	Landrace	Purple	Black
424	G. max	SLSB-B-4	IT134463	DD-0383	South Korea	Landrace	Purple	Brown
425	G. max	SLSB-B-11	IT134466	DD-0384	South Korea	Landrace	White	Brown
	G. max	KAS530-35	IT115660	EE-0278	South Korea	Landrace		
426	G. max	SLSB-B-15	IT134470	DD-0385	South Korea	Landrace	Purple	Black
427	G. max	SLSB-B-26	IT134476	DD-0388	South Korea	Landrace	Purple	Black
428	G. max	SLSB-B-27	IT134477	DD-0389	South Korea	Landrace	Purple	Yellow
429	G. max	SLSB-B-32	IT134479	DD-0390	South Korea	Landrace	Purple	Black
430	G. max	SLSB-B-41	IT134484	DD-0391	South Korea	Landrace	Purple	Black
431	G. max	SLSB-B-44	IT134485	DD-0392	South Korea	Landrace	Purple	Black
432	G. max	SLSB-B-61	IT134495	DD-0393	South Korea	Landrace	Purple	Green
433	G. max	SLSB-B-63	IT134496	DD-0394	South Korea	Landrace	Purple	Green
434	G. max	SLSN41-1	IT134504	DD-0395	South Korea	Landrace	White	Green yellow
435	G. max	SLSN261-3	IT134525	DD-0398	South Korea	Landrace	White	Black
436	G. max	SLSN263-1	IT134527	DD-0399	South Korea	Landrace	White	Black
437	G. max	IT136088		DD-0401	South Korea	Landrace	Purple	Yellow
438	G. max	KAS200-12	IT141466	DD-0402	South Korea	Landrace	White	Yellow
439	G. max	KAS210-22	PI 458043	DD-0403	South Korea	Landrace	White	Black
440	G. max	KAS220-17	IT141492	DD-0404	South Korea	Landrace	Purple	Yellow
441	G. max	KAS241-4	PI 458093	DD-0407	South Korea	Landrace	Purple	Black
442	G. max	KAS243-4	PI 458100	DD-0408	South Korea	Landrace	Purple	Black

	G. max	SLSN-Bonghwoachunyang-5	IT022099	DD-0005	South Korea	Landrace			
	G. max	VIR 2962	IT230190	EE-1008	North Korea	Not determined			
	G. max	Geomjeongjilgeum	IT195512	DD-1024	South Korea	Landrace			
	G. max	PI 458109		DD-1190	South Korea	Landrace			
443	G. max	KAS301-11	PI 458117	DD-0409	South Korea	Landrace	White	Yellow	
444	G. max	KAS352-18	IT141586	DD-0411	South Korea	Landrace	Purple	Black	
445	G. max	KAS371-13	IT141651	DD-0413	South Korea	Landrace	Purple	Yellow	
446	G. max	KAS504-14	IT141668	DD-0416	South Korea	Landrace	Purple	Yellow	
447	G. max	KAS523-7	PI 458164	DD-0419	South Korea	Landrace	Purple	Black	
	G. max	KAS503-6	IT154215	EE-0490	South Korea	Landrace			
448	G. max	KAS523-17	IT141700	DD-0421	South Korea	Landrace	Purple	Yellow	
449	G. max	KAS523-24	IT141705	DD-0422	South Korea	Landrace	Purple	Yellow	
450	G. max	KAS540-30	PI 458187	DD-0424	South Korea	Landrace	Purple	Black	
451	G. max	KAS544-22	PI 424440	DD-0425	South Korea	Landrace	Purple	Black	
452	G. max	KAS574-8	PI 458228	DD-0428	South Korea	Landrace	Purple	Green	
453	G. max	KAS574-18	IT141792	DD-0429	South Korea	Landrace	Purple	Black	
	G. max	KAS632-33	IT154489	EE-0506	South Korea	Landrace			
454	G. max	KAS577-16	PI 458253	DD-0430	South Korea	Landrace	Purple	Yellow	
455	G. max	KAS580-16	IT141828	DD-0431	South Korea	Landrace	Purple	Black	
456	G. max	KAS590-14	IT141852	DD-0434	South Korea	Landrace	Purple	Green	
457	G. max	KAS590-16	IT141853	DD-0435	South Korea	Landrace	White	Yellow	
458	G. max	KAS600-5-2	IT141855	DD-0436	South Korea	Landrace	Purple	Green	
459	G. max	KAS606-16	IT141880	DD-0437	South Korea	Landrace	Purple	Yellow	
460	G. max	KAS607-4	IT141897	DD-0440	South Korea	Landrace	Purple	Yellow	
461	G. max	KAS611-1	IT141910	DD-0441	South Korea	Landrace	Purple	Yellow	
462	G. max	KAS612-16	IT141915	DD-0443	South Korea	Landrace	White	Green	
463	G. max	KAS613-11	IT141920	DD-0444	South Korea	Landrace	Purple	Yellow	
464	G. max	KAS616-5	IT141931	DD-0445	South Korea	Landrace	Purple	Brown	
465	G. max	KAS622-4	PI 408280	DD-0446	South Korea	Landrace	Purple	Yellow	
	G. max	KAS624-6	PI 408287	DD-0585	South Korea	Landrace			
	G. max	KAS624-17	IT154436	EE-0503	South Korea	Landrace			
	G. max	KAS624-7	PI 408288	EE-0502	South Korea	Landrace			
466	G. max	KAS624-15	IT141955	DD-0448	South Korea	Landrace	Purple	Black	
467	G. max	KAS624-20	PI 424531	DD-0449	South Korea	Landrace	Purple	Black	
468	G. max	KAS679-12	IT141962	DD-0450	South Korea	Landrace	Purple	Yellow	
469	G. max	KAS629-3	PI 424136	DD-0453	South Korea	Landrace	Purple	Green yellow	
470	G. max	KAS631-29	IT142058	DD-0455	South Korea	Landrace	Purple	Green	
471	G. max	Suwon130	IT142788	DD-0456	South Korea	Improved line	White	Yellow	
472	G. max	Baektae-eul	IT142820	DD-0459	South Korea	Landrace	Purple	Green	
473	G. max	Ol	IT142823	DD-0460	South Korea	Landrace	White	Yellow	
474	G. max	KLS86083	IT142839	DD-0461	South Korea	Landrace	White	Black	
475	G. max	KLS86085	IT142845	DD-0462	South Korea	Landrace	Purple	Black	
476	G. max	KLS86086	IT142848	DD-0463	South Korea	Landrace	Purple	Yellow	
477	G. max	KLS86094	IT142878	DD-0466	South Korea	Landrace	White	Green	
478	G. max	KLS86095	IT142882	DD-0467	South Korea	Landrace	White	Yellow	
479	G. max	KLS86105	IT142913	DD-0468	South Korea	Landrace	Purple	Yellow	
480	G. max	KLS86117	IT142938	DD-0470	South Korea	Landrace	Purple	Black	
481	G. max	KLS85001	IT142944	DD-0471	South Korea	Landrace	White	Yellow	
482	G. max	KLS85004	IT142947	DD-0472	South Korea	Landrace	White	Yellow	
483	G. max	KLS85008	IT142950	DD-0474	South Korea	Landrace	White	Yellow	
484	G. max	KLS85013	IT142955	DD-0475	South Korea	Landrace	White	Yellow	
485	G. max	KLS85014	IT142956	DD-0476	South Korea	Landrace	White	Yellow	
486	G. max	KLS85018	IT142960	DD-0477	South Korea	Landrace	White	Yellow	
487	G. max	KLS85024	IT142966	DD-0479	South Korea	Landrace	White	Yellow	
488	G. max	KLS85025	IT142967	DD-0480	South Korea	Landrace	White	Yellow	
489	G. max	KLS85034	IT142976	DD-0481	South Korea	Landrace	White	Yellow	
490	G. max	KLS85041	IT142983	DD-0482	South Korea	Landrace	White	Yellow	
491	G. max	KLS85052	IT142994	DD-0484	South Korea	Landrace	White	Yellow	
492	G. max	KLS85062	IT143004	DD-0486	South Korea	Landrace	White	Yellow	
493	G. max	KLS85065	IT143007	DD-0487	South Korea	Landrace	White	Yellow	
494	G. max	KLS85068	IT143010	DD-0488	South Korea	Landrace	White	Black	
495	G. max	KLS85084	IT143025	DD-0489	South Korea	Landrace	White	Yellow	
496	G. max	KLS85087	IT143028	DD-0490	South Korea	Landrace	White	Yellow	Representative
497	G. max	KLS85093	IT143034	DD-0492	South Korea	Landrace	White	Yellow	
498	G. max	KLS85110	IT143051	DD-0493	South Korea	Landrace	Purple	Black	

499	G. max	KLS85112	IT143053	DD-0494	South Korea	Landrace	White	Yellow
500	G. max	KLS85117	IT143058	DD-0495	South Korea	Landrace	White	Yellow
501	G. max	KLS85193	IT143112	DD-0497	South Korea	Landrace	Purple	Black
	G. max	KAS632-20	PI 424545	DD-0592	South Korea	Landrace		
	G. max	Geomjeong-2	IT177374	EE-0758	South Korea	Landrace		
	G. max	KAS651-15	PI 424580	EE-0297	South Korea	Landrace		
502	G. max	KLS85196	IT143115	DD-0498	South Korea	Landrace	Purple	Yellow
503	G. max	KLS85206	IT143125	DD-0500	South Korea	Landrace	Purple	Yellow
504	G. max	KLS85208	IT143127	DD-0501	South Korea	Landrace	Purple	Yellow
505	G. max	KLS85220	IT143138	DD-0502	South Korea	Landrace	Purple	Black
506	G. max	KLS85226	IT143144	DD-0504	South Korea	Landrace	White	Brown
507	G. max	KLS86119	IT143154	DD-0505	South Korea	Landrace	Purple	Black
508	G. max	KLS85241	IT143158	DD-0506	South Korea	Landrace	White	Yellow
509	G. max	KLS86002	IT143202	DD-0509	South Korea	Landrace	Purple	Brown
510	G. max	KLS86013	IT143213	DD-0511	South Korea	Landrace	Purple	Green
511	G. max	KLS86019	IT143219	DD-0512	South Korea	Landrace	Purple	Black
512	G. max	KLS86043	IT143243	DD-0514	South Korea	Landrace	Purple	Yellow
513	G. max	KLS86055	IT143255	DD-0515	South Korea	Landrace	Purple	Green
514	G. max	KLS86056	IT143256	DD-0516	South Korea	Landrace	Purple	Black
515	G. max	KLS86076	IT143276	DD-0518	South Korea	Landrace	Purple	Black
516	G. max	KLS86150	IT143313	DD-0520	South Korea	Landrace	White	Black
517	G. max	KLS86158	IT143321	DD-0521	South Korea	Landrace	Purple	Brown
518	G. max	KLS86161	IT143323	DD-0522	South Korea	Landrace	Purple	Yellow
519	G. max	KLS86164	IT143326	DD-0523	South Korea	Landrace	White	Yellow
520	G. max	KLS86179	IT143341	DD-0524	South Korea	Landrace	Purple	Yellow
521	G. max	KLS86188	IT143350	DD-0525	South Korea	Landrace	Purple	Yellow
	G. max	IT162896		DD-0787	South Korea	Landrace		
	G. max	Essex	PI 548667	DD-1429	United States	Improved line		
	G. max	KLS85047	IT142989	DD-0483	South Korea	Landrace		
	G. max	KLS85053	IT142995	DD-0485	South Korea	Landrace		
	G. max	Danyeop	IT021852	EE-0004	South Korea	Improved line		
	G. max	KLS85256	IT143173	EE-0430	South Korea	Landrace		
	G. max	KAS630-44	IT219649	EE-0903	South Korea	Landrace		
522	G. max	KLS86190	IT143352	DD-0526	South Korea	Landrace	Purple	Green
523	G. max	Suwon140	IT153343	DD-0527	South Korea	Improved line	Purple	Yellow
524	G. max	KLS087011	IT153351	DD-0528	South Korea	Landrace	Purple	Black
525	G. max	KLS087013	IT153352	DD-0529	South Korea	Landrace	Purple	Yellow
526	G. max	KLS087032	IT153366	DD-0530	South Korea	Landrace	Purple	Yellow
527	G. max	KLS087046	IT153373	DD-0532	South Korea	Landrace	Purple	Black
528	G. max	KLS087062	IT153382	DD-0534	South Korea	Landrace	Purple	Green
529	G. max	KLS087102	IT153397	DD-0536	South Korea	Landrace	Purple	Green
530	G. max	KLS087232	IT153429	DD-0539	South Korea	Landrace	Purple	Yellow
531	G. max	KLS87024	IT153654	DD-0540	South Korea	Landrace	Purple	Black
532	G. max	KLS87035	IT153662	DD-0541	South Korea	Landrace	White	Yellow
533	G. max	KLS87096	IT153718	DD-0544	South Korea	Landrace	White	Yellow
534	G. max	KLS87108	IT153725	DD-0546	South Korea	Landrace	White	Yellow
535	G. max	KLS87113	IT153730	DD-0547	South Korea	Landrace	Purple	Black
	G. max	KLS087119	IT153404	EE-0447	South Korea	Landrace		
536	G. max	KLS87129	IT153745	DD-0549	South Korea	Landrace	Purple	Yellow
537	G. max	KLS87149	IT153762	DD-0550	South Korea	Landrace	Purple	Green yellow
538	G. max	KLS87154	IT153765	DD-0551	South Korea	Landrace	White	Yellow
539	G. max	KLS87170	IT153778	DD-0553	South Korea	Landrace	Purple	Green
540	G. max	KLS87183	IT153788	DD-0555	South Korea	Landrace	Purple	Black
541	G. max	KLS87191	IT153796	DD-0556	South Korea	Landrace	Purple	Black
542	G. max	KLS87232	IT153830	DD-0557	South Korea	Landrace	White	Black
	G. max	PI 458060 B		EE-1146	South Korea	Landrace		
543	G. max	KLS87263	IT153857	DD-0561	South Korea	Landrace	Purple	Yellow
544	G. max	KLS87314	IT153892	DD-0563	South Korea	Landrace	Purple	Black
545	G. max	Nongrim44	IT153936	DD-0564	Japan	Improved line	Purple	Yellow
546	G. max	Nongrim67	IT153938	DD-0565	Japan	Improved line	White	Yellow
547	G. max	KAS380-15	IT154172	DD-0566	South Korea	Landrace	Purple	Yellow
548	G. max	KAS390-2	IT154177	DD-0567	South Korea	Landrace	Purple	Black
549	G. max	KAS390-3	IT154178	DD-0568	South Korea	Landrace	Purple	Yellow
550	G. max	KAS390-8	IT154182	DD-0569	South Korea	Landrace	Purple	Yellow
551	G. max	KAS391-3	PI 423849	DD-0570	South Korea	Landrace	Purple	Dark brown
	G. max	KLS86169	IT143331	EE-0438	South Korea	Landrace		
552	G. max	KAS503-15	PI 424388	DD-0571	South Korea	Landrace	Purple	Yellow

553	G. max	KAS511-3	IT154246	DD-0573	South Korea	Landrace	Purple	Yellow	
554	G. max	KAS521-8	PI 407886	DD-0575	South Korea	Landrace	Purple	Yellow	
555	G. max	KAS521-16-2	PI 424393	DD-0576	South Korea	Landrace	Purple	Green	
556	G. max	KAS521-22	PI 423853	DD-0577	South Korea	Landrace	Purple	Dark brown	
557	G. max	KAS524-4	IT154281	DD-0578	South Korea	Landrace	Purple	Yellow	
558	G. max	KAS574-11	PI 458231	DD-0580	South Korea	Landrace	Purple	Green	
559	G. max	KAS581-15	PI 424467	DD-0581	South Korea	Landrace	Purple	Black	
560	G. max	KAS581-16	PI 424468	DD-0582	South Korea	Landrace	Purple	Brown	
561	G. max	KAS622-8	PI 424525	DD-0583	South Korea	Landrace	Purple	Green	
562	G. max	KAS625-5	IT154442	DD-0586	South Korea	Landrace	Purple	Green	
563	G. max	KAS630-9	PI 408064	DD-0588	South Korea	Landrace	Purple	Yellow	
564	G. max	KAS631-37	IT154465	DD-0590	South Korea	Landrace	White	Yellow	
	G. max	KAS523-34	IT115630	DD-0311	South Korea	Landrace			
565	G. max	KAS632-18	PI 509108	DD-0591	South Korea	Landrace	Purple	Black	
566	G. max	KAS632-29	IT154485	DD-0593	South Korea	Landrace	Purple	Brown	
	G. max	SLSB161-1	IT134383	DD-0371	South Korea	Landrace			
567	G. max	KAS632-37	IT154492	DD-0594	South Korea	Landrace	Purple	Yellow	
568	G. max	KAS633-10	IT154503	DD-0597	South Korea	Landrace	Purple	Green	
	G. max	PI 458306 B		EE-1116	South Korea	Landrace			
569	G. max	KAS636-21	IT154540	DD-0599	South Korea	Landrace	Purple	Green	
570	G. max	KAS637-3	IT154545	DD-0600	South Korea	Landrace	Purple	Black	
571	G. max	KAS638-1	IT154561	DD-0601	South Korea	Landrace	Purple	Brown	
572	G. max	KAS638-10	IT154569	DD-0603	South Korea	Landrace	Purple	Green yellow	
573	G. max	KAS640-1	PI 408132	DD-0604	South Korea	Landrace	Purple	Black	
574	G. max	KAS640-49	IT154624	DD-0605	South Korea	Landrace	White	Green	
575	G. max	KAS641-1	PI 424142	DD-0606	South Korea	Landrace	White	Green yellow	
576	G. max	KAS641-7	IT154633	DD-0607	South Korea	Landrace	White	Brown	
577	G. max	KAS641-8	IT154634	DD-0608	South Korea	Landrace	Purple	Brown	
578	G. max	KAS644-5	PI 424166	DD-0610	South Korea	Landrace	Purple	Green	
579	G. max	KAS644-8	PI 442020	DD-0611	South Korea	Landrace	White	Yellow	
580	G. max	KAS648-1	IT154709	DD-0615	South Korea	Landrace	Purple	Green	
	G. max	KAS651-3	IT154714	DD-0617	South Korea	Landrace			
581	G. max	KAS648-7	IT154711	DD-0616	South Korea	Landrace	Purple	Yellow	
582	G. max	KAS651-8	PI 423862	DD-0618	South Korea	Landrace	Purple	Brown	
583	G. max	KAS651-23	PI 424588	DD-0619	South Korea	Landrace	Purple	Green	
584	G. max	KAS651-37	IT154735	DD-0620	South Korea	Landrace	Purple	Green	
	G. max	KAS683-7	IT115927	DD-0346	South Korea	Landrace			
585	G. max	KAS651-39	IT154737	DD-0621	South Korea	Landrace	Purple	Green yellow	
	G. max	IT138079		EE-0332	South Korea	Landrace			
586	G. max	KAS660-12	PI 408170	DD-0624	South Korea	Landrace	White	Yellow	
587	G. max	Dongsan108	IT154776	DD-0625	Japan	Improved line	Purple	Green	
588	G. max	Dongsan133	IT154786	DD-0626	Japan	Improved line	Purple	Yellow	
	G. max	TOUSAN 134	IT142925	EE-0390	Japan	Improved line			
589	G. max	Mokpo7	IT154806	DD-0627	South Korea	Improved line	Purple	Yellow	
590	G. max	IT155162		DD-0630	South Korea	Landrace	Purple	Green	
	G. max	IT155161		EE-0538	South Korea	Landrace			
591	G. max	KLS87098	IT155939	DD-0631	South Korea	Landrace	White	Yellow	
592	G. max	KLS87227	IT155947	DD-0632	South Korea	Landrace	Purple	Brown	
593	G. max	KLS87005	IT155984	DD-0633	South Korea	Landrace	White	Yellow	
	G. max	KAS649-1	IT154712	EE-0525	South Korea	Landrace			
	G. max	YB111-3	IT024406	EE-0108	South Korea	Landrace			
	G. max	YJ237-3	IT024772	EE-0155	South Korea	Landrace			
	G. max	KLS87167	IT155990	DD-0634	South Korea	Landrace			
594	G. max	KLS87202	IT155995	DD-0635	South Korea	Landrace	Purple	Green	
595	G. max	KLS87323	IT156001	DD-0636	South Korea	Landrace	Purple	Black	
596	G. max	KLS87348	IT156003	DD-0637	South Korea	Landrace	White	Black	
597	G. max	KLS87352	IT156006	DD-0638	South Korea	Landrace	White	Green	
598	G. max	CN290	PI 518677	DD-0640	United States	Improved line	Purple	Yellow	
599	G. max	Kangwon sujib2-4	IT156134	DD-0641	South Korea	Landrace	White	Black	
600	G. max	Kangwon sujib2-29	IT156157	DD-0642	South Korea	Landrace	Purple	Black	
601	G. max	Kangwon sujib2-33	IT156161	DD-0643	South Korea	Landrace	Purple	Brown	
602	G. max	Kangwon sujib3-1	IT156162	DD-0644	South Korea	Landrace	Purple	Green	
603	G. max	Kangwon sujib3-25	IT156184	DD-0645	South Korea	Landrace	Purple	Green	
604	G. max	IT156223	Kangwon sujib5-26	DD-0647	South Korea	Landrace	White	Brown	Representative
605	G. max	Gyeonggi5	IT157888	DD-0649	South Korea	Improved line	White	Yellow	
606	G. max	Milyang12	IT157938	DD-0650	South Korea	Improved line	White	Yellow	

607	G. max	Milyang26	IT157944	DD-0651	South Korea	Improved line	White	Yellow	
608	G. max	Milyang27	IT157945	DD-0652	South Korea	Improved line	White	Yellow	
609	G. max	Protana	PI 548528	DD-0654	United States	Improved line	Purple	Yellow	
	G. max	IT113169		EE-0247	South Korea	Landrace			
	G. max	Kongnamul	IT104067	EE-0220	South Korea	Landrace			
	G. max	PI399074		EE-0739	South Korea	Landrace			
	G. max	Kongnamul	IT104023	DD-0234	South Korea	Landrace			
	G. max	KLS88056	IT160118	DD-0661	South Korea	Landrace			
	G. max	Ol	IT157960	DD-0653	South Korea	Landrace			
610	G. max	SLS B30-2	IT158125	DD-0656	South Korea	Landrace	Purple	Black	
	G. max	SLSB411-1	IT134436	DD-0379	South Korea	Landrace			
	G. max	SLSB30-2	IT134371	DD-0369	South Korea	Landrace			
611	G. max	SLS B229-1	IT158136	DD-0657	South Korea	Landrace	Purple	Brown	
	G. max	YB358-1	IT025191	DD-0197	South Korea	Landrace			
	G. max	SLSB166-1	IT134385	DD-0372	South Korea	Landrace			
	G. max	KAS390-36	IT141663	DD-0414	South Korea	Landrace			
	G. max	KAS645-6	IT154684	DD-0612	South Korea	Landrace			
612	G. max	SLS B359-1	IT158141	DD-0658	South Korea	Landrace	White	Brown	
613	G. max	Gapsan jaerae	IT022370	DD-0659	South Korea	Landrace	White	Yellow	
	G. max	Gapsanjaelae	IT230721, Ii	DD-1340	South Korea	Landrace			
614	G. max	KLS88048	IT160111	DD-0660	South Korea	Landrace	White	Yellow	
615	G. max	KLS88064	IT160127	DD-0662	South Korea	Landrace	Purple	Black	
616	G. max	KLS88066	IT160129	DD-0663	South Korea	Landrace	White	Yellow	
	G. max	Jang	IT242606	EE-1270	South Korea	Landrace			
	G. max	Kyoungbuk Uiseong-1997-51	IT224179	DD-1138	South Korea	Landrace			
617	G. max	KLS88068-2	IT160132	DD-0664	South Korea	Landrace	Purple	Yellow	
	G. max	PI 458175 D		DD-1201	South Korea	Landrace			
	G. max	PI 458290		DD-1210	South Korea	Landrace			
	G. max	PI 509088		EE-0968	South Korea	Landrace			
618	G. max	KLS88070	IT160134	DD-0665	South Korea	Landrace	Purple	Yellow	
619	G. max	Kongnamul	IT160569	DD-0666	South Korea	Landrace	Purple	Green	
620	G. max	PI 60269-8		DD-0667	South Korea	Not determined	White	Yellow	
621	G. max	PI 62203-8		DD-0668	China	Not determined	White	Yellow	
622	G. max	PI 82588		DD-0669	South Korea	Landrace	Purple	Green yellow	
623	G. max	PI 84669		DD-0670	South Korea	Landrace	White	Yellow	
624	G. max	PI 84734		DD-0671	South Korea	Landrace	White	Yellow	
625	G. max	PI 85089		DD-0672	South Korea	Landrace	-	Yellow	
626	G. max	PI 85252		DD-0673	South Korea	Landrace	White	Black	
627	G. max	PI 86084		DD-0674	Japan	Not determined	Purple	Yellow	
628	G. max	PI 86982		DD-0675	South Korea	Landrace	Purple	Yellow	
629	G. max	PI 88820		DD-0676	North Korea	Not determined	White	Yellow	
630	G. max	PI 90251		DD-0677	South Korea	Landrace	White	Yellow	
631	G. max	PI 91100		DD-0678	China	Not determined	White	Yellow	Representative
632	G. max	PI 91725		DD-0679	North Korea	Not determined	White	Yellow	
	G. max	Akazu	PI 91725	DD-0838	North Korea	Not determined			
633	G. max	PI 95780		DD-0680	North Korea	Landrace	Purple	Yellow	
634	G. max	PI 96089-5		DD-0681	North Korea	Not determined	White	Yellow	
635	G. max	PI 196168		DD-0682	South Korea	Landrace	White	Yellow	
	G. max	SLSJ65-1	IT134218	DD-0358	South Korea	Landrace			
636	G. max	PI 196175		DD-0683	South Korea	Landrace	Purple	Yellow	
637	G. max	PI 227159		DD-0684	South Korea	Landrace	Purple	Yellow	
638	G. max	PI 340043		DD-0685	South Korea	Landrace	White	Yellow	
639	G. max	PI 86490		DD-0686	Japan	Not determined	Purple	Yellow	Representative
640	G. max	PI 95860		DD-0687	South Korea	Landrace	White	Yellow	
641	G. max	PI 97150		DD-0688	North Korea	Not determined	White	Yellow	
642	G. max	PI 97161		DD-0689	North Korea	Not determined	Purple	Yellow	
643	G. max	PI 84642		DD-0690	South Korea	Landrace	Purple	Yellow	
644	G. max	PI 87565		DD-0691	North Korea	Not determined	Purple	Yellow	
645	G. max	Cheongwon jaerae	IT161016	DD-0692	South Korea	Landrace	White	Yellow	
646	G. max	PI 54619		DD-0693	China	Not determined	White	Yellow	
	G. max	PI 85508		EE-0642	South Korea	Landrace			
647	G. max	PI 60269-2		DD-0694	South Korea	Not determined	Purple	Yellow	
648	G. max	PI 64698		DD-0695	South Korea	Landrace	White	Black	
649	G. max	PI 68011		DD-0696	China	Not determined	White	Yellow	
650	G. max	PI 68484-4		DD-0697	China	Not determined	Purple	Yellow	Representative
651	G. max	PI 80470		DD-0699	Japan	Not determined	Purple	Green	

652	G. max	PI 82183		DD-0700	South Korea	Landrace	Purple	Yellow	
653	G. max	PI 82218		DD-0701	South Korea	Landrace	Purple	Yellow	
	G. max	PI 82218		CD-1367	South Korea	Landrace			
	G. max	PI 339981		EE-0979	South Korea	Landrace			
	G. max	PI 82315		EE-0611	South Korea	Landrace			
654	G. max	PI 82246		DD-0702	South Korea	Landrace	Purple	Yellow	
655	G. max	PI 82278		DD-0703	South Korea	Landrace	Purple	Black	
656	G. max	PI 82291		DD-0704	South Korea	Landrace	Purple	Yellow	
657	G. max	PI 82509		DD-0705	North Korea	Not determined	Purple	Yellow	Representative
658	G. max	PI 82544		DD-0706	North Korea	Not determined	Purple	Yellow	
659	G. max	PI 83868		DD-0707	North Korea	Not determined	White	Yellow	
660	G. max	PI 83881A		DD-0708	North Korea	Not determined	Purple	Yellow	
661	G. max	PI 83893		DD-0710	South Korea	Landrace	White	Yellow	
662	G. max	PI 83923		DD-0711	Japan	Not determined	White	Yellow	
663	G. max	PI 83944		DD-0712	South Korea	Landrace	Purple	Yellow	
664	G. max	PI 84581		DD-0714	South Korea	Landrace	Purple	Green	
	G. max	PI 84581		DD-0054	South Korea	Landrace			
665	G. max	PI 84611		DD-0715	South Korea	Landrace	Purple	Black	Representative
666	G. max	PI 84639		DD-0717	South Korea	Landrace	White	Yellow	
667	G. max	PI 84644		DD-0718	South Korea	Landrace	Purple	Yellow	
	G. max	PI 84656		DD-0720	South Korea	Landrace			
668	G. max	PI 84646-2		DD-0719	South Korea	Not determined	Purple	Yellow	
669	G. max	PI 84680		DD-0721	South Korea	Landrace	White	Yellow	
670	G. max	PI 84681		DD-0722	South Korea	Landrace	Purple	Yellow	
671	G. max	PI 84713		DD-0723	South Korea	Landrace	Purple	Green	
672	G. max	PI 84928		DD-0725	North Korea	Not determined	Purple	Yellow	
	G. max	Hinkon	IT238307	DD-1402	South Korea	Landrace			
673	G. max	PI 85355		DD-0726	South Korea	Landrace	Purple	Yellow	
674	G. max	PI 85356		DD-0727	South Korea	Landrace	White	Yellow	
675	G. max	PI 85590		DD-0728	South Korea	Landrace	Purple	Yellow	
676	G. max	PI 85625		DD-0730	South Korea	Landrace	Purple	Yellow	
677	G. max	PI 86904-1		DD-0734	South Korea	Not determined	White	Yellow	
678	G. max	PI 86908		DD-0735	South Korea	Landrace	Purple	Yellow	
679	G. max	PI 87011		DD-0736	South Korea	Landrace	Purple	Yellow	
680	G. max	PI 87013		DD-0737	South Korea	Landrace	Purple	Yellow	
681	G. max	PI 87574		DD-0739	North Korea	Not determined	Purple	Yellow	Representative
	G. max	PI 84662		EE-0626	South Korea	Landrace			
682	G. max	PI 87575		DD-0740	North Korea	Not determined	Purple	Yellow	
683	G. max	PI 87619		DD-0741	North Korea	Not determined	Purple	Yellow	
684	G. max	PI 88306-1		DD-0744	China	Not determined	Purple	Yellow	
685	G. max	PI 88810		DD-0745	North Korea	Not determined	White	Yellow	
686	G. max	PI 88815		DD-0746	North Korea	Not determined	White	Yellow	
687	G. max	PI 89128		DD-0747	North Korea	Not determined	Purple	Yellow	
688	G. max	PI 89143		DD-0748	North Korea	Not determined	Purple	Yellow	Representative
689	G. max	PI 89154-1		DD-0749	North Korea	Not determined	Purple	Yellow	
690	G. max	PI 90208		DD-0750	North Korea	Not determined	Purple	Yellow	
691	G. max	PI 90221		DD-0751	North Korea	Not determined	Purple	Yellow	Representative
692	G. max	PI 90249		DD-0752	North Korea	Not determined	Purple	Yellow	
	G. max	Neihen	IT238315	EE-1249	North Korea	Not determined			
693	G. max	PI 90256		DD-0753	North Korea	Not determined	White	Yellow	
694	G. max	PI 91082		DD-0754	South Korea	Landrace	White	Yellow	
695	G. max	PI 91083		DD-0755	South Korea	Landrace	White	Yellow	
696	G. max	PI 92663		DD-0756	China	Not determined	Purple	Yellow	
697	G. max	PI 92720		DD-0757	China	Not determined	White	Black	
698	G. max	PI 95740		DD-0758	South Korea	Landrace	White	Yellow	
699	G. max	PI 95853		DD-0760	South Korea	Landrace	White	Yellow	
700	G. max	PI 96118		DD-0761	North Korea	Not determined	Purple	Yellow	
701	G. max	PI 96194P		DD-0762	China	Not determined	Purple	Yellow	
702	G. max	PI 96280		DD-0763	North Korea	Not determined	Purple	Yellow	
703	G. max	PI 97235		DD-0765	North Korea	Not determined	White	Yellow	
	G. max	PI 85624		DD-0729	South Korea	Landrace			
	G. max	PI 88826		EE-0654	North Korea	Not determined			
	G. max	Bongchonbaekmi	IT162649	EE-0676	South Korea	Landrace			
704	G. max	Oial	IT162657	DD-0766	South Korea	Landrace	Purple	Yellow	
	G. max	Gilkeumkong	IT230723	EE-1174	South Korea	Landrace			
	G. max	Oialkong No.5	IT162659	EE-0679	South Korea	Landrace			
705	G. max	Oialkong	IT162657	DD-0767	South Korea	Landrace	Purple	Yellow	

706	G. max	Baiktae	IT162665	DD-0768	South Korea	Landrace	Purple	Yellow	
707	G. max	Bangkohng	IT162666	DD-0769	South Korea	Landrace	Purple	Yellow	
708	G. max	Gwangjugun	IT162674	DD-0770	South Korea	Landrace	White	Yellow	
709	G. max	Cheonangun	IT162681	DD-0773	South Korea	Landrace	Purple	Yellow	
710	G. max	Goseonggun	IT162685	DD-0776	South Korea	Landrace	White	Yellow	
711	G. max	PI159764		DD-0777	South Korea	Landrace	Purple	Yellow	
712	G. max	Mochimame	IT162700	DD-0778	Japan	Not determined	Purple	Yellow	
713	G. max	Kongnamul	IT162744	DD-0779	South Korea	Landrace	Purple	Yellow	
714	G. max	Kongnamul	IT162846	DD-0781	South Korea	Landrace	Purple	Yellow	
715	G. max	IT162866		DD-0782	South Korea	Landrace	Purple	Black	
716	G. max	IT162868		DD-0783	South Korea	Landrace	White	Green	
717	G. max	IT162869		DD-0784	South Korea	Landrace	Purple	Black	
	G. max	IT162871		DD-0785	South Korea	Landrace			
718	G. max	Kongnamul	IT162873	DD-0786	South Korea	Landrace	Purple	Yellow	
719	G. max	Jujeonjeori	IT162906	DD-0788	South Korea	Landrace	Purple	Black	
720	G. max	KLS88020	IT163567	DD-0789	South Korea	Landrace	White	Yellow	
721	G. max	KLS88028	IT163575	DD-0790	South Korea	Landrace	White	Yellow	
722	G. max	KLS88035	IT163579	DD-0791	South Korea	Landrace	Purple	Green	
723	G. max	KLS88038-2	IT163583	DD-0792	South Korea	Landrace	White	Yellow	
724	G. max	Gregg	PI 510675	DD-0793	United States	Improved line	Purple	Yellow	
725	G. max	Harper	PI 548558	DD-0794	United States	Improved line	Purple	Yellow	
726	G. max	Hutcheson	PI 518664	DD-0795	United States	Improved line	White	Yellow	
727	G. max	Sibley	PI 508084	DD-0796	United States	Improved line	White	Yellow	
728	G. max	Ware	PI 548627	DD-0797	United States	Improved line	Purple	Yellow	
729	G. max	PI 479709		DD-0800	China	Not determined	Purple	Yellow	Representative
730	G. max	SLS90-3	IT167641	DD-0801	South Korea	Landrace	White	Yellow	
731	G. max	SLS90-7	IT167645	DD-0802	South Korea	Landrace	White	Yellow	
732	G. max	SLS90-9	IT167647	DD-0803	South Korea	Landrace	Purple	Yellow	
	G. max	Yuwoltae	PI 339868 A	EE-1259	South Korea	Landrace			
733	G. max	SLS90-32	IT167670	DD-0804	South Korea	Landrace	Purple	Brown	
734	G. max	SLS90-35	IT167673	DD-0805	South Korea	Landrace	Purple	Yellow	
	G. max	IT180648		EE-0807	South Korea	Landrace			
735	G. max	SLS90-57	IT167695	DD-0806	South Korea	Landrace	Purple	Yellow	
736	G. max	SLS90-78	IT167716	DD-0808	South Korea	Landrace	Purple	Black	
737	G. max	SLS90-79	IT167717	DD-0809	South Korea	Landrace	Purple	Yellow	
738	G. max	SLS90-102	IT167740	DD-0811	South Korea	Landrace	Purple	Green	
739	G. max	SLS90-106	IT167744	DD-0812	South Korea	Landrace	Purple	Yellow	
740	G. max	SLS90-119	IT167757	DD-0814	South Korea	Landrace	Purple	Green	
741	G. max	SLS90-121	IT167759	DD-0815	South Korea	Landrace	White	Yellow	
742	G. max	SLS90-142	IT167780	DD-0816	South Korea	Landrace	Purple	Yellow	
743	G. max	SLS90-172	IT167810	DD-0818	South Korea	Landrace	White	Yellow	
744	G. max	SLS90-197	IT167835	DD-0821	South Korea	Landrace	Purple	Black	
	G. max	KLS87081	IT153703	EE-0458	South Korea	Landrace			
745	G. max	SLS90-229	IT167867	DD-0822	South Korea	Landrace	White	Yellow	
746	G. max	SLS90-233	IT167871	DD-0823	South Korea	Landrace	White	Yellow	
747	G. max	SLS90-296	IT167934	DD-0826	South Korea	Landrace	White	Yellow	
748	G. max	SLS90-303	IT167941	DD-0827	South Korea	Landrace	White	Yellow	
749	G. max	KLS704-2	IT168196	DD-0829	South Korea	Landrace	Purple	Black	
750	G. max	Avery	PI 518663	DD-0830	United States	Improved line	White	Yellow	
751	G. max	Centennial	PI 548975	DD-0831	United States	Improved line	Purple	Yellow	
752	G. max	Conrad	PI 525453	DD-0832	United States	Improved line	Purple	Yellow	
753	G. max	Pershing	PI 548604	DD-0833	United States	Improved line	White	Yellow	
754	G. max	Sparks	PI 548619	DD-0834	United States	Improved line	White	Yellow	
755	G. max	Vickery	PI 548617	DD-0835	United States	Improved line	Purple	Yellow	
756	G. max	PI458505	IT170980	DD-0836	China	Not determined	Purple	Yellow	
757	G. max	Tracy-M	PI 548984	DD-0837	United States	Improved line	White	Yellow	
758	G. max	PI339736		DD-0840	South Korea	Landrace	Purple	Brown	
759	G. max	PI398420		DD-0841	South Korea	Landrace	Purple	Yellow	
760	G. max	PI 407795A		DD-0842	South Korea	Landrace	Purple	Yellow	
761	G. max	PI407877B		DD-0843	South Korea	Landrace	Purple	Yellow	
762	G. max	PI407892A		DD-0844	South Korea	Landrace	White	Yellow	
763	G. max	Choseng No.1	PI 427138	DD-0845	South Korea	Landrace	White	Yellow	
764	G. max	Heuin	IT175795	DD-0846	South Korea	Landrace	Purple	Yellow	
765	G. max	Sokuri	IT175866	DD-0849	South Korea	Landrace	Purple	Black	
766	G. max	Pureun	IT175903	DD-0850	South Korea	Landrace	Purple	Green	
767	G. max	Heuin	IT175923	DD-0851	South Korea	Landrace	Purple	Yellow	
768	G. max	Geomjeong	IT175926	DD-0852	South Korea	Landrace	Purple	Black	

	G. max	PI 458146		DD-1283	South Korea	Landrace		
769	G. max	Babmit	IT175966	DD-0853	South Korea	Landrace	Purple	Yellow
770	G. max	Babmit	IT175969	DD-0854	South Korea	Landrace	Purple	Black
771	G. max	Geomjeong-2	IT177200	DD-0856	South Korea	Landrace	Purple	Black
772	G. max	Geomjeong-3	IT177212	DD-0857	South Korea	Landrace	Purple	Black
773	G. max	Geomjeong-4	IT177226	DD-0858	South Korea	Landrace	Purple	Black
	G. max	Geomjeong-4	IT177226	DD-0859	South Korea	Landrace		
774	G. max	Geomjeong-6	IT177234	DD-0860	South Korea	Landrace	Purple	Black
775	G. max	Geomjeong-4	IT177242	DD-0861	South Korea	Landrace	Purple	Black
776	G. max	Geomjeong-10	IT177248	DD-0862	South Korea	Landrace	Purple	Black
777	G. max	Geomjeong-3	IT177305	DD-0866	South Korea	Landrace	Purple	Black
	G. max	Jeonnam Wondo-2000-26	IT224435	DD-1144	South Korea	Landrace		
778	G. max	Geomjeong-1	IT177314	DD-0867	South Korea	Landrace	Purple	Black
	G. max	Geomjeong-3	IT177316	DD-0868	South Korea	Landrace		
779	G. max	IT177322		DD-0870	South Korea	Landrace	Purple	Black
780	G. max	Geomjeong-6	IT177324	DD-0871	South Korea	Landrace	Purple	Black
781	G. max	IT177327		DD-0873	South Korea	Landrace	Purple	Black
782	G. max	Geomjeong-4	IT177328	DD-0874	South Korea	Landrace	Purple	Black
783	G. max	Geomjeong-3	IT177365	DD-0875	South Korea	Landrace	Purple	Black
784	G. max	Geomjeong-1	IT177379	DD-0877	South Korea	Landrace	Purple	Black
785	G. max	Geomjeong-4	IT177382	DD-0878	South Korea	Landrace	Purple	Black
786	G. max	IT177388		DD-0879	South Korea	Landrace	Purple	Black
787	G. max	Geomjeong-1	IT177394	DD-0880	South Korea	Landrace	Purple	Black
788	G. max	Geomjeong-1	IT177399	DD-0881	South Korea	Landrace	Purple	Black
789	G. max	Geomjeong-5	IT177408	DD-0883	South Korea	Landrace	Purple	Black
790	G. max	Geomjeong-3	IT177418	DD-0886	South Korea	Landrace	Purple	Green
	G. max	Geomjeong-4	IT177419	EE-0766	South Korea	Landrace		
791	G. max	Geomjeong-3	IT177423	DD-0888	South Korea	Landrace	Purple	Black
792	G. max	Geomjeong-1	IT177435	DD-0889	South Korea	Landrace	Purple	Black
793	G. max	Geomjeong-3	IT177437	DD-0890	South Korea	Landrace	Purple	Black
794	G. max	IT177513		DD-0893	South Korea	Landrace	White	Black
795	G. max	IT177518		DD-0894	South Korea	Landrace	Purple	Black
796	G. max	Geomjeong-3	IT177536	DD-0895	South Korea	Landrace	Purple	Black
797	G. max	Geomjeong-3	IT177541	DD-0896	South Korea	Landrace	White	Black
798	G. max	Geomjeong-3	IT177581	DD-0897	South Korea	Landrace	Purple	Black
799	G. max	IT177627		DD-0898	South Korea	Landrace	Purple	Black
	G. max	CS00825	IT219753	EE-0906	South Korea	Landrace		
	G. max	Geomjeong-2	IT177685	DD-0901	South Korea	Landrace		
800	G. max	Geomjeong-2	IT177645	DD-0900	South Korea	Landrace	Purple	Black
801	G. max	Geomjeong-3	IT177738	DD-0903	South Korea	Landrace	Purple	Black
802	G. max	Geomjeong-5	IT177783	DD-0905	South Korea	Landrace	Purple	Black
803	G. max	IT177793		DD-0906	South Korea	Landrace	Purple	Black
804	G. max	Geomjeong-5	IT177865	DD-0909	South Korea	Landrace	Purple	Black
805	G. max	Geomjeong-2	IT177918	DD-0911	South Korea	Landrace	Purple	Black
806	G. max	Geomjeong-5	IT177945	DD-0913	South Korea	Landrace	Purple	Black
807	G. max	IT177955		DD-0914	South Korea	Landrace	Purple	Black
808	G. max	IT178024		DD-0915	South Korea	Landrace	Purple	Black
809	G. max	Geomjeong-5	IT178072	DD-0916	South Korea	Landrace	Purple	Black
810	G. max	Geomjeong-3	IT178128	DD-0918	South Korea	Landrace	Purple	Black
811	G. max	Geomjeong	IT178160	DD-0920	South Korea	Landrace	Purple	Green
812	G. max	Geomjeong-2	IT178182	DD-0922	South Korea	Landrace	Purple	Brown
813	G. max	Geomjeong-1	IT178200	DD-0923	South Korea	Landrace	Purple	Green
814	G. max	Gulgeun	IT178368	DD-0925	South Korea	Landrace	Purple	Black
	G. max	Ajuggali	IT021840	DD-0001	South Korea	Landrace		
815	G. max	Bang	IT178374	DD-0926	South Korea	Landrace	Purple	Black
816	G. max	IT178450		DD-0928	South Korea	Landrace	Purple	Green
817	G. max	Namul	IT178511	DD-0931	South Korea	Landrace	Purple	Green yellow
818	G. max	Meju	IT178680	DD-0934	South Korea	Landrace	White	Yellow
819	G. max	Geomeunbak	IT178683	DD-0935	South Korea	Landrace	Purple	Black
	G. max	Geomjeongbak	IT196842	EE-0862	South Korea	Landrace		
	G. max	Geomjeong-1	IT177644	DD-0899	South Korea	Landrace		
	G. max	Geomjeong-1	IT177741	DD-0904	South Korea	Landrace		
820	G. max	Jeonnam jinheungwon NO.3	IT178695	DD-0936	South Korea	Landrace	Purple	Black
821	G. max	Jeonnam jinheungwon No.11	IT178701	DD-0937	South Korea	Landrace	Purple	Black

822	G. max	Jeonnam jinheungwon NO.25	IT178711	DD-0939	South Korea	Landrace	Purple	Black
823	G. max	Cheongwon104	IT179882	DD-0940	Japan	Improved line	White	Green yellow
824	G. max	Kangwon sujib2-20	IT179960	DD-0941	South Korea	Landrace	Purple	Black
825	G. max	IT180313		DD-0942	South Korea	Landrace	Purple	Yellow
826	G. max	IT180335		DD-0943	South Korea	Landrace	Purple	Brown
827	G. max	IT180348		DD-0944	South Korea	Landrace	Purple	Black
828	G. max	IT180361		DD-0945	South Korea	Landrace	Purple	Green
829	G. max	Galsaek	IT180497	DD-0948	South Korea	Landrace	Purple	Brown
830	G. max	Ajuggali	IT180504	DD-0949	South Korea	Landrace	Purple	Black
831	G. max	Ajuggali	IT180576	DD-0950	South Korea	Landrace	Purple	Black
832	G. max	Mokpo11	IT181356	DD-0954	South Korea	Improved line	White	Yellow
833	G. max	KAS151-30	IT181371	DD-0955	South Korea	Landrace	White	Yellow
834	G. max	KAS173-1	PI 398293	DD-0956	South Korea	Landrace	Purple	Brown
835	G. max	KAS231-1	PI 423768	DD-0957	South Korea	Landrace	White	Yellow
	G. max	KLS77017	IT025220	DD-0200	South Korea	Landrace		
	G. max	Cheongdogun	IT162687	EE-0684	South Korea	Landrace		
	G. max	Chougpukpaik	IT162664	EE-0680	South Korea	Landrace		
	G. max	IT105420		EE-0232	South Korea	Landrace		
	G. max	KAS511-9	IT154250	EE-0492	South Korea	Landrace		
	G. max	KLS87109	IT153726	EE-0460	South Korea	Landrace		
	G. max	Heuin	IT103717	EE-0215	South Korea	Landrace		
	G. max	Chungbukbaek	IT221981	EE-0937	South Korea	Improved line		
	G. max	PI 458307 B		EE-1263	South Korea	Landrace		
	G. max	KAS613-10	IT141919	EE-0372	South Korea	Landrace		
	G. max	PI 458042		EE-1144	South Korea	Landrace		
	G. max	IT186175		EE-0839	South Korea	Landrace		
	G. max	KLS087109	IT153399	DD-0537	South Korea	Landrace		
	G. max	KLS087117	IT153403	DD-0538	South Korea	Landrace		
	G. max	Goyang	IT162677	DD-0771	South Korea	Landrace		
	G. max	Yeongwolgun	IT162680	DD-0772	South Korea	Landrace		
	G. max	Mujugun	IT162684	DD-0775	South Korea	Landrace		
836	G. max	KAS300-11	IT181498	DD-0959	South Korea	Landrace	Purple	Green
837	G. max	KAS304-9	PI 424302	DD-0960	South Korea	Landrace	Purple	Black
838	G. max	KAS304-12	PI 424305	DD-0961	South Korea	Landrace	White	Yellow
839	G. max	KAS320-1	PI 398681	DD-0963	South Korea	Landrace	Purple	Green
840	G. max	KAS339-1	PI 423809	DD-0964	South Korea	Landrace	Purple	Black
841	G. max	KAS590-6	IT181672	DD-0967	South Korea	Landrace	Purple	Yellow
842	G. max	Nondureong	IT181943	DD-0968	South Korea	Landrace	Purple	Brown
843	G. max	IT186014		DD-0973	South Korea	Landrace	White	Black
844	G. max	IT186019		DD-0975	South Korea	Landrace	Purple	Black
845	G. max	IT186030		DD-0977	South Korea	Landrace	Purple	Black
846	G. max	IT186060		DD-0981	South Korea	Landrace	Purple	Black
847	G. max	IT186062		DD-0982	South Korea	Landrace	Purple	Green
848	G. max	IT186066		DD-0983	South Korea	Landrace	Purple	Black
849	G. max	IT186069		DD-0984	South Korea	Landrace	Purple	Brown
850	G. max	Kangwon69	IT186081	DD-0985	South Korea	Landrace	Purple	Black
851	G. max	Kangwon72	IT186084	DD-0986	South Korea	Landrace	Purple	Black
	G. max	KLS86120	IT143174	EE-0431	South Korea	Landrace		
	G. max	IT186093		EE-0835	South Korea	Landrace		
	G. max	Sokpareangi	IT242615	EE-1273	South Korea	Landrace		
	G. max	KLS087249	IT153437	EE-0451	South Korea	Landrace		
	G. max	Geomjeong-2	IT178163	EE-0786	South Korea	Landrace		
	G. max	CS00812	IT230135	EE-1135	South Korea	Landrace		
	G. max	Geomjeong	IT191169	EE-0848	South Korea	Landrace		
	G. max	Busan sujib	IT195624	EE-0857	South Korea	Landrace		
	G. max	Babmit	IT180857	EE-0812	South Korea	Landrace		
	G. max	Cheongtae	IT195519	EE-0855	South Korea	Landrace		
	G. max	CS 01991	IT231271	EE-1214	South Korea	Landrace		
	G. max	Geomjeong	IT209356	EE-0880	South Korea	Landrace		
	G. max	KLS85200	IT143119	DD-0499	South Korea	Landrace		
	G. max	KLS87249	IT153845	DD-0559	South Korea	Landrace		
	G. max	KLS87270	IT153862	DD-0562	South Korea	Landrace		
	G. max	Yak	IT175855	DD-0848	South Korea	Landrace		
	G. max	Geomjeong-2	IT177712	DD-0902	South Korea	Landrace		
	G. max	Geomjeong-2	IT177823	DD-0907	South Korea	Landrace		

	G. max	Geomjeong-3	IT177824	DD-0908	South Korea	Landrace			
	G. max	Geomjeong-2	IT177888	DD-0910	South Korea	Landrace			
	G. max	Geomjeong-5	IT177925	DD-0912	South Korea	Landrace			
	G. max	Geomjeong-3	IT178075	DD-0917	South Korea	Landrace			
	G. max	Geomjeong	IT178143	DD-0919	South Korea	Landrace			
	G. max	Seoribang	IT178376	DD-0927	South Korea	Landrace			
	G. max	IT180364		DD-0946	South Korea	Landrace			
	G. max	Ajuggali	IT180855	DD-0953	South Korea	Landrace			
	G. max	IT186058		DD-0980	South Korea	Landrace			
	G. max	IT186101		DD-0988	South Korea	Landrace			
	G. max	IT194545		DD-1015	South Korea	Landrace			
	G. max	IT194556		DD-1017	South Korea	Landrace			
	G. max	Geomjeong	IT208528	DD-1044	South Korea	Landrace			
	G. max	Seoritae	IT210088	DD-1048	South Korea	Landrace			
	G. max	Sokcheong	IT213320	DD-1053	South Korea	Landrace			
	G. max	Bammit	IT224597, Sokcheong	DD-1150	South Korea	Landrace			
	G. max	Geomeunparang	IT224918	DD-1159	South Korea	Landrace			
	G. max	Paring	IT224919	DD-1160	South Korea	Landrace			
	G. max	CS 01973	IT229075	DD-1301	South Korea	Landrace			
	G. max	Geomjeong-1	IT177440	DD-0891	South Korea	Landrace			
	G. max	Geomjeong-3	IT177472	DD-0892	South Korea	Landrace			
	G. max	Cheongtae	IT242612	DD-1420	South Korea	Landrace			
	G. max	Geomjeong	IT105500	DD-0250	South Korea	Landrace			
	G. max	KAS552-9	IT141778	DD-0427	South Korea	Landrace			
	G. max	KLS86115	IT142935	DD-0469	South Korea	Landrace			
	G. max	Chungnam Seocheon-1997-70	IT224177	EE-0944	South Korea	Landrace			
	G. max	IT160586		EE-0584	South Korea	Landrace			
	G. max	Jeongnak Hwasun-1997-40	IT224170	EE-0942	South Korea	Landrace			
	G. max	Sokcheong	IT224211	EE-0948	South Korea	Landrace			
	G. max	IT186043		EE-0831	South Korea	Landrace			
	G. max	Chungnamyesan-1999-64	IT224414	EE-0952	South Korea	Landrace			
	G. max	CS 02042	IT229089	EE-1128	South Korea	Landrace			
	G. max	Geomeun	IT180860	EE-0813	South Korea	Landrace			
852	G. max	Daedu	IT186114	DD-0991	South Korea	Landrace	White	Green	
853	G. max	Geomjeong	IT186146	DD-0993	South Korea	Landrace	Purple	Black	
854	G. max	IT186166		DD-0995	South Korea	Landrace	White	Yellow	
855	G. max	Babmit	IT186210	DD-0997	South Korea	Landrace	Purple	Green	
856	G. max	Jindo sujib	IT189239	DD-0999	South Korea	Landrace	Purple	Black	
857	G. max	IT194540		DD-1014	South Korea	Landrace	White	Yellow	
	G. max	IT186145		EE-0838	South Korea	Landrace			
	G. max	Keunol		KS119_C12	South Korea	Landrace			
	G. max	Keunol		KI118	South Korea	Landrace			
	G. max	Hwaeumput		CD-1290	South Korea	Improved line			
	G. max	Kyoungbukhadaedu	IT157887	DD-0648	South Korea	Landrace			
858	G. max	Heuksaekdaedu	IT194552	DD-1016	South Korea	Landrace	Purple	Black	
859	G. max	Seoritea	IT195241	DD-1019	South Korea	Landrace	Purple	Black	
860	G. max	GeomeunBabmit	IT195327	DD-1020	South Korea	Landrace	Purple	Black	
	G. max	Geomjeong-1	IT177385	EE-0761	South Korea	Landrace			
861	G. max	Jilgeum	IT195370	DD-1022	South Korea	Landrace	Purple	Yellow	
862	G. max	IT 195619		DD-1026	South Korea	Landrace	White	Green	Representative
863	G. max	Daegu sujib	IT195620	DD-1027	South Korea	Landrace	Purple	Black	
864	G. max	Hamyang sujib	IT195630	DD-1029	South Korea	Landrace	Purple	Black	
865	G. max	Geomjeong	IT196826	DD-1031	South Korea	Landrace	Purple	Black	
866	G. max	Yak	IT196827, bamsaek	DD-1032	South Korea	Landrace	Purple	Brown	
867	G. max	GeomjeongBabmit	IT196846	DD-1033	South Korea	Landrace	Purple	Black	
868	G. max	Chang Dan Bac Mok	IT196930	DD-1035	North Korea	Landrace	White	Yellow	
869	G. max	WIR2962	IT199101	DD-1036	North Korea	Not determined	White	Black	
870	G. max	WIR2987	IT199107	DD-1037	North Korea	Not determined	White	Yellow	
871	G. max	Tsak Nach	IT199123	DD-1038	North Korea	Not determined	Purple	Yellow	
872	G. max	Yak	IT201849	DD-1039	South Korea	Landrace	Purple	Black	
873	G. max	Yak	IT201866	DD-1040	South Korea	Landrace	Purple	Black	
874	G. max	Yak	IT201868	DD-1041	South Korea	Landrace	White	Black	

875	G. max	Babmit	IT204157	DD-1043	South Korea	Landrace	Purple	Brown
876	G. max	IT208844		DD-1045	South Korea	Landrace	Purple	Black
877	G. max	Namul	IT209187	DD-1046	South Korea	Landrace	White	Green
	G. max	KAS 150-21	PI 423742	DD-1116	South Korea	Landrace		
878	G. max	Gyeongnam Sancheong-1998-69	IT209912	DD-1047	South Korea	Landrace	White	Green yellow
	G. max	KAS581-33	IT141847	DD-0432	South Korea	Landrace		
879	G. max	Heuin	IT212806	DD-1050	South Korea	Landrace	Purple	Yellow
880	G. max	Jeonnam wando-2000-16	IT212852	DD-1051	South Korea	Landrace	White	Green yellow
	G. max	Jeonnam Wando-2000-57	IT224436	EE-0954	South Korea	Landrace		
	G. max	Pureunbang	IT220689	EE-0933	South Korea	Landrace		
	G. max	Jeonnam Goheung-1999-26	IT219470	EE-0890	South Korea	Landrace		
881	G. max	Kangwon goseong-2001-42	IT212878	DD-1052	South Korea	Landrace	Purple	Yellow
882	G. max	KAS 100-8-2	PI 423727	DD-1061	South Korea	Landrace	Purple	Yellow
883	G. max	KAS 150-24	PI 424238	DD-1063	South Korea	Landrace	White	Yellow
884	G. max	KAS 205-22	PI 424258	DD-1064	South Korea	Landrace	Purple	Brown
	G. max	PI 458106		EE-1149	South Korea	Landrace		
	G. max	KAS361-28	IT219785	DD-1104	South Korea	Landrace		
	G. max	Bul	IT228633	DD-1296	South Korea	Landrace		
	G. max	PI 458082	IT226868	EE-1016	South Korea	Landrace		
	G. max	YN7-1	IT024129	EE-0094	South Korea	Landrace		
	G. max	Bul	IT108951, Kongnamul	EE-0238	South Korea	Landrace		
885	G. max	Kongnamul	IT219318	DD-1065	South Korea	Landrace	Purple	Green
886	G. max	Kyoungbuk Kyoungsan-1997-54	IT219319	DD-1066	South Korea	Landrace	Purple	Black
887	G. max	Namul	IT219430	DD-1068	South Korea	Landrace	Purple	Green
888	G. max	NEBSOY	PI 548566	DD-1070	United States	Improved line	White	Yellow
889	G. max	Jwinuni	IT219522, yak	DD-1071	South Korea	Landrace	White	Black
	G. max	CS00837	IT219762	DD-1095	South Korea	Landrace		
	G. max	KAS351-35	PI 424367	DD-1099	South Korea	Landrace		
	G. max	KAS362-7	PI 423828	DD-1105	South Korea	Landrace		
	G. max	PI 399067		DD-1249	South Korea	Landrace		
	G. max	ORD 8118	IT228490	DD-1260	South Korea	Not determined		
	G. max	ORD 8132	PI 407798	DD-1261	South Korea	Landrace		
	G. max	ORD 8163	PI 407816	DD-1266	South Korea	Landrace		
	G. max	ORD 8152	PI 407841	DD-1270	South Korea	Landrace		
	G. max	Nondureong	IT155159	DD-0629	South Korea	Landrace		
	G. max	Kongnamul	IT186219	DD-0998	South Korea	Landrace		
	G. max	KAS651-50	IT218320	DD-1059	South Korea	Landrace		
	G. max	Cheongsutae	IT231354	DD-1368	South Korea	Landrace		
	G. max	YN98-4	IT024531	DD-0135	South Korea	Landrace		
	G. max	IT105268		DD-0246	South Korea	Landrace		
	G. max	Geomjeong	IT105634	DD-0252	South Korea	Landrace		
	G. max	KAS232-17	IT115441	DD-0290	South Korea	Landrace		
	G. max	SLSN234-3	IT134315	DD-0366	South Korea	Landrace		
	G. max	SLSN273-1	IT134528	DD-0400	South Korea	Landrace		
	G. max	YN117	IT023860	EE-0074	South Korea	Landrace		
	G. max	YN109-4	IT024136	EE-0095	South Korea	Landrace		
	G. max	CS 01966	IT229074	EE-1124	South Korea	Landrace		
	G. max	PI 399059		EE-1075	South Korea	Landrace		
	G. max	YN225-4	IT023969	EE-0082	South Korea	Landrace		
	G. max	YN166-1	IT023900	EE-0078	South Korea	Landrace		
	G. max	CS00839	IT219763	EE-0908	South Korea	Landrace		
	G. max	ORD 8142	PI 407804	EE-1093	South Korea	Landrace		
	G. max	SLS90-275	IT167913	EE-0719	South Korea	Landrace		
	G. max	KLS85192	IT143111	DD-0496	South Korea	Landrace		
	G. max	KLS86067	IT143267	DD-0517	South Korea	Landrace		
	G. max	Kongnamul	IT162772	DD-0780	South Korea	Landrace		
	G. max	IT178451		DD-0929	South Korea	Landrace		
	G. max	IT186157		DD-0994	South Korea	Landrace		
	G. max	KAS 100-11-1	PI 398186	DD-1062	South Korea	Landrace		
890	G. max	PI 424304		DD-1072	South Korea	Landrace	Purple	Yellow

891	G. max	KAS 150-7	IT219605	DD-1074	South Korea	Landrace	Purple	Green
892	G. max	KAS 150-9	PI 398245	DD-1075	South Korea	Landrace	Purple	Black
893	G. max	KAS 150-23	PI 423744	DD-1076	South Korea	Landrace	Purple	Yellow
	G. max	KAS171-6	IT115365	DD-0285	South Korea	Landrace		
894	G. max	KAS 160-5	IT219609	DD-1077	South Korea	Landrace	Purple	Black
895	G. max	KAS 160-21	IT219610	DD-1078	South Korea	Landrace	Purple	Yellow
896	G. max	KAS 201-7-1	IT219613	DD-1079	South Korea	Landrace	Purple	Yellow
897	G. max	KAS 205-5	IT219616	DD-1080	South Korea	Landrace	Purple	Green
898	G. max	KAS541-2	PI 407977	DD-1081	South Korea	Landrace	Purple	Yellow
899	G. max	KAS552-2	PI 407930	DD-1083	South Korea	Landrace	Purple	Green yellow
900	G. max	KAS604-3	IT219639	DD-1085	South Korea	Landrace	Purple	Yellow
	G. max	Meju	IT111090	DD-0266	South Korea	Landrace		
901	G. max	KAS604-12	PI 408236	DD-1086	South Korea	Landrace	Purple	Green
902	G. max	KAS616-6	IT219648	DD-1087	South Korea	Landrace	Purple	Yellow
903	G. max	KAS645-12	IT219651	DD-1088	South Korea	Landrace	Purple	Green
904	G. max	L-36	IT219697	DD-1089	South Korea	Landrace	Purple	Yellow
905	G. max	Yak	IT219699	DD-1090	South Korea	Landrace	Purple	Black
906	G. max	CS00650	IT219700	DD-1091	South Korea	Landrace	Purple	Black
907	G. max	CS00701	IT219704	DD-1093	South Korea	Landrace	Purple	Black
	G. max	CS 00831	IT221943	DD-1128	South Korea	Landrace		
	G. max	Spry	PI 553051	DD-1130	United States	Improved line		
	G. max	Jeju Jejusi-2008-409	IT228822	DD-1299	South Korea	Landrace		
	G. max	Subaktae(Ⅱ)	IT231359	DD-1370	South Korea	Landrace		
	G. max	KAS640-38	IT115816	DD-0333	South Korea	Landrace		
	G. max	IT161032		EE-0601	South Korea	Landrace		
	G. max	Socheongseomoktae	IT231355	EE-1220	South Korea	Landrace		
	G. max	Jilgeum	IT175829	EE-0742	South Korea	Landrace		
	G. max	Subaktae	IT112837	EE-0244	South Korea	Landrace		
	G. max	CS00676	IT219703	EE-0904	South Korea	Landrace		
	G. max	Juinuni	IT231390	EE-1222	South Korea	Landrace		
	G. max	Jeonbuk sujib	IT189301	EE-0845	South Korea	Landrace		
	G. max	Jannamul	IT181942	EE-0829	South Korea	Landrace		
	G. max	SLS90-111	IT167749	DD-0813	South Korea	Landrace		
	G. max	Subaktae	IT191159	DD-1001	South Korea	Landrace		
	G. max	Juinuni	IT218966	DD-1060	South Korea	Landrace		
	G. max	CS00653	IT219701	DD-1092	South Korea	Landrace		
908	G. max	CS00821	IT219751	DD-1094	South Korea	Landrace	Purple	Black
	G. max	Gyungnam Namhae-2000-2	IT219481	DD-1069	South Korea	Landrace		
	G. max	CS00828	IT225111	EE-0973	South Korea	Landrace		
	G. max	Yak	IT219479	EE-0893	South Korea	Landrace		
909	G. max	KAS102-5-2	IT219765	DD-1097	South Korea	Landrace	Purple	Yellow
910	G. max	KAS220-6	PI 423759	DD-1098	South Korea	Landrace	Purple	Yellow
911	G. max	KAS355-5	PI 423820	DD-1100	South Korea	Landrace	Purple	Green
912	G. max	KAS360-13	IT219779	DD-1102	South Korea	Landrace	White	Black
913	G. max	KAS360-15	IT219780	DD-1103	South Korea	Landrace	White	Green
914	G. max	KAS370-1	PI 398538	DD-1106	South Korea	Landrace	Purple	Yellow
915	G. max	KAS503-23	IT219795	DD-1107	South Korea	Landrace	Purple	Yellow
916	G. max	KAS521-33	IT219800	DD-1108	South Korea	Landrace	Purple	Green
917	G. max	KAS544-1	IT219810	DD-1109	South Korea	Landrace	Purple	Green
918	G. max	KAS643-25	IT219815	DD-1111	South Korea	Landrace	Purple	Black
919	G. max	KAS660-24	IT219816	DD-1112	South Korea	Landrace	Purple	Green yellow
920	G. max	KAS 100-12	PI 398187	DD-1113	South Korea	Landrace	Purple	Yellow
921	G. max	KAS 102-4-2	PI 398215	DD-1114	South Korea	Landrace	Purple	Black
922	G. max	KAS 134-2	IT220548	DD-1115	South Korea	Landrace	Purple	Black
923	G. max	KAS 150-25	PI 424239	DD-1117	South Korea	Landrace	Purple	Black
924	G. max	KAS 170-4	IT220555	DD-1118	South Korea	Landrace	Purple	Green
925	G. max	KAS 172-4	PI 398281	DD-1119	South Korea	Landrace	Purple	Brown
926	G. max	KAS 200-18	IT220559	DD-1120	South Korea	Landrace	Purple	Black
927	G. max	KAS 201-4	PI 398408	DD-1121	South Korea	Landrace	Purple	Brown
928	G. max	KAS 202-2	PI 424254	DD-1122	South Korea	Landrace	Purple	Black
929	G. max	KAS 205-13	PI 398442	DD-1123	South Korea	Landrace	Purple	Brown
930	G. max	KAS 205-21	IT220571	DD-1124	South Korea	Landrace	Purple	Brown
931	G. max	IT220684		DD-1125	South Korea	Landrace	Purple	Black
932	G. max	Sangjugeomjeongeolluk	IT220686	DD-1126	South Korea	Landrace	Purple	Black
933	G. max	Milyang166	IT221867	DD-1127	South Korea	Improved line	Purple	Brown

934	G. max	Cheongsong sujib-1	IT221947	DD-1129	South Korea	Landrace	White	Black
935	G. max	KAS 521-17	PI 424394	DD-1132	South Korea	Landrace	Purple	Green
936	G. max	Meju	IT224160	DD-1133	South Korea	Landrace	Purple	Yellow
	G. max	Baekun		CD-1178	South Korea	Improved line		
937	G. max	Gyeongbuk gyeongju-1997-11	IT224163	DD-1134	South Korea	Landrace	White	Yellow
	G. max	Kongnamul	IT109051	DD-0259	South Korea	Landrace		
	G. max	KLS87273	IT153864	EE-0477	South Korea	Landrace		
	G. max	KLS87256	IT153850	EE-0475	South Korea	Landrace		
938	G. max	Jeonnam jangheung-1997-58	IT224171	DD-1135	South Korea	Landrace	Purple	Yellow
939	G. max	Jeonnam yeonggwang-1997-73	IT224173	DD-1136	South Korea	Landrace	Purple	Black
940	G. max	Heuin	IT224182	DD-1139	South Korea	Landrace	White	Yellow
941	G. max	Seonbi	IT224183	DD-1140	South Korea	Landrace	Purple	Green
942	G. max	Jeonnam heanam-1998-16	IT224190	DD-1141	South Korea	Landrace	Purple	Yellow
943	G. max	Keumbwayu-tae	IT022373	DD-1143	South Korea	Landrace	Purple	Yellow
944	G. max	Flint	PI 595843	DD-1145	United States	Improved line	Purple	Yellow
945	G. max	Kangwon goseong-2001-12	IT224513	DD-1147	South Korea	Landrace	Purple	Yellow
946	G. max	Hanagari/bam	IT224808	DD-1151	South Korea	Landrace	Purple	Yellow
947	G. max	Kanto No. 21	PI 238928	DD-1155	Japan	Landrace	Purple	Yellow
948	G. max	PI 398577		DD-1156	South Korea	Landrace	Purple	Black
	G. max	Geomjeong-2	IT177295	DD-0865	South Korea	Landrace		
	G. max	YB202-4	IT025045	DD-0183	South Korea	Landrace		
	G. max	Geomjeong-4	IT177457	EE-0769	South Korea	Landrace		
949	G. max	Heihokuta	PI 88820	DD-1161	North Korea	Landrace	Purple	Yellow
950	G. max	U-ki-chol	PI 157483	DD-1162	South Korea	Landrace	Purple	Yellow
951	G. max	Ul-san	IT226756	DD-1163	South Korea	Landrace	Purple	Yellow
952	G. max	Well-man)	IT226757	DD-1164	South Korea	Landrace	White	Green
953	G. max	PI 339987		DD-1166	South Korea	Landrace	Purple	Yellow
954	G. max	PI 398988		DD-1167	South Korea	Landrace	White	Yellow
	G. max	KLS717-2	IT022599	DD-0036	South Korea	Landrace		
955	G. max	PI 407805 A		DD-1169	South Korea	Landrace	Purple	Yellow
956	G. max	PI 407805 D		DD-1170	South Korea	Landrace	Purple	Yellow
957	G. max	PI 407812		DD-1171	South Korea	Landrace	Purple	Yellow
958	G. max	PI 407817		DD-1172	South Korea	Landrace	Purple	Yellow
959	G. max	PI 407818 A		DD-1173	South Korea	Landrace	Purple	Yellow
	G. max	PI 407832 A		EE-0996	South Korea	Landrace		
960	G. max	KAS 239-3	IT226833	DD-1174	South Korea	Landrace	White	Yellow
961	G. max	Owonchoseng ji doo	PI 427139	DD-1178	South Korea	Landrace	Purple	Yellow
962	G. max	PI 458020		DD-1179	South Korea	Landrace	White	Green yellow
	G. max	ORD 8172	PI 407770	EE-0992	South Korea	Landrace		
963	G. max	PI 458026		DD-1180	South Korea	Landrace	Purple	Black
964	G. max	PI 458037		DD-1181	South Korea	Landrace	Purple	Yellow
965	G. max	PI 458041		DD-1182	South Korea	Landrace	White	Black
966	G. max	PI 458050		DD-1183	South Korea	Landrace	Purple	Yellow
967	G. max	PI 458051 A		DD-1184	South Korea	Landrace	Purple	Yellow
968	G. max	PI 458080		DD-1186	South Korea	Landrace	Purple	Yellow
969	G. max	PI 458101		DD-1188	South Korea	Landrace	White	Yellow
970	G. max	PI 458105		DD-1189	South Korea	Landrace	Purple	Yellow
	G. max	YN81	IT024519	EE-0125	South Korea	Landrace		
971	G. max	PI 458121		DD-1192	South Korea	Landrace	Purple	Brown
972	G. max	PI 458129		DD-1194	South Korea	Landrace	Purple	Yellow
973	G. max	PI 458135		DD-1196	South Korea	Landrace	Purple	Yellow
	G. max	KAS355-10	PI 458135	EE-0336	South Korea	Landrace		
974	G. max	PI 458157		DD-1197	South Korea	Landrace	Purple	Green
	G. max	PI 458158		DD-1198	South Korea	Landrace		
975	G. max	PI 458172 A		DD-1199	South Korea	Landrace	Purple	Black
976	G. max	PI 458175 C		DD-1200	South Korea	Landrace	White	Yellow
977	G. max	PI 458224		DD-1203	South Korea	Landrace	White	Yellow
978	G. max	PI 458244 B		DD-1205	South Korea	Landrace	White	Yellow
979	G. max	PI 458252		DD-1207	South Korea	Landrace	White	Green yellow
980	G. max	PI 458269		DD-1209	South Korea	Landrace	Purple	Green
981	G. max	PI 458295		DD-1211	South Korea	Landrace	Purple	Brown

982	G. max	VIR 2978	IT228333	DD-1214	North Korea	Not determined	Purple	Yellow	
983	G. max	Back Tac	PI 567273 A	DD-1215	South Korea	Landrace	Purple	Yellow	
984	G. max	Keunolkong	IT228343	DD-1217	South Korea	Improved line	Purple	Yellow	
985	G. max	GL 2624 /96	PI 603156	DD-1218	North Korea	Not determined	White	Green	
986	G. max	GL 2626 /96	PI 603158	DD-1219	North Korea	Not determined	Purple	Brown	
987	G. max	GL 2628 /96	PI 603159	DD-1220	North Korea	Not determined	Purple	Yellow	
988	G. max	GL 2631 /96	PI 603162	DD-1221	North Korea	Not determined	White	Black	Representative
989	G. max	GL 2678A /96	PI 603166	DD-1222	North Korea	Not determined	White	Yellow	
990	G. max	GL 2678B /96	PI 603167	DD-1223	North Korea	Not determined	Purple	Green yellow	
991	G. max	PI 603174 A		DD-1224	North Korea	Not determined	White	Black	
992	G. max	Byol	PI 603909 A	DD-1225	North Korea	Landrace	Purple	Yellow	
993	G. max	Cin	PI 603910 A	DD-1226	North Korea	Landrace	Purple	Black	Representative
994	G. max	Jijori	PI 603911 A	DD-1227	North Korea	Landrace	White	Yellow	
995	G. max	Uid	PI 603915 A	DD-1228	North Korea	Landrace	Purple	Yellow	
996	G. max	Musan-1	PI 612610	DD-1230	South Korea	Landrace	White	Brown	
	G. max	Khambuk 1	IT199136	EE-0869	North Korea	Landrace			
997	G. max	Browngilgun	PI 612611	DD-1231	South Korea	Landrace	Purple	Brown	
998	G. max	Ryong song	PI 612612 A	DD-1232	South Korea	Landrace	White	Yellow	
999	G. max	Br-45	PI 84874	DD-1233	South Korea	Landrace	Purple	Green	
1000	G. max	White-soybean	PI 157488	DD-1234	South Korea	Landrace	White	Yellow	
1001	G. max	PI 158751		DD-1235	South Korea	Landrace	Purple	Yellow	
1002	G. max	PI 339979		DD-1236	South Korea	Landrace	Purple	Brown	
1003	G. max	PI 340003		DD-1238	South Korea	Landrace	Purple	Yellow	
1004	G. max	PI 340014		DD-1239	South Korea	Landrace	Purple	Green	
1005	G. max	PI 340021 B		DD-1241	South Korea	Landrace	White	Yellow	
1006	G. max	KAERI-GNT 170-6	IT228424	DD-1243	South Korea	Landrace	Purple	Green	
	G. max	KLS86012	IT143212	DD-0510	South Korea	Landrace			
	G. max	PI 458204		DD-1202	South Korea	Landrace			
1007	G. max	IR 4791-89	PI 398372	DD-1244	South Korea	Landrace	Purple	Yellow	
1008	G. max	KLS 607-1	PI 398945	DD-1245	South Korea	Landrace	Purple	Brown	
1009	G. max	PI 399049		DD-1247	South Korea	Landrace	Purple	Green yellow	
1010	G. max	PI 399057		DD-1248	South Korea	Landrace	White	Green yellow	
1011	G. max	PI 399081		DD-1250	South Korea	Landrace	Purple	Green	
1012	G. max	PI 399082		DD-1251	South Korea	Landrace	White	Green	
1013	G. max	PI 399100		DD-1252	South Korea	Landrace	Purple	Green yellow	
1014	G. max	PI 399110		DD-1254	South Korea	Landrace	White	Green	
1015	G. max	PI 399111		DD-1255	South Korea	Landrace	White	Green yellow	
1016	G. max	PI 399122		DD-1257	South Korea	Landrace	Purple	Yellow	
1017	G. max	PI 399124		DD-1258	South Korea	Landrace	White	Green yellow	
1018	G. max	ORD 8110	IT228485	DD-1259	South Korea	Landrace	White	Yellow	
1019	G. max	ORD 8146	IT228504	DD-1263	South Korea	Not determined	Purple	Green yellow	
1020	G. max	ORD 8117	PI 407830	DD-1268	South Korea	Landrace	Purple	Yellow	
1021	G. max	ORD 8135	PI 407837	DD-1269	South Korea	Landrace	White	Yellow	
1022	G. max	ORD 8153	PI 407842	DD-1271	South Korea	Landrace	Purple	Black	
1023	G. max	KAERI 620-2	PI 408274	DD-1272	South Korea	Landrace	Purple	Black	
1024	G. max	Kinshuu ao shouryuu	PI 417041	DD-1273	South Korea	Landrace	Purple	Green	
	G. max	PI 458032		EE-1102	South Korea	Landrace			
1025	G. max	VIR 2978	IT228333	DD-1274	North Korea	Landrace	White	Yellow	Representative
1026	G. max	PI 458044		DD-1275	South Korea	Landrace	Purple	Yellow	
1027	G. max	PI 458048		DD-1276	South Korea	Landrace	Purple	Yellow	
1028	G. max	PI 458070 D		DD-1277	South Korea	Landrace	White	Black	
1029	G. max	PI 458073		DD-1278	South Korea	Landrace	Purple	Green	
1030	G. max	PI 458083		DD-1279	South Korea	Landrace	Purple	Brown	
1031	G. max	PI 458107		DD-1281	South Korea	Landrace	Purple	Brown	
1032	G. max	PI 458128		DD-1282	South Korea	Landrace	Purple	Green	
1033	G. max	PI 458154		DD-1284	South Korea	Landrace	Purple	Yellow	
1034	G. max	PI 458168		DD-1285	South Korea	Landrace	Purple	Green	
1035	G. max	PI 458218		DD-1287	South Korea	Landrace	Purple	Black	
1036	G. max	PI 458265		DD-1288	South Korea	Landrace	Purple	Black	
1037	G. max	PI 458268		DD-1289	South Korea	Landrace	Purple	Brown	
	G. max	PI 398943		DD-1157	South Korea	Landrace			
	G. max	KAS504-15	IT141669	DD-0417	South Korea	Landrace			
	G. max	YB192	IT025029	EE-0170	South Korea	Landrace			
1038	G. max	PI 458278 B		DD-1290	South Korea	Landrace	White	Brown	
1039	G. max	PI 458284		DD-1292	South Korea	Landrace	Purple	Black	
	G. max	KAS581-22	PI 458284	EE-0361	South Korea	Landrace			

1040	G. max	L-B	IT228619	DD-1294	South Korea	Landrace	Purple	Brown	
1041	G. max	CS 01939	IT228790	DD-1298	South Korea	Landrace	White	Black	
1042	G. max	CS 01993	IT229078	DD-1302	South Korea	Landrace	Purple	Green	
1043	G. max	CS 02008	IT229083	DD-1303	South Korea	Landrace	Purple	Black	
1044	G. max	Sugae #43(A)	IT229363	DD-1304	South Korea	Improved line	White	Yellow	
1045	G. max	Suwongaetong #2	IT229364	DD-1305	South Korea	Improved line	Purple	Yellow	
1046	G. max	Baekjung #42	IT229365	DD-1306	South Korea	Landrace	Purple	Yellow	
1047	G. max	Saepureun	IT229405	DD-1307	South Korea	Landrace	Purple	Green	
1048	G. max	Pureundogseagi	IT229464	DD-1308	South Korea	Landrace	Purple	Green	
1049	G. max	KAS 102-2	IT229952	DD-1310	South Korea	Landrace	Purple	Green	
1050	G. max	KAS 132-3	IT229956	DD-1311	South Korea	Landrace	Purple	Yellow	
1051	G. max	KAS 134-5	IT229958	DD-1312	South Korea	Landrace	Purple	Brown	
1052	G. max	Chang-tan-da-mac	PI 157409	DD-1314	South Korea	Landrace	Purple	Yellow	
1053	G. max	PI 273483 E		DD-1315	South Korea	Landrace	White	Yellow	
1054	G. max	Yuwoltae)	IT226762	DD-1316	South Korea	Landrace	White	Yellow	
1055	G. max	CHIRPAN 90 (BULGARIA)	PI 398276	DD-1317	South Korea	Landrace	Purple	Black	
1056	G. max	PI 399106		DD-1319	South Korea	Landrace	Purple	Brown	
1057	G. max	PI 407778 A		DD-1320	South Korea	Landrace	Purple	Black	
1058	G. max	ORD 8123	IT230180	DD-1321	South Korea	Landrace	Purple	Yellow	
1059	G. max	VIR 2962	IT230190	DD-1323	North Korea	Not determined	Purple	Black	Representative
1060	G. max	KLS 223	IT230212	DD-1324	South Korea	Landrace	White	Yellow	
1061	G. max	Peing bukai	PI 417240	DD-1325	South Korea	Landrace	Purple	Yellow	
1062	G. max	PI 458264		DD-1326	South Korea	Landrace	Purple	Green	
1063	G. max	Kaishoku Daizu	PI 416977	DD-1327	South Korea	Landrace	Purple	Brown	
1064	G. max	Choutan	IT230661	DD-1328	South Korea	Landrace	White	Yellow	
	G. max	Baekbamkong	IT230710	DD-1338	South Korea	Landrace			
	G. max	Baekbum Kong	IT230797	EE-1203	South Korea	Landrace			
	G. max	Baekjamkong	IT230709	EE-1169	South Korea	Landrace			
1065	G. max	Nezumi Meta	PI 417193	DD-1329	South Korea	Landrace	Purple	Black	
1066	G. max	Orukon	IT230665	DD-1330	South Korea	Not determined	Purple	Yellow	
1067	G. max	Noucha Taitai	PI 417199	DD-1331	South Korea	Landrace	Purple	Dark brown	
1068	G. max	Akasome Daizu	IT230692	DD-1333	South Korea	Landrace	Purple	Brown	
	G. max	Akasome daizu (Korea)	PI 416771	EE-1099	South Korea	Landrace			
1069	G. max	Suigen Ao	IT230695	DD-1334	South Korea	Landrace	Purple	Green	
	G. max	Suigen ao (Korea)	PI 417350	EE-1100	South Korea	Landrace			
1070	G. max	Kinzu	IT230701	DD-1336	South Korea	Landrace	White	Yellow	
	G. max	Keumdu	IT230747	EE-1183	South Korea	Landrace			
1071	G. max	Bongui	IT230715	DD-1339	South Korea	Landrace	Purple	Yellow	
	G. max	Bongui	IT230715	CD-1348	South Korea	Improved line			
	G. max	Kwangkyo	IT142800	DD-0457	South Korea	Improved line			
	G. max	KAS531-2	PI 424408	EE-0497	South Korea	Landrace			
	G. max	PI 458147		EE-1262	South Korea	Landrace			
	G. max	Suweon 9	IT230761	EE-1189	South Korea	Improved line			
	G. max	KAS671-3	IT181730	EE-0825	South Korea	Landrace			
1072	G. max	Iksan	IT230736	DD-1342	South Korea	Landrace	Purple	Yellow	
1073	G. max	KLS 631-2	IT230754	DD-1343	South Korea	Landrace	Purple	Green	
	G. max	SLS90-196	IT167834	DD-0820	South Korea	Landrace			
1074	G. max	KLS 704-3	IT230755	DD-1344	South Korea	Landrace	Purple	Green yellow	
1075	G. max	Oeal Kong	IT230756	DD-1345	South Korea	Landrace	Purple	Yellow	
1076	G. max	Baekmo #9	IT230778	DD-1348	South Korea	Landrace	Purple	Yellow	
1077	G. max	Sugae #44	IT230781	DD-1349	South Korea	Improved line	Purple	Yellow	
1078	G. max	Sugae #41	IT230785	DD-1350	South Korea	Improved line	White	Yellow	
1079	G. max	Yung Kwang #1	IT230786	DD-1351	South Korea	Landrace	White	Yellow	
1080	G. max	Jaeraejong	IT230790	DD-1352	South Korea	Landrace	Purple	Yellow	
1081	G. max	Sugae #46(A 1)	IT230798	DD-1353	South Korea	Improved line	Purple	Yellow	
1082	G. max	Sugae #46(B2)	IT230800	DD-1354	South Korea	Improved line	Purple	Yellow	
1083	G. max	Sugae #42	IT230802	DD-1355	South Korea	Improved line	Purple	Yellow	
1084	G. max	Yungil	IT230803	DD-1356	South Korea	Landrace	Purple	Yellow	
1085	G. max	Chonggok	IT230808	DD-1358	South Korea	Landrace	Purple	Yellow	
1086	G. max	Pyngyang	IT230812	DD-1359	South Korea	Landrace	Purple	Yellow	
1087	G. max	KLS 222-2	IT230817	DD-1360	South Korea	Landrace	White	Yellow	
1088	G. max	KLS 512-1(A)	IT230819	DD-1361	South Korea	Landrace	Purple	Yellow	
1089	G. max	KLS 708-3(1)	IT230823	DD-1362	South Korea	Landrace	White	Yellow	
	G. max	KLS 710(B)	IT230825	EE-1209	South Korea	Landrace			
1090	G. max	KLS 821	IT230832	DD-1363	South Korea	Landrace	Purple	Yellow	

1091	G. max	KLS 150-2-1	IT230835	DD-1364	South Korea	Landrace	Purple	Brown	
1092	G. max	KAS 150-2-2	IT230840	DD-1365	South Korea	Landrace	Purple	Brown	
1093	G. max	KAS 150-2-6	IT230841	DD-1366	South Korea	Landrace	Purple	Green	
	G. max	KAS 150-2-8	IT230843	EE-1213	South Korea	Landrace			
1094	G. max	Bultae	IT231352	DD-1367	South Korea	Landrace	Purple	Brown	
1095	G. max	PI 87002		DD-1371	South Korea	Landrace	White	Yellow	
1096	G. max	PI 90241		DD-1372	North Korea	Not determined	Purple	Yellow	
	G. max	PI90241		DD-0350	North Korea	Not determined			
	G. max	PI90233		EE-0657	North Korea	Not determined			
	G. max	PI90223		EE-0055	North Korea	Not determined			
1097	G. max	Jilgeum	IT231543	DD-1373	South Korea	Landrace	White	Green	
1098	G. max	IT234975		DD-1374	South Korea	Landrace	Purple	Black	
1099	G. max	PI 458250		DD-1376	South Korea	Landrace	Purple	Green	
1100	G. max	Milyang206		DD-1377	South Korea	Improved line	White	Yellow	
	G. max	Deamang		CD-1202	South Korea	Improved line			
	G. max	Neulchan		CD-1210	South Korea	Improved line			
	G. max	Neulchan		EE-1278	South Korea	Improved line			
1101	G. max	Milyang194	IT236161	DD-1378	South Korea	Improved line	Purple	Black	
1102	G. max	Milyang195	IT236162	DD-1379	South Korea	Improved line	Purple	Black	
1103	G. max	Iksan66	IT236163	DD-1380	South Korea	Improved line	Purple	Yellow	
1104	G. max	KAS 150-3	IT236702	DD-1381	South Korea	Landrace	Purple	Black	
1105	G. max	PI 458058		DD-1382	South Korea	Landrace	Purple	Yellow	
1106	G. max	Yuumou Shinnamon	PI 507569	DD-1383	South Korea	Landrace	White	Brown	
1107	G. max	CS 02038	IT237763	DD-1385	South Korea	Landrace	Purple	Black	Representative
1108	G. max	KAS 633-19	IT238134	DD-1387	South Korea	Landrace	Purple	Brown	
	G. max	PI 408105A	KAS 633-19	CD-1313	South Korea	Landrace			
1109	G. max	No. 39 Green	PI 171430	DD-1388	China	Not determined	White	Green yellow	
1110	G. max	Hua huang dou	IT238194	DD-1389	China	Not determined	Purple	Brown	
1111	G. max	Bai hua chi	PI 567519	DD-1390	China	Not determined	White	Yellow	
1112	G. max	Niu mao huang	IT238204	DD-1391	China	Not determined	White	Yellow	
1113	G. max	Xiao tie jiao	PI 567601	DD-1392	China	Not determined	Purple	Yellow	
1114	G. max	Ji li huang dou	PI 567458	DD-1393	China	Not determined	Purple	Yellow	
	G. max	Xiao bai huang dou	IT238226	EE-1242	China	Not determined			
1115	G. max	PI 567591		DD-1394	China	Not determined	White	Yellow	Representative
1116	G. max	Si li da dou	PI 567587 A	DD-1395	China	Not determined	White	Yellow	
1117	G. max	PI 567660 A		DD-1396	China	Not determined	Purple	Yellow	Representative
1118	G. max	Tong shan huang da dou jia	PI 567773	DD-1397	China	Not determined	Purple	Yellow	
1119	G. max	Huang pi feng zi wo	PI 587718	DD-1398	China	Not determined	White	Yellow	
1120	G. max	Cai dou	PI 567297	DD-1399	China	Not determined	Purple	Brown	
1121	G. max	Pakute)	IT238303	DD-1401	South Korea	Landrace	Purple	Yellow	
	G. max	PI 87005		EE-0647	South Korea	Landrace			
1122	G. max	Moyashimame	PI 87059	DD-1403	South Korea	Landrace	White	Black	
	G. max	PI 87059		EE-0648	South Korea	Landrace			
1123	G. max	Mote	PI 88814	DD-1404	North Korea	Landrace	Purple	Yellow	
1124	G. max	Chankon	IT238319	DD-1405	North Korea	Landrace	Purple	Yellow	
1125	G. max	KAERI-GNT 330-1	IT238321	DD-1406	South Korea	Improved line	Purple	Yellow	
1126	G. max	Jilgeum	IT238356	DD-1407	South Korea	Landrace	Purple	Yellow	
1127	G. max	PI 340039		DD-1410	South Korea	Landrace	Purple	Green yellow	
1128	G. max	PI 340046		DD-1411	South Korea	Landrace	White	Yellow	
1129	G. max	PI 340052		DD-1412	South Korea	Landrace	White	Yellow	
1130	G. max	PI 398504		DD-1413	South Korea	Landrace	White	Yellow	
1131	G. max	PI 398885		DD-1414	South Korea	Landrace	White	Yellow	
1132	G. max	PI 458038		DD-1415	South Korea	Landrace	Purple	Yellow	
1133	G. max	Hwaseongputkong	PI 597487	DD-1416	South Korea	Improved line	White	Yellow	
1134	G. max	PI 398593		DD-1418	South Korea	Landrace	Purple	Yellow	
1135	G. max	geuru	IT242610	DD-1419	South Korea	Landrace	Purple	Yellow	
1136	G. max	Pureude	IT242614	DD-1421	South Korea	Landrace	Purple	Green	
1137	G. max	Ajuggalibam	IT242616	DD-1422	South Korea	Landrace	Purple	Black	
1138	G. max	Daechubam	IT242621	DD-1423	South Korea	Landrace	Purple	Brown	
	G. max	YB266	IT025101	DD-0190	South Korea	Landrace			
1139	G. max	Junghwang35		DD-1424	China	Improved line	White	Yellow	
1140	G. max	TS3-2		DD-1425	South Korea	Improved line	Purple	Yellow	
1141	G. max	HS5		DD-1426	South Korea	Improved line	Purple	Yellow	
1142	G. max	CNS	PI 548445	DD-1428	China	Landrace	Purple	Yellow	

1143	G. max	GWS95-Kangwongdo		DD-1430	South Korea	Landrace	Purple	Black	
1144	G. max	Lindou 9		DD-1431	China	Improved line	Purple	Yellow	
1145	G. max	Gaozuoxuan 1		DD-1432	China	Improved line	Purple	Yellow	
1146	G. max	Tiefeng 31		DD-1433	China	Improved line	Purple	Yellow	
1147	G. max	Yannong 12		DD-1434	China	Improved line	Purple	Yellow	
1148	G. max	Changnong 21		DD-1435	China	Improved line	White	Yellow	
1149	G. max	Jiyu 97		DD-1436	China	Improved line	Purple	Yellow	
1150	G. max	Jiyu 202		DD-1437	China	Improved line	White	Yellow	
1151	G. max	Jiyu 86		DD-1438	China	Improved line	Purple	Yellow	
1152	G. max	Heinong 52		DD-1439	China	Improved line	Purple	Yellow	
1153	G. max	PI407736		DD-0166	China	Not determined	Purple	Yellow	
1154	G. max	PI68564		DD-0698	China	Not determined	Purple	Yellow	
1155	G. max	PI464916		DD-0798	China	Not determined	White	Yellow	
1156	G. max	IT180422		DD-0947	China	Not determined	Purple	Green	Representative
1157	G. max	IT224873		DD-1152	China	Landrace	White	Yellow	Representative
1158	G. max	Nan zhao cao huang dou	PI 567642 A	DD-1153	China	Landrace	Purple	Yellow	
1159	G. max	Fu yang (30)	PI 567709	DD-1154	China	Landrace	White	Yellow	
1160	G. max	Soheung2		DD-0639	Japan	Improved line	Purple	Green yellow	
1161	G. max	PI87630		DD-0742	Japan	Not determined	Purple	Yellow	
1162	G. max	PI87631-1		DD-0743	Japan	Not determined	Purple	Green yellow	Representative
1163	G. max	Daeheug		Deahek	South Korea	Improved line	Purple	Black	
	G. max	Daeheug		CD-1268	South Korea	Improved line			
1164	G. max	Milyang5	IT021588	EE-0001	South Korea	Improved line	Purple	Yellow	
	G. max	Milyang5	IT134176	EE-0310	South Korea	Improved line			
1165	G. max	Horangi	IT021842	EE-0003	South Korea	Landrace	Purple	Brown	
1166	G. max	Keumkangdaelib	IT021889	EE-0006	South Korea	Improved line	Purple	Yellow	
1167	G. max	Hyong	IT022328	EE-0011	South Korea	Not determined	White	Yellow	
1168	G. max	KLS136	IT022438	EE-0014	South Korea	Landrace	Purple	Yellow	
1169	G. max	KLS137-1	PI 398888	EE-0015	South Korea	Landrace	Purple	Yellow	
1170	G. max	KLS202	PI 398890	EE-0016	South Korea	Landrace	Purple	Yellow	
1171	G. max	KLS216-2	IT022463	EE-0018	South Korea	Landrace	Purple	Green yellow	
	G. max	KLS201-1	PI 398889	DD-0017	South Korea	Landrace			
	G. max	YJ16-2	IT024398	EE-0106	South Korea	Landrace			
1172	G. max	KLS417-1	PI 398929	EE-0021	South Korea	Landrace	Purple	Black	
1173	G. max	KLS505-1	PI 398935	EE-0023	South Korea	Landrace	Purple	Green	
1174	G. max	KLS606-2	IT022527	EE-0026	South Korea	Landrace	Purple	Green	
1175	G. max	KLS611	PI 398949	EE-0027	South Korea	Landrace	Purple	Green	
1176	G. max	KLS616-2	IT022546	EE-0028	South Korea	Landrace	White	Yellow	
1177	G. max	KLS618-1	IT022549	EE-0030	South Korea	Landrace	Purple	Yellow	
1178	G. max	KLS621-1	PI 398958	EE-0031	South Korea	Landrace	Purple	Green	
1179	G. max	KLS624-2	IT022558	EE-0032	South Korea	Landrace	Purple	Yellow	
1180	G. max	KLS701-3	IT022576	EE-0034	South Korea	Landrace	Purple	Green	
1181	G. max	KLS72O-1	IT022602	EE-0035	South Korea	Landrace	Purple	Yellow	
	G. max	KLS625-1	IT024458	EE-0117	South Korea	Landrace			
1182	G. max	KLS721-2	IT022605	EE-0036	South Korea	Landrace	White	Yellow	
1183	G. max	KLS722	IT022606	EE-0037	South Korea	Landrace	White	Yellow	
1184	G. max	KLS724-2	IT022611	EE-0038	South Korea	Landrace	White	Yellow	
1185	G. max	KLS801-2	IT022639	EE-0041	South Korea	Landrace	White	Yellow	
1186	G. max	KLS807	PI 399024	EE-0043	South Korea	Landrace	Purple	Yellow	
1187	G. max	KLS815	IT022663	EE-0045	South Korea	Landrace	White	Green	
1188	G. max	KLS816	IT022664	EE-0046	South Korea	Landrace	Purple	Brown	
	G. max	PI 458302		DD-1212	South Korea	Landrace			
	G. max	YN113-2	IT024537	DD-0137	South Korea	Landrace			
1189	G. max	KLS819-2	IT022669	EE-0047	South Korea	Landrace	Purple	Yellow	
1190	G. max	KLS823-3	IT022676	EE-0048	South Korea	Landrace	Purple	Green	
	G. max	KLS 808-2	IT230831	EE-1211	South Korea	Landrace			
1191	G. max	PI 72227		EE-0052	China	Not determined	White	Yellow	
1192	G. max	PI 171434		EE-0056	China	Not determined	Purple	Yellow	
1193	G. max	Pyeongyang	IT023085	EE-0057	South Korea	Landrace	Purple	Green yellow	
1194	G. max	Sugye30	IT023248	EE-0058	South Korea	Improved line	Purple	Yellow	
1195	G. max	Sugye51	IT023261	EE-0061	South Korea	Improved line	Purple	Yellow	
1196	G. max	Sugye56	IT023265	EE-0065	South Korea	Improved line	Purple	Yellow	
1197	G. max	Bienville	PI 567788	EE-0067	United States	Improved line	Purple	Yellow	
1198	G. max	YN58-1	IT023817	EE-0071	South Korea	Landrace	Purple	Yellow	
1199	G. max	YN107	IT023845	EE-0072	South Korea	Landrace	Purple	Green	

1200	G. max	YN137-4	IT023874	EE-0076	South Korea	Landrace	Purple	Yellow	
	G. max	KLS77174-3	IT025447	EE-0196	South Korea	Landrace			
1201	G. max	YN213-1	IT023952	EE-0080	South Korea	Landrace	Purple	Yellow	
1202	G. max	YN214-2	IT023956	EE-0081	South Korea	Landrace	White	Yellow	
1203	G. max	YJ90	IT024019	EE-0085	South Korea	Landrace	Purple	Yellow	
	G. max	PI 82307		EE-0054	South Korea	Landrace			
	G. max	SLSJ-B-20	IT134289	EE-0319	South Korea	Landrace			
	G. max	PI83889		DD-0709	South Korea	Landrace			
	G. max	KAS530-23	IT141730	DD-0423	South Korea	Landrace			
	G. max	Chungbukwang	IT021591	EE-0002	South Korea	Landrace			
1204	G. max	YJ117-4	IT024038	EE-0087	South Korea	Landrace	Purple	Yellow	
1205	G. max	YJ174-2	IT024070	EE-0089	South Korea	Landrace	White	Yellow	
1206	G. max	YJ217-2	IT024106	EE-0090	South Korea	Landrace	White	Yellow	
1207	G. max	YJ219-4	IT024110	EE-0091	South Korea	Landrace	Purple	Yellow	
1208	G. max	YJ224-2	IT024120	EE-0093	South Korea	Landrace	Purple	Yellow	
1209	G. max	YN186-2	IT024150	EE-0096	South Korea	Landrace	Purple	Black	
1210	G. max	YJ80-3	IT024162	EE-0097	South Korea	Landrace	Purple	Yellow	
1211	G. max	YJ87	IT024167	EE-0098	South Korea	Landrace	Purple	Yellow	
1212	G. max	YJ100-4	IT024176	EE-0099	South Korea	Landrace	White	Yellow	
1213	G. max	YB11	IT024384	EE-0103	South Korea	Landrace	Purple	Black	
1214	G. max	YB330-1	IT024396	EE-0104	South Korea	Landrace	Purple	Black	
	G. max	Gganchi	IT186130	DD-0992	South Korea	Landrace			
	G. max	IT105525		DD-0251	South Korea	Landrace			
1215	G. max	YB331-2	IT024397	EE-0105	South Korea	Landrace	Purple	Brown	
1216	G. max	YJ102-3	IT024403	EE-0107	South Korea	Landrace	White	Yellow	
1217	G. max	YB133-2	IT024409	EE-0109	South Korea	Landrace	Purple	Yellow	
1218	G. max	YB148-4	IT024412	EE-0110	South Korea	Landrace	Purple	Yellow	
1219	G. max	YN108-2	IT024429	EE-0113	South Korea	Landrace	Purple	Black	
1220	G. max	YN101-4	IT024446	EE-0114	South Korea	Landrace	Purple	Green	
1221	G. max	YJ101-4	IT024452	EE-0116	South Korea	Landrace	White	Yellow	
1222	G. max	KAS895-1-26	IT024462	EE-0118	South Korea	Landrace	Purple	Yellow	
1223	G. max	YN1-2	IT024465	EE-0119	South Korea	Landrace	Purple	Yellow	
1224	G. max	YN12-3	IT024473	EE-0120	South Korea	Landrace	Purple	Yellow	
1225	G. max	Suwon58-3	IT024496	EE-0121	South Korea	Improved line	Purple	Yellow	
1226	G. max	YN67-1	IT024503	EE-0122	South Korea	Landrace	Purple	Yellow	
1227	G. max	YN74-2	IT024511	EE-0123	South Korea	Landrace	White	Yellow	
1228	G. max	YN121-1	IT024540	EE-0126	South Korea	Landrace	Purple	Yellow	
1229	G. max	YN124-1	IT024545	EE-0127	South Korea	Landrace	White	Black	
1230	G. max	YN139-2	IT024553	EE-0129	South Korea	Landrace	Purple	Green	Representative
1231	G. max	Suwon122	IT024558	EE-0131	South Korea	Improved line	Purple	Yellow	
1232	G. max	YN151-1	IT024565	EE-0132	South Korea	Landrace	Purple	Black	
	G. max	YN142-3	IT024555	EE-0130	South Korea	Landrace			
1233	G. max	YN158-1	IT024569	EE-0133	South Korea	Landrace	Purple	Brown	
1234	G. max	YN161-3	IT024572	EE-0134	South Korea	Landrace	Purple	Yellow	
1235	G. max	YN170-2	IT024576	EE-0135	South Korea	Landrace	Purple	Brown	
1236	G. max	YN177-1	IT024580	EE-0136	South Korea	Landrace	Purple	Green	
1237	G. max	YN193-3	IT024585	EE-0137	South Korea	Landrace	Purple	Green	
1238	G. max	YN203-4	IT024592	EE-0138	South Korea	Landrace	Purple	Black	
1239	G. max	YN234-1	IT024605	EE-0139	South Korea	Landrace	Purple	Green	
1240	G. max	YJ110-4	IT024658	EE-0145	South Korea	Landrace	Purple	Brown	
1241	G. max	YJ112-1	IT024659	EE-0146	South Korea	Landrace	White	Yellow	
1242	G. max	YJ116-4	IT024666	EE-0147	South Korea	Landrace	White	Yellow	
1243	G. max	YJ135-2	IT024673	EE-0148	South Korea	Landrace	Purple	Yellow	
1244	G. max	YJ168-2	IT024702	EE-0149	South Korea	Landrace	Purple	Yellow	
1245	G. max	YJ187-2	IT024720	EE-0150	South Korea	Landrace	Purple	Yellow	
1246	G. max	YJ214-2	IT024740	EE-0151	South Korea	Landrace	Purple	Yellow	
1247	G. max	YJ233-4	IT024760	EE-0153	South Korea	Landrace	White	Yellow	
1248	G. max	YJ237-2	IT024771	EE-0154	South Korea	Landrace	Purple	Yellow	
1249	G. max	KAS700-39	IT024780	EE-0156	South Korea	Landrace	Purple	Yellow	
1250	G. max	YJ251-4	IT024788	EE-0158	South Korea	Landrace	White	Yellow	
1251	G. max	YJ264-2	IT024798	EE-0159	South Korea	Landrace	Purple	Yellow	
1252	G. max	YJ273-1	IT024811	EE-0160	South Korea	Landrace	Purple	Brown	
1253	G. max	YB31-4	IT024879	EE-0162	South Korea	Landrace	Purple	Black	
	G. max	KAS578-27	IT181662	DD-0966	South Korea	Landrace			
	G. max	SLSB373-1	IT134424	EE-0327	South Korea	Landrace			
1254	G. max	YB78-1	IT024924	EE-0163	South Korea	Landrace	Purple	Brown	
1255	G. max	YB143-4	IT024975	EE-0166	South Korea	Landrace	Purple	Black	

1256	G. max	YB150-3	IT024981	EE-0167	South Korea	Landrace	Purple	Brown
1257	G. max	YB156	IT024985	EE-0168	South Korea	Landrace	Purple	Brown
1258	G. max	YB186-1	IT025018	EE-0169	South Korea	Landrace	Purple	Yellow
1259	G. max	YB195-1	IT024425	EE-0171	South Korea	Landrace	Purple	Green
1260	G. max	YB208-3	IT025053	EE-0172	South Korea	Landrace	Purple	Green
1261	G. max	YB211	IT025055	EE-0173	South Korea	Landrace	Purple	Black
1262	G. max	YB229	IT025076	EE-0175	South Korea	Landrace	Purple	Green
1263	G. max	YB247-2	IT025092	EE-0176	South Korea	Landrace	Purple	Green
1264	G. max	YB247-4	IT025094	EE-0177	South Korea	Landrace	Purple	Green
1265	G. max	YB270	IT025104	EE-0178	South Korea	Landrace	Purple	Black
	G. max	PI 458192		DD-1286	South Korea	Landrace		
	G. max	Jeonnamjinheungwon NO.16	IT178705	DD-0938	South Korea	Landrace		
1266	G. max	YB298	IT025127	EE-0179	South Korea	Landrace	Purple	Green
1267	G. max	KLS77011-1	IT025210	EE-0181	South Korea	Landrace	Purple	Green
1268	G. max	KLS77013	IT025215	EE-0183	South Korea	Landrace	Purple	Green
1269	G. max	KLS77030	IT025234	EE-0184	South Korea	Landrace	Purple	Yellow
1270	G. max	KLS77035	IT025240	EE-0185	South Korea	Landrace	White	Black
1271	G. max	KLS77077-4	IT025283	EE-0186	South Korea	Landrace	Purple	Brown
1272	G. max	KLS77094-4	IT025302	EE-0187	South Korea	Landrace	Purple	Yellow
1273	G. max	KLS77102-2	IT025313	EE-0188	South Korea	Landrace	White	Yellow
1274	G. max	KLS82001	IT025340	EE-0189	South Korea	Landrace	White	Yellow
1275	G. max	KLS77130-2	IT025367	EE-0192	South Korea	Landrace	Purple	Green yellow
1276	G. max	KLS77133-1	IT025374	EE-0193	South Korea	Landrace	White	Yellow
1277	G. max	KLS77147	IT025399	EE-0194	South Korea	Landrace	Purple	Brown
1278	G. max	KLS77154-2	IT025412	EE-0195	South Korea	Landrace	White	Brown
1279	G. max	KLS77181-3	IT025461	EE-0197	South Korea	Landrace	Purple	Yellow
1280	G. max	KLS77196-1	IT025484	EE-0199	South Korea	Landrace	Purple	Brown
1281	G. max	KLS77211	IT025509	EE-0201	South Korea	Landrace	Purple	Green
1282	G. max	IT102595		EE-0203	South Korea	Landrace	White	Yellow
1283	G. max	Yuwol	IT102611	EE-0204	South Korea	Landrace	White	Yellow
1284	G. max	IT102668		EE-0205	South Korea	Landrace	Purple	Yellow
1285	G. max	Kongnamul	IT102773	EE-0207	South Korea	Landrace	White	Yellow
1286	G. max	Hadaedu	IT103051	EE-0208	South Korea	Landrace	White	Yellow
1287	G. max	IT103189		EE-0209	South Korea	Landrace	Purple	Yellow
1288	G. max	Sasimil	IT103284	EE-0210	South Korea	Landrace	White	Yellow
1289	G. max	Meju_Jeonnam	IT103340	EE-0211	South Korea	Landrace	Purple	Yellow
1290	G. max	Meju	IT103501	EE-0213	South Korea	Landrace	White	Yellow
1291	G. max	Heuin	IT103691	EE-0214	South Korea	Landrace	Purple	Yellow
1292	G. max	Geomeun	IT103718	EE-0216	South Korea	Landrace	Purple	Black
1293	G. max	Hadaedu	IT103809	EE-0217	South Korea	Landrace	White	Yellow
1294	G. max	Kongnamul	IT103924	EE-0218	South Korea	Landrace	Purple	Green
1295	G. max	Ol	IT103926	EE-0219	South Korea	Landrace	White	Yellow
1296	G. max	IT104205		EE-0222	South Korea	Landrace	Purple	Yellow
1297	G. max	Ul	IT104361	EE-0223	South Korea	Landrace	White	Yellow
1298	G. max	Jilgeum	IT104411	EE-0224	South Korea	Landrace	White	Green
1299	G. max	Janjari	IT104564	EE-0227	South Korea	Landrace	White	Yellow
1300	G. max	Hadaedu	IT104627	EE-0229	South Korea	Landrace	Purple	Yellow
1301	G. max	Nunggamegi	IT104657	EE-0230	South Korea	Landrace	White	Yellow
1302	G. max	Geomjeongbul	IT104687	EE-0231	South Korea	Landrace	Purple	Black
1303	G. max	Kongnamul	IT105704	EE-0233	South Korea	Landrace	Purple	Yellow
1304	G. max	IT105874		EE-0234	South Korea	Landrace	White	Yellow
	G. max	Meoktae	IT105966	EE-0235	South Korea	Landrace		
1305	G. max	Bul	IT108710	EE-0237	South Korea	Landrace	Purple	Brown
1306	G. max	Nonduleong	IT109029	EE-0239	South Korea	Landrace	Purple	Green
1307	G. max	Kongnamul	IT111052	EE-0242	South Korea	Landrace	Purple	Yellow
1308	G. max	Paran	IT112838	EE-0245	South Korea	Landrace	Purple	Green
1309	G. max	Geomjeong	IT113067	EE-0246	South Korea	Landrace	Purple	Black
	G. max	KAS504-9	IT141666	DD-0415	South Korea	Landrace		
1310	G. max	Bam	IT113425	EE-0249	South Korea	Landrace	White	Black
1311	G. max	IT113572		EE-0250	South Korea	Landrace	Purple	Yellow
1312	G. max	KAS172-13	IT115372	EE-0252	South Korea	Landrace	Purple	Yellow
1313	G. max	KAS174-3	IT115380	EE-0253	South Korea	Landrace	Purple	Green yellow
	G. max	KAS608-10	IT141902	EE-0370	South Korea	Landrace		
1314	G. max	KAS210-7	PI 398452	EE-0255	South Korea	Landrace	Purple	Green
1315	G. max	KAS232-12	IT115436	EE-0257	South Korea	Landrace	Purple	Yellow
1316	G. max	KAS301-13	PI 458119	EE-0261	South Korea	Landrace	Purple	Black

1317	G. max	KAS301-16	PI 458122	EE-0262	South Korea	Landrace	Purple	Green
	G. max	PI 399101		DD-1253	South Korea	Landrace		
	G. max	PI 399121		DD-1256	South Korea	Landrace		
1318	G. max	KAS301-20	IT115510	EE-0263	South Korea	Landrace	Purple	Brown
1319	G. max	KAS302-9	IT115515	EE-0264	South Korea	Landrace	Purple	Yellow
	G. max	SLSJ198-1	IT134250	EE-0317	South Korea	Landrace		
1320	G. max	KAS312-8	IT115531	EE-0267	South Korea	Landrace	Purple	Yellow
1321	G. max	KAS320-20	IT115538	EE-0269	South Korea	Landrace	Purple	Yellow
1322	G. max	KAS320-25	IT115540	EE-0270	South Korea	Landrace	Purple	Yellow
1323	G. max	KAS330-3	IT115545	EE-0271	South Korea	Landrace	Purple	Yellow
	G. max	KAS540-23	PI 458180	EE-0279	South Korea	Landrace		
	G. max	KAS524-38	IT141728	EE-0349	South Korea	Landrace		
	G. max	KAS503-2	IT115597	DD-0308	South Korea	Landrace		
	G. max	KLS87205	IT153806	EE-0467	South Korea	Landrace		
1324	G. max	KAS331-13	IT115549	EE-0272	South Korea	Landrace	Purple	Brown
1325	G. max	KAS390-35	IT115589	EE-0273	South Korea	Landrace	Purple	Yellow
1326	G. max	KAS502-6	IT115594	EE-0274	South Korea	Landrace	Purple	Green
1327	G. max	KAS503-5	IT115598	EE-0275	South Korea	Landrace	Purple	Black
1328	G. max	KAS504-16	IT115606	EE-0276	South Korea	Landrace	Purple	Yellow
1329	G. max	KAS530-17	IT115645	EE-0277	South Korea	Landrace	Purple	Yellow
1330	G. max	KAS540-29	PI 458186	EE-0280	South Korea	Landrace	Purple	Black
1331	G. max	KAS551-3	IT115704	EE-0282	South Korea	Landrace	Purple	Yellow
1332	G. max	KAS551-18	IT115710	EE-0284	South Korea	Landrace	Purple	Green
1333	G. max	KAS571-24	IT115728	EE-0285	South Korea	Landrace	White	Black
	G. max	Geomjeong-5	IT177816	EE-0779	South Korea	Landrace		
1334	G. max	KAS604-1	IT115753	EE-0287	South Korea	Landrace	White	Yellow
1335	G. max	KAS629-2	PI 424135	EE-0288	South Korea	Landrace	Purple	Yellow
1336	G. max	KAS629-10	IT115768	EE-0289	South Korea	Landrace	White	Yellow
	G. max	PI 458217		EE-1038	South Korea	Landrace		
	G. max	YJ250-1	IT024785	DD-0161	South Korea	Landrace		
1337	G. max	KAS630-8	IT115771	EE-0290	South Korea	Landrace	White	Yellow
1338	G. max	KAS632-13	PI 424544	EE-0292	South Korea	Landrace	Purple	Brown
1339	G. max	KAS636-9	PI 424556	EE-0293	South Korea	Landrace	Purple	Black
1340	G. max	KAS640-14	IT115809	EE-0294	South Korea	Landrace	Purple	Yellow
1341	G. max	KAS660-14	IT115869	EE-0299	South Korea	Landrace	Purple	Yellow
1342	G. max	KAS660-20	IT115871	EE-0300	South Korea	Landrace	Purple	Green yellow
1343	G. max	KAS663-8	IT115880	EE-0301	South Korea	Landrace	Purple	Yellow
1344	G. max	KAS679-5	IT115889	EE-0302	South Korea	Landrace	White	Yellow
1345	G. max	KAS681-20	PI 424607	EE-0303	South Korea	Landrace	Purple	Brown
1346	G. max	RAIKO	IT120618	EE-0304	Japan	Improved line	White	Yellow
1347	G. max	PI96550		EE-0305	North Korea	Not determined	Purple	Yellow
1348	G. max	KANRICH	PI 548552	EE-0306	United States	Improved line	Purple	Yellow
1349	G. max	Ajuggalibam	IT120728	EE-0307	South Korea	Landrace	Purple	Brown
	G. max	IT186079		EE-0833	South Korea	Landrace		
	G. max	KLS87158	IT153768	DD-0552	South Korea	Landrace		
	G. max	Gyungjuhwangsaek	IT228630	DD-1295	South Korea	Landrace		
	G. max	kwangwon sujib4-6	IT156196	EE-0557	South Korea	Landrace		
	G. max	KLS77011-2	IT025211	EE-0182	South Korea	Landrace		
1350	G. max	Kongnamul	IT121463	EE-0308	South Korea	Landrace	Purple	Yellow
1351	G. max	Gulgeun	IT121480	EE-0309	South Korea	Landrace	Purple	Yellow
1352	G. max	Milyang11	IT134178	EE-0311	South Korea	Improved line	Purple	Yellow
1353	G. max	SLSJ134-2	IT134231	EE-0313	South Korea	Landrace	Purple	Yellow
1354	G. max	SLSJ150-2	IT134237	EE-0314	South Korea	Landrace	White	Yellow
1355	G. max	SLSJ190-2	IT134248	EE-0316	South Korea	Landrace	Purple	Yellow
	G. max	KAS660-13	IT154754	EE-0530	South Korea	Landrace		
	G. max	KAS506-1	IT141670	EE-0343	South Korea	Landrace		
	G. max	KAS549-12	IT115696	DD-0321	South Korea	Landrace		
	G. max	SLSJ113-2	IT134222	DD-0360	South Korea	Landrace		
1356	G. max	SLSN294-2	IT134309	EE-0320	South Korea	Landrace	Purple	Black
1357	G. max	Aobatake	IT134331	EE-0323	South Korea	Landrace	Purple	Green
1358	G. max	SLSB196-1	IT134394	EE-0324	South Korea	Landrace	Purple	Brown
1359	G. max	SLSB197-1	IT134395	EE-0325	South Korea	Landrace	Purple	Green yellow
1360	G. max	SLSB339-1	IT134420	EE-0326	South Korea	Landrace	Purple	Green
1361	G. max	SLSB-B-1	IT134460	EE-0328	South Korea	Landrace	Purple	Brown
	G. max	KAS371-16	IT141654	EE-0341	South Korea	Landrace		
1362	G. max	SLSB-B-10	IT134465	EE-0329	South Korea	Landrace	Purple	Brown

1363	G. max	Nonglim73	IT135700	EE-0331	Japan	Improved line	Purple	Yellow
1364	G. max	KAS310-2	IT141537	EE-0335	South Korea	Landrace	White	Yellow
1365	G. max	KAS361-23	IT141628	EE-0337	South Korea	Landrace	Purple	Yellow
1366	G. max	KAS361-24	IT141629	EE-0338	South Korea	Landrace	Purple	Brown
1367	G. max	KAS370-20	PI 423839	EE-0340	South Korea	Landrace	Purple	Yellow
1368	G. max	KAS503-19	IT141664	EE-0342	South Korea	Landrace	White	Yellow
1369	G. max	KAS510-11	IT141674	EE-0344	South Korea	Landrace	Purple	Black
1370	G. max	KAS513-7	IT141686	EE-0345	South Korea	Landrace	Purple	Black
1371	G. max	KAS523-12	IT141697	EE-0346	South Korea	Landrace	White	Yellow
	G. max	PI 424433		EE-0966	South Korea	Landrace		
	G. max	Namul	IT196831	EE-0861	South Korea	Landrace		
	G. max	Kongnamul	IT178646	DD-0933	South Korea	Landrace		
	G. max	Jeonnam Gokseong-1997-35	IT219320	DD-1067	South Korea	Landrace		
1372	G. max	KAS523-26	IT141707	EE-0347	South Korea	Landrace	Purple	Yellow
1373	G. max	KAS523-35	IT141712	EE-0348	South Korea	Landrace	Purple	Yellow
1374	G. max	KAS540-17	PI 424423	EE-0350	South Korea	Landrace	White	Yellow
1375	G. max	KAS542-18	IT141753	EE-0351	South Korea	Landrace	Purple	Black
1376	G. max	KAS547-2	PI 408006	EE-0352	South Korea	Landrace	Purple	Yellow
1377	G. max	KAS573-9	PI 458218	EE-0355	South Korea	Landrace	Purple	Black
1378	G. max	KAS574-21	IT141794	EE-0356	South Korea	Landrace	White	Yellow
1379	G. max	KAS580-18	IT141830	EE-0359	South Korea	Landrace	Purple	Green
1380	G. max	KAS581-9	PI 424461	EE-0360	South Korea	Landrace	Purple	Green
1381	G. max	KAS602-12	IT141865	EE-0363	South Korea	Landrace	Purple	Black
1382	G. max	KAS603-12	IT141868	EE-0365	South Korea	Landrace	Purple	Yellow
1383	G. max	KAS604-24	IT141871	EE-0366	South Korea	Landrace	Purple	Green
1384	G. max	KAS605-1	IT141872	EE-0367	South Korea	Landrace	Purple	Green
1385	G. max	KAS606-31	IT141892	EE-0368	South Korea	Landrace	Purple	Black
1386	G. max	KAS607-8	IT141899	EE-0369	South Korea	Landrace	Purple	Brown
1387	G. max	KAS616-2	IT141928	EE-0373	South Korea	Landrace	White	Yellow
1388	G. max	KAS620-5	IT141937	EE-0374	South Korea	Landrace	Purple	Yellow
1389	G. max	KAS620-11	IT141941	EE-0375	South Korea	Landrace	Purple	Black
1390	G. max	KAS624-14	IT141954	EE-0376	South Korea	Landrace	Purple	Black
1391	G. max	KAS630-18	PI 509102	EE-0379	South Korea	Landrace	Purple	Green
	G. max	KAS624-1	IT154427	DD-0584	South Korea	Landrace		
	G. max	KAS624-2	IT141952	DD-0447	South Korea	Landrace		
1392	G. max	Kangwonhadaedu	IT142777	EE-0382	South Korea	Landrace	Purple	Yellow
1393	G. max	Suwon135	IT142793	EE-0383	South Korea	Improved line	Purple	Yellow
1394	G. max	Baengcheon	IT142805	EE-0384	South Korea	Improved line	White	Yellow
	G. max	Baekchun)	IT228329	EE-1051	South Korea	Improved line		
	G. max	Baegcheon	IT230771	EE-1194	South Korea	Landrace		
	G. max	Baekchun	PI 483082 A	DD-1213	South Korea	Improved line		
	G. max	Hakusen	IT230703	DD-1337	South Korea	Landrace		
1395	G. max	KLS89091	IT142871	EE-0385	South Korea	Landrace	Purple	Green
	G. max	Jejusi-KHC-1999-6	IT212800	DD-1049	South Korea	Landrace		
	G. max	Ilinnangjidu	IT021778	CD-1377	South Korea	Not determined		
1396	G. max	KLS86097	IT142891	EE-0386	South Korea	Landrace	Purple	Yellow
1397	G. max	KLS86098	IT142894	EE-0387	South Korea	Landrace	Purple	Black
1398	G. max	KLS86104	IT142912	EE-0388	South Korea	Landrace	Purple	Yellow
	G. max	Sugae27	IT023246	DD-0059	South Korea	Improved line		
	G. max	HANKYONG-JEOJI#1	IT142874	DD-0465	South Korea	Landrace		
1399	G. max	KLS86110	IT142920	EE-0389	South Korea	Landrace	Purple	Green
1400	G. max	KLS85010	IT142952	EE-0391	South Korea	Landrace	White	Yellow
1401	G. max	KLS85017	IT142959	EE-0392	South Korea	Landrace	White	Yellow
1402	G. max	KLS85019	IT142961	EE-0393	South Korea	Landrace	White	Yellow
1403	G. max	KLS85026	IT142968	EE-0394	South Korea	Landrace	White	Yellow
1404	G. max	KLS85042	IT142984	EE-0396	South Korea	Landrace	White	Yellow
1405	G. max	KLS85044	IT142986	EE-0397	South Korea	Landrace	White	Yellow
1406	G. max	KLS85045	IT142987	EE-0398	South Korea	Landrace	White	Yellow
1407	G. max	KLS85059	IT143001	EE-0400	South Korea	Landrace	White	Yellow
1408	G. max	KLS85069	IT143011	EE-0401	South Korea	Landrace	White	Yellow
1409	G. max	KLS85071	IT143013	EE-0402	South Korea	Landrace	Purple	Yellow
1410	G. max	KLS85079	IT143020	EE-0403	South Korea	Landrace	White	Yellow
1411	G. max	KLS85101	IT143042	EE-0404	South Korea	Landrace	White	Yellow
1412	G. max	KLS85106	IT143047	EE-0406	South Korea	Landrace	White	Yellow
1413	G. max	KLS85109	IT143050	EE-0407	South Korea	Landrace	White	Yellow

1414	G. max	KLS85119	IT143060	EE-0408	South Korea	Landrace	White	Yellow
1415	G. max	KLS85132	IT143073	EE-0411	South Korea	Landrace	White	Yellow
1416	G. max	KLS85135	IT143076	EE-0412	South Korea	Landrace	White	Yellow
1417	G. max	KLS85163	IT143084	EE-0413	South Korea	Landrace	White	Yellow
1418	G. max	KLS85164	IT143085	EE-0414	South Korea	Landrace	Purple	Yellow
1419	G. max	KLS85179	IT143098	EE-0415	South Korea	Landrace	Purple	Green
1420	G. max	KLS85181	IT143100	EE-0416	South Korea	Landrace	Purple	Black
1421	G. max	KLS85182	IT143101	EE-0417	South Korea	Landrace	White	Green
1422	G. max	KLS85188	IT143107	EE-0418	South Korea	Landrace	Purple	Black
1423	G. max	KLS85197	IT143116	EE-0419	South Korea	Landrace	Purple	Yellow
1424	G. max	KLS85202	IT143121	EE-0420	South Korea	Landrace	White	Black
1425	G. max	KLS85228	IT143146	EE-0423	South Korea	Landrace	Purple	Yellow
1426	G. max	KLS85240	IT143157	EE-0424	South Korea	Landrace	White	Yellow
1427	G. max	KLS85242	IT143159	EE-0425	South Korea	Landrace	Purple	Black
1428	G. max	KLS85247	IT143164	EE-0427	South Korea	Landrace	White	Yellow
1429	G. max	KLS85250	IT143167	EE-0428	South Korea	Landrace	Purple	Black
1430	G. max	KLS86037	IT143237	EE-0434	South Korea	Landrace	White	Black
1431	G. max	KLS86059	IT143259	EE-0435	South Korea	Landrace	Purple	Green
1432	G. max	KLS86141	IT143304	EE-0436	South Korea	Landrace	Purple	Black
1433	G. max	KLS86166	IT143328	EE-0437	South Korea	Landrace	White	Yellow
1434	G. max	KLS85156	IT143369	EE-0439	South Korea	Landrace	White	Yellow
1435	G. max	KLS85158	IT143371	EE-0440	South Korea	Landrace	White	Yellow
1436	G. max	Suwon134	IT153338	EE-0441	South Korea	Improved line	Purple	Yellow
1437	G. max	KLS087019	IT153356	EE-0443	South Korea	Landrace	Purple	Black
1438	G. max	KLS087025	IT153359	EE-0444	South Korea	Landrace	White	Yellow
1439	G. max	KLS087160	IT153411	EE-0448	South Korea	Landrace	Purple	Yellow
1440	G. max	KLS087171	IT153415	EE-0449	South Korea	Landrace	Purple	Yellow
1441	G. max	KLS087211	IT153421	EE-0450	South Korea	Landrace	Purple	Black
1442	G. max	KLS87058	IT153682	EE-0455	South Korea	Landrace	Purple	Black
1443	G. max	KLS87065	IT153689	EE-0456	South Korea	Landrace	Purple	Yellow
	G. max	KLS86124	IT143178	EE-0432	South Korea	Landrace		
	G. max	KwangkyoV1		KwangkyoV1	South Korea			
	G. max	SLS90-165	IT167803	DD-0817	South Korea	Landrace		
	G. max	Jang	IT239895	DD-1409	South Korea	Landrace		
	G. max	Pyungbuktae	IT021757	CD-1371	South Korea	Landrace		
	G. max	Kwangkyo		kwangkyo	South Korea			
	G. max	Kwangkyo	IT023534	DD-0061	South Korea	Improved line		
	G. max	YJ302-3	IT024849	DD-0169	South Korea	Landrace		
	G. max	IT103954		DD-0231	South Korea	Landrace		
	G. max	IT103960		DD-0232	South Korea	Landrace		
	G. max	Kwangkyo	IT023554	EE-0068	South Korea	Improved line		
	G. max	Jang	IT224175	EE-0943	South Korea	Landrace		
	G. max	Gausari	IT105640	DD-0253	South Korea	Landrace		
	G. max	Kwangkyo	IT023515	EE-0066	South Korea	Improved line		
	G. max	YJ83	IT024643	EE-0144	South Korea	Landrace		
	G. max	KLS87036	IT156251	EE-0559	South Korea	Landrace		
	G. max	SLS;90-130	IT167768	EE-0714	South Korea	Landrace		
	G. max	Kwangkyo		KwangkyoV2	South Korea	Improved line		
	G. max	Kwangkyo		Kwangkyo	South Korea	Improved line		
	G. max	Meju	IT180844	DD-0952	South Korea	Landrace		
	G. max	Sugae39	IT021780	CD-1378	South Korea	Improved line		
	G. max	KLS511	IT022517	DD-0026	South Korea	Landrace		
	G. max	PI84683A		DD-0055	South Korea	Landrace		
1444	G. max	KLS87076	IT153698	EE-0457	South Korea	Landrace	White	Brown
1445	G. max	KLS87114	IT153731	EE-0461	South Korea	Landrace	White	Black
1446	G. max	KLS87178	IT153783	EE-0462	South Korea	Landrace	Purple	Yellow
1447	G. max	KLS87179	IT153784	EE-0463	South Korea	Landrace	Purple	Yellow
1448	G. max	KLS87185	IT153790	EE-0465	South Korea	Landrace	White	Yellow
1449	G. max	KLS87193	IT153798	EE-0466	South Korea	Landrace	Purple	Yellow
1450	G. max	KLS87207	IT153807	EE-0468	South Korea	Landrace	Purple	Yellow
1451	G. max	KLS87210	IT153810	EE-0469	South Korea	Landrace	Purple	Yellow
1452	G. max	KLS87220	IT153820	EE-0471	South Korea	Landrace	Purple	Black
1453	G. max	KLS87241	IT153837	EE-0474	South Korea	Landrace	White	Black
1454	G. max	KLS87313	IT153891	EE-0478	South Korea	Landrace	Purple	Black
1455	G. max	KLS87316	IT153893	EE-0479	South Korea	Landrace	White	Black

	G. max	KLS85209	IT143128	EE-0422	South Korea	Landrace			
	G. max	PI 458062 A		EE-1147	South Korea	Landrace			
	G. max	IT186036		DD-0978	South Korea	Landrace			
	G. max	KAS 210-9	PI 567280	DD-1216	South Korea	Landrace			
1456	G. max	KAS380-11	IT154169	EE-0485	South Korea	Landrace	Purple	Black	
1457	G. max	KAS380-16	PI 423844	EE-0486	South Korea	Landrace	Purple	Dark brown	
1458	G. max	KAS380-18	IT154175	EE-0487	South Korea	Landrace	Purple	Yellow	
1459	G. max	KAS390-9	IT154183	EE-0488	South Korea	Landrace	Purple	Yellow	
1460	G. max	KAS391-2	IT154202	EE-0489	South Korea	Landrace	Purple	Yellow	
1461	G. max	KAS503-8	IT154217	EE-0491	South Korea	Landrace	Purple	Yellow	
1462	G. max	KAS521-1	IT154255	EE-0493	South Korea	Landrace	White	Green	
1463	G. max	KAS521-6	IT154258	EE-0494	South Korea	Landrace	Purple	Yellow	
1464	G. max	KAS524-3	PI 407897	EE-0495	South Korea	Landrace	Purple	Yellow	
1465	G. max	KAS530-4	PI 407906	EE-0496	South Korea	Landrace	Purple	Green	
1466	G. max	KAS574-10	IT154319	EE-0498	South Korea	Landrace	Purple	Black	
1467	G. max	KAS575-1	IT154323	EE-0499	South Korea	Landrace	Purple	Green	
1468	G. max	KAS633-26	IT154512	EE-0508	South Korea	Landrace	Purple	Yellow	
1469	G. max	KAS634-17	IT154523	EE-0509	South Korea	Landrace	Purple	Yellow	
1470	G. max	KAS637-2	PI 408116	EE-0512	South Korea	Landrace	Purple	Yellow	
1471	G. max	KAS637-5	PI 408119	EE-0513	South Korea	Landrace	Purple	Yellow	
1472	G. max	KAS639-4	IT154583	EE-0517	South Korea	Landrace	Purple	Black	
1473	G. max	KAS640-3	IT154603	EE-0518	South Korea	Landrace	Purple	Green	
1474	G. max	KAS640-19	PI 424574	EE-0519	South Korea	Landrace	Purple	Yellow	
1475	G. max	KAS640-46	IT154621	EE-0520	South Korea	Landrace	Purple	Green	
	G. max	KAS579-4	IT219638	DD-1084	South Korea	Landrace			
1476	G. max	KAS642-3	PI 424149	EE-0521	South Korea	Landrace	Purple	Black	
1477	G. max	KAS644-12	IT154668	EE-0522	South Korea	Landrace	Purple	Yellow	
1478	G. max	KAS644-17	IT154673	EE-0523	South Korea	Landrace	Purple	Green	
1479	G. max	KAS646-4	IT154691	EE-0524	South Korea	Landrace	Purple	Black	
1480	G. max	KAS651-16	PI 424581	EE-0526	South Korea	Landrace	Purple	Brown	
1481	G. max	KAS651-42	IT154739	EE-0528	South Korea	Landrace	Purple	Black	
1482	G. max	KAS660-9	IT154751	EE-0529	South Korea	Landrace	White	Yellow	
1483	G. max	KAS660-16	IT154756	EE-0531	South Korea	Landrace	Purple	Green	
1484	G. max	Dongsan103	IT154772	EE-0532	Japan	Improved line	Purple	Yellow	
1485	G. max	Dongsan118	IT154778	EE-0533	Japan	Improved line	Purple	Yellow	
1486	G. max	Milyang23	IT154802	EE-0535	South Korea	Improved line	White	Yellow	
1487	G. max	Mokpo6	IT154805	EE-0536	South Korea	Improved line	Purple	Yellow	
1488	G. max	KLS87097	IT155938	EE-0539	South Korea	Landrace	White	Yellow	
1489	G. max	KLS87277	IT155954	EE-0540	South Korea	Landrace	Purple	Green	
1490	G. max	Nongrim51	IT155963	EE-0541	Japan	Improved line	White	Yellow	
1491	G. max	KLS87199	IT155994	EE-0543	South Korea	Landrace	Purple	Yellow	
1492	G. max	Dongsan131	IT156093	EE-0544	Japan	Improved line	Purple	Yellow	
1493	G. max	Kangwon sujib1-1	IT156123	EE-0545	South Korea	Landrace	Purple	Yellow	
1494	G. max	Kangwon sujib2-16	IT156144	EE-0547	South Korea	Landrace	Purple	Black	
1495	G. max	Kangwon sujib2-17	IT156145	EE-0548	South Korea	Landrace	Purple	Black	
1496	G. max	Kangwon sujib2-24	IT156152	EE-0549	South Korea	Landrace	Purple	Yellow	
1497	G. max	Kangwon sujib3-10	IT156170	EE-0551	South Korea	Landrace	Purple	Green yellow	
1498	G. max	Kangwon sujib3-11	IT156171	EE-0552	South Korea	Landrace	Purple	Green	
1499	G. max	Kangwon sujib3-24	IT156183	EE-0553	South Korea	Landrace	Purple	Green	
	G. max	Geomjeong-6	IT178177	EE-0789	South Korea	Landrace			
	G. max	Babmit	IT195510	DD-1023	South Korea	Landrace			
	G. max	kwangwonsujib3-28	IT156187	EE-0554	South Korea	Landrace			
	G. max	kwangwonsujib3-30	IT156189	EE-0555	South Korea	Landrace			
	G. max	KAS 200-34	PI 423750	EE-1142	South Korea	Landrace			
	G. max	IT186044		EE-0832	South Korea	Landrace			
1500	G. max	Kangwon sujib3-31	IT156190	EE-0556	South Korea	Landrace	Purple	Green	
1501	G. max	IT156217	Kangwon sujib5-19	EE-0558	South Korea	Landrace	Purple	Green	Representative
1502	G. max	KLS87345	IT156261	EE-0560	South Korea	Landrace	White	Yellow	
1503	G. max	Bongwhoa SLSN-3	IT157861	EE-0561	South Korea	Landrace	Purple	Brown	
1504	G. max	gyeonggi29	IT157890	EE-0562	South Korea	Improved line	White	Yellow	
	G. max	KLS85038	IT142980	EE-0395	South Korea	Landrace			
	G. max	KLS85092	IT143033	DD-0491	South Korea	Landrace			
	G. max	IT178456		DD-0930	South Korea	Landrace			
	G. max	SLS90-337	IT167975	DD-0828	South Korea	Landrace			
	G. max	Yuwol	IT101434	EE-0202	South Korea	Landrace			

1505	G. max	Milyang24	IT157942	EE-0564	South Korea	Improved line	White	Yellow	
1506	G. max	Monroe	PI 548599	EE-0565	United States	Improved line	White	Yellow	
1507	G. max	SLS B16	IT158121	EE-0568	South Korea	Landrace	Purple	Yellow	
1508	G. max	SLS B37	IT158126	EE-0569	South Korea	Landrace	Purple	Yellow	
1509	G. max	SLS B229-2-3	IT158137	EE-0571	South Korea	Landrace	Purple	Yellow	
1510	G. max	SLS B408-1	IT158145	EE-0572	South Korea	Landrace	Purple	Black	
1511	G. max	Deokyu	IT158148	EE-0573	South Korea	Improved line	Purple	Yellow	
1512	G. max	Tousan 65	PI 507403	EE-0574	Japan	Improved line	Purple	Yellow	
1513	G. max	Noksaekbam	IT158248	EE-0576	South Korea	Landrace	Purple	Green	
1514	G. max	KLS88042-1	IT160104	EE-0577	South Korea	Landrace	White	Yellow	
1515	G. max	KLS88045	IT160108	EE-0578	South Korea	Landrace	White	Yellow	
1516	G. max	KLS88046	IT160109	EE-0579	South Korea	Landrace	White	Yellow	
1517	G. max	KLS88061	IT160123	EE-0580	South Korea	Landrace	Purple	Black	
1518	G. max	KLS88078	IT160141	EE-0581	South Korea	Landrace	Purple	Black	
1519	G. max	KLS88087	IT160150	EE-0582	South Korea	Landrace	Purple	Yellow	
1520	G. max	PI 90763		EE-0583	China	Not determined	Purple	Black	Representative
1521	G. max	PI 86892		EE-0588	South Korea	Landrace	Purple	Green	
1522	G. max	PI 87037		EE-0589	South Korea	Landrace	Purple	Yellow	
1523	G. max	PI 89154-S		EE-0590	North Korea	Not determined	Purple	Yellow	
1524	G. max	PI 96786		EE-0591	North Korea	Not determined	White	Yellow	
1525	G. max	PI 81037		EE-0593	Japan	Not determined	Purple	Yellow	
1526	G. max	PI 87002		EE-0594	South Korea	Landrace	White	Yellow	
1527	G. max	PI 88816-S		EE-0595	North Korea	Not determined	White	Yellow	
1528	G. max	PI 95969		EE-0596	North Korea	Landrace	Purple	Yellow	
1529	G. max	PI 96035		EE-0597	North Korea	Not determined	White	Yellow	
1530	G. max	PI 96354		EE-0598	North Korea	Not determined	White	Yellow	
1531	G. max	PI 85416		EE-0599	South Korea	Landrace	White	Yellow	
1532	G. max	PI 95960		EE-0600	North Korea	Landrace	White	Yellow	
1533	G. max	PI 54818		EE-0602	China	Not determined	White	Yellow	
1534	G. max	PI 58955		EE-0603	China	Not determined	White	Yellow	
1535	G. max	PI 68696		EE-0604	China	Not determined	Purple	Yellow	
1536	G. max	PI 68770		EE-0605	China	Not determined	Purple	Yellow	
1537	G. max	PI 82184		EE-0606	South Korea	Landrace	Purple	Yellow	
1538	G. max	PI 82235		EE-0607	South Korea	Landrace	Purple	Brown	
1539	G. max	PI 82295		EE-0608	South Korea	Landrace	Purple	Yellow	
	G. max	PI 458052		EE-1145	South Korea	Landrace			
	G. max	PI 83874		EE-0587	North Korea	Not determined			
1540	G. max	PI 82302		EE-0609	South Korea	Landrace	White	Yellow	
1541	G. max	PI 82312N		EE-0610	South Korea	Landrace	Purple	Yellow	
1542	G. max	PI 82527		EE-0612	North Korea	Not determined	Purple	Yellow	
1543	G. max	PI 82555		EE-0613	South Korea	Landrace	Purple	Yellow	
1544	G. max	PI 82558		EE-0614	South Korea	Landrace	White	Yellow	
1545	G. max	PI 83853		EE-0615	South Korea	Landrace	Purple	Yellow	
1546	G. max	PI 83892		EE-0616	South Korea	Landrace	Purple	Yellow	
1547	G. max	PI 83915		EE-0617	South Korea	Landrace	White	Yellow	
1548	G. max	Buchae	IT161901	EE-0618	South Korea	Landrace	White	Yellow	
1549	G. max	PI 84609		EE-0620	South Korea	Landrace	Purple	Black	
1550	G. max	PI 84633		EE-0621	South Korea	Landrace	White	Yellow	
1551	G. max	PI 84637		EE-0622	South Korea	Landrace	Purple	Yellow	Representative
1552	G. max	PI 84646		EE-0623	South Korea	Landrace	Purple	Yellow	
1553	G. max	PI 84657		EE-0624	South Korea	Landrace	Purple	Yellow	
1554	G. max	PI 84660		EE-0625	South Korea	Landrace	Purple	Yellow	
1555	G. max	PI 84665		EE-0627	South Korea	Landrace	Purple	Yellow	
1556	G. max	PI 84669N		EE-0628	South Korea	Landrace	White	Yellow	
1557	G. max	PI 84896		EE-0631	South Korea	Landrace	White	Yellow	
1558	G. max	PI 84912		EE-0632	North Korea	Not determined	White	Yellow	
1559	G. max	PI 84946-1		EE-0633	South Korea	Not determined	White	Yellow	
1560	G. max	PI 84946-2		EE-0634	South Korea	Not determined	Purple	Yellow	
1561	G. max	PI 84987A		EE-0635	Japan	Not determined	Purple	Yellow	
1562	G. max	PI 85340		EE-0636	South Korea	Landrace	Purple	Yellow	
	G. max	PI 84682		EE-0629	South Korea	Landrace			
1563	G. max	PI 85407		EE-0637	South Korea	Landrace	White	Yellow	
1564	G. max	PI 85437		EE-0638	South Korea	Landrace	Purple	Yellow	
1565	G. max	PI 85456		EE-0639	South Korea	Landrace	White	Yellow	
1566	G. max	PI 85505		EE-0640	South Korea	Landrace	Purple	Yellow	
	G. max	PI 398878		EE-0985	South Korea	Landrace			
	G. max	KLS85205	IT143124	EE-0421	South Korea	Landrace			

	G. max	Chae-rae-chong	IT226749	EE-0976	South Korea	Landrace			
1567	G. max	PI 85506		EE-0641	South Korea	Landrace	Purple	Yellow	
	G. max	PI 87029		DD-0738	South Korea	Landrace			
	G. max	PI 86903-3		DD-0733	South Korea	Not determined			
1568	G. max	PI 85519		EE-0643	South Korea	Landrace	Purple	Yellow	
1569	G. max	PI 86972-1		EE-0645	South Korea	Not determined	Purple	Yellow	
1570	G. max	PI 86972-2		EE-0646	South Korea	Not determined	Purple	Yellow	
	G. max	Kujongumurukon	PI 86908	EE-1246	South Korea	Landrace			
1571	G. max	PI 87540		EE-0649	North Korea	Not determined	Purple	Black	Representative
1572	G. max	PI 87600-2		EE-0650	South Korea	Not determined	Purple	Yellow	
1573	G. max	PI 87619-1		EE-0651	North Korea	Not determined	Purple	Yellow	
1574	G. max	PI 87632		EE-0653	Japan	Not determined	Purple	Yellow	
1575	G. max	PI 89138		EE-0655	North Korea	Not determined	Purple	Yellow	
1576	G. max	PI 89162		EE-0656	North Korea	Not determined	Purple	Yellow	
1577	G. max	PI 90245		EE-0659	North Korea	Not determined	White	Yellow	
1578	G. max	PI 91073		EE-0660	South Korea	Landrace	Purple	Brown	
1579	G. max	PI 91679		EE-0661	North Korea	Not determined	White	Yellow	Representative
1580	G. max	PI 91719		EE-0662	North Korea	Not determined	Purple	Yellow	
1581	G. max	PI 91729		EE-0663	North Korea	Not determined	White	Brown	
	G. max	PI 84631		DD-0716	South Korea	Landrace			
	G. max	PI 96321		DD-0764	North Korea	Not determined			
1582	G. max	PI 92568		EE-0664	China	Not determined	Purple	Yellow	Representative
1583	G. max	PI 92590		EE-0665	China	Not determined	White	Yellow	
1584	G. max	PI 92688		EE-0666	China	Not determined	White	Yellow	
1585	G. max	PI 93559		EE-0667	China	Not determined	Purple	Yellow	
1586	G. max	PI 96171		EE-0668	North Korea	Not determined	White	Yellow	Representative
1587	G. max	PI 96333		EE-0669	North Korea	Not determined	Purple	Green	
1588	G. max	PI 96549		EE-0670	North Korea	Not determined	Purple	Yellow	
1589	G. max	PI 96927		EE-0672	North Korea	Not determined	Purple	Yellow	
1590	G. max	PI 97139		EE-0673	North Korea	Not determined	White	Yellow	Representative
1591	G. max	PI 97225		EE-0674	North Korea	Not determined	Purple	Yellow	
1592	G. max	Ham-hung-dal-ri	PI 157424	EE-0677	South Korea	Landrace	Purple	Yellow	
1593	G. max	Huktae	IT162668	EE-0681	South Korea	Landrace	Purple	Black	
1594	G. max	Buseok	IT162669	EE-0682	South Korea	Landrace	Purple	Yellow	
1595	G. max	Hoengseonggun	IT162679	EE-0683	South Korea	Landrace	White	Yellow	
1596	G. max	Toyo-naga	PI 243547	EE-0685	Japan	Not determined	Purple	Yellow	Representative
1597	G. max	Pixie	PI 543856	EE-0686	United States	Improved line	Purple	Yellow	
1598	G. max	Meju_Jeonbuk	IT162760	EE-0688	South Korea	Landrace	White	Yellow	
1599	G. max	Kongnamul	IT162810	EE-0689	South Korea	Landrace	Purple	Yellow	
1600	G. max	Suwon148	IT163421	EE-0690	South Korea	Improved line	White	Yellow	
1601	G. max	Mokpo10	IT163423	EE-0691	South Korea	Improved line	White	Yellow	
1602	G. max	KLS88008	IT163555	EE-0692	South Korea	Landrace	White	Yellow	
1603	G. max	KLS88021	IT163568	EE-0693	South Korea	Landrace	White	Yellow	
1604	G. max	KLS88027	IT163574	EE-0694	South Korea	Landrace	White	Yellow	
1605	G. max	BSR 302	PI 548525	EE-0695	United States	Improved line	Purple	Yellow	
1606	G. max	Desoto	PI 548549	EE-0697	United States	Improved line	Purple	Yellow	
1607	G. max	Dowling	PI 548663	EE-0698	United States	Improved line	White	Yellow	
1608	G. max	Kershaw	PI 548985	EE-0700	United States	Improved line	White	Yellow	
1609	G. max	Preston	PI 548520	EE-0701	United States	Improved line	Purple	Yellow	
1610	G. max	Vansoy	PI 548623	EE-0702	United States	Improved line	White	Yellow	
1611	G. max	PI475783A		EE-0704	China	Not determined	Purple	Yellow	
1612	G. max	PI 475812 B		EE-0705	China	Not determined	Purple	Yellow	Representative
1613	G. max	PI475824A		EE-0707	China	Not determined	Purple	Yellow	
1614	G. max	SLS90-5	IT167643	EE-0708	South Korea	Landrace	White	Yellow	
1615	G. max	SLS90-23	IT167661	EE-0709	South Korea	Landrace	White	Yellow	
1616	G. max	SLS90-26	IT167664	EE-0710	South Korea	Landrace	White	Yellow	
1617	G. max	SLS90-83	IT167721	EE-0711	South Korea	Landrace	White	Yellow	
	G. max	SLS90-84	IT167722	DD-0810	South Korea	Landrace			
	G. max	Jangba다	IT021902	DD-0002	South Korea	Improved line			
1618	G. max	SLS90-101	IT167739	EE-0712	South Korea	Landrace	Purple	Green	
1619	G. max	SLS90-108	IT167746	EE-0713	South Korea	Landrace	Purple	Green	
1620	G. max	SLS90-146	IT167784	EE-0717	South Korea	Landrace	Purple	Yellow	
1621	G. max	SLS90-210	IT167848	EE-0718	South Korea	Landrace	White	Yellow	
1622	G. max	SLS90-312	IT167950	EE-0722	South Korea	Landrace	White	Yellow	
1623	G. max	Cutler 71	PI 548518	EE-0723	United States	Improved line	Purple	Yellow	
	G. max	KAS576-11	IT141801	EE-0357	South Korea	Landrace			
1624	G. max	Franklin	PI 548563	EE-0724	United States	Improved line	Purple	Yellow	

1625	G. max	Gasoy 17	PI 553046	EE-0725	United States	Improved line	White	Yellow	
1626	G. max	Gordon	PI 553047	EE-0726	United States	Improved line	White	Yellow	
1627	G. max	Hutton	PI 548662	EE-0727	United States	Improved line	Purple	Yellow	
1628	G. max	Sherman	PI 548614	EE-0728	United States	Improved line	White	Yellow	
1629	G. max	TN 4-86	PI 518668	EE-0729	United States	Improved line	Purple	Yellow	
1630	G. max	Winchester	PI 548585	EE-0730	United States	Improved line	White	Yellow	
	G. max	KLS 129-1	IT022065	DD-0062	South Korea	Landrace			
1631	G. max	Wright	PI 553042	EE-0731	United States	Improved line	Purple	Yellow	
1632	G. max	PI 475814		EE-0732	China	Not determined	White	Black	Representative
	G. max	PI 475814		DD-0799	China	Not determined			
1633	G. max	Sharkey	PI 515960	EE-0734	United States	Improved line	White	Yellow	
1634	G. max	Leflore	PI 548981	EE-0735	United States	Improved line	Purple	Yellow	
1635	G. max	PI 398440		EE-0737	South Korea	Landrace	White	Yellow	
1636	G. max	PI 398970		EE-0738	South Korea	Landrace	Purple	Yellow	
1637	G. max	PI 407773B		EE-0740	South Korea	Landrace	White	Yellow	
1638	G. max	Baektae	IT175817	EE-0741	South Korea	Landrace	Purple	Yellow	
1639	G. max	Cheongtae	IT175994	EE-0743	South Korea	Landrace	Purple	Green	
1640	G. max	Dubu	IT176015	EE-0744	South Korea	Landrace	Purple	Yellow	
1641	G. max	Geomjeong-1	IT177239	EE-0745	South Korea	Landrace	Purple	Black	
1642	G. max	Geomjeong-2	IT177250	EE-0746	South Korea	Landrace	Purple	Black	
1643	G. max	Geomjeong-2	IT177280	EE-0747	South Korea	Landrace	Purple	Black	
	G. max	YB139-4	IT024969	EE-0165	South Korea	Landrace			
	G. max	YB24-2	IT024869	DD-0171	South Korea	Landrace			
	G. max	KAS310-11	IT115528	DD-0300	South Korea	Landrace			
	G. max	KAS310-12	IT115529	EE-0266	South Korea	Landrace			
	G. max	KAS312-11	IT115534	EE-0268	South Korea	Landrace			
1644	G. max	Geomjeong-2	IT177282	EE-0748	South Korea	Landrace	Purple	Black	
	G. max	Geomjeong-3	IT177401	EE-0765	South Korea	Landrace			
	G. max	Jeonnamjinheungwon NO.24	IT178710	EE-0793	South Korea	Landrace			
1645	G. max	Geomjeong-2	IT177284	EE-0749	South Korea	Landrace	Purple	Black	
1646	G. max	Geomjeong-4	IT177297	EE-0750	South Korea	Landrace	Purple	Black	
1647	G. max	Geomjeong-1	IT177303	EE-0751	South Korea	Landrace	Purple	Black	
1648	G. max	Geomjeong-1	IT177319	EE-0753	South Korea	Landrace	Purple	Black	
1649	G. max	IT177337		EE-0754	South Korea	Landrace	White	Black	
1650	G. max	Geomjeong-5	IT177339	EE-0755	South Korea	Landrace	Purple	Black	
1651	G. max	Geomjeong-2	IT177364	EE-0757	South Korea	Landrace	Purple	Black	
1652	G. max	Geomjeong-4	IT177376	EE-0759	South Korea	Landrace	Purple	Black	
1653	G. max	Geomjeong-1	IT177383	EE-0760	South Korea	Landrace	Purple	Black	
1654	G. max	Geomjeong-4	IT177397	EE-0763	South Korea	Landrace	Purple	Black	
	G. max	kwangwonsujib5-16	IT156214	DD-0646	South Korea	Landrace			
	G. max	KLS87223	IT153823	EE-0472	South Korea	Landrace			
1655	G. max	Geomjeong-2	IT177400	EE-0764	South Korea	Landrace	Purple	Black	
1656	G. max	IT177528		EE-0770	South Korea	Landrace	Purple	Black	
1657	G. max	Geomjeong-5	IT177588	EE-0772	South Korea	Landrace	White	Black	
	G. max	Geomjeong-1	IT177529	EE-0771	South Korea	Landrace			
	G. max	KLS087051	IT153378	DD-0533	South Korea	Landrace			
	G. max	PI 458089		DD-1187	South Korea	Landrace			
	G. max	ORD 8145	PI 407807	EE-1094	South Korea	Landrace			
	G. max	PI 399125		EE-1087	South Korea	Landrace			
	G. max	Geomjeong-3	IT177661	EE-0775	South Korea	Landrace			
	G. max	KAS651-28	PI 424593	EE-0527	South Korea	Landrace			
	G. max	YN30	IT023805	EE-0069	South Korea	Landrace			
	G. max	PI 340019		DD-1240	South Korea	Landrace			
	G. max	ORD 8136	PI 407800	DD-1262	South Korea	Landrace			
	G. max	ORD 8146	IT228504	DD-1264	South Korea	Not determined			
	G. max	ORD 8147	PI 407809	DD-1265	South Korea	Landrace			
1658	G. max	Geomjeong-5	IT177648	EE-0773	South Korea	Landrace	Purple	Black	
	G. max	Babmit	IT216357	EE-0886	South Korea	Landrace			
1659	G. max	Geomjeong-4	IT177714	EE-0776	South Korea	Landrace	White	Black	
1660	G. max	Geomjeong-4	IT177739	EE-0777	South Korea	Landrace	Purple	Black	
1661	G. max	Geomjeong-4	IT177797	EE-0778	South Korea	Landrace	Purple	Black	
	G. max	KLS87332	IT153905	EE-0481	South Korea	Landrace			
	G. max	IT178513		EE-0792	South Korea	Landrace			
1662	G. max	Geomjeong-5	IT177906	EE-0780	South Korea	Landrace	White	Black	
	G. max	Geomjeongol		blackalkong	South Korea				

	G. max	Heksaekyuwoldu	IT102712	EE-0206	South Korea	Landrace			
	G. max	KLS85253	IT143170	DD-0507	South Korea	Landrace			
	G. max	Geomjeongol		CD-1291	South Korea	Improved line			
	G. max	IT186090		DD-0987	South Korea	Landrace			
	G. max	Geomjeongsoknoran	IT195525	DD-1025	South Korea	Landrace			
	G. max	KLS85023	IT142965	DD-0478	South Korea	Landrace			
	G. max	Ggeomeoknamul	IT108705	EE-0236	South Korea	Landrace			
	G. max	KLS85244	IT143161	EE-0426	South Korea	Landrace			
1663	G. max	IT177951		EE-0782	South Korea	Landrace	Purple	Black	
1664	G. max	IT178037		EE-0783	South Korea	Landrace	Purple	Black	
	G. max	IT220682		EE-0931	South Korea	Landrace			
1665	G. max	Geomjeong-2	IT178138	EE-0784	South Korea	Landrace	Purple	Black	
1666	G. max	Geomjeong-1	IT178144	EE-0785	South Korea	Landrace	Purple	Green	
1667	G. max	Geomjeong	IT178164	EE-0787	South Korea	Landrace	Purple	Green	
1668	G. max	Geomjeong-4	IT178175	EE-0788	South Korea	Landrace	Purple	Green	
1669	G. max	Geomjeong	IT178207	EE-0790	South Korea	Landrace	Purple	Green	
1670	G. max	KLS 77004-2	IT179936	EE-0794	South Korea	Landrace	Purple	Black	
	G. max	KAS600-8	IT154388	EE-0500	South Korea	Landrace			
1671	G. max	SLSB-B-47	IT179946	EE-0795	South Korea	Landrace	Purple	Black	
	G. max	SLSB-B-21	IT134472	DD-0387	South Korea	Landrace			
	G. max	KAS651-43	IT115863	EE-0298	South Korea	Landrace			
1672	G. max	IT180325		EE-0796	South Korea	Landrace	White	Yellow	
1673	G. max	IT180331		EE-0798	South Korea	Landrace	Purple	Brown	
	G. max	IT180330		EE-0797	South Korea	Landrace			
	G. max	PI 458193		EE-1109	South Korea	Landrace			
1674	G. max	IT180342		EE-0800	South Korea	Landrace	Purple	Black	
1675	G. max	IT180365		EE-0802	South Korea	Landrace	Purple	Green	
	G. max	YB308-4	IT025138	DD-0194	South Korea	Landrace			
	G. max	SLSB310-1	IT134410	DD-0376	South Korea	Landrace			
1676	G. max	Meju	IT180473	EE-0804	South Korea	Landrace	White	Yellow	
1677	G. max	Babmit	IT180845	EE-0808	South Korea	Landrace	Purple	Dark brown	
1678	G. max	Meju	IT180849	EE-0811	South Korea	Landrace	White	Yellow	
1679	G. max	KAS151-22	IT181368	EE-0814	South Korea	Landrace	Purple	Green	
1680	G. max	KAS183-2	IT181404	EE-0817	South Korea	Landrace	Purple	Yellow	
1681	G. max	KAS232-2	PI 398487	EE-0818	South Korea	Landrace	Purple	Brown	
	G. max	KAS636-2	PI 408111	EE-0511	South Korea	Landrace			
	G. max	KLS351-1	IT022480	EE-0019	South Korea	Landrace			
	G. max	KLS733-1	PI 399004	DD-0039	South Korea	Landrace			
	G. max	YN125-4	IT024546	EE-0128	South Korea	Landrace			
1682	G. max	KAS233-1	PI 423776	EE-0819	South Korea	Landrace	White	Yellow	
1683	G. max	KAS310-6	PI 424307	EE-0821	South Korea	Landrace	Purple	Brown	
1684	G. max	KAS622-6	IT181687	EE-0823	South Korea	Landrace	Purple	Yellow	
1685	G. max	KAS640-20	PI 424575	EE-0824	South Korea	Landrace	Purple	Green	
1686	G. max	Meju	IT181893	EE-0826	South Korea	Landrace	Purple	Yellow	
1687	G. max	NunGeomjeongi	IT181938	EE-0827	South Korea	Landrace	Purple	Yellow	
	G. max	Gangrim	IT021781	CD-1379	South Korea	Improved line			
	G. max	IT103619		DD-0229	South Korea	Landrace			
1688	G. max	Kangwon68	IT186080	EE-0834	South Korea	Landrace	Purple	Black	
1689	G. max	IT186196		EE-0840	South Korea	Landrace	Purple	Black	
	G. max	Seoribam	IT224176	DD-1137	South Korea	Landrace			
	G. max	Geomjeong-5	IT177653	EE-0774	South Korea	Landrace			
1690	G. max	Daechubam	IT186235	EE-0841	South Korea	Landrace	Purple	Brown	
1691	G. max	Jeonbuk sujib	IT189289	EE-0843	South Korea	Landrace	White	Yellow	
1692	G. max	Jeonbuk sujib	IT189294	EE-0844	South Korea	Landrace	Purple	Green	
1693	G. max	Jeonnam sujib	IT189312	EE-0846	South Korea	Landrace	Purple	Yellow	
1694	G. max	Jilgeum	IT191166	EE-0847	South Korea	Landrace	Purple	Green	
1695	G. max	Geomjeong	IT194559	EE-0851	South Korea	Landrace	Purple	Black	
1696	G. max	IT194564		EE-0852	South Korea	Landrace	Purple	Brown	
1697	G. max	Meju	IT195340	EE-0853	South Korea	Landrace	White	Yellow	
1698	G. max	Busan sujib	IT195623	EE-0856	South Korea	Landrace	Purple	Yellow	
1699	G. max	Hamyang sujib	IT195625	EE-0858	South Korea	Landrace	Purple	Yellow	
	G. max	Manri		CD-1185	South Korea	Improved line			
1700	G. max	Jwinuni	IT196908	EE-0864	South Korea	Landrace	White	Black	
1701	G. max	IT196925		EE-0865	North Korea	Landrace	Purple	Yellow	Representative
1702	G. max	IT196926		EE-0866	North Korea	Landrace	Purple	Yellow	Representative
1703	G. max	Jefferson	PI 548351	EE-0867	North Korea	Not determined	White	Yellow	

	G. max	KAS629-9	IT142007	EE-0378	South Korea	Landrace			
	G. max	KLS87242	IT153838	DD-0558	South Korea	Landrace			
	G. max	KAS310-7	PI 424308	DD-0962	South Korea	Landrace			
	G. max	KAS354-3	PI 458125	DD-0965	South Korea	Landrace			
	G. max	KAS646-8	PI 408334	DD-0614	South Korea	Landrace			
	G. max	KAS233-8	PI 458055	DD-0405	South Korea	Landrace			
	G. max	KAS630-28	IT154452	EE-0504	South Korea	Landrace			
	G. max	KAS624-18	PI 408299	EE-0377	South Korea	Landrace			
	G. max	KAS602-13	IT141866	EE-0364	South Korea	Landrace			
	G. max	KLS77125-2	IT025354	EE-0190	South Korea	Landrace			
	G. max	KAS571-10	PI 424443	EE-0354	South Korea	Landrace			
	G. max	SLSJ171-1	IT134242	EE-0315	South Korea	Landrace			
1704	G. max	Kin-du	PI 157440	EE-0871	South Korea	Landrace	Purple	Yellow	
1705	G. max	Milyang46	IT203564	EE-0872	South Korea	Improved line	White	Yellow	
1706	G. max	Yak	IT203616	EE-0875	South Korea	Landrace	Purple	Black	Representative
1707	G. max	Junjeori	IT208524	EE-0876	South Korea	Landrace	Purple	Yellow	
1708	G. max	Pyeongyang	IT209069	EE-0878	South Korea	Landrace	Purple	Yellow	
1709	G. max	Gyeongnam Sacheon-1998-16	IT209906	EE-0881	South Korea	Landrace	Purple	Yellow	
1710	G. max	Palwolbae	IT212802	EE-0883	South Korea	Landrace	Purple	Yellow	
1711	G. max	Jeonnam wando-2000-53	IT216868	EE-0887	South Korea	Landrace	Purple	Yellow	
1712	G. max	Chungnam Yeongi-1999-4	IT219471	EE-0891	South Korea	Landrace	White	Black	
1713	G. max	Jilgeum	IT219511	EE-0895	South Korea	Landrace	Purple	Black	
1714	G. max	KAS 134-7	IT219604	EE-0896	South Korea	Landrace	White	Green yellow	
1715	G. max	KAS544-2	PI 407996	EE-0897	South Korea	Landrace	White	Green	
1716	G. max	KAS544-13	PI 424431	EE-0898	South Korea	Landrace	Purple	Black	
1717	G. max	KAS547-11	IT219627	EE-0899	South Korea	Landrace	Purple	Green	
1718	G. max	KAS571-26	IT219637	EE-0901	South Korea	Landrace	Purple	Brown	
1719	G. max	CS00704	IT219705	EE-0905	South Korea	Landrace	Purple	Green	
1720	G. max	KAS302-19	IT219770	EE-0909	South Korea	Landrace	Purple	Yellow	
1721	G. max	KAS361-2	IT219783	EE-0911	South Korea	Landrace	Purple	Yellow	
1722	G. max	KAS370-5	PI 398543	EE-0914	South Korea	Landrace	Purple	Brown	
	G. max	YN208	IT023944	DD-0090	South Korea	Landrace			
	G. max	SLSN233-1	IT134312	DD-0365	South Korea	Landrace			
	G. max	KAS633-6	IT154499	EE-0507	South Korea	Landrace			
	G. max	KLS77195-2	IT025481	EE-0198	South Korea	Landrace			
	G. max	YN233-4	IT023978	EE-0083	South Korea	Landrace			
	G. max	SLSN233-2	IT134313	EE-0321	South Korea	Landrace			
1723	G. max	KAS510-18	IT219797	EE-0915	South Korea	Landrace	Purple	Green	
1724	G. max	KAS513-14	IT219799	EE-0916	South Korea	Landrace	Purple	Brown	
1725	G. max	KAS523-38	IT219802	EE-0917	South Korea	Landrace	Purple	Black	
	G. max	PI 458164		EE-1107	South Korea	Landrace			
1726	G. max	KAS540-2	IT219804	EE-0918	South Korea	Landrace	Purple	Yellow	
1727	G. max	KAS540-31	IT219809	EE-0919	South Korea	Landrace	Purple	Green	
1728	G. max	KAS679-24	IT219818	EE-0920	South Korea	Landrace	Purple	Yellow	
1729	G. max	KAS 131-7	IT220545	EE-0921	South Korea	Landrace	White	Yellow	
1730	G. max	KAS 150-6	PI 398241	EE-0922	South Korea	Landrace	Purple	Green	
1731	G. max	KAS 150-27	PI 424241	EE-0923	South Korea	Landrace	Purple	Green	
1732	G. max	KAS 201-6-2	PI 398413	EE-0924	South Korea	Landrace	Purple	Black	
1733	G. max	KAS 201-8-2	IT220566	EE-0925	South Korea	Landrace	Purple	Yellow	
1734	G. max	KAS 205-9	PI 398437	EE-0926	South Korea	Landrace	Purple	Black	
1735	G. max	KAS 205-17	PI 423754	EE-0927	South Korea	Landrace	Purple	Black	
1736	G. max	KAS 205-25	PI 424261	EE-0928	South Korea	Landrace	Purple	Yellow	
	G. max	YB195-1	IT024425	EE-0112	South Korea	Landrace			
1737	G. max	KAS 210-9-1	IT220574	EE-0929	South Korea	Landrace	Purple	Green yellow	
	G. max	KAS362-10	IT115581	DD-0306	South Korea	Landrace			
1738	G. max	KAS 210-16	PI 458037	EE-0930	South Korea	Landrace	White	Yellow	
1739	G. max	Sangjupureun	IT220685	EE-0932	South Korea	Landrace	Purple	Green	
1740	G. max	CS 00829	IT221942	EE-0934	South Korea	Landrace	Purple	Black	
1741	G. max	Saline	PI 578057	EE-0938	United States	Improved line	White	Yellow	
1742	G. max	Jeonnam hwasun-1997-37	IT224169	EE-0941	South Korea	Landrace	Purple	Yellow	
1743	G. max	Incheon ganghwa-1997-11	IT224181	EE-0945	South Korea	Landrace	Purple	Brown	
1744	G. max	Juinuni	IT224191	EE-0946	South Korea	Landrace	White	Brown	
1745	G. max	GL2689	IT224194	EE-0947	North Korea	Not determined	Purple	Black	

1746	G. max	Jang	IT224216	EE-0949	South Korea	Landrace	White	Yellow	
	G. max	kwangwon Pyungchang-1999-17	IT224272	EE-0950	South Korea	Landrace			
1747	G. max	Jeonnam goheung-1999-2	IT224403	EE-0951	South Korea	Landrace	Purple	Yellow	
1748	G. max	Gyeongnam tongyeong-2000-64	IT224431	EE-0953	South Korea	Landrace	White	Yellow	
1749	G. max	Savoy	PI 597381	EE-0955	United States	Improved line	Purple	Yellow	
1750	G. max	SAKO 25-26	IT224498	EE-0956	Japan	Improved line	White	Yellow	
1751	G. max	Daechu	IT224516	EE-0957	South Korea	Landrace	Purple	Brown	
1752	G. max	Kangwon cheolwon-2001-62	IT224519	EE-0960	South Korea	Landrace	Purple	Black	
1753	G. max	Kangwon samcheok-2001-47	IT224536	EE-0963	South Korea	Landrace	Purple	Yellow	
	G. max	Neuj	IT224594	EE-0965	South Korea	Landrace			
	G. max	Meju	IT224922	EE-0972	South Korea	Landrace			
	G. max	IT196433		EE-0860	South Korea	Landrace			
	G. max	Bam	IT209178	EE-0879	South Korea	Landrace			
	G. max	IT186103		DD-0989	South Korea	Landrace			
	G. max	Heuin	IT186108	DD-0990	South Korea	Landrace			
	G. max	IT189661		DD-1000	South Korea	Landrace			
	G. max	IT196886		DD-1034	South Korea	Landrace			
	G. max	kwangwon양양-2010-72	IT224541	DD-1149	South Korea	Landrace			
	G. max	Meju	IT224917	DD-1158	South Korea	Landrace			
1754	G. max	Jang	IT224915	EE-0970	South Korea	Landrace	Purple	Yellow	
1755	G. max	25	IT224920	EE-0971	South Korea	Landrace	Purple	Yellow	
1756	G. max	Baec-moc-sa-ryu	PI 157398	EE-0975	South Korea	Landrace	Purple	Yellow	
	G. max	Jeokgak	IT230741	EE-1180	South Korea	Landrace			
1757	G. max	Chae-rae-chong	IT226749	EE-0977	South Korea	Landrace	Purple	Yellow	
1758	G. max	Chung-buc-huwang No. 1	PI 157414	EE-0978	South Korea	Landrace	Purple	Yellow	
1759	G. max	PI 339984		EE-0980	South Korea	Landrace	Purple	Yellow	
	G. max	PI 398873		EE-0984	South Korea	Landrace			
1760	G. max	PI 340002		EE-0981	South Korea	Landrace	Purple	Yellow	
1761	G. max	Hikmok sorip	PI 372415 A	EE-0982	South Korea	Landrace	White	Yellow	Representative
1762	G. max	PI 398493		EE-0983	South Korea	Landrace	Purple	Yellow	
1763	G. max	PI 398912		EE-0986	South Korea	Landrace	White	Yellow	
1764	G. max	PI 399079		EE-0989	South Korea	Landrace	Purple	Yellow	
1765	G. max	Kang lim	PI 406709	EE-0991	South Korea	Improved line	White	Yellow	
1766	G. max	ORD 8188	IT226795	EE-0993	South Korea	Landrace	Purple	Yellow	
1767	G. max	PI 407810		EE-0994	South Korea	Landrace	Purple	Black	
	G. max	Geomjeong-4	IT177413	DD-0884	South Korea	Landrace			
1768	G. max	PI 407848		EE-0998	South Korea	Landrace	Purple	Yellow	
1769	G. max	KAS 662-6	PI 424175	EE-1003	South Korea	Landrace	Purple	Yellow	
1770	G. max	PI 427136		EE-1005	South Korea	Landrace	White	Yellow	Representative
1771	G. max	VIR 2987	IT226846	EE-1009	North Korea	Not determined	Purple	Yellow	
1772	G. max	DV-2370	PI 438311	EE-1010	North Korea	Not determined	White	Yellow	
1773	G. max	PI 458030		EE-1011	South Korea	Landrace	Purple	Black	
1774	G. max	PI 458035		EE-1012	South Korea	Landrace	Purple	Black	
1775	G. max	PI 458051 B		EE-1014	South Korea	Landrace	Purple	Yellow	
1776	G. max	PI 458074 B		EE-1015	South Korea	Landrace	Purple	Yellow	
1777	G. max	PI 458090 A		EE-1017	South Korea	Landrace	Purple	Yellow	
1778	G. max	PI 458103		EE-1020	South Korea	Landrace	Purple	Yellow	
1779	G. max	Kangwon yangyang-2001-15	IT227862	EE-1021	South Korea	Landrace	White	Yellow	
	G. max	Hanagari	IT104537	EE-0226	South Korea	Landrace			
1780	G. max	PI 458110		EE-1023	South Korea	Landrace	Purple	Yellow	
1781	G. max	PI 458111		EE-1024	South Korea	Landrace	White	Yellow	
1782	G. max	PI 458120		EE-1025	South Korea	Landrace	Purple	Yellow	
1783	G. max	PI 458138		EE-1027	South Korea	Landrace	Purple	Yellow	
1784	G. max	PI 458144		EE-1028	South Korea	Landrace	Purple	Yellow	
1785	G. max	PI 458152		EE-1029	South Korea	Landrace	White	Yellow	
1786	G. max	PI 458156		EE-1030	South Korea	Landrace	Purple	Black	
	G. max	YB355	IT025188	EE-0180	South Korea	Landrace			
	G. max	PI 458112 A		DD-1191	South Korea	Landrace			
1787	G. max	PI 458181		EE-1033	South Korea	Landrace	Purple	Brown	
	G. max	Bam	IT104535	EE-0225	South Korea	Landrace			

	G. max	KAS210-35	IT115412	EE-0256	South Korea	Landrace			
	G. max	PI 458134		EE-1026	South Korea	Landrace			
	G. max	KAS239-6	PI 424288	EE-0820	South Korea	Landrace			
	G. max	KAS363-8	PI 458151	EE-0339	South Korea	Landrace			
	G. max	IT186054		DD-0979	South Korea	Landrace			
	G. max	PI 458131		DD-1195	South Korea	Landrace			
	G. max	KAS645-10	IT154688	DD-0613	South Korea	Landrace			
	G. max	KAS637-16	IT154557	EE-0515	South Korea	Landrace			
1788	G. max	PI 458184		EE-1034	South Korea	Landrace	Purple	Black	
1789	G. max	PI 458189 B		EE-1035	South Korea	Landrace	White	Yellow	
1790	G. max	PI 458209		EE-1037	South Korea	Landrace	White	Black	
1791	G. max	PI 458232		EE-1040	South Korea	Landrace	White	Green yellow	
	G. max	Babmit	IT178645	DD-0932	South Korea	Landrace			
	G. max	PI 458249		DD-1206	South Korea	Landrace			
	G. max	Meju	IT180466	EE-0803	South Korea	Landrace			
1792	G. max	PI 458248		EE-1043	South Korea	Landrace	Purple	Yellow	
	G. max	KAS577-18	PI 458255	EE-0358	South Korea	Landrace			
1793	G. max	PI 458259		EE-1044	South Korea	Landrace	White	Yellow	
1794	G. max	PI 458277		EE-1045	South Korea	Landrace	Purple	Yellow	
	G. max	PI 458266		DD-1208	South Korea	Landrace			
	G. max	KLS087072	IT153385	EE-0446	South Korea	Landrace			
	G. max	KLS87057	IT153681	DD-0542	South Korea	Landrace			
	G. max	PI 458234		DD-1204	South Korea	Landrace			
1795	G. max	PI 458287		EE-1046	South Korea	Landrace	Purple	Green	
1796	G. max	PI 458298		EE-1049	South Korea	Landrace	White	Green	
1797	G. max	PI 458303		EE-1050	South Korea	Landrace	Purple	Green	
1798	G. max	VIR 2977	IT228332	EE-1052	North Korea	Not determined	Purple	Yellow	Representative
1799	G. max	VIR 2980	IT228335	EE-1053	North Korea	Not determined	Purple	Yellow	
1800	G. max	Back Tac	PI 567273 A	EE-1054	South Korea	Landrace	Purple	Yellow	
1801	G. max	Sinpaldalkong	PI 597482	EE-1055	South Korea	Improved line	White	Yellow	
1802	G. max	GL 2684 /95	PI 603171	EE-1056	North Korea	Not determined	Purple	Brown	
1803	G. max	GL 2688 /96	PI 603175	EE-1057	North Korea	Not determined	Purple	Brown	
1804	G. max	Cin	PI 603910 A	EE-1058	South Korea	Landrace	White	Black	
1805	G. max	PI 603913 A		EE-1059	South Korea	Landrace	Purple	Yellow	
1806	G. max	PI 603913 D		EE-1060	North Korea	Landrace	White	Green yellow	Representative
1807	G. max	Gumgang	PI 612614	EE-1061	South Korea	Landrace	Purple	Yellow	
1808	G. max	Giant	PI 157422	EE-1063	South Korea	Landrace	Purple	Brown	
	G. max	Southern Prolific	PI 157473	EE-1066	South Korea	Landrace			
1809	G. max	I chu tau chow	PI 157430	EE-1064	South Korea	Landrace	Purple	Yellow	
1810	G. max	Ic san	IT228391	EE-1065	South Korea	Landrace	White	Yellow	
1811	G. max	Well-man	PI 157487 A	EE-1067	South Korea	Landrace	White	Yellow	
1812	G. max	Yu tae	IT228397	EE-1068	South Korea	Landrace	Purple	Yellow	
1813	G. max	PI 339982		EE-1069	South Korea	Landrace	Purple	Yellow	
	G. max	PI85619		EE-0644	South Korea	Landrace			
	G. max	Sekishoku Zunonikon	PI 87561	EE-1250	South Korea	Landrace			
1814	G. max	KLS 808-1	PI 399025	EE-1074	South Korea	Landrace	Purple	Green	
1815	G. max	PI 399062		EE-1076	South Korea	Landrace	Purple	Yellow	
1816	G. max	PI 399078		EE-1077	South Korea	Landrace	Purple	Yellow	
1817	G. max	PI 399089		EE-1078	South Korea	Landrace	Purple	Green brown	
1818	G. max	PI 399097		EE-1079	South Korea	Landrace	Purple	Yellow	
1819	G. max	PI 399108		EE-1081	South Korea	Landrace	White	Yellow	
1820	G. max	PI 399113		EE-1082	South Korea	Landrace	White	Yellow	
	G. max	YJ227-3	IT024750	EE-0152	South Korea	Landrace			
	G. max	PI 340032		EE-1072	South Korea	Landrace			
	G. max	YN76-3	IT024516	EE-0124	South Korea	Landrace			
	G. max	SLSJ311-3	IT134260	EE-0318	South Korea	Landrace			
	G. max	KAS320-5	IT181547	EE-0822	South Korea	Landrace			
	G. max	KAS571-2	IT219636	EE-0900	South Korea	Landrace			
	G. max	KAS220-16	IT141491	EE-0334	South Korea	Landrace			
	G. max	KAS370-3	IT219790	EE-0913	South Korea	Landrace			
	G. max	KAS609-11	IT219645	EE-0902	South Korea	Landrace			
	G. max	KAS354-19	IT219776	EE-0910	South Korea	Landrace			
	G. max	KAS360-11	IT219778	DD-1101	South Korea	Landrace			
	G. max	PI 339999		DD-1237	South Korea	Landrace			
	G. max	PI 340028		DD-1242	South Korea	Landrace			
	G. max	PI 458096		DD-1280	South Korea	Landrace			

	G. max	PI 458293		DD-1293	South Korea	Landrace			
	G. max	KAS625-6	IT154443	DD-0587	South Korea	Landrace			
	G. max	KAS643-13	IT154654	DD-0609	South Korea	Landrace			
	G. max	KLS 723-1-1	IT230773	DD-1347	South Korea	Landrace			
	G. max	KLS126-3	IT022427	DD-0015	South Korea	Landrace			
	G. max	KLS208	PI 398894	DD-0019	South Korea	Landrace			
	G. max	YJ11	IT023983	DD-0095	South Korea	Landrace			
	G. max	YJ66	IT024014	DD-0099	South Korea	Landrace			
	G. max	YJ105-2	IT024025	DD-0101	South Korea	Landrace			
	G. max	YJ230-2	IT024096	DD-0110	South Korea	Landrace			
	G. max	YN105-3	IT024428	DD-0126	South Korea	Landrace			
	G. max	YJ93-2	IT024647	DD-0146	South Korea	Landrace			
	G. max	KLS77026	IT025231	DD-0201	South Korea	Landrace			
	G. max	KAS302-16	IT115522	DD-0299	South Korea	Landrace			
	G. max	KAS640-42	IT115820	DD-0334	South Korea	Landrace			
	G. max	KAS354-5	PI 458127	DD-0412	South Korea	Landrace			
	G. max	KAS233-18	IT115447	EE-0258	South Korea	Landrace			
	G. max	KAS302-12	IT115518	EE-0265	South Korea	Landrace			
	G. max	PI 458100		EE-1104	South Korea	Landrace			
	G. max	YJ123-4	IT024041	EE-0088	South Korea	Landrace			
	G. max	KLS627-2	IT022562	EE-0033	South Korea	Landrace			
	G. max	KAS243-3	PI 458099	EE-0260	South Korea	Landrace			
	G. max	KLS824-1	IT022677	EE-0049	South Korea	Landrace			
	G. max	KAS 680-4	IT226823	EE-1000	South Korea	Landrace			
	G. max	KLS616-3	IT022547	EE-0029	South Korea	Landrace			
	G. max	PI 458161		EE-1106	South Korea	Landrace			
	G. max	KLS732-2	PI 399003	EE-0039	South Korea	Landrace			
	G. max	KLS77210-2	IT025507	EE-0200	South Korea	Landrace			
	G. max	PI 458099		EE-1103	South Korea	Landrace			
	G. max	PI 340008		EE-1070	South Korea	Landrace			
	G. max	YN155-2	IT023889	EE-0077	South Korea	Landrace			
	G. max	KAS200-12	IT141466	EE-0254	South Korea	Landrace			
	G. max	YJ250-2	IT024786	EE-0157	South Korea	Landrace			
1821	G. max	PI 399115		EE-1084	South Korea	Landrace	White	Yellow	
1822	G. max	PI 399126		EE-1088	South Korea	Landrace	Purple	Black	
1823	G. max	ORD 8109	PI 407785	EE-1089	South Korea	Landrace	Purple	Black	
1824	G. max	PI 407801		EE-1091	South Korea	Landrace	Purple	Green yellow	
1825	G. max	ORD 8139	PI 407803	EE-1092	South Korea	Landrace	Purple	Green yellow	
1826	G. max	ORD 8197	PI 407846	EE-1098	South Korea	Landrace	Purple	Yellow	
1827	G. max	PI 458150 A		EE-1105	South Korea	Landrace	Purple	Yellow	
1828	G. max	PI 458211		EE-1110	South Korea	Landrace	White	Brown	Representative
1829	G. max	PI 458236 B		EE-1112	South Korea	Landrace	Purple	Green	
1830	G. max	PI 458260		EE-1113	South Korea	Landrace	Purple	Green	
1831	G. max	PI 458291		EE-1115	South Korea	Landrace	White	Yellow	
1832	G. max	Baksungtae	PI 603907	EE-1119	South Korea	Landrace	Purple	Yellow	
1833	G. max	Sogcheong	IT228629	EE-1120	South Korea	Landrace	Purple	Black	
1834	G. max	CS 01922	IT228787	EE-1121	South Korea	Landrace	Purple	Black	
1835	G. max	CS 01935	IT228789	EE-1122	South Korea	Landrace	Purple	Black	
1836	G. max	CS 01964	IT229073	EE-1123	South Korea	Landrace	White	Yellow	
	G. max	CS 01915	IT228786	DD-1297	South Korea	Landrace			
1837	G. max	CS 02004	IT229080	EE-1125	South Korea	Landrace	Purple	Black	
1838	G. max	CS 02005	IT229081	EE-1126	South Korea	Landrace	Purple	Black	
1839	G. max	CS 02006	IT229082	EE-1127	South Korea	Landrace	White	Black	
1840	G. max	Nungeomjaeongi	IT229404	EE-1129	South Korea	Landrace	Purple	Green yellow	
1841	G. max	KLS 87179	IT229455	EE-1130	South Korea	Landrace	Purple	Yellow	
1842	G. max	IT229463		EE-1131	South Korea	Landrace	Purple	Yellow	
	G. max	IT180846		EE-0809	South Korea	Landrace			
1843	G. max	KAS 130-7	IT229954	EE-1132	South Korea	Landrace	Purple	Black	
1844	G. max	KAS 131-4	IT229955	EE-1133	South Korea	Landrace	Purple	Yellow	
1845	G. max	PI 340020		EE-1136	South Korea	Landrace	White	Yellow	
1846	G. max	PI 340049		EE-1137	South Korea	Landrace	Purple	Yellow	
1847	G. max	PI 399028		EE-1138	South Korea	Landrace	Purple	Yellow	
1848	G. max	PI 399070		EE-1139	South Korea	Landrace	Purple	Black	
	G. max	Geomjeong	IT111024	DD-0265	South Korea	Landrace			
1849	G. max	PI 399112		EE-1140	South Korea	Landrace	Purple	Brown	
1850	G. max	PI 407783		EE-1141	South Korea	Landrace	Purple	Yellow	
1851	G. max	KAS 636-10	PI 424557	EE-1143	South Korea	Landrace	Purple	Yellow	

	G. max	Lee Yiong Kong	IT230687	DD-1332	South Korea	Landrace			
	G. max	Yuku7	IT023340	DD-0060	Japan	Improved line			
1852	G. max	PI 603176 A		EE-1150	North Korea	Not determined	Purple	Black	
1853	G. max	Banchongdoo	PI 339864 A	EE-1151	South Korea	Landrace	White	Yellow	
1854	G. max	PI 458045 B		EE-1154	South Korea	Landrace	Purple	Green	
1855	G. max	Anchuumu Ripaamukon	PI 416785	EE-1156	Japan	Landrace	Purple	Black	
1856	G. max	Heijou	IT230667	EE-1157	Japan	Landrace	Purple	Yellow	
1857	G. max	Rikankan	PI 417247	EE-1158	Japan	Landrace	Purple	Yellow	
	G. max	Oikibal	IT230783	EE-1197	South Korea	Landrace			
1858	G. max	Ryuugan	PI 417259	EE-1159	Japan	Landrace	Purple	Yellow	
1859	G. max	Uronkon	PI 417440	EE-1162	Japan	Landrace	Purple	Green yellow	
1860	G. max	Chung Tai	IT230685	EE-1163	South Korea	Landrace	Purple	Green	
	G. max	Seita	PI 88809	EE-1170	South Korea	Landrace			
1861	G. max	Chuuhoku 2	IT230706	EE-1167	South Korea	Landrace	Purple	Yellow	
1862	G. max	Daidouta	IT230707	EE-1168	South Korea	Landrace	Purple	Yellow	
1863	G. max	Danchun	IT230719	EE-1173	South Korea	Landrace	Purple	Yellow	
1864	G. max	Hankjongjaeji	IT230726	EE-1175	South Korea	Landrace	Purple	Yellow	
	G. max	KLS 902-1	PI 399042	DD-1246	South Korea	Landrace			
	G. max	Bukjaejuhankyeong	IT230717	EE-1172	South Korea	Landrace			
1865	G. max	Anbyontae	IT230728	EE-1176	South Korea	Landrace	Purple	Yellow	
1866	G. max	Jaelae 2	IT230739	EE-1179	South Korea	Landrace	Purple	Green	
	G. max	KLS614-1	PI 398951	DD-0028	South Korea	Landrace			
1867	G. max	Jeonjnjaelae	IT230742	EE-1181	South Korea	Landrace	Purple	Yellow	
1868	G. max	Keumhwajaelae	IT230748	EE-1184	South Korea	Landrace	Purple	Yellow	
1869	G. max	Hoisekdaelip	IT230752	EE-1185	South Korea	Landrace	White	Yellow	Representative
	G. max	KLS113	IT022406	DD-1148	South Korea	Landrace			
1870	G. max	Sangdu	IT230758	EE-1186	South Korea	Landrace	Purple	Yellow	
1871	G. max	Shunbijebi Kong	IT230759	EE-1187	South Korea	Landrace	White	Green	
	G. max	Sunbijabikong	PI 339867	EE-1152	South Korea	Landrace			
1872	G. max	Suweon 22	IT230762	EE-1190	South Korea	Improved line	Purple	Yellow	
1873	G. max	Ulsan	IT230768	EE-1192	South Korea	Landrace	White	Yellow	
1874	G. max	Jaerae #20	IT230780	EE-1196	South Korea	Landrace	Purple	Yellow	
1875	G. max	Sugae #43(A)	IT229363	EE-1198	South Korea	Improved line	White	Yellow	
1876	G. max	Jaerae #14	IT230787	EE-1199	South Korea	Landrace	Purple	Brown	
1877	G. max	Jaerae #1	IT230789	EE-1200	South Korea	Landrace	Purple	Yellow	
1878	G. max	Jaesa #2	IT230794	EE-1201	South Korea	Landrace	Purple	Yellow	
1879	G. max	Potae	IT230801	EE-1204	South Korea	Landrace	Purple	Yellow	
1880	G. max	Duidu	IT230809	EE-1205	South Korea	Landrace	Purple	Yellow	
1881	G. max	Suwongaetong #3	IT230814	EE-1207	South Korea	Improved line	White	Yellow	
1882	G. max	KLS 150-2-2	IT230836	EE-1212	South Korea	Landrace	Purple	Brown	
1883	G. max	Buakdari	IT231341	EE-1215	South Korea	Landrace	Purple	Yellow	
1884	G. max	Buchae	IT231342	EE-1216	South Korea	Landrace	White	Yellow	
	G. max	Buchae	IT022163	EE-0008	South Korea	Landrace			
	G. max	Hwacho	IT186237	EE-0842	South Korea	Landrace			
1885	G. max	Nunkkamegi	IT231348	EE-1218	South Korea	Landrace	White	Yellow	
1886	G. max	Milyang203	IT236156	EE-1226	South Korea	Improved line	White	Yellow	
1887	G. max	Suwon258	IT236159	EE-1227	South Korea	Improved line	White	Yellow	
1888	G. max	KAS 200-36	PI 424243	EE-1232	South Korea	Landrace	Purple	Green	
1889	G. max	CS 01968	IT237760	EE-1233	South Korea	Landrace	Purple	Brown	
1890	G. max	KAERI 590-6	PI 408342	EE-1235	South Korea	Not determined	White	Brown	
1891	G. max	KLG 15793	IT238182	EE-1237	China	Not determined	Purple	Yellow	
1892	G. max	You huang dou	PI 567354	EE-1238	China	Not determined	White	Brown	
1893	G. max	Xiao bai dou	IT238208	EE-1239	China	Not determined	White	Yellow	
1894	G. max	Gong xian huang dou	PI 567618 A	EE-1240	China	Not determined	Purple	Yellow	
1895	G. max	Pei xian xiao bai jian ke	PI 567759	EE-1241	China	Not determined	White	Yellow	
1896	G. max	Fen dou 16	PI 574476 A	EE-1244	China	Not determined	Purple	Yellow	Representative
1897	G. max	KLG 15980	IT238308	EE-1247	South Korea	Landrace	Purple	Yellow	
1898	G. max	Ryumuo	PI 88813	EE-1248	North Korea	Landrace	White	Yellow	
1899	G. max	KLG 16001	IT238318	EE-1251	North Korea	Not determined	Purple	Yellow	
1900	G. max	Wu tong shu huang dou	IT238326	EE-1252	China	Not determined	Purple	Yellow	
1901	G. max	Qi si wa	IT238327	EE-1253	China	Not determined	White	Yellow	
1902	G. max	Jilgeum	IT238357	EE-1254	South Korea	Landrace	Purple	Yellow	
1903	G. max	Napjoraegi	IT238358	EE-1255	South Korea	Landrace	Purple	Brown	Representative

	G. max	Nabjirgi	IT111141, Kongnamul	EE-0243	South Korea	Landrace			
1904	G. max	Jeonnam jangheung-1997-61	IT239893	EE-1256	South Korea	Landrace	Purple	Black	
1905	G. max	Jwineori	IT239896	EE-1257	South Korea	Landrace	White	Black	
1906	G. max	PI 273483 A		EE-1258	South Korea	Landrace	White	Yellow	
1907	G. max	KAS 604-1-1	IT230186	EE-1261	South Korea	Landrace	Purple	Yellow	
1908	G. max	GL 2687 A	IT242452	EE-1265	North Korea	Not determined	Purple	Black	
1909	G. max	Hanagari	IT242601	EE-1266	South Korea	Landrace	White	Yellow	
1910	G. max	Baektae	IT242602	EE-1267	South Korea	Landrace	White	Yellow	
1911	G. max	Meju	IT242604	EE-1269	South Korea	Landrace	Purple	Yellow	
	G. max	IT186167		DD-0996	South Korea	Landrace			
	G. max	KLS87172	IT153779	DD-0554	South Korea	Landrace			
	G. max	Baektae	IT178367	EE-0791	South Korea	Landrace			
1912	G. max	Ol	IT242609	EE-1271	South Korea	Landrace	White	Yellow	
1913	G. max	Meoru	IT242613	EE-1272	South Korea	Landrace	Purple	Green yellow	
	G. max	SLS90-71	IT167709	DD-0807	South Korea	Landrace			
	G. max	Geomjeong-3	IT177264	DD-0863	South Korea	Landrace			
	G. max	IT186020		DD-0976	South Korea	Landrace			
	G. max	Pureun	IT195328	DD-1021	South Korea	Landrace			
	G. max	Gyungnamgeochang sujib	IT224402	DD-1142	South Korea	Landrace			
	G. max	KAS 150-5	PI 398240	DD-1313	South Korea	Landrace			
	G. max	Geomjeong-2	IT178173	DD-0921	South Korea	Landrace			
	G. max	(3) Kyongi	PI 399048	DD-1417	South Korea	Landrace			
	G. max	KLS87259	IT153853	DD-0560	South Korea	Landrace			
	G. max	KAS638-9	IT154568	DD-0602	South Korea	Landrace			
	G. max	KLS77090	IT025297	DD-0207	South Korea	Landrace			
	G. max	KAS638-15	IT154574	EE-0516	South Korea	Landrace			
	G. max	kwangwonsujib3-8	IT156168	EE-0550	South Korea	Landrace			
	G. max	Cheongtae	IT158247	EE-0575	South Korea	Landrace			
1914	G. max	Gangil		EE-1279	South Korea	Improved line	Purple	Yellow	
	G. max	Gangil		CD-1214	South Korea	Improved line			
	G. max	Heassal		CD-1219	South Korea	Improved line			sampling mistake
1915	G. max	IlpumGeomjeong2		EE-1281	South Korea	Improved line	Purple	Black	
1916	G. max	Hwangkeumol		EE-1282	South Korea	Improved line	White	Yellow	
1917	G. max	Galmi		Galmi	South Korea	Landrace	-	Brown	
	G. max	Bancheongdu	IT230769	EE-1193	South Korea	Landrace			
1918	G. max	Hannam		Hannam	South Korea	Improved line	Purple	Yellow	
	G. max	Hannam		CD-1236	South Korea	Improved line			
1919	G. max	Heugcheong		Hekchung	South Korea	Landrace	Purple	Black	
	G. max	Seoritae	IT224517	EE-0958	South Korea	Landrace			
	G. max	KAS637-11	IT154553	EE-0514	South Korea	Landrace			
	G. max	kwangwon Yanggu-2001-18	IT224537	EE-0964	South Korea	Landrace			
	G. max	Geomjeong-3	IT177360	EE-0756	South Korea	Landrace			
	G. max	IT186095		EE-0836	South Korea	Landrace			
	G. max	Bidulgi	IT211802	EE-0882	South Korea	Landrace			
	G. max	Geomjeong	IT186111	EE-0837	South Korea	Landrace			
	G. max	Heugcheong		CD-1282	South Korea	Improved line			
	G. max	KLS86028	IT143228	DD-0513	South Korea	Landrace			
	G. max	Geomjeong-1	IT177285	DD-0864	South Korea	Landrace			
	G. max	Geomjeong-5	IT177318	DD-0869	South Korea	Landrace			
	G. max	Geomjeong-1	IT177325	DD-0872	South Korea	Landrace			
	G. max	Geomjeong-1	IT177373	DD-0876	South Korea	Landrace			
	G. max	Geomjeong-2	IT177405	DD-0882	South Korea	Landrace			
	G. max	Geomjeong-6	IT177415	DD-0885	South Korea	Landrace			
	G. max	IT177420		DD-0887	South Korea	Landrace			
	G. max	KAS232-11	IT181436	DD-0958	South Korea	Landrace			
	G. max	IT186015		DD-0974	South Korea	Landrace			
	G. max	Hamyang sujib	IT195627	DD-1028	South Korea	Landrace			
	G. max	kwangwon Koseong-2001-11	IT224512	DD-1146	South Korea	Landrace			
	G. max	Parangomul	IT110987	DD-0263	South Korea	Landrace			
	G. max	KAS234-10	IT141511	DD-0406	South Korea	Landrace			
	G. max	KLS86014	IT143214	EE-0433	South Korea	Landrace			
1920	G. max	Hwangkeum		Hwanggm	South Korea	Improved line	Purple	Yellow	
	G. max	Ojeong	IT180841	DD-0951	South Korea	Landrace			

	G. max	bbuyeon	IT155157	DD-0628	South Korea	Landrace			
	G. max	PI 486355B		EE-0483	South Korea	Improved line			
	G. max	SLS90-295	IT167933	EE-0721	South Korea	Landrace			
	G. max	Hwangkeum		CD-1172	South Korea	Improved line			
	G. max	Jungmo3006		CD-1207	South Korea	Improved line			
	G. max	KLS85222	IT143140	DD-0503	South Korea	Landrace			
	G. max	KLS86123	IT143177	DD-0508	South Korea	Landrace			
	G. max	SLS90-289	IT167927	DD-0824	South Korea	Landrace			
	G. max	SLS90-292	IT167930	DD-0825	South Korea	Landrace			
	G. max	Heuin	IT175813	DD-0847	South Korea	Landrace			
1921	G. max	Iksan10		Iksan10	South Korea	Improved line	-	Yellow	
	G. max	**Iksan10**		KI119_D03	South Korea	Improved line			
	G. max	**Iksan10**		KI119_A06	South Korea	Improved line			
1922	G. max	Ilpumgeomjeong		Ilpum	South Korea	Improved line	Purple	Black	
	G. max	KAS606-22	IT141885	DD-0438	South Korea	Landrace			
	G. max	Ilpumgeomjeong		CD-1274	South Korea	Improved line			
1923	G. max	Josaengseori		Josaengsuri	South Korea	Improved line	Purple	Black	
1924	G. max	KS118		KS118	South Korea	Improved line	-	Yellow	
1925	G. max	L29		L29	South Korea	Improved line	-	Yellow	
	G. max	L77-1794	PI 547890	L77-1794	United States	Improved line			
	G. max	KLS85102	IT143043	EE-0405	South Korea	Landrace			
1926	G. max	Lee68		Lee68	United States	Improved line	-	Yellow	
	G. max	Lee-68	IT023544	DD-0063	United States	Improved line			
1927	G. max	Marshall	PI 548693	Marshall	United States	Improved line	Purple	Yellow	
1928	G. max	Mooyepbyeng		Mooyepbyeng	South Korea	Improved line	-	Yellow	
1929	G. max	Ogden	PI 548477	Ogden	United States	Improved line	Purple	Green yellow	
1930	G. max	PI 189891		PI189891	France	Not determined	Purple	Yellow	
1931	G. max	PI 417329		PI417329	Japan	Landrace	-	Yellow	Representative
	G. max	Pochol	IT230795	EE-1202	South Korea	Landrace			
	G. max	Sodarikon	PI 87542	EE-1062	North Korea	Landrace			
1932	G. max	PI 486355		PI486355	South Korea	Landrace	Purple	Yellow	
	G. max	kwangwonjaerae	IT153922	EE-0482	South Korea	Landrace			
1933	G. max	Shin2		PI 507238	Japan	Landrace	-	Yellow	
	G. max	PI 507239		PI 507239	Japan	Landrace			
1934	G. max	PI 548510	Beeson	PI 548510	South Korea	Improved line	Purple	Yellow	
1935	G. max	Clark	PI 548533	PI548533-1	United States	Improved line	Purple	Yellow	
	G. max	Clark (Nod)	IT153949	EE-0484	United States	Improved line			
	G. max	Clark 63	PI 548532	EE-0312	United States	Improved line			
	G. max	L62-1579	PI547413	PI547413-1	United States	Improved line			
	G. max	Clark	PI 548533	EE-0009	United States	Improved line			
	G. max	Clark	PI 548533	EE-1275	United States	Improved line			
1936	G. max	PI 556949		PI556949	China	Landrace	White	Brown	
1937	G. max	PI 559931	Corsica	PI559931	United States	Improved line	Purple	Yellow	
1938	G. max	PJ043		PJ043	South Korea	Improved line	-	Green yellow	
1939	G. max	Pungsannamul		Poongsan	South Korea	Improved line	Purple	Yellow	
	G. max	CS00838	IT225113	EE-0974	South Korea	Landrace			
	G. max	Pungsannamul		CD-1224	South Korea	Improved line			
1940	G. max	Pungwon		Poongwon	South Korea	Improved line	White	Yellow	
	G. max	Pungwon		CD-1225	South Korea	Improved line			
1941	G. max	RRR		RRR	South Korea	Improved line	-	Yellow	
	G. max	Archer	PI 546487	Archer	United States	Improved line			
1942	G. max	Saedanbaek		Saedanbaek	South Korea	Improved line	White	Yellow	
	G. max	Saedanbaek		CD-1170	South Korea	Improved line			
1943	G. max	Sinpaldal		shin1	South Korea	Improved line	White	Yellow	
	G. max	Sinpaldal		KS120	South Korea	Improved line			
1944	G. max	Shinhwa		Shinhwa	South Korea	Improved line	Purple	Yellow	
	G. max	Shinhwa		CD-1229	South Korea	Improved line			
1945	G. max	Socheong2		Sochung2	South Korea	Improved line	White	Yellow	
	G. max	Socheong2		CD-1223	South Korea	Improved line			
1946	G. max	Soho		Soho	South Korea	Improved line	White	Yellow	
1947	G. max	Somyeong		Somyung	South Korea	Improved line	Purple	Yellow	
	G. max	Somyeong		CD-1241	South Korea	Improved line			
	G. max	CS00840	IT219764	DD-1096	South Korea	Landrace			
	G. max	Junjari	IT228823	DD-1300	South Korea	Landrace			
1948	G. max	Sowon		Sowon	South Korea	Improved line	Purple	Yellow	
	G. max	Singang		CD-1230	South Korea	Improved line			

	G. max	Sowon		CD-1243	South Korea	Improved line		
	G. max	Sowon2010		CD-1263	South Korea	Improved line		
1949	G. max	Suklyangpootkong		Suklyangpootkong	South Korea	Improved line	-	Green yellow
	G. max	IT196438		DD-1030	South Korea	Landrace		
1950	G. max	Seoritae		Suritaejung	South Korea	Landrace	Purple	Black
	G. max	CS 01937	IT237550	DD-1384	South Korea	Landrace		
1951	G. max	Taekwang		Taekwang	South Korea	Improved line	Purple	Yellow
	G. max	Taekwang		CD-1165	South Korea	Improved line		
	G. max	Jungmo3007		CD-1208	South Korea	Improved line		
	G. max	Taekwang		CD-1329	South Korea	Improved line		
1952	G. max	V94-5152	PI 596752	V94-5152_E08	United States	Improved line	Purple	Yellow
	G. max	V94-5152	PI 596752	V94-5152	United States	Improved line		
1953	G. max	Williams 82K		Williams82	United States	Improved line	White	Yellow
	G. max	Uid	IT228374	DD-1229	North Korea	Landrace		
1954	G. max	Uram		Wooram	South Korea	Improved line	White	Green yellow
	G. max	Uram		CD-1169	South Korea	Improved line		
1955	G. max	Yemsol		Yemsol	South Korea	Landrace	Purple	Black
	G. max	Yemsol	IT143295	DD-0519	South Korea	Landrace		
	G. max	Yemsol	IT175993	DD-0855	South Korea	Landrace		
1956	G. max	Yonpoong		YoenPoong	South Korea	Improved line	Purple	Yellow
	G. max	Yonpoong		CD-1211	South Korea	Improved line		
1957	G. max	York	PI 553038	York	United States	Improved line	Purple	Yellow
1958	G. max x G. soja	74050-9	IT183426	DD-0971	South Korea		White	Black
1959	G. max x G. soja	74052-5	IT183431	DD-0972	South Korea		Purple	Black
1960	G. max x G. soja	7402-6	IT191202	DD-1005	South Korea		White	Yellow
1961	G. max x G. soja	7402-18	IT191214	DD-1008	South Korea		White	Yellow
1962	G. max x G. soja	7402-23	IT191219	DD-1009	South Korea		White	Green yellow
1963	G. max x G. soja	7402-29	IT191225	DD-1010	South Korea		White	Black
1964	G. max x G. soja	7402-46	IT191242	DD-1013	South Korea		White	Yellow
1965	G. max x G. soja	Kangwon hwacheon sujib	IT188359	FF-0573	South Korea		Purple	Black
1966	G. max x G. soja	YWS 122		FF-0033	Unknown		Purple	Black
1967	G. max x G. soja	YWS 125		CD-0091	Unknown		Purple	Black
1968	G. max x G. soja	YWS 151	IT188417	CD-0100	South Korea		Purple	Black
1969	G. max x G. soja	YWS 151	IT188417	FF-0052	South Korea		Purple	Black
1970	G. max x G. soja	YWS707		CD-0464	South Korea		Purple	Black
1971	G. max x G. soja	Kangwon inje sujib	IT188371	FF-0576	South Korea		Purple	Black
1972	G. max x G. soja	YWS269		CD-0159	South Korea		Purple	Black
1973	G. max x G. soja	PI 366121		PI366121	Japan		Purple	Black
1974	G. max x G. soja	YWS237		CD-0142	South Korea		Purple	Black
1975	G. max x G. soja	YWS 80		CD-0062	South Korea		Purple	Black
1976	G. max x G. soja	YWS 80		CD-0063	South Korea		Purple	Black
1977	G. max x G. soja	YWS 74		CD-0057	South Korea		Purple	Black
1978	G. max x G. soja	YWS1270		CD-0864	South Korea		Purple	Black
1979	G. max x G. soja	PI 549048	CHN2	CD-1113	China		Purple	Black
1980	G. max x G. soja	YWS 93		CD-0072	South Korea		Purple	Black
1981	G. max x G. soja	PI 597452 C	ZYD2900, CHN57	CD-1152	China		Purple	Black
1982	G. max x G. soja	YWS 67		CD-0049	South Korea		Purple	Yellow

1983	G. max x G. soja	YWS 67		CD-0050	South Korea	Purple	Black
1984	G. max x G. soja	YWS 106		CD-0081	China	Purple	Black
1985	G. max x G. soja	YWS 110		FF-0028	China	Purple	Black
1986	G. max x G. soja	YWS 107		FF-0025	China	Purple	Black
1987	G. max x G. soja	YWS380		FF-0137	South Korea	Purple	Black
1988	G. max x G. soja	YWS 108		FF-0026	China	Purple	Black
1989	G. max x G. soja	YWS 16		CD-0012	South Korea	Purple	Black
1990	G. max x G. soja	YWS 94		CD-0073	South Korea	Purple	Yellow
1991	G. max x G. soja	74050-8	IT183425	DD-0970	South Korea, Kangwon	Purple	Black
1992	G. max x G. soja	7402-5	IT191201	DD-1004	South Korea, Gyeonggi	White	Green yellow
1993	G. max x G. soja	7402-32	IT191228	DD-1011	South Korea, Gyeonggi	White	Green yellow
1994	G. max x G. soja	7402-45	IT191241	DD-1012	South Korea, Gyeonggi	White	Black
1995	G. max x G. soja	7402-4	IT191200	DD-1003	South Korea, Gyeonggi	White	Green yellow
1996	G. max x G. soja	7402-14	IT191210	DD-1007	South Korea, Gyeonggi	White	Yellow
1997	G. max x G. soja	7402-1	IT191197	DD-1002	South Korea, Gyeonggi	White	Yellow
1998	G. max x G. soja	PI 291309 C		DD-0839	China	Purple	Brown
1999	G. max x G. soja	74002-2	IT183413	DD-0969	South Korea, Gyeonggi	White	Yellow
2000	G. max x G. soja	PI 70197		DD-0050	China	Purple	Black
2001	G. max x G. soja	PI 86046		DD-0731	Japan	Purple	Black
2002	G. max x G. soja	Chousenshu(Cha)	IT230681	EE-1161	South Korea	Purple	Brown
2003	G. max x G. soja	7402-10	IT191206	DD-1006	South Korea, Gyeonggi	White	Yellow
2004	G. max x G. soja	PI 72341		DD-0052	China	White	Black
2005	G. max x G. soja	VIR 2956	PI 438303	EE-1007	South Korea	Purple	Black
2006	G. max x G. soja	Arlington	PI 548439	EE-0894	China	Purple	Black
2007	G. max x G. soja	Bai hei dou	PI 567420	EE-1245	China	Purple	Yellow
2008	G. soja	YWS 1		CD-0001	South Korea, Gyeongbuk	Purple	Black
2009	G. soja	YWS 5		CD-0004	South Korea, Gyeongnam	Purple	Black
2010	G. soja	YWS 6		CD-0005	South Korea, Gyeongnam	Purple	Black
2011	G. soja	YWS 8		CD-0006	South Korea, Gyeongbuk	Purple	Black
2012	G. soja	YWS 9		CD-0007	South Korea, Gyeongnam	Purple	Black
2013	G. soja	YWS 10		CD-0008	South Korea, Gyeongbuk	Purple	Black
2014	G. soja	YWS 11		CD-0009	South Korea, Gyeongnam	Purple	Black
2015	G. soja	YWS 13		CD-0011	South Korea, Gyeongnam	Purple	Black
2016	G. soja	YWS 18		CD-0014	South Korea, Gyeongbuk	Purple	Black
2017	G. soja	YWS 19		CD-0015	South Korea, Gyeongnam	Purple	Black
2018	G. soja	YWS 20		CD-0016	South Korea, Gyeongnam	Purple	Black
2019	G. soja	YWS 21		CD-0017	South Korea, Gyeongbuk	Purple	Black
2020	G. soja	YWS 23		CD-0018	South Korea, Gyeongnam	Purple	Black
2021	G. soja	YWS 27		CD-0020	South Korea, Gyeongbuk	Purple	Black
2022	G. soja	YWS 28		CD-0021	South Korea, Gyeongbuk	Purple	Black
2023	G. soja	YWS 30		CD-0022	South Korea, Gyeongbuk	Purple	Black
2024	G. soja	YWS 31		CD-0023	South Korea, Gyeongbuk	Purple	Black
2025	G. soja	YWS 32		CD-0024	South Korea, Gyeongbuk	Purple	Black
2026	G. soja	YWS 33		CD-0025	South Korea, Gyeongnam	Purple	Black
2027	G. soja	YWS 34		CD-0026	South Korea, Gyeongnam	Purple	Black
2028	G. soja	YWS 35		CD-0027	South Korea, Gyeongnam	Purple	Black
2029	G. soja	YWS 36		CD-0028	South Korea, Gyeongbuk	Purple	Black
2030	G. soja	YWS 38		CD-0029	South Korea, Gyeongnam	Purple	Black
2031	G. soja	YWS 42		CD-0030	South Korea, Gyeongnam	Purple	Black

2032	G. soja	YWS 43		CD-0031	South Korea, Gyeongnam	Purple	Black	
2033	G. soja	YWS 44		CD-0032	South Korea, Gyeongbuk	Purple	Black	
2034	G. soja	YWS 46		CD-0034	South Korea, Gyeongbuk	Purple	Black	
2035	G. soja	YWS 48		CD-0035	South Korea, Gyeongbuk	Purple	Black	
2036	G. soja	YWS 50		CD-0036	South Korea, Gyeongnam	Purple	Black	
2037	G. soja	YWS 53		CD-0037	South Korea, Gyeongbuk	Purple	Black	
2038	G. soja	YWS 55		CD-0038	South Korea, Gyeongbuk	Purple	Black	
2039	G. soja	YWS 56		CD-0039	South Korea, Gyeongbuk	Purple	Black	
2040	G. soja	YWS 59		CD-0041	South Korea, Gyeongbuk	Purple	Black	
2041	G. soja	YWS 60		CD-0042	South Korea, Gyeongbuk	Purple	Black	
2042	G. soja	YWS 61		CD-0043	South Korea, Gyeongbuk	Purple	Black	
2043	G. soja	YWS 62		CD-0044	South Korea, Gyeongbuk	Purple	Black	
2044	G. soja	YWS 64		CD-0046	South Korea, Gyeongnam	Purple	Black	
2045	G. soja	YWS 65		CD-0047	South Korea, Gyeongnam	Purple	Black	
2046	G. soja	YWS 66		CD-0048	South Korea, Gyeongnam	Purple	Black	
2047	G. soja	YWS 70		CD-0053	South Korea, Gyeongnam	Purple	Black	
2048	G. soja	YWS71		CD-0054	South Korea, Gyeongnam	Purple	Black	Representative
	G. soja	YWS 101		CD-0078	South Korea, Gyeongnam			
	G. soja	YWS 72		CD-0055	South Korea, Gyeongnam			
2049	G. soja	YWS 73		CD-0056	South Korea, Gyeongnam	Purple	Black	
2050	G. soja	YWS 77		CD-0060	South Korea, Gyeongnam	Purple	Black	
	G. soja	YWS 58		CD-0040	South Korea, Gyeongbuk			
2051	G. soja	YWS 78		CD-0061	South Korea, Gyeongnam	Purple	Black	
2052	G. soja	YWS 82		CD-0064	South Korea, Gyeongnam	Purple	Black	
2053	G. soja	YWS 84		CD-0065	South Korea, Gyeongnam	Purple	Black	
	G. soja	YWS 69		CD-0052	South Korea, Gyeongnam			
2054	G. soja	YWS 85		CD-0066	South Korea, Gyeongnam	Purple	Black	
2055	G. soja	YWS 86		CD-0067	South Korea, Gyeongnam	Purple	Black	
2056	G. soja	YWS 90		CD-0069	South Korea, Gyeongnam	Purple	Black	
2057	G. soja	YWS 92		CD-0071	South Korea, Gyeongbuk	Purple	Black	
2058	G. soja	YWS 95		CD-0074	South Korea, Gyeongbuk	Purple	Black	
2059	G. soja	YWS 96		CD-0075	South Korea, Gyeongbuk	Purple	Black	
2060	G. soja	YWS 99		CD-0077	South Korea, Gyeongnam	Purple	Black	
	G. soja	YWS 74		CD-0058	South Korea, Gyeongnam			
2061	G. soja	YWS 103		CD-0079	South Korea, Gyeongnam	Purple	Black	
2062	G. soja	YWS 111	IT188363	CD-0082	South Korea, Gangwon	Purple	Black	
	G. soja	kwangwon Yanggu sujib	IT188363	FF-0574	South Korea, Gangwon			
2063	G. soja	YWS 112	IT188365	CD-0083	South Korea, Gangwon	Purple	Black	
2064	G. soja	YWS113	IT183036	CD-0084	South Korea, Gangwon	Purple	Black	Representative
2065	G. soja	YWS 114	IT183033	CD-0085	South Korea, Gangwon	Purple	Black	
2066	G. soja	YWS 116	IT184156	CD-0087	South Korea, Gangwon	Purple	Black	
2067	G. soja	YWS122		CD-0089	South Korea, Gangwon	Purple	Black	Representative
2068	G. soja	YWS 124	IT184226	CD-0090	South Korea, Gangwon	Purple	Black	
2069	G. soja	YWS 127	IT184246	CD-0092	South Korea, Gangwon	Purple	Black	
2070	G. soja	YWS 132	IT188375	CD-0094	South Korea, Gyeonggi	Purple	Black	
2071	G. soja	YWS 139	IT183026	CD-0096	South Korea, Gyeonggi	Purple	Black	
2072	G. soja	YWS 142	IT184204	CD-0097	South Korea, Gyeonggi	Purple	Black	
	G. soja	YWS 146	IT184213	CD-0099	South Korea, Gyeongnam			
2073	G. soja	YWS 143	IT184207	CD-0098	South Korea, Gyeonggi	Purple	Black	
2074	G. soja	YWS 154	IT183044	CD-0101	South Korea, Gyeongbuk	Purple	Black	
2075	G. soja	YWS 157	IT188412	CD-0103	South Korea, Gyeongbuk	Purple	Black	
2076	G. soja	YWS 161	IT182859	CD-0104	South Korea, Gyeongbuk	Purple	Black	
2077	G. soja	YWS 162	IT103384	CD-0105	South Korea, Gyeongbuk	Purple	Black	
	G. soja	Kyoungbuk Yeacheonsujib	IT103384	FF-0471	South Korea, Gyeongbuk			
2078	G. soja	YWS 164	IT188415	CD-0106	South Korea, Gyeongbuk	Purple	Black	
2079	G. soja	YWS 165	IT188413	CD-0107	South Korea, Gyeongbuk	Purple	Black	
2080	G. soja	YWS 166	IT183001	CD-0108	South Korea, Jeonnam	Purple	Black	
2081	G. soja	YWS 167	IT188421	CD-0109	South Korea, Jeonnam	Purple	Black	
	G. soja	Kyoungbuk Cheongdo sujib	IT188431	FF-0591	South Korea, Gyeongbuk			
2082	G. soja	YWS 173	IT182841	CD-0110	South Korea, Jeonbuk	Purple	Black	
	G. soja	YWS 173	IT182841	CD-0111	South Korea, Jeonbuk			
2083	G. soja	YWS 176	IT182821	CD-0112	South Korea, Chungnam	Purple	Black	
	G. soja	YWS 195		CD-0128	South Korea, Chungbuk			
2084	G. soja	YWS 177	IT178539	CD-0113	South Korea, Chungnam	Purple	Black	
	G. soja	YWS 177	IT178539	CD-0114	South Korea, Chungnam			

2085	G. soja	YWS 178	IT183013	CD-0115	South Korea, Chungnam	Purple	Black	
2086	G. soja	YWS 181	IT178543	CD-0118	South Korea, Chungbuk	Purple	Black	
	G. soja	YWS 187	IT183041	CD-0121	South Korea, Chungbuk			
2087	G. soja	YWS 184	IT188397	CD-0119	South Korea, Chungbuk	Purple	Black	
	G. soja	YWS 183	IT188393	FF-0070	South Korea, Chungbuk			
2088	G. soja	YWS 185	IT178486	CD-0120	South Korea, Chungbuk	Purple	Black	
2089	G. soja	YWS 191	IT188386	CD-0125	South Korea, Chungnam	Purple	Black	
	G. soja	YWS 87		CD-0068	South Korea, Gyeongnam			
	G. soja	YWS 190		CD-0124	South Korea, Chungbuk			
2090	G. soja	YWS 192	IT188399	CD-0126	South Korea, Chungbuk	Purple	Black	
2091	G. soja	YWS 193	IT183098	CD-0127	South Korea, Chungbuk	Purple	Black	
2092	G. soja	YWS 199	IT188396	CD-0129	South Korea, Chungbuk	Purple	Black	
2093	G. soja	YWS 200	IT195552	CD-0130	South Korea, Chungbuk	Purple	Black	
2094	G. soja	YWS 202	IT195551	CD-0131	South Korea, Chungnam	Purple	Black	
2095	G. soja	YWS 204	IT183052	CD-0132	South Korea, Chungnam	Purple	Black	
	G. soja	PI 378683		CD-1157	Japan, Ishikawa			
2096	G. soja	YWS216		CD-0133	South Korea, Jeonnam	Purple	Black	
	G. soja	YWS238		CD-0143	South Korea, Jeonnam			
	G. soja	YWS242		CD-0144	South Korea, Jeonnam			
	G. soja	YWS243		CD-0145	South Korea, Jeonnam			
	G. soja	YWS228		CD-0138	South Korea, Jeonnam			
2097	G. soja	YWS225		CD-0136	South Korea, Jeonnam	Purple	Black	
	G. soja	YWS235		CD-0141	South Korea, Jeonnam			
2098	G. soja	YWS229		CD-0139	South Korea, Jeonnam	Purple	Black	
2099	G. soja	YWS234		CD-0140	South Korea, Jeonnam	Purple	Black	
2100	G. soja	YWS244		CD-0146	South Korea, Jeonnam	Purple	Black	
	G. soja	YWS223		CD-0135	South Korea, Jeonnam			
	G. soja	YWS226		CD-0137	South Korea, Jeonnam			
2101	G. soja	YWS248		CD-0148	South Korea, Jeonnam	Purple	Black	
2102	G. soja	YWS251		CD-0149	South Korea, Jeonnam	Purple	Black	
2103	G. soja	YWS253		CD-0150	South Korea, Jeonnam	Purple	Black	
2104	G. soja	YWS255		CD-0152	South Korea, Jeonnam	Purple	Black	
	G. soja	YWS257		FF-0100	South Korea, Jeonnam			
	G. soja	YWS255		CD-0151	South Korea, Jeonnam			
2105	G. soja	YWS259		CD-0153	South Korea, Jeonnam	Purple	Black	
2106	G. soja	YWS265		CD-0156	South Korea, Jeonbuk	Purple	Black	
	G. soja	YWS277		CD-0164	South Korea, Jeonbuk			
	G. soja	YWS280		CD-0166	South Korea, Jeonbuk			
	G. soja	YWS289		CD-0170	South Korea, Jeonbuk			
	G. soja	YWS266		FF-0103	South Korea, Jeonbuk			
	G. soja	YWS261		CD-0154	South Korea, Jeonbuk			
	G. soja	YWS262		CD-0155	South Korea, Jeonbuk			
	G. soja	YWS267		CD-0157	South Korea, Jeonbuk			
	G. soja	YWS268		CD-0158	South Korea, Jeonbuk			
	G. soja	YWS270		CD-0160	South Korea, Jeonbuk			
2107	G. soja	YWS276		CD-0163	South Korea, Jeonbuk	Purple	Black	
	G. soja	YWS279		CD-0165	South Korea, Jeonbuk			
	G. soja	YWS275		FF-0105	South Korea, Jeonbuk			
	G. soja	YWS246		CD-0147	South Korea, Jeonnam			
	G. soja	YWS272		CD-0161	South Korea, Jeonbuk			
	G. soja	YWS274		CD-0162	South Korea, Jeonbuk			
2108	G. soja	YWS285		CD-0167	South Korea, Jeonbuk	Purple	Black	
2109	G. soja	YWS287		CD-0168	South Korea, Jeonbuk	Purple	Black	
2110	G. soja	YWS288		CD-0169	South Korea, Jeonbuk	Purple	Black	Representative
2111	G. soja	YWS294		CD-0171	South Korea, Chungnam	Purple	Black	
2112	G. soja	YWS296		CD-0172	South Korea, Chungnam	Purple	Black	
2113	G. soja	YWS298		CD-0173	South Korea, Chungnam	Purple	Black	
2114	G. soja	YWS299		CD-0174	South Korea, Chungnam	Purple	Black	
	G. soja	YWS314		CD-0185	South Korea, Chungnam			
2115	G. soja	YWS306		CD-0179	South Korea, Chungnam	Purple	Black	
2116	G. soja	YWS308		CD-0181	South Korea, Chungnam	Purple	Black	
2117	G. soja	YWS309		CD-0182	South Korea, Chungnam	Purple	Black	
2118	G. soja	YWS310		CD-0183	South Korea, Chungnam	Purple	Black	
2119	G. soja	YWS319		CD-0187	South Korea, Chungbuk	Purple	Black	
2120	G. soja	YWS320		CD-0188	South Korea, Chungbuk	Purple	Black	
2121	G. soja	YWS323		CD-0189	South Korea, Chungbuk	Purple	Black	
2122	G. soja	YWS324		CD-0190	South Korea, Chungbuk	Purple	Black	

2123	G. soja	YWS331	CD-0193	South Korea, Chungbuk	Purple	Black	
2124	G. soja	YWS334	CD-0195	South Korea, Chungbuk	Purple	Black	
	G. soja	YWS307	CD-0180	South Korea, Chungnam			
2125	G. soja	YWS335	CD-0196	South Korea, Chungbuk	Purple	Black	
2126	G. soja	YWS336	CD-0197	South Korea, Chungbuk	Purple	Black	
2127	G. soja	YWS340	CD-0198	South Korea, Chungbuk	Purple	Black	Representative
2128	G. soja	YWS341	CD-0199	South Korea, Chungbuk	Purple	Black	
2129	G. soja	YWS349	CD-0203	South Korea, Chungbuk	Purple	Black	
2130	G. soja	YWS354	CD-0206	South Korea, Chungbuk	Purple	Black	
2131	G. soja	YWS355	CD-0207	South Korea, Chungbuk	Purple	Black	
2132	G. soja	YWS360	CD-0211	South Korea, Chungbuk	Purple	Black	
	G. soja	YWS384	CD-0227	South Korea, Chungbuk			
2133	G. soja	YWS364	CD-0214	South Korea, Chungbuk	Purple	Black	
	G. soja	YWS385	CD-0228	South Korea, Chungbuk			
2134	G. soja	YWS366	CD-0216	South Korea, Chungbuk	Purple	Black	
	G. soja	YWS369	CD-0218	South Korea, Chungbuk			
	G. soja	YWS370	CD-0219	South Korea, Chungbuk			
	G. soja	YWS379	CD-0226	South Korea, Chungbuk			
	G. soja	YWS374	FF-0135	South Korea, Chungbuk			
	G. soja	YWS382	FF-0139	South Korea, Chungbuk			
	G. soja	YWS352	CD-0204	South Korea, Chungbuk			
	G. soja	YWS357	CD-0208	South Korea, Chungbuk			
	G. soja	YWS359	CD-0210	South Korea, Chungbuk			
	G. soja	YWS362	CD-0213	South Korea, Chungbuk			
	G. soja	YWS365	CD-0215	South Korea, Chungbuk			
2135	G. soja	YWS368	CD-0217	South Korea, Chungbuk	Purple	Black	
2136	G. soja	YWS373	CD-0222	South Korea, Chungbuk	Purple	Black	
2137	G. soja	YWS388	CD-0230	South Korea, Gyeonggi	Purple	Black	
2138	G. soja	YWS391	CD-0233	South Korea, Gyeonggi	Purple	Black	
2139	G. soja	YWS401	CD-0238	South Korea, Gyeongbuk	Purple	Black	
2140	G. soja	YWS403	CD-0240	South Korea, Gyeongbuk	Purple	Black	
2141	G. soja	YWS405	CD-0241	South Korea, Gyeongbuk	Purple	Black	
2142	G. soja	YWS406	CD-0242	South Korea, Gyeongnam	Purple	Black	
2143	G. soja	YWS408	CD-0244	South Korea, Gyeongnam	Purple	Black	Representative
2144	G. soja	YWS409	CD-0245	South Korea, Gyeongnam	Purple	Black	
2145	G. soja	YWS413	CD-0248	South Korea, Gangwon	Purple	Black	
2146	G. soja	YWS414	CD-0249	South Korea, Gangwon	Purple	Black	
2147	G. soja	YWS415	CD-0250	South Korea, Gangwon	White	Black	
	G. soja	YWS416	CD-0251	South Korea, Gangwon			
2148	G. soja	YWS418	CD-0252	South Korea, Gangwon	Purple	Black	
2149	G. soja	YWS419	CD-0253	South Korea, Gangwon	Purple	Black	
2150	G. soja	YWS420A	CD-0254	South Korea, Gangwon	Purple	Black	
2151	G. soja	YWS422	CD-0256	South Korea, Gangwon	Purple	Black	
2152	G. soja	YWS423	CD-0257	South Korea, Gangwon	Purple	Black	
2153	G. soja	YWS424	CD-0258	South Korea, Gangwon	Purple	Black	
2154	G. soja	YWS425	CD-0259	South Korea, Gangwon	Purple	Black	
2155	G. soja	YWS427	CD-0261	South Korea, Gangwon	Purple	Black	
2156	G. soja	YWS429	CD-0262	South Korea, Gangwon	Purple	Black	
2157	G. soja	YWS430	CD-0263	South Korea, Gangwon	Purple	Black	
2158	G. soja	YWS432	CD-0264	South Korea, Gangwon	Purple	Black	
2159	G. soja	YWS434	CD-0265	South Korea, Gangwon	Purple	Black	
2160	G. soja	YWS435	CD-0266	South Korea, Gangwon	Purple	Black	
2161	G. soja	YWS436	CD-0267	South Korea, Gangwon	Purple	Black	
2162	G. soja	YWS437	CD-0268	South Korea, Gangwon	Purple	Black	
2163	G. soja	YWS438	CD-0269	South Korea, Gangwon	Purple	Black	
2164	G. soja	YWS439	CD-0270	South Korea, Gangwon	Purple	Black	
2165	G. soja	YWS440	CD-0271	South Korea, Gangwon	Purple	Black	
2166	G. soja	YWS441	CD-0272	South Korea, Gangwon	Purple	Black	Representative
	G. soja	YWS445	CD-0275	South Korea, Gangwon			
2167	G. soja	YWS443	CD-0273	South Korea, Gangwon	Purple	Black	
2168	G. soja	YWS444	CD-0274	South Korea, Gangwon	Purple	Black	
2169	G. soja	YWS446	CD-0276	South Korea, Gangwon	Purple	Black	
2170	G. soja	YWS447	CD-0277	South Korea, Gangwon	Purple	Black	
2171	G. soja	YWS451	CD-0279	South Korea, Gangwon	Purple	Black	
2172	G. soja	YWS452	CD-0280	South Korea, Gangwon	Purple	Black	
2173	G. soja	YWS453	CD-0281	South Korea, Gangwon	Purple	Black	
2174	G. soja	YWS454	CD-0282	South Korea, Gangwon	Purple	Black	

2175	G. soja	YWS455	CD-0283	South Korea, Gangwon	Purple	Black	
2176	G. soja	YWS459	CD-0284	South Korea, Gangwon	Purple	Black	
2177	G. soja	YWS460	CD-0285	South Korea, Gangwon	Purple	Black	
2178	G. soja	YWS461	CD-0286	South Korea, Gangwon	Purple	Black	
2179	G. soja	YWS462	CD-0287	South Korea, Gangwon	Purple	Black	
	G. soja	YWS1393	CD-0939	South Korea, Chungbuk			
	G. soja	YWS1377	CD-0930	South Korea, Gyeongbuk			
2180	G. soja	YWS464	CD-0288	South Korea, Gangwon	Purple	Black	
2181	G. soja	YWS465	CD-0289	South Korea, Gangwon	Purple	Black	
2182	G. soja	YWS468	CD-0290	South Korea, Gangwon	Purple	Black	
2183	G. soja	YWS469	CD-0291	South Korea, Gangwon	Purple	Black	
2184	G. soja	YWS470	CD-0292	South Korea, Gangwon	Purple	Black	
2185	G. soja	YWS472	CD-0294	South Korea, Gangwon	Purple	Black	
2186	G. soja	YWS473	CD-0295	South Korea, Gangwon	Purple	Black	
2187	G. soja	YWS474	CD-0296	South Korea, Gangwon	Purple	Black	
2188	G. soja	YWS475	CD-0297	South Korea, Gangwon	Purple	Black	
2189	G. soja	YWS478	CD-0299	South Korea, Gangwon	Purple	Black	Representative
2190	G. soja	YWS480	CD-0300	South Korea, Gangwon	Purple	Black	
2191	G. soja	YWS482	CD-0302	South Korea, Gangwon	Purple	Black	
2192	G. soja	YWS483	CD-0303	South Korea, Gangwon	Purple	Black	Representative
	G. soja	YWS504	CD-0323	South Korea, Gangwon			
2193	G. soja	YWS484	CD-0304	South Korea, Gangwon	Purple	Black	
2194	G. soja	YWS485	CD-0305	South Korea, Gangwon	Purple	Black	
2195	G. soja	YWS487	CD-0307	South Korea, Gangwon	Purple	Black	
2196	G. soja	YWS488	CD-0308	South Korea, Gangwon	Purple	Black	Representative
2197	G. soja	YWS489	CD-0309	South Korea, Gangwon	Purple	Black	
2198	G. soja	YWS490	CD-0310	South Korea, Gangwon	Purple	Black	
2199	G. soja	YWS491	CD-0311	South Korea, Gangwon	Purple	Black	
2200	G. soja	YWS492	CD-0312	South Korea, Gangwon	Purple	Black	
2201	G. soja	YWS493	CD-0313	South Korea, Gangwon	Purple	Black	
	G. soja	YWS504	FF-0163	South Korea, Gangwon			
2202	G. soja	YWS495	CD-0314	South Korea, Gangwon	Purple	Black	
2203	G. soja	YWS496	CD-0315	South Korea, Gangwon	Purple	Black	
2204	G. soja	YWS497	CD-0316	South Korea, Gangwon	Purple	Black	
2205	G. soja	YWS498	CD-0317	South Korea, Gangwon	Purple	Black	
2206	G. soja	YWS499	CD-0318	South Korea, Gangwon	Purple	Brown	
2207	G. soja	YWS500	CD-0319	South Korea, Gangwon	Purple	Black	
2208	G. soja	YWS501	CD-0320	South Korea, Gangwon	Purple	Black	
2209	G. soja	YWS502	CD-0321	South Korea, Gangwon	Purple	Black	
2210	G. soja	YWS503	CD-0322	South Korea, Gangwon	Purple	Black	
2211	G. soja	YWS505	CD-0324	South Korea, Gangwon	Purple	Black	
2212	G. soja	YWS507	CD-0326	South Korea, Gangwon	Purple	Black	Representative
2213	G. soja	YWS508	CD-0327	South Korea, Gangwon	Purple	Black	
2214	G. soja	YWS509	CD-0328	South Korea, Gangwon	Purple	Black	
2215	G. soja	YWS510	CD-0329	South Korea, Gangwon	Purple	Black	Representative
2216	G. soja	YWS511	CD-0330	South Korea, Gangwon	Purple	Black	Representative
2217	G. soja	YWS512	CD-0331	South Korea, Gangwon	Purple	Black	
2218	G. soja	YWS513	CD-0332	South Korea, Gangwon	Purple	Black	
2219	G. soja	YWS514	CD-0333	South Korea, Gangwon	Purple	Black	
2220	G. soja	YWS516	CD-0334	South Korea, Gangwon	Purple	Black	
2221	G. soja	YWS517	CD-0335	South Korea, Gangwon	Purple	Black	
2222	G. soja	YWS518	CD-0336	South Korea, Gangwon	Purple	Black	
2223	G. soja	YWS519	CD-0337	South Korea, Gyeonggi	Purple	Black	
2224	G. soja	YWS520	CD-0338	South Korea, Gyeonggi	Purple	Black	
2225	G. soja	YWS521	CD-0339	South Korea, Gyeonggi	Purple	Black	
2226	G. soja	YWS522	CD-0340	South Korea, Gyeonggi	Purple	Black	
2227	G. soja	YWS523	CD-0341	South Korea, Gyeonggi	Purple	Black	
2228	G. soja	YWS524	CD-0342	South Korea, Gyeonggi	Purple	Black	
2229	G. soja	YWS526	CD-0344	South Korea, Gyeonggi	Purple	Black	
2230	G. soja	YWS527	CD-0345	South Korea, Gyeonggi	Purple	Black	
2231	G. soja	YWS528	CD-0346	South Korea, Gyeonggi	Purple	Black	Representative
2232	G. soja	YWS529	CD-0347	South Korea, Gyeonggi	Purple	Black	
2233	G. soja	YWS530	CD-0348	South Korea, Gyeonggi	Purple	Black	
2234	G. soja	YWS534	CD-0349	South Korea, Gyeonggi	Purple	Black	
2235	G. soja	YWS535	CD-0350	South Korea, Gyeonggi	Purple	Black	
2236	G. soja	YWS536	CD-0351	South Korea, Gyeonggi	Purple	Black	
2237	G. soja	YWS537	CD-0352	South Korea, Gyeonggi	Purple	Black	

2238	G. soja	YWS538	CD-0353	South Korea, Gyeonggi	Purple	Black	
2239	G. soja	YWS541	CD-0354	South Korea, Gyeonggi	Purple	Black	
2240	G. soja	YWS546	CD-0355	South Korea, Gyeonggi	Purple	Black	
2241	G. soja	YWS550	CD-0356	South Korea, Gyeonggi	Purple	Black	
2242	G. soja	YWS551	CD-0357	South Korea, Gyeonggi	Purple	Black	
2243	G. soja	YWS552	CD-0358	South Korea, Gyeonggi	Purple	Black	
2244	G. soja	YWS554	CD-0359	South Korea, Gyeonggi	Purple	Black	
2245	G. soja	YWS556	CD-0361	South Korea, Gyeonggi	Purple	Black	
2246	G. soja	YWS558	CD-0362	South Korea, Gyeonggi	Purple	Black	
	G. soja	YWS557	FF-0178	South Korea, Gyeonggi			
2247	G. soja	YWS560	CD-0363	South Korea, Gyeonggi	Purple	Black	
2248	G. soja	YWS561	CD-0364	South Korea, Gyeonggi	Purple	Black	
	G. soja	YWS557	CD-0365	South Korea, Gyeonggi			
2249	G. soja	YWS563	CD-0366	South Korea, Gyeonggi	Purple	Black	
2250	G. soja	YWS564	CD-0367	South Korea, Gyeonggi	Purple	Black	
2251	G. soja	YWS565	CD-0368	South Korea, Gyeonggi	Purple	Black	
2252	G. soja	YWS566	CD-0369	South Korea, Gyeonggi	Purple	Black	
2253	G. soja	YWS567	CD-0370	South Korea, Gyeonggi	Purple	Black	
2254	G. soja	YWS570	CD-0372	South Korea, Gyeonggi	Purple	Black	
2255	G. soja	YWS571	CD-0373	South Korea, Gyeonggi	Purple	Black	
2256	G. soja	YWS572	CD-0374	South Korea, Gyeonggi	Purple	Black	
	G. soja	YWS557	FF-0124	South Korea, Chungbuk			
2257	G. soja	YWS573	CD-0375	South Korea, Gyeonggi	Purple	Black	
2258	G. soja	YWS575	CD-0377	South Korea, Gyeonggi	Purple	Black	
2259	G. soja	YWS579	CD-0380	South Korea, Gyeonggi	Purple	Black	
2260	G. soja	YWS580	CD-0381	South Korea, Gyeonggi	Purple	Black	
2261	G. soja	YWS581	CD-0382	South Korea, Gyeonggi	Purple	Black	
2262	G. soja	YWS584	CD-0385	South Korea, Gyeonggi	Purple	Black	
2263	G. soja	YWS585	CD-0386	South Korea, Gyeonggi	Purple	Black	
2264	G. soja	YWS586	CD-0387	South Korea, Gyeonggi	Purple	Black	
	G. soja	YWS592	CD-0393	South Korea, Gyeonggi			
	G. soja	YWS591	CD-0392	South Korea, Gyeonggi			
2265	G. soja	YWS587	CD-0388	South Korea, Gyeonggi	Purple	Black	
2266	G. soja	YWS588	CD-0389	South Korea, Gyeonggi	Purple	Black	
2267	G. soja	YWS589	CD-0390	South Korea, Gyeonggi	Purple	Black	
2268	G. soja	YWS590	CD-0391	South Korea, Gyeonggi	Purple	Black	
2269	G. soja	YWS593	CD-0394	South Korea, Gyeonggi	Purple	Black	
2270	G. soja	YWS594	CD-0395	South Korea, Gyeonggi	Purple	Black	
2271	G. soja	YWS595	CD-0396	South Korea, Gyeonggi	Purple	Black	
2272	G. soja	YWS597	CD-0398	South Korea, Gyeonggi	Purple	Black	
2273	G. soja	YWS598	CD-0399	South Korea, Gyeonggi	Purple	Black	
2274	G. soja	YWS599	CD-0400	South Korea, Gyeonggi	Purple	Black	
2275	G. soja	YWS600	CD-0401	South Korea, Gyeonggi	Purple	Black	
2276	G. soja	YWS601	CD-0402	South Korea, Gyeonggi	Purple	Black	
2277	G. soja	YWS602	CD-0403	South Korea, Gyeonggi	Purple	Black	
2278	G. soja	YWS603	CD-0404	South Korea, Gyeonggi	Purple	Black	Representative
2279	G. soja	YWS604	CD-0405	South Korea, Gyeonggi	Purple	Black	
2280	G. soja	YWS605	CD-0406	South Korea, Gyeonggi	Purple	Black	
2281	G. soja	YWS608	CD-0407	South Korea, Gyeonggi	Purple	Black	
	G. soja	YWS609	CD-0408	South Korea, Gyeonggi			
2282	G. soja	YWS610	CD-0409	South Korea, Gyeonggi	Purple	Black	
2283	G. soja	YWS613	CD-0410	South Korea, Gyeonggi	Purple	Black	
2284	G. soja	YWS614	CD-0411	South Korea, Gyeonggi	Purple	Black	
2285	G. soja	YWS615	CD-0412	South Korea, Gyeonggi	Purple	Black	
2286	G. soja	YWS616	CD-0413	South Korea, Gyeonggi	Purple	Black	
2287	G. soja	YWS617	CD-0414	South Korea, Gyeonggi	Purple	Black	
2288	G. soja	YWS619	CD-0415	South Korea, Gyeonggi	Purple	Black	
2289	G. soja	YWS620	CD-0416	South Korea, Gyeonggi	Purple	Black	
2290	G. soja	YWS621	CD-0417	South Korea, Gyeonggi	Purple	Black	
2291	G. soja	YWS623	CD-0419	South Korea, Gyeonggi	Purple	Black	
	G. soja	YWS622	CD-0418	South Korea, Gyeonggi			
2292	G. soja	YWS629	CD-0420	South Korea, Gyeonggi	Purple	Black	
2293	G. soja	YWS636	CD-0423	South Korea, Gyeonggi	Purple	Black	
2294	G. soja	YWS637	CD-0424	South Korea, Gyeonggi	Purple	Black	
2295	G. soja	YWS638	CD-0425	South Korea, Gyeonggi	Purple	Black	
2296	G. soja	YWS641	CD-0426	South Korea, Gyeonggi	Purple	Black	
2297	G. soja	YWS643	CD-0427	South Korea, Gyeonggi	Purple	Black	

2298	G. soja	YWS644	CD-0428	South Korea, Gyeonggi	Purple	Black	
2299	G. soja	YWS650	CD-0429	South Korea, Jeju	Purple	Black	
2300	G. soja	YWS651	CD-0430	South Korea, Jeju	Purple	Black	
2301	G. soja	YWS652	CD-0431	South Korea, Jeju	Purple	Black	
2302	G. soja	YWS654	CD-0432	South Korea, Jeju	Purple	Black	
2303	G. soja	YWS656	CD-0434	South Korea, Jeju	Purple	Black	
2304	G. soja	YWS657	CD-0435	South Korea, Jeju	Purple	Black	
2305	G. soja	YWS659	CD-0437	South Korea, Jeju	Purple	Black	
2306	G. soja	YWS664	CD-0439	South Korea, Jeju	Purple	Black	
2307	G. soja	YWS666	CD-0441	South Korea, Jeju	Purple	Black	
2308	G. soja	YWS667	CD-0442	South Korea, Jeju	Purple	Black	
2309	G. soja	YWS668	CD-0443	South Korea, Jeju	Purple	Black	Representative
2310	G. soja	YWS669	CD-0444	South Korea, Jeju	Purple	Black	
2311	G. soja	YWS671	CD-0445	South Korea, Jeju	Purple	Black	
2312	G. soja	YWS672	CD-0446	South Korea, Jeju	Purple	Black	Representative
2313	G. soja	YWS673	CD-0447	South Korea, Jeju	Purple	Black	
2314	G. soja	YWS675	CD-0448	South Korea, Jeju	Purple	Black	
	G. soja	YWS676	CD-0449	South Korea, Jeju			
2315	G. soja	YWS677	CD-0450	South Korea, Jeju	Purple	Black	
2316	G. soja	YWS678	CD-0451	South Korea, Jeju	Purple	Black	
2317	G. soja	YWS679	CD-0452	South Korea, Jeju	Purple	Black	
2318	G. soja	YWS681	CD-0453	South Korea, Jeju	Purple	Black	
2319	G. soja	YWS682	CD-0454	South Korea, Jeju	Purple	Black	Representative
2320	G. soja	YWS683	CD-0455	South Korea, Jeju	Purple	Black	
	G. soja	YWS647	FF-0200	South Korea, Jeju			
2321	G. soja	YWS684	CD-0456	South Korea, Jeju	Purple	Black	
2322	G. soja	YWS687	CD-0457	South Korea, Chungbuk	Purple	Black	
2323	G. soja	YWS688	CD-0458	South Korea, Chungbuk	Purple	Black	
2324	G. soja	YWS690	CD-0459	South Korea, Chungbuk	Purple	Black	
2325	G. soja	YWS700	CD-0460	South Korea, Chungbuk	Purple	Black	
2326	G. soja	YWS703	CD-0461	South Korea, Chungbuk	Purple	Black	
2327	G. soja	YWS705	CD-0462	South Korea, Chungbuk	Purple	Black	
2328	G. soja	YWS706	CD-0463	South Korea, Chungbuk	Purple	Black	
2329	G. soja	YWS710	CD-0465	South Korea, Chungbuk	Purple	Black	
	G. soja	YWS1472	CD-0979	South Korea, Gyeongbuk			
2330	G. soja	YWS712	CD-0466	South Korea, Chungbuk	Purple	Black	
2331	G. soja	YWS714	CD-0467	South Korea, Chungbuk	Purple	Black	
2332	G. soja	YWS715	CD-0468	South Korea, Chungbuk	Purple	Black	
2333	G. soja	YWS716	CD-0469	South Korea, Chungbuk	Purple	Black	
2334	G. soja	YWS717	CD-0470	South Korea, Chungbuk	Purple	Black	
2335	G. soja	YWS718	CD-0471	South Korea, Chungbuk	Purple	Black	
2336	G. soja	YWS719	CD-0472	South Korea, Chungbuk	Purple	Black	
2337	G. soja	YWS720	CD-0473	South Korea, Chungbuk	Purple	Black	
2338	G. soja	YWS721	CD-0474	South Korea, Chungbuk	Purple	Black	
2339	G. soja	YWS723	CD-0475	South Korea, Chungbuk	Purple	Black	
2340	G. soja	YWS729	CD-0479	South Korea, Chungnam	Purple	Black	Representative
2341	G. soja	YWS730	CD-0480	South Korea, Chungnam	Purple	Black	
	G. soja	YWS726	CD-0477	South Korea, Chungnam			
	G. soja	YWS727	CD-0478	South Korea, Chungnam			
2342	G. soja	YWS731	CD-0481	South Korea, Chungnam	Purple	Black	
2343	G. soja	YWS732	CD-0482	South Korea, Chungnam	Purple	Black	
	G. soja	YWS734	CD-0484	South Korea, Chungnam			
	G. soja	YWS735	CD-0485	South Korea, Chungnam			
	G. soja	YWS736	CD-0486	South Korea, Chungnam			
	G. soja	YWS739	CD-0489	South Korea, Chungnam			
	G. soja	YWS741	CD-0491	South Korea, Chungnam			
	G. soja	YWS743	CD-0492	South Korea, Chungnam			
2344	G. soja	YWS733	CD-0483	South Korea, Chungnam	Purple	Black	
2345	G. soja	YWS737	CD-0487	South Korea, Chungnam	Purple	Black	
	G. soja	YWS740	CD-0490	South Korea, Chungnam			
2346	G. soja	YWS738	CD-0488	South Korea, Chungnam	Purple	Black	
2347	G. soja	YWS747	CD-0496	South Korea, Chungnam	Purple	Black	
	G. soja	YWS754	CD-0503	South Korea, Chungnam			
	G. soja	YWS755	CD-0504	South Korea, Chungnam			
	G. soja	YWS935	CD-0632	South Korea, Chungnam			
2348	G. soja	YWS748	CD-0497	South Korea, Chungnam	Purple	Black	
	G. soja	YWS744	CD-0493	South Korea, Chungnam			

	G. soja	YWS745	CD-0494	South Korea, Chungnam		
	G. soja	YWS746	CD-0495	South Korea, Chungnam		
	G. soja	YWS749	CD-0498	South Korea, Chungnam		
	G. soja	YWS750	CD-0499	South Korea, Chungnam		
2349	G. soja	YWS751	CD-0500	South Korea, Chungnam	Purple	Black
2350	G. soja	YWS752	CD-0501	South Korea, Chungnam	Purple	Black
2351	G. soja	YWS753	CD-0502	South Korea, Chungnam	Purple	Black
2352	G. soja	YWS756	CD-0505	South Korea, Chungnam	Purple	Black
2353	G. soja	YWS760	CD-0508	South Korea, Chungnam	Purple	Black
2354	G. soja	YWS761	CD-0509	South Korea, Chungnam	Purple	Black
	G. soja	YWS757	CD-0506	South Korea, Chungnam		
	G. soja	YWS759	CD-0507	South Korea, Chungnam		
2355	G. soja	YWS762	CD-0510	South Korea, Jeonbuk	Purple	Black
	G. soja	YWS764	FF-0229	South Korea, Jeonbuk		
	G. soja	YWS763	CD-0511	South Korea, Jeonbuk		
2356	G. soja	YWS769	CD-0514	South Korea, Jeonbuk	Purple	Black
2357	G. soja	YWS783	CD-0524	South Korea, Jeonbuk	Purple	Black
	G. soja	YWS786	CD-0527	South Korea, Jeonbuk		
	G. soja	YWS780	CD-0521	South Korea, Jeonbuk		
	G. soja	YWS782	CD-0523	South Korea, Jeonbuk		
	G. soja	YWS784	CD-0525	South Korea, Jeonbuk		
	G. soja	YWS785	CD-0526	South Korea, Jeonbuk		
	G. soja	YWS778	CD-0519	South Korea, Jeonbuk		
	G. soja	YWS779	CD-0520	South Korea, Jeonbuk		
2358	G. soja	YWS788	CD-0529	South Korea, Jeonnam	Purple	Black
2359	G. soja	YWS798	CD-0536	South Korea, Jeonnam	Purple	Black
	G. soja	YWS796	CD-0535	South Korea, Jeonnam		
	G. soja	YWS801	CD-0537	South Korea, Jeonnam		
	G. soja	YWS802	CD-0538	South Korea, Jeonnam		
	G. soja	YWS799	FF-0239	South Korea, Jeonnam		
	G. soja	YWS800	FF-0240	South Korea, Jeonnam		
	G. soja	YWS792	CD-0531	South Korea, Jeonnam		
	G. soja	YWS793	CD-0532	South Korea, Jeonnam		
	G. soja	YWS794	CD-0533	South Korea, Jeonnam		
	G. soja	YWS795	CD-0534	South Korea, Jeonnam		
2360	G. soja	YWS806	CD-0540	South Korea, Jeonnam	Purple	Black
	G. soja	YWS808	CD-0541	South Korea, Jeonnam		
	G. soja	YWS810	CD-0542	South Korea, Jeonnam		
	G. soja	YWS811	CD-0543	South Korea, Jeonnam		
	G. soja	YWS813	CD-0545	South Korea, Jeonnam		
	G. soja	YWS814	CD-0546	South Korea, Jeonnam		
	G. soja	YWS809	FF-0244	South Korea, Jeonnam		
	G. soja	YWS807	FF-0243	South Korea, Jeonnam		
	G. soja	YWS805	CD-0539	South Korea, Jeonnam		
2361	G. soja	YWS828	CD-0556	South Korea, Gangwon	Purple	Black
	G. soja	YWS823	CD-0551	South Korea, Gangwon		
	G. soja	YWS833	CD-0561	South Korea, Gangwon		
	G. soja	YWS840	CD-0566	South Korea, Gangwon		
2362	G. soja	YWS832	CD-0560	South Korea, Gangwon	Purple	Black
	G. soja	YWS829	CD-0557	South Korea, Gangwon		
	G. soja	YWS831	CD-0559	South Korea, Gangwon		
2363	G. soja	YWS835	CD-0562	South Korea, Gangwon	Purple	Black
2364	G. soja	YWS836	CD-0563	South Korea, Gangwon	Purple	Black
2365	G. soja	YWS838	CD-0564	South Korea, Gangwon	Purple	Black
	G. soja	YWS830	CD-0558	South Korea, Gangwon		
	G. soja	YWS812	CD-0544	South Korea, Jeonnam		
	G. soja	YWS824	CD-0552	South Korea, Gangwon		
	G. soja	YWS826	CD-0554	South Korea, Gangwon		
	G. soja	YWS827	CD-0555	South Korea, Gangwon		
2366	G. soja	YWS839	CD-0565	South Korea, Gangwon	Purple	Black
2367	G. soja	YWS841	CD-0567	South Korea, Gangwon	Purple	Black
	G. soja	YWS842	CD-0568	South Korea, Gangwon		
2368	G. soja	YWS846A	CD-0571	South Korea, Gangwon	Purple	Black
2369	G. soja	YWS853A	CD-0575	South Korea, Gyeongbuk	Purple	Black
2370	G. soja	YWS857	CD-0578	South Korea, Gyeongbuk	Purple	Black
2371	G. soja	YWS860	CD-0580	South Korea, Gyeongbuk	Purple	Black
2372	G. soja	YWS862	CD-0581	South Korea, Gyeongbuk	Purple	Black

2373	G. soja	YWS863		CD-0582	South Korea, Gyeongbuk	Purple	Black	
2374	G. soja	YWS864		CD-0583	South Korea, Gyeongbuk	Purple	Black	
2375	G. soja	YWS865		CD-0584	South Korea, Gyeongbuk	Purple	Black	
2376	G. soja	YWS868		CD-0586	South Korea, Gyeongbuk	Purple	Black	
	G. soja	YWS881		CD-0595	South Korea, Gyeongnam			
2377	G. soja	YWS869		CD-0587	South Korea, Gyeongbuk	Purple	Black	
2378	G. soja	YWS872		CD-0590	South Korea, Gyeongbuk	Purple	Black	
2379	G. soja	YWS873		CD-0591	South Korea, Gyeongbuk	Purple	Black	
	G. soja	YWS874		CD-0592	South Korea, Gyeongbuk			
2380	G. soja	YWS876		CD-0593	South Korea, Gyeongbuk	Purple	Black	
2381	G. soja	YWS878		CD-0594	South Korea, Jeonnam	Purple	Black	
2382	G. soja	YWS885		CD-0596	South Korea, Gyeongnam	Purple	Black	
	G. soja	YWS887		CD-0598	South Korea, Gyeongnam			
	G. soja	YWS886		CD-0597	South Korea, Gyeongnam			
2383	G. soja	YWS891		CD-0600	South Korea, Gyeongnam	Purple	Black	
2384	G. soja	YWS896		CD-0602	South Korea, Gyeongnam	Purple	Black	Representative
2385	G. soja	YWS898		CD-0603	South Korea, Gyeongnam	Purple	Black	
2386	G. soja	YWS899		CD-0604	South Korea, Gyeongnam	Purple	Black	
2387	G. soja	YWS900		CD-0605	South Korea, Gyeongnam	Purple	Black	
2388	G. soja	YWS901		CD-0606	South Korea, Gyeongnam	Purple	Black	
2389	G. soja	YWS902		CD-0607	South Korea, Gyeongnam	Purple	Black	
2390	G. soja	YWS903		CD-0608	South Korea, Gyeongnam	Purple	Black	
2391	G. soja	YWS904		CD-0609	South Korea, Gyeongnam	Purple	Black	
2392	G. soja	YWS907		CD-0611	South Korea, Gyeongnam	Purple	Black	
2393	G. soja	YWS908		CD-0612	South Korea, Gyeongnam	Purple	Black	
	G. soja	YWS915		CD-0619	South Korea, Gyeongnam			
	G. soja	YWS906		CD-0610	South Korea, Gyeongnam			
	G. soja	YWS910		CD-0614	South Korea, Gyeongnam			
	G. soja	YWS911		CD-0615	South Korea, Gyeongnam			
	G. soja	YWS912		CD-0616	South Korea, Gyeongnam			
2394	G. soja	YWS909		CD-0613	South Korea, Gyeongnam	Purple	Black	
2395	G. soja	YWS916		CD-0620	South Korea, Gyeongnam	Purple	Black	
	G. soja	YWS928		CD-0627	South Korea, Gyeonggi			
	G. soja	YWS1346		CD-0910	South Korea, Gyeongnam			
	G. soja	YWS913		CD-0617	South Korea, Gyeongnam			
	G. soja	YWS914		CD-0618	South Korea, Gyeongnam			
2396	G. soja	YWS918		CD-0621	South Korea, Gyeonggi	Purple	Black	
	G. soja	YWS920		CD-0623	South Korea, Gyeonggi			
2397	G. soja	YWS919		CD-0622	South Korea, Gyeonggi	Purple	Black	
	G. soja	YWS921		CD-0624	South Korea, Gyeonggi			
2398	G. soja	YWS926A		CD-0626	South Korea, Gyeonggi	Purple	Black	
2399	G. soja	YWS929		CD-0628	South Korea, Gyeonggi	Purple	Black	
	G. soja	Gyeonggi hwangseong sujib	IT182955	FF-0502	South Korea, Gyeonggi			
2400	G. soja	YWS934		CD-0631	South Korea, Chungnam	Purple	Black	
2401	G. soja	YWS936		CD-0633	South Korea, Chungbuk	Purple	Black	
2402	G. soja	YWS938		CD-0634	South Korea, Gyeongbuk	Purple	Black	
	G. soja	YWS804		FF-0242	South Korea, Jeonnam			
2403	G. soja	YWS939		CD-0635	South Korea, Gyeongbuk	Purple	Black	
	G. soja	YWS930		CD-0629	South Korea, Gyeonggi			
2404	G. soja	YWS941		CD-0636	South Korea, Gyeongbuk	Purple	Black	
2405	G. soja	YWS942		CD-0637	South Korea, Gyeongbuk	Purple	Black	
2406	G. soja	YWS944		CD-0638	South Korea, Gyeongbuk	Purple	Black	
2407	G. soja	YWS948		CD-0639	South Korea, Jeonnam	Purple	Black	
2408	G. soja	YWS950		CD-0640	South Korea, Jeonnam	Purple	Black	
2409	G. soja	YWS951		CD-0641	South Korea, Jeonnam	Purple	Black	
2410	G. soja	YWS955		CD-0642	South Korea, Jeonnam	Purple	Black	
2411	G. soja	YWS956		CD-0643	South Korea, Jeonnam	Purple	Black	
2412	G. soja	YWS957		CD-0644	South Korea, Jeonnam	Purple	Black	
2413	G. soja	YWS958		CD-0645	South Korea, Jeonnam	Purple	Black	
2414	G. soja	YWS959		CD-0646	South Korea, Jeonnam	Purple	Black	
2415	G. soja	YWS961		CD-0647	South Korea, Jeonnam	Purple	Black	
2416	G. soja	YWS963		CD-0648	South Korea, Jeonnam	Purple	Black	
2417	G. soja	YWS965		CD-0650	South Korea, Chungbuk	Purple	Black	
	G. soja	YWS960		FF-0281	South Korea, Jeonnam			
2418	G. soja	YWS969		CD-0652	South Korea, Chungbuk	Purple	Black	Representative
2419	G. soja	YWS970		CD-0653	South Korea, Jeonbuk	Purple	Black	

2420	G. soja	YWS982	CD-0661	South Korea, Jeonbuk	Purple	Black	
	G. soja	YWS1010	CD-0683	South Korea, Jeonbuk			
2421	G. soja	YWS985	CD-0662	South Korea, Jeonbuk	Purple	Black	
2422	G. soja	YWS986	CD-0663	South Korea, Jeonbuk	Purple	Black	
2423	G. soja	YWS987	CD-0664	South Korea, Jeonbuk	Purple	Black	
2424	G. soja	YWS988	CD-0665	South Korea, Jeonbuk	Purple	Black	
2425	G. soja	YWS990	CD-0666	South Korea, Jeonbuk	Purple	Black	
2426	G. soja	YWS991	CD-0667	South Korea, Jeonbuk	Purple	Black	
2427	G. soja	YWS993	CD-0668	South Korea, Jeonbuk	Purple	Black	
2428	G. soja	YWS994	CD-0669	South Korea, Jeonbuk	Purple	Black	
2429	G. soja	YWS995	CD-0670	South Korea, Jeonbuk	Purple	Black	
2430	G. soja	YWS996	CD-0671	South Korea, Jeonbuk	Purple	Black	
2431	G. soja	YWS997	CD-0672	South Korea, Jeonbuk	Purple	Black	
2432	G. soja	YWS998	CD-0673	South Korea, Jeonbuk	Purple	Black	
2433	G. soja	YWS1002	CD-0675	South Korea, Jeonbuk	Purple	Black	
2434	G. soja	YWS1003	CD-0676	South Korea, Jeonbuk	Purple	Black	
2435	G. soja	YWS1004	CD-0677	South Korea, Jeonbuk	Purple	Black	
2436	G. soja	YWS1005	CD-0678	South Korea, Jeonbuk	Purple	Black	
2437	G. soja	YWS1007	CD-0680	South Korea, Jeonbuk	Purple	Black	
2438	G. soja	YWS1008	CD-0681	South Korea, Jeonbuk	Purple	Black	
2439	G. soja	YWS1012	CD-0684	South Korea, Jeonbuk	Purple	Black	
	G. soja	YWS1019	CD-0688	South Korea, Jeonbuk			
2440	G. soja	YWS1015	CD-0685	South Korea, Jeonbuk	Purple	Black	
2441	G. soja	YWS1016	CD-0686	South Korea, Jeonbuk	Purple	Black	
2442	G. soja	YWS1018	CD-0687	South Korea, Jeonbuk	Purple	Black	
	G. soja	YWS1017	FF-0298	South Korea, Jeonbuk			
2443	G. soja	YWS1020	CD-0689	South Korea, Jeonnam	Purple	Black	
2444	G. soja	YWS1021	CD-0690	South Korea, Jeonnam	Purple	Black	
2445	G. soja	YWS1022	CD-0691	South Korea, Jeonnam	Purple	Black	
2446	G. soja	YWS1023	CD-0692	South Korea, Jeonnam	Purple	Black	
2447	G. soja	YWS1025	CD-0693	South Korea, Jeonnam	Purple	Black	
2448	G. soja	YWS1026	CD-0694	South Korea, Jeonnam	Purple	Black	
2449	G. soja	YWS1027	CD-0695	South Korea, Jeonnam	Purple	Black	
2450	G. soja	YWS1028	CD-0696	South Korea, Jeonnam	Purple	Black	
	G. soja	YWS1532	CD-1012	South Korea, Jeonnam			
2451	G. soja	YWS1029	CD-0697	South Korea, Jeonnam	Purple	Black	
2452	G. soja	YWS1030	CD-0698	South Korea, Jeonnam	Purple	Black	
2453	G. soja	YWS1031	CD-0699	South Korea, Jeonnam	Purple	Black	
2454	G. soja	YWS1032	CD-0700	South Korea, Jeonnam	Purple	Black	
2455	G. soja	YWS1035	CD-0702	South Korea, Gyeongbuk	Purple	Black	
	G. soja	YWS1036	CD-0703	South Korea, Gyeongbuk			
	G. soja	YWS1037	CD-0704	South Korea, Gyeongbuk			
	G. soja	YWS1038	CD-0705	South Korea, Gyeongbuk			
2456	G. soja	YWS1039	CD-0706	South Korea, Gyeongbuk	Purple	Black	
2457	G. soja	YWS1040	CD-0707	South Korea, Gyeongbuk	Purple	Black	
	G. soja	YWS1044	CD-0710	South Korea, Gyeongbuk			
2458	G. soja	YWS1041	CD-0708	South Korea, Gyeongbuk	Purple	Black	
2459	G. soja	YWS1043	CD-0709	South Korea, Gyeongbuk	Purple	Black	
2460	G. soja	YWS1045	CD-0711	South Korea, Gyeongbuk	Purple	Black	
2461	G. soja	YWS1046	CD-0712	South Korea, Gyeongbuk	Purple	Black	
2462	G. soja	YWS1047	CD-0713	South Korea, Gyeongbuk	Purple	Black	
	G. soja	YWS1048	CD-0714	South Korea, Gyeongbuk			
	G. soja	YWS1049	CD-0715	South Korea, Gyeongbuk			
	G. soja	YWS1050	CD-0716	South Korea, Gyeongbuk			
2463	G. soja	YWS1051	CD-0717	South Korea, Gyeongbuk	Purple	Black	
	G. soja	YWS1063	CD-0726	South Korea, Gyeongbuk			
	G. soja	YWS1062	FF-0302	South Korea, Gyeongbuk			
	G. soja	YWS1052	CD-0718	South Korea, Gyeongbuk			
	G. soja	YWS1060	CD-0724	South Korea, Gyeongbuk			
	G. soja	YWS1061	CD-0725	South Korea, Gyeongbuk			
2464	G. soja	YWS1054	CD-0719	South Korea, Gyeongbuk	Purple	Black	
2465	G. soja	YWS1056	CD-0721	South Korea, Gyeongbuk	Purple	Black	
2466	G. soja	YWS1058	CD-0722	South Korea, Gyeongbuk	Purple	Black	
2467	G. soja	YWS1059	CD-0723	South Korea, Gyeongbuk	Purple	Black	Representative
2468	G. soja	YWS1065	CD-0728	South Korea, Gyeongbuk	Purple	Black	
	G. soja	YWS1070	CD-0731	South Korea, Gyeongbuk			
	G. soja	YWS1068	CD-0730	South Korea, Gyeongbuk			

2469	G. soja	YWS1066	CD-0729	South Korea, Gyeongbuk	Purple	Black	
2470	G. soja	YWS1071	CD-0732	South Korea, Gyeongbuk	Purple	Black	
2471	G. soja	YWS1074	CD-0735	South Korea, Gyeongbuk	Purple	Black	
	G. soja	YWS1072	CD-0733	South Korea, Gyeongbuk			
	G. soja	YWS1073	CD-0734	South Korea, Gyeongbuk			
2472	G. soja	YWS1076	CD-0736	South Korea, Chungnam	Purple	Black	
2473	G. soja	YWS1077	CD-0737	South Korea, Chungnam	Purple	Black	
2474	G. soja	YWS1078	CD-0738	South Korea, Chungnam	Purple	Black	
2475	G. soja	YWS1079	CD-0739	South Korea, Chungnam	Purple	Black	
2476	G. soja	YWS1080	CD-0740	South Korea, Chungnam	Purple	Black	
2477	G. soja	YWS1081	CD-0741	South Korea, Chungnam	Purple	Black	
2478	G. soja	YWS1084	CD-0742	South Korea, Chungnam	Purple	Black	
2479	G. soja	YWS1085	CD-0743	South Korea, Chungnam	Purple	Black	
2480	G. soja	YWS1087	CD-0744	South Korea, Chungnam	Purple	Black	
2481	G. soja	YWS1088	CD-0745	South Korea, Chungnam	Purple	Black	
2482	G. soja	YWS1092	CD-0747	South Korea, Chungnam	Purple	Black	
2483	G. soja	YWS1093	CD-0748	South Korea, Chungnam	Purple	Black	
2484	G. soja	YWS1094	CD-0749	South Korea, Chungnam	Purple	Black	
2485	G. soja	YWS1095	CD-0750	South Korea, Chungnam	Purple	Black	
2486	G. soja	YWS1096	CD-0751	South Korea, Chungnam	Purple	Black	
2487	G. soja	YWS1097	CD-0752	South Korea, Chungnam	Purple	Black	
2488	G. soja	YWS1098	CD-0753	South Korea, Chungnam	Purple	Black	
2489	G. soja	YWS1099	CD-0754	South Korea, Chungnam	Purple	Black	
2490	G. soja	YWS1100	CD-0755	South Korea, Chungnam	Purple	Black	
2491	G. soja	YWS1101	CD-0756	South Korea, Chungnam	Purple	Black	
2492	G. soja	YWS1102	CD-0757	South Korea, Chungnam	Purple	Black	
2493	G. soja	YWS1104	CD-0758	South Korea, Gangwon	Purple	Black	
2494	G. soja	YWS1107	CD-0759	South Korea, Gangwon	Purple	Black	
2495	G. soja	YWS1109	CD-0760	South Korea, Gangwon	Purple	Black	
2496	G. soja	YWS1110	CD-0761	South Korea, Gangwon	Purple	Black	
2497	G. soja	YWS1112	CD-0762	South Korea, Gangwon	Purple	Black	
2498	G. soja	YWS1114	CD-0763	South Korea, Gangwon	Purple	Black	Representative
2499	G. soja	YWS1116	CD-0765	South Korea, Gangwon	Purple	Black	
2500	G. soja	YWS1119	CD-0768	South Korea, Gangwon	Purple	Black	
	G. soja	YWS1117	CD-0766	South Korea, Gangwon			
2501	G. soja	YWS1120	CD-0769	South Korea, Gangwon	Purple	Black	
	G. soja	YWS1127	CD-0774	South Korea, Gangwon			
	G. soja	YWS1204	CD-0820	South Korea, Chungbuk			
2502	G. soja	YWS1121	CD-0770	South Korea, Gangwon	Purple	Black	
	G. soja	YWS1193	CD-0812	South Korea, Chungbuk			
2503	G. soja	YWS1123	CD-0771	South Korea, Gangwon	Purple	Black	
	G. soja	YWS1210	CD-0825	South Korea, Chungbuk			
	G. soja	YWS1192	CD-0811	South Korea, Chungbuk			
2504	G. soja	YWS1124	CD-0772	South Korea, Gangwon	Purple	Black	
2505	G. soja	YWS1129	CD-0775	South Korea, Gyeongbuk	Purple	Black	
2506	G. soja	YWS1132	CD-0776	South Korea, Gyeongbuk	Purple	Black	Representative
2507	G. soja	YWS1150	CD-0778	South Korea, Chungnam	Purple	Black	
2508	G. soja	YWS1151	CD-0779	South Korea, Chungnam	Purple	Black	
2509	G. soja	YWS1153	CD-0781	South Korea, Chungnam	Purple	Black	
2510	G. soja	YWS1155	CD-0782	South Korea, Chungnam	Purple	Black	
2511	G. soja	YWS1158	CD-0783	South Korea, Chungnam	Purple	Black	
2512	G. soja	YWS1160	CD-0785	South Korea, Chungnam	Purple	Black	
2513	G. soja	YWS1162	CD-0786	South Korea, Chungnam	Purple	Black	
2514	G. soja	YWS1167	CD-0787	South Korea, Chungnam	Purple	Black	
2515	G. soja	YWS1168	CD-0788	South Korea, Chungnam	Purple	Black	
2516	G. soja	YWS1169	CD-0789	South Korea, Chungnam	Purple	Black	
2517	G. soja	YWS1170	CD-0790	South Korea, Chungnam	Purple	Black	
2518	G. soja	YWS1171	CD-0791	South Korea, Chungnam	Purple	Black	
2519	G. soja	YWS1173	CD-0793	South Korea, Chungnam	Purple	Black	
2520	G. soja	YWS1175	CD-0794	South Korea, Chungnam	Purple	Black	
2521	G. soja	YWS1176	CD-0795	South Korea, Chungnam	Purple	Black	
2522	G. soja	YWS1177	CD-0796	South Korea, Chungnam	Purple	Black	
2523	G. soja	YWS1179	CD-0798	South Korea, Chungnam	Purple	Black	Representative
2524	G. soja	YWS1180	CD-0799	South Korea, Chungnam	Purple	Black	
2525	G. soja	YWS1181	CD-0800	South Korea, Chungbuk	Purple	Black	
	G. soja	YWS1182	CD-0801	South Korea, Chungbuk			
	G. soja	YWS1183	CD-0802	South Korea, Chungbuk			

	G. soja	YWS1184	CD-0803	South Korea, Chungbuk			
2526	G. soja	YWS1185	CD-0804	South Korea, Chungbuk	Purple	Black	
	G. soja	YWS1191	CD-0810	South Korea, Chungbuk			
	G. soja	YWS1195	CD-0814	South Korea, Chungbuk			
	G. soja	YWS1186	CD-0805	South Korea, Chungbuk			
	G. soja	YWS1188	CD-0807	South Korea, Chungbuk			
	G. soja	YWS1189	CD-0808	South Korea, Chungbuk			
2527	G. soja	YWS1187	CD-0806	South Korea, Chungbuk	Purple	Black	
2528	G. soja	YWS1190	CD-0809	South Korea, Chungbuk	Purple	Black	
2529	G. soja	YWS1196	CD-0815	South Korea, Chungbuk	Purple	Black	
2530	G. soja	YWS1197	CD-0816	South Korea, Chungbuk	Purple	Black	
2531	G. soja	YWS1198	CD-0817	South Korea, Chungbuk	Purple	Black	
2532	G. soja	YWS1199	CD-0818	South Korea, Chungbuk	Purple	Black	
2533	G. soja	YWS1200	CD-0819	South Korea, Chungbuk	Purple	Black	
2534	G. soja	YWS1206	CD-0821	South Korea, Chungbuk	Purple	Black	
2535	G. soja	YWS1207	CD-0822	South Korea, Chungbuk	Purple	Black	
2536	G. soja	YWS1208	CD-0823	South Korea, Chungbuk	Purple	Black	
2537	G. soja	YWS1209	CD-0824	South Korea, Chungbuk	Purple	Black	
	G. soja	YWS1118	CD-0767	South Korea, Gangwon			
2538	G. soja	YWS1214	CD-0827	South Korea, Chungbuk	Purple	Black	
2539	G. soja	YWS1216	CD-0828	South Korea, Chungbuk	Purple	Black	
	G. soja	YWS1194	CD-0813	South Korea, Chungbuk			
	G. soja	YWS1211	CD-0826	South Korea, Chungbuk			
2540	G. soja	YWS1217	CD-0829	South Korea, Gyeongnam	Purple	Black	
	G. soja	YWS1398	FF-0369	South Korea, Chungbuk			
2541	G. soja	YWS1218	CD-0830	South Korea, Gyeongnam	Purple	Black	Representative
2542	G. soja	YWS1221	CD-0831	South Korea, Gyeongnam	Purple	Black	
2543	G. soja	YWS1224	CD-0832	South Korea, Gyeongnam	Purple	Black	
2544	G. soja	YWS1225	CD-0833	South Korea, Gyeongnam	Purple	Black	
2545	G. soja	YWS1229	CD-0835	South Korea, Gyeongnam	Purple	Black	
2546	G. soja	YWS1230	CD-0836	South Korea, Gyeongnam	Purple	Black	
2547	G. soja	YWS1231	CD-0837	South Korea, Gyeongnam	Purple	Black	
2548	G. soja	YWS1232	CD-0838	South Korea, Gyeongnam	Purple	Black	
2549	G. soja	YWS1233	CD-0839	South Korea, Gyeongnam	Purple	Black	
2550	G. soja	YWS1234	CD-0840	South Korea, Gyeongnam	Purple	Black	
2551	G. soja	YWS1236	CD-0841	South Korea, Gyeongnam	Purple	Black	
2552	G. soja	YWS1237	CD-0842	South Korea, Gyeongnam	Purple	Black	
2553	G. soja	YWS1239	CD-0843	South Korea, Gyeongnam	Purple	Black	Representative
2554	G. soja	YWS1241	CD-0844	South Korea, Gyeongnam	Purple	Black	
2555	G. soja	YWS1247	CD-0849	South Korea, Gyeongnam	Purple	Black	
2556	G. soja	YWS1249	CD-0850	South Korea, Jeonnam	Purple	Black	
2557	G. soja	YWS1251	CD-0851	South Korea, Jeonnam	Purple	Black	
2558	G. soja	YWS1253	CD-0852	South Korea, Jeonnam	Purple	Black	
2559	G. soja	YWS1256	CD-0853	South Korea, Jeonbuk	Purple	Black	
2560	G. soja	YWS1257	CD-0854	South Korea, Jeonbuk	Purple	Black	
2561	G. soja	YWS1258	CD-0855	South Korea, Jeonbuk	Purple	Black	
2562	G. soja	YWS1259	CD-0856	South Korea, Jeonbuk	Purple	Black	
2563	G. soja	YWS1260	CD-0857	South Korea, Jeonbuk	Purple	Black	
2564	G. soja	YWS1262	CD-0859	South Korea, Chungnam	Purple	Black	
2565	G. soja	YWS1263	CD-0860	South Korea, Chungnam	Purple	Black	
2566	G. soja	YWS1265	CD-0861	South Korea, Chungnam	Purple	Black	
2567	G. soja	YWS1266	CD-0862	South Korea, Chungnam	Purple	Black	
2568	G. soja	YWS1268	CD-0863	South Korea, Chungnam	Purple	Black	
2569	G. soja	YWS1271	CD-0865	South Korea, Gyeonggi	Purple	Black	
	G. soja	YWS1272	CD-0866	South Korea, Gyeonggi			
2570	G. soja	YWS1273	CD-0867	South Korea, Gyeonggi	Purple	Black	
2571	G. soja	YWS1274	CD-0868	South Korea, Gyeonggi	Purple	Black	
2572	G. soja	YWS1276	CD-0869	South Korea, Gyeonggi	Purple	Black	
2573	G. soja	YWS1277	CD-0870	South Korea, Gyeonggi	Purple	Black	
2574	G. soja	YWS1279	CD-0871	South Korea, Gyeonggi	Purple	Black	
2575	G. soja	YWS1280	CD-0872	South Korea, Gyeonggi	Purple	Black	
	G. soja	YWS1303	CD-0889	South Korea, Gyeonggi			
2576	G. soja	YWS1281	CD-0873	South Korea, Gyeonggi	Purple	Black	
2577	G. soja	YWS1282	CD-0874	South Korea, Gyeonggi	Purple	Black	
2578	G. soja	YWS1284	CD-0875	South Korea, Gyeonggi	Purple	Black	
2579	G. soja	YWS1285	CD-0876	South Korea, Gyeonggi	Purple	Black	
2580	G. soja	YWS1287	CD-0877	South Korea, Gyeonggi	Purple	Black	

2581	G. soja	YWS1288	CD-0878	South Korea, Gyeonggi	Purple	Black	
2582	G. soja	YWS1289	CD-0879	South Korea, Gyeonggi	Purple	Black	
	G. soja	YWS1299	CD-0887	South Korea, Chungbuk			
2583	G. soja	YWS1290	CD-0880	South Korea, Gyeonggi	Purple	Black	
2584	G. soja	YWS1293	CD-0881	South Korea, Gyeonggi	Purple	Black	
2585	G. soja	YWS1294	CD-0882	South Korea, Gyeonggi	Purple	Black	
2586	G. soja	YWS1295	CD-0883	South Korea, Gyeonggi	Purple	Black	
2587	G. soja	YWS1296	CD-0884	South Korea, Gyeonggi	Purple	Black	
2588	G. soja	YWS1297	CD-0885	South Korea, Chungbuk	Purple	Black	
2589	G. soja	YWS1298	CD-0886	South Korea, Chungbuk	Purple	Black	
2590	G. soja	YWS1300	CD-0888	South Korea, Chungbuk	Purple	Black	
2591	G. soja	YWS1304	CD-0890	South Korea, Gyeonggi	Purple	Black	
2592	G. soja	YWS1305	CD-0891	South Korea, Gyeonggi	Purple	Black	
2593	G. soja	YWS1306	CD-0892	South Korea, Chungbuk	Purple	Black	
2594	G. soja	YWS1307	CD-0893	South Korea, Chungbuk	Purple	Black	
2595	G. soja	YWS1308	CD-0894	South Korea, Gyeonggi	Purple	Black	
2596	G. soja	YWS1325	CD-0896	South Korea, Gyeongnam	Purple	Black	
2597	G. soja	YWS1327	CD-0897	South Korea, Gyeongnam	Purple	Black	
2598	G. soja	YWS1328	CD-0898	South Korea, Gyeongnam	Purple	Black	
2599	G. soja	YWS1329	CD-0899	South Korea, Gyeongnam	Purple	Black	
2600	G. soja	YWS1332	CD-0900	South Korea, Gyeongnam	Purple	Black	
	G. soja	YWS1333	CD-0901	South Korea, Gyeongnam			
2601	G. soja	YWS1334	CD-0902	South Korea, Gyeongnam	Purple	Black	
2602	G. soja	YWS1336	CD-0903	South Korea, Gyeongnam	Purple	Black	
2603	G. soja	YWS1337	CD-0904	South Korea, Gyeongbuk	Purple	Black	
2604	G. soja	YWS1338	CD-0905	South Korea, Gyeongnam	Purple	Black	
2605	G. soja	YWS1339	CD-0906	South Korea, Gyeongnam	Purple	Black	
2606	G. soja	YWS1341	CD-0907	South Korea, Gyeongnam	Purple	Black	Representative
2607	G. soja	YWS1342	CD-0908	South Korea, Gyeongnam	Purple	Black	
2608	G. soja	YWS1343	CD-0909	South Korea, Gyeongnam	Purple	Black	
2609	G. soja	YWS1347	CD-0911	South Korea, Gyeongnam	Purple	Black	
2610	G. soja	YWS1348	CD-0912	South Korea, Gyeongnam	Purple	Black	
2611	G. soja	YWS1349	CD-0913	South Korea, Gyeongnam	Purple	Black	
2612	G. soja	YWS1351	CD-0914	South Korea, Gyeongnam	Purple	Black	
2613	G. soja	YWS1352	CD-0915	South Korea, Gyeongnam	Purple	Black	
2614	G. soja	YWS1356	CD-0916	South Korea, Gyeongbuk	Purple	Black	
2615	G. soja	YWS1358	CD-0917	South Korea, Gyeongbuk	Purple	Black	
2616	G. soja	YWS1361	CD-0918	South Korea, Gyeongbuk	Purple	Black	
2617	G. soja	YWS1362	CD-0919	South Korea, Gyeongbuk	Purple	Black	
2618	G. soja	YWS1363	CD-0920	South Korea, Gyeongbuk	Purple	Black	
2619	G. soja	YWS1366	CD-0921	South Korea, Gyeongbuk	Purple	Black	
2620	G. soja	YWS1367	CD-0922	South Korea, Gyeongbuk	Purple	Black	
2621	G. soja	YWS1368	CD-0923	South Korea, Gyeongbuk	Purple	Black	
2622	G. soja	YWS1370	CD-0924	South Korea, Gyeongbuk	Purple	Black	
2623	G. soja	YWS1371	CD-0925	South Korea, Gyeongbuk	Purple	Black	
2624	G. soja	YWS1373	CD-0926	South Korea, Gyeongbuk	Purple	Black	
2625	G. soja	YWS1374	CD-0927	South Korea, Gyeongbuk	Purple	Black	
2626	G. soja	YWS1375	CD-0928	South Korea, Gyeongbuk	Purple	Black	
2627	G. soja	YWS1376	CD-0929	South Korea, Gyeongbuk	Purple	Black	
2628	G. soja	YWS1379	CD-0931	South Korea, Gyeongbuk	Purple	Black	
2629	G. soja	YWS1380	CD-0932	South Korea, Gyeongbuk	Purple	Black	
2630	G. soja	YWS1381	CD-0933	South Korea, Gyeongbuk	Purple	Black	
	G. soja	YWS817	CD-0549	South Korea, Jeonnam			
	G. soja	YWS1382	CD-0934	South Korea, Gyeongbuk			
	G. soja	YWS815	CD-0547	South Korea, Jeonnam			
	G. soja	YWS816	CD-0548	South Korea, Jeonnam			
2631	G. soja	YWS1384	CD-0935	South Korea, Gyeonggi	Purple	Black	
2632	G. soja	YWS1386	CD-0936	South Korea, Gyeonggi	Purple	Black	
2633	G. soja	YWS1387	CD-0937	South Korea, Chungnam	Purple	Black	
2634	G. soja	YWS1389	CD-0938	South Korea, Chungbuk	Purple	Black	
2635	G. soja	YWS1396	CD-0940	South Korea, Chungbuk	Purple	Black	
2636	G. soja	YWS1399	CD-0941	South Korea, Chungbuk	Purple	Black	
2637	G. soja	YWS1400	CD-0942	South Korea, Chungbuk	Purple	Black	
2638	G. soja	YWS1401	CD-0943	South Korea, Chungbuk	Purple	Black	
2639	G. soja	YWS1402	CD-0944	South Korea, Chungbuk	Purple	Black	
2640	G. soja	YWS1403	CD-0945	South Korea, Chungbuk	Purple	Black	
2641	G. soja	YWS1404	CD-0946	South Korea, Chungbuk	Purple	Black	

	G. soja	YWS1415	CD-0953	South Korea, Jeonnam			
2642	G. soja	YWS1406	CD-0947	South Korea, Chungbuk	Purple	Black	
2643	G. soja	YWS1407	CD-0948	South Korea, Chungbuk	Purple	Black	
2644	G. soja	YWS1410	CD-0949	South Korea, Jeonnam	Purple	Black	
2645	G. soja	YWS1411	CD-0950	South Korea, Jeonnam	Purple	Black	
2646	G. soja	YWS1412	CD-0951	South Korea, Jeonnam	Purple	Black	Representative
	G. soja	YWS1413	FF-0373	South Korea, Jeonnam			
2647	G. soja	YWS1414	CD-0952	South Korea, Jeonnam	Purple	Black	
2648	G. soja	YWS1416	CD-0954	South Korea, Jeonnam	Purple	Black	
2649	G. soja	YWS1420	CD-0956	South Korea, Jeonnam	Purple	Black	Representative
2650	G. soja	YWS1437	CD-0957	South Korea, Gyeongnam	Purple	Black	
2651	G. soja	YWS1440	CD-0959	South Korea, Gyeongnam	Purple	Black	
2652	G. soja	YWS1441	CD-0960	South Korea, Gyeongnam	Purple	Black	
2653	G. soja	YWS1446	CD-0961	South Korea, Gyeongnam	White	Black	
2654	G. soja	YWS1447	CD-0962	South Korea, Gyeongnam	Purple	Black	
2655	G. soja	YWS1449	CD-0963	South Korea, Gyeongnam	Purple	Black	
2656	G. soja	YWS1451	CD-0964	South Korea, Gyeongnam	Purple	Black	
2657	G. soja	YWS1453	CD-0966	South Korea, Gyeongnam	Purple	Black	
2658	G. soja	YWS1454	CD-0967	South Korea, Gyeongnam	Purple	Black	
2659	G. soja	YWS1458	CD-0969	South Korea, Gyeongbuk	Purple	Black	
2660	G. soja	YWS1459	CD-0970	South Korea, Gyeongbuk	Purple	Black	
2661	G. soja	YWS1460	CD-0971	South Korea, Gyeongbuk	Purple	Black	
2662	G. soja	YWS1461	CD-0972	South Korea, Gyeongbuk	Purple	Black	
2663	G. soja	YWS1462	CD-0973	South Korea, Gyeongbuk	Purple	Black	
2664	G. soja	YWS1465	CD-0974	South Korea, Gyeongbuk	Purple	Black	
2665	G. soja	YWS1466	CD-0975	South Korea, Gyeongbuk	Purple	Black	
2666	G. soja	YWS1469	CD-0976	South Korea, Gyeongbuk	Purple	Black	
2667	G. soja	YWS1470	CD-0977	South Korea, Gyeongbuk	Purple	Black	
2668	G. soja	YWS1471	CD-0978	South Korea, Gyeongbuk	Purple	Black	
2669	G. soja	YWS1473	CD-0980	South Korea, Gyeongbuk	Purple	Black	
	G. soja	YWS1474	CD-0981	South Korea, Gyeongbuk			
2670	G. soja	YWS1475	CD-0982	South Korea, Gyeongbuk	Purple	Black	
2671	G. soja	YWS1478	CD-0983	South Korea, Gyeongbuk	Purple	Black	
2672	G. soja	YWS1479	CD-0984	South Korea, Gyeongbuk	Purple	Black	
2673	G. soja	YWS1481	CD-0985	South Korea, Gyeongbuk	Purple	Black	
2674	G. soja	YWS1482	CD-0986	South Korea, Gyeongbuk	Purple	Black	
2675	G. soja	YWS1495	CD-0987	South Korea, Gyeongbuk	Purple	Black	
2676	G. soja	YWS1501	CD-0988	South Korea, Gyeonggi	Purple	Black	
2677	G. soja	YWS1503	CD-0989	South Korea, Gyeonggi	Purple	Black	
2678	G. soja	YWS1506	CD-0992	South Korea, Gyeongnam	Purple	Black	
2679	G. soja	YWS1507	CD-0993	South Korea, Gyeongnam	Purple	Black	
2680	G. soja	YWS1508	CD-0994	South Korea, Gyeongnam	Purple	Black	
2681	G. soja	YWS1509	CD-0995	South Korea, Gyeongnam	Purple	Black	
2682	G. soja	YWS1510	CD-0996	South Korea, Gyeongnam	Purple	Black	
2683	G. soja	YWS1511	CD-0997	South Korea, Gyeongnam	Purple	Black	
2684	G. soja	YWS1512	CD-0998	South Korea, Gyeongnam	Purple	Black	
2685	G. soja	YWS1513	CD-0999	South Korea, Gyeongnam	Purple	Black	
2686	G. soja	YWS1514	CD-1000	South Korea, Gyeongnam	Purple	Black	
2687	G. soja	YWS1515	CD-1001	South Korea, Gyeongnam	Purple	Black	
2688	G. soja	YWS1517	CD-1003	South Korea, Gyeongnam	Purple	Black	
2689	G. soja	YWS1518	CD-1004	South Korea, Gyeongnam	Purple	Black	
2690	G. soja	YWS1519	CD-1005	South Korea, Gyeongnam	Purple	Black	
2691	G. soja	YWS1520	CD-1006	South Korea, Gyeongnam	Purple	Black	
2692	G. soja	YWS1521	CD-1007	South Korea, Gyeongbuk	Purple	Black	
2693	G. soja	YWS1529	CD-1009	South Korea, Jeonnam	Purple	Black	
2694	G. soja	YWS1530	CD-1010	South Korea, Jeonnam	Purple	Black	
2695	G. soja	YWS1531	CD-1011	South Korea, Jeonnam	Purple	Black	
2696	G. soja	YWS1533	CD-1013	South Korea, Jeonnam	Purple	Black	
2697	G. soja	YWS1539	CD-1014	South Korea, Jeonnam	Purple	Black	
2698	G. soja	YWS1540	CD-1015	South Korea, Jeonnam	Purple	Black	
2699	G. soja	YWS1542	CD-1017	South Korea, Jeonnam	Purple	Black	
2700	G. soja	YWS1543	CD-1018	South Korea, Jeonnam	Purple	Black	
2701	G. soja	YWS1544	CD-1019	South Korea, Jeonnam	Purple	Black	
2702	G. soja	YWS1546	CD-1020	South Korea, Jeonnam	Purple	Black	
2703	G. soja	YWS1547	CD-1021	South Korea, Jeonnam	Purple	Black	
2704	G. soja	YWS1548	CD-1022	South Korea, Jeonnam	Purple	Black	
2705	G. soja	YWS1549	CD-1023	South Korea, Jeonnam	Purple	Black	

2706	G. soja	YWS1550		CD-1024	South Korea, Jeonnam	Purple	Black	
2707	G. soja	YWS1551		CD-1025	South Korea, Chungnam	Purple	Black	
2708	G. soja	YWS1552		CD-1026	South Korea, Chungnam	Purple	Black	
2709	G. soja	YWS1554		CD-1028	South Korea, Chungnam	Purple	Black	
2710	G. soja	YWS1556		CD-1029	South Korea, Chungnam	Purple	Black	
2711	G. soja	YWS1557		CD-1030	South Korea, Chungnam	Purple	Black	
2712	G. soja	YWS1559		CD-1032	South Korea, Chungnam	Purple	Black	
2713	G. soja	YWS1560		CD-1033	South Korea, Chungnam	Purple	Black	
2714	G. soja	YWS1561		CD-1034	South Korea, Chungnam	Purple	Black	
2715	G. soja	YWS1562		CD-1035	South Korea, Chungnam	Purple	Black	
2716	G. soja	YWS1567		CD-1036	South Korea, Gyeongbuk	Purple	Black	
2717	G. soja	YWS1569		CD-1038	South Korea, Gyeongbuk	Purple	Black	Representative
2718	G. soja	YWS1578		CD-1042	South Korea, Jeonnam	Purple	Black	
2719	G. soja	YWS1579		CD-1043	South Korea, Jeonnam	Purple	Black	
2720	G. soja	YWS1580		CD-1044	South Korea, Jeonnam	Purple	Black	
2721	G. soja	YWS1581		CD-1045	South Korea, Jeonnam	Purple	Black	
2722	G. soja	YWS1584		CD-1047	South Korea, Jeonnam	Purple	Black	
2723	G. soja	YWS1585		CD-1048	South Korea, Jeonnam	Purple	Black	
	G. soja	YWS1408		FF-0371	South Korea, Chungbuk			
2724	G. soja	YWS1586		CD-1049	South Korea, Jeonnam	Purple	Black	
2725	G. soja	YWS1590		CD-1050	South Korea, Jeonnam	Purple	Black	
2726	G. soja	YWS1591		CD-1051	South Korea, Jeonbuk	Purple	Black	
2727	G. soja	YWS1592		CD-1052	South Korea, Jeonbuk	Purple	Black	
2728	G. soja	YWS1593		CD-1053	South Korea, Jeonbuk	Purple	Black	
2729	G. soja	YWS1594		CD-1054	South Korea, Jeonbuk	Purple	Black	
2730	G. soja	YWS1596		CD-1055	South Korea, Jeonbuk	Purple	Black	
2731	G. soja	YWS1597		CD-1056	South Korea, Jeonbuk	Purple	Black	
2732	G. soja	YWS1598		CD-1057	South Korea, Jeonbuk	Purple	Black	
2733	G. soja	YWS1599		CD-1058	South Korea, Jeonbuk	Purple	Black	
2734	G. soja	YWS1600		CD-1059	South Korea, Jeonbuk	Purple	Black	
2735	G. soja	YWS1601		CD-1060	South Korea, Jeonbuk	Purple	Black	
2736	G. soja	YWS1605		CD-1061	South Korea, Jeonbuk	Purple	Black	
2737	G. soja	YWS1606		CD-1062	South Korea, Jeonbuk	Purple	Black	
2738	G. soja	YWS1607		CD-1063	South Korea, Jeonbuk	Purple	Black	
2739	G. soja	YWS1608		CD-1064	South Korea, Jeonbuk	Purple	Black	
2740	G. soja	YWS1609		CD-1065	South Korea, Jeonbuk	Purple	Black	
2741	G. soja	YWS1610		CD-1066	South Korea, Jeonbuk	Purple	Black	
2742	G. soja	YWS1611		CD-1067	South Korea, Jeonbuk	Purple	Black	
2743	G. soja	YWS1612		CD-1068	South Korea, Jeonbuk	Purple	Black	
2744	G. soja	YWS1613		CD-1069	South Korea, Jeonbuk	Purple	Black	
2745	G. soja	YWS1614		CD-1070	South Korea, Jeonbuk	Purple	Black	
2746	G. soja	YWS1615		CD-1071	South Korea, Jeju	Purple	Black	
2747	G. soja	YWS1616		CD-1072	South Korea, Jeju	Purple	Black	Representative
2748	G. soja	YWS1617		CD-1073	South Korea, Jeju	Purple	Black	
2749	G. soja	YWS1618		CD-1074	South Korea, Jeju	Purple	Black	
2750	G. soja	YWS1619		CD-1075	South Korea, Jeonbuk	Purple	Black	
2751	G. soja	YWS1620		CD-1076	South Korea, Jeonnam	Purple	Black	
2752	G. soja	B01133	JPN4	CD-1079	Japan, Hokkaido	Purple	Black	Representative
2753	G. soja	B01156	JPN9	CD-1081	Japan, Hokkaido	Purple	Black	Representative
2754	G. soja	B01164	JPN10	CD-1082	Japan, Hokkaido	Purple	Black	Representative
2755	G. soja	B02039	JPN11	CD-1083	Japan, Anomori	Purple	Black	Representative
2756	G. soja	B03016	JPN21	CD-1085	Japan, Niigata	Purple	Black	Representative
2757	G. soja	B03028	JPN24	CD-1087	Japan, Toyama	Purple	Black	Representative
2758	G. soja	B03046	JPN29	CD-1088	Japan, Fukui	Purple	Black	Representative
2759	G. soja	B04057	JPN32	CD-1089	Japan, Nagano	Purple	Black	Representative
	G. soja	B04065	JPN33	FF-0427	Japan, Nagano			
2760	G. soja	B04118	JPN36	CD-1090	Japan, Chiba	Purple	Black	Representative
2761	G. soja	B05049	JPN41	CD-1093	Japan, Mie	Purple	Black	Representative
2762	G. soja	B05053	JPN43	CD-1095	Japan, Mie	Purple	Black	Representative
2763	G. soja	B06033	JPN46	CD-1096	Japan, Hyogo	Purple	Black	Representative
2764	G. soja	B06037	JPN47	CD-1097	Japan, Kyoto	Purple	Black	Representative
2765	G. soja	B06103	JPN51	CD-1098	Japan, Hyogo	Purple	Black	Representative
2766	G. soja	B07083	JPN53	CD-1099	Japan, Kochi	Purple	Black	Representative
2767	G. soja	B07145	JPN55	CD-1101	Japan, Kochi	Purple	Black	Representative
2768	G. soja	B07162	JPN56	CD-1102	Japan, Ehime	Purple	Black	Representative
	G. soja	B07126	JPN54	CD-1100	Japan, Ehime			
2769	G. soja	B08038	JPN59	CD-1103	Japan, Yamaguchi	Purple	Black	Representative

2770	G. soja	B08041	JPN61	CD-1104	Japan, Yamaguchi	Purple	Black	Representative
2771	G. soja	B08055	JPN63	CD-1105	Japan, Shimane	Purple	Black	Representative
2772	G. soja	B08062	JPN64	CD-1106	Japan, Okayama	Purple	Black	Representative
2773	G. soja	B09014	JPN65	CD-1107	Japan, Hokkaido	Purple	Black	Representative
2774	G. soja	B09058	JPN66	CD-1108	Japan, Miyazaki	Purple	Black	Representative
2775	G. soja	B09107	JPN72	CD-1111	Japan, Saga	Purple	Black	Representative
2776	G. soja	B09115	JPN74	CD-1112	Japan, Nagashaki	Purple	Black	Representative
2777	G. soja	PI 597448 B	Jiamusi-1, CHN9	CD-1117	China, Heilongjiang	Purple	Black	Representative
2778	G. soja	PI 597448 D	Jiamusi-1, CHN10	CD-1118	China, Heilongjiang	Purple	Black	Representative
2779	G. soja	PI 464935	81-200002, CHN20	CD-1126	China, Jiangsu	Purple	Black	Representative
2780	G. soja	PI 532449	GD50003-2, CHN27	CD-1130	China, Jilin	Purple	Black	Representative
2781	G. soja	PI 440913 B	CHN28	CD-1131	China, Jilin	Purple	Black	Representative
2782	G. soja	PI 464891 B	Gong di No. 2022, CHN29	CD-1132	China, Jilin	Purple	Black	Representative
2783	G. soja	PI 532452 B	GD50549, CHN30	CD-1133	China, Jilin	Purple	Black	Representative
2784	G. soja	PI 549032	ZYDO 2632, CHN32	CD-1135	China, Liaoning	Purple	Black	Representative
2785	G. soja	PI 447003 B	CHN37	CD-1139	China, Nei Monggol	Purple	Black	Representative
2786	G. soja	PI 468400 B	CHN38	CD-1140	China, Ningxia	Purple	Black	Representative
2787	G. soja	PI 483465	CHN41	CD-1141	China, Shaanxi	Purple	Black	Representative
2788	G. soja	PI 597459 D	ZYD3234, CHN46	CD-1145	China, Shandong	Purple	Black	Representative
2789	G. soja	PI 378685		CD-1158	Japan, Ehime	Purple	Black	Representative
2790	G. soja	PI 407288		CD-1162	China, Jilin	Purple	Black	Representative
2791	G. soja	PI 407296		CD-1163	China, Liaoning	Purple	Black	Representative
2792	G. soja	PI 522180	ZYD 68, CHN7	CD-1115	China, Heilongjiang	Purple	Black	Representative
	G. soja	PI 464928	LS-008, CHN31	CD-1134	China, Liaoning			
	G. soja	PI 464869 B	L 79-1809, CHN14	CD-1121	China, Heilongjiang			
2793	G. soja	PI 464866 A	L 79-0009, CHN13	CD-1120	China, Heilongjiang	Purple	Black	Representative
	G. soja	PI 464925 A	LS-001, CHN33	CD-1136	China, Liaoning			
	G. soja	PI 522183 B	ZYD 403, CHN15	CD-1122	China, Heilongjiang			
2794	G. soja	PI 483468 B	CHN17	CD-1123	China, Henan	Purple	Black	Representative
	G. soja	PI 483467	CHN16	FF-0446	China, Henan			
	G. soja	PI 483468 A	CHN18	CD-1124	China, Henan			
2795	G. soja	PI 464934	81-200001, CHN19	CD-1125	China, Jiangsu	Purple	Black	Representative
	G. soja	PI 464936 A	81-200004, CHN21	CD-1127	China, Jiangsu			
2796	G. soja	PI 464937 B	81-200014, CHN23	CD-1128	China, Jiangsu	Purple	Black	Representative
2797	G. soja	PI 464939 A	81-200027, CHN24	CD-1129	China, Jiangsu	Purple	Black	Representative
	G. soja	PI 464939 B	81-200027, CHN25	FF-0448	China, Jiangsu			
2798	G. soja	PI 483460 C	CCHN35	CD-1137	China, Liaoning	Purple	Black	Representative
2799	G. soja	PI 549046	ZYDO 3728, CHN42	CD-1142	China, Shaanxi	Purple	Black	Representative
2800	G. soja	PI 597458 C	ZYD3233, CHN45	CD-1144	China, Shandong	Purple	Black	Representative
2801	G. soja	PI 407305	CHN49	CD-1147	China, Shanghai	Purple	Black	Representative
	G. soja	PI 407306	CHN50	FF-0455	China, Shanghai			
	G. soja	PI 407307	CHN51	FF-0456	China, Shanghai			
2802	G. soja	PI 597455	ZYD3024, CHN53	CD-1148	China, Shaanxi	Purple	Black	Representative
2803	G. soja	PI 597456	ZYD3036, CHN54	CD-1149	China, Shaanxi	Purple	Black	Representative
2804	G. soja	PI 597454 B	ZYD3015, CHN56	CD-1151	China, Shaanxi	Purple	Yellow	Representative
2805	G. soja	PI 468396 A	CHN58	CD-1153	China, Shaanxi	Purple	Black	Representative
2806	G. soja	PI 468398 C	CHN60	CD-1155	China, Shaanxi	Purple	Black	Representative
	G. soja	PI 468398 A	CHN59	CD-1154	China, Shaanxi			
2807	G. soja	PI 407300	CHN61	CD-1156	China, Zhejiang	Purple	Black	Representative
	G. soja	PI 407303	CHN64	FF-0460	China, Zhejiang			
2808	G. soja	PI 65549		CD-1160	China, Heilongjiang	Purple	Black	Representative
	G. soja	PI 135624		CD-1161	China, Heilongjiang			

2809	G. soja	B01042	JPN1	CD-1078	Japan, Hokkaido	Purple	Black	Representative
2810	G. soja	B02206	JPN18	CD-1084	Japan, Akita	Purple	Black	Representative
2811	G. soja	B04134	JPN38	CD-1091	Japan, Yamanashi	Purple	Black	Representative
2812	G. soja	B05047	JPN40	CD-1092	Japan, Gifu	Purple	Black	Representative
2813	G. soja	B05051	JPN42	CD-1094	Japan, Mie	White	Black	Representative
2814	G. soja	B09089	JPN70	CD-1109	Japan, Oita	Purple	Black	Representative
2815	G. soja	PI 378689		CD-1159	Japan, Niigata	Purple	Black	Representative
2816	G. soja	PI 458537 A		PI458537A-1	China, Heilongjiang	Purple	Black	Representative
	G. soja	PI 458537 B	CHN12	CD-1119	China, Heilongjiang			
	G. soja	PI 522184	ZYD 512, CHN8	CD-1116	China, Heilongjiang			
2817	G. soja	PI 507822		PI507822-2	Russia, Amur	Purple	Black	Representative
	G. soja	PI 507839		PI507839-1	South Korea,			
	G. soja	PI 424032-1		PI424032-1	,			Seeds mislabelled
2818	G. soja	YWS 7		FF-0001	South Korea, Gyeongnam	Purple	Black	
	G. soja	YWS 12		CD-0010	South Korea, Gyeongbuk			
2819	G. soja	YWS 25		FF-0003	South Korea, Gyeongnam	Purple	Black	
2820	G. soja	YWS 26		FF-0004	South Korea, Gyeongnam	Purple	Black	
2821	G. soja	YWS 39		FF-0006	South Korea, Gyeongnam	Purple	Black	
2822	G. soja	YWS 40		FF-0007	South Korea, Gyeongbuk	Purple	Black	
2823	G. soja	YWS 41		FF-0008	South Korea, Gyeongbuk	Purple	Black	
2824	G. soja	YWS 49		FF-0010	South Korea, Gyeongnam	Purple	Black	
2825	G. soja	YWS 51		FF-0011	South Korea, Gyeongbuk	Purple	Black	
2826	G. soja	YWS 52		FF-0012	South Korea, Gyeongbuk	Purple	Black	
2827	G. soja	YWS 54		FF-0013	South Korea, Gyeongbuk	Purple	Black	
2828	G. soja	YWS 83		FF-0018	South Korea, Gyeongnam	Purple	Black	
2829	G. soja	YWS 89		FF-0020	South Korea, Gyeongnam	Purple	Black	
	G. soja	YWS 189	IT184253	CD-0123	South Korea, Chungbuk			
	G. soja	YWS 81		FF-0017	South Korea, Gyeongnam			
2830	G. soja	YWS 109	PI291277	FF-0027	China, Heilongjiang	Purple	Black	
	G. soja	YWS 24		CD-0019	South Korea, Gyeongnam			
2831	G. soja	YWS 118	IT182992	FF-0029	South Korea, Gangwon	Purple	Black	
2832	G. soja	YWS 119	IT188372	FF-0030	South Korea, Gangwon	Purple	Black	
2833	G. soja	YWS 120	IT184247	FF-0031	South Korea, Gangwon	Purple	Black	
2834	G. soja	YWS 121	IT188382	FF-0032	South Korea, Gangwon	Purple	Black	
2835	G. soja	YWS 129	IT183030	FF-0037	South Korea, Gangwon	Purple	Black	
2836	G. soja	YWS 137	IT182940	FF-0043	South Korea, Gyeonggi	Purple	Black	
2837	G. soja	YWS 140	IT183025	FF-0044	South Korea, Gyeonggi	Purple	Black	
2838	G. soja	YWS 141	IT183065	FF-0045	South Korea, Gyeonggi	Purple	Black	Representative
	G. soja	Chungbuk Boeun sujib	IT183070	FF-0531	South Korea, Chungbuk			
2839	G. soja	YWS 155	IT188420	FF-0055	South Korea, Gyeongbuk	Purple	Black	
2840	G. soja	YWS 160	IT113061	FF-0058	South Korea, Gyeongbuk	Purple	Black	
	G. soja	Kyoungbuk Seongjusujib	IT113061	FF-0475	South Korea, Gyeongbuk			
2841	G. soja	YWS 168	IT182849	FF-0060	South Korea, Jeonnam	Purple	Black	
2842	G. soja	YWS 169	IT182851	FF-0061	South Korea, Jeonbuk	Purple	Black	
2843	G. soja	YWS 179	IT183083	FF-0068	South Korea, Chungnam	Purple	Black	
	G. soja	Chungnam Yeasan sujib	IT183083	FF-0535	South Korea, Chungnam			
2844	G. soja	YWS 189	IT184253	FF-0071	South Korea, Chungbuk	Purple	Black	
2845	G. soja	YWS 198	IT195545	FF-0076	South Korea, Chungbuk	Purple	Black	
2846	G. soja	YWS282		FF-0108	South Korea, Jeonbuk	Purple	Black	
2847	G. soja	YWS283		FF-0109	South Korea, Jeonbuk	Purple	Black	
2848	G. soja	YWS293		FF-0115	South Korea, Chungnam	Purple	Black	
2849	G. soja	YWS305		FF-0119	South Korea, Chungnam	Purple	Black	Representative
2850	G. soja	YWS322		FF-0121	South Korea, Chungbuk	Purple	Black	Representative
2851	G. soja	YWS330		FF-0125	South Korea, Chungbuk	Purple	Black	
2852	G. soja	YWS347		FF-0129	South Korea, Chungbuk	Purple	Black	
	G. soja	YWS348		CD-0202	South Korea, Chungbuk			
2853	G. soja	YWS356		FF-0132	South Korea, Chungbuk	Purple	Black	
	G. soja	YWS328		FF-0123	South Korea, Chungbuk			
	G. soja	YWS325		CD-0191	South Korea, Chungbuk			
	G. soja	YWS327		CD-0192	South Korea, Chungbuk			
	G. soja	YWS342		CD-0200	South Korea, Chungbuk			
	G. soja	YWS345		CD-0201	South Korea, Chungbuk			
	G. soja	YWS353		CD-0205	South Korea, Chungbuk			
	G. soja	YWS358		CD-0209	South Korea, Chungbuk			

	G. soja	YWS372	CD-0221	South Korea, Chungbuk			
	G. soja	YWS375	CD-0223	South Korea, Chungbuk			
	G. soja	YWS376	CD-0224	South Korea, Chungbuk			
	G. soja	YWS377	CD-0225	South Korea, Chungbuk			
	G. soja	YWS350	FF-0130	South Korea, Chungbuk			
2854	G. soja	YWS367	FF-0134	South Korea, Chungbuk	Purple	Black	
2855	G. soja	YWS392	FF-0142	South Korea, Gyeonggi	Purple	Black	
	G. soja	YWS389	CD-0231	South Korea, Gyeonggi			
	G. soja	YWS390	CD-0232	South Korea, Gyeonggi			
	G. soja	YWS394	CD-0235	South Korea, Gyeonggi			
2856	G. soja	YWS395	FF-0143	South Korea, Gyeonggi	Purple	Black	
	G. soja	YWS407	CD-0243	South Korea, Gyeongnam			
	G. soja	YWS402	CD-0239	South Korea, Gyeongbuk			
	G. soja	YWS398	CD-0236	South Korea, Gyeongbuk			
	G. soja	YWS399	CD-0237	South Korea, Gyeongbuk			
2857	G. soja	YWS400	FF-0145	South Korea, Gyeongbuk	Purple	Black	
2858	G. soja	YWS428	FF-0149	South Korea, Gangwon	Purple	Black	
2859	G. soja	YWS431	FF-0150	South Korea, Gangwon	Purple	Black	
2860	G. soja	YWS433	FF-0151	South Korea, Gangwon	Purple	Black	
2861	G. soja	YWS449	FF-0153	South Korea, Gangwon	Purple	Black	
2862	G. soja	YWS450	FF-0154	South Korea, Gangwon	Purple	Black	
2863	G. soja	YWS456	FF-0155	South Korea, Gangwon	Purple	Black	
2864	G. soja	YWS457	FF-0156	South Korea, Gangwon	Purple	Black	
2865	G. soja	YWS458	FF-0157	South Korea, Gangwon	Purple	Black	
2866	G. soja	YWS463	FF-0158	South Korea, Gangwon	Purple	Black	
2867	G. soja	YWS466	FF-0159	South Korea, Gangwon	Purple	Black	
2868	G. soja	YWS477	FF-0161	South Korea, Gangwon	Purple	Black	
2869	G. soja	YWS479	FF-0162	South Korea, Gangwon	Purple	Black	Representative
2870	G. soja	YWS515	FF-0164	South Korea, Gangwon	Purple	Black	
2871	G. soja	YWS539	FF-0168	South Korea, Gyeonggi	Purple	Black	
2872	G. soja	YWS540	FF-0169	South Korea, Gyeonggi	Purple	Black	
2873	G. soja	YWS542	FF-0170	South Korea, Gyeonggi	Purple	Black	
2874	G. soja	YWS543	FF-0171	South Korea, Gyeonggi	Purple	Black	
2875	G. soja	YWS547	FF-0174	South Korea, Gyeonggi	Purple	Black	
2876	G. soja	YWS549	FF-0176	South Korea, Gyeonggi	Purple	Black	
2877	G. soja	YWS568	FF-0180	South Korea, Gyeonggi	Purple	Black	Representative
2878	G. soja	YWS627	FF-0190	South Korea, Gyeonggi	Purple	Black	
2879	G. soja	YWS630	FF-0192	South Korea, Gyeonggi	Purple	Black	
	G. soja	YWS628	FF-0191	South Korea, Gyeonggi			
2880	G. soja	YWS632	FF-0193	South Korea, Gyeonggi	Purple	Black	
2881	G. soja	YWS639	FF-0195	South Korea, Gyeonggi	Purple	Black	
2882	G. soja	YWS642	FF-0197	South Korea, Gyeonggi	Purple	Black	
	G. soja	YWS640	FF-0196	South Korea, Gyeonggi			
2883	G. soja	YWS645	FF-0198	South Korea, Jeju	Purple	Black	
2884	G. soja	YWS646	FF-0199	South Korea, Jeju	Purple	Black	
2885	G. soja	YWS649	FF-0201	South Korea, Jeju	Purple	Black	
2886	G. soja	YWS661	FF-0203	South Korea, Jeju	Purple	Black	
2887	G. soja	YWS662	FF-0204	South Korea, Jeju	Purple	Black	
2888	G. soja	YWS694	FF-0213	South Korea, Chungbuk	Purple	Black	
2889	G. soja	YWS697	FF-0215	South Korea, Chungbuk	Purple	Black	
2890	G. soja	YWS698	FF-0216	South Korea, Chungbuk	Purple	Black	
2891	G. soja	YWS699	FF-0217	South Korea, Chungbuk	Purple	Black	
2892	G. soja	YWS724	FF-0225	South Korea, Chungnam	Purple	Black	
	G. soja	YWS725	CD-0476	South Korea, Chungnam			
2893	G. soja	YWS772	FF-0234	South Korea, Jeonbuk	Purple	Black	
	G. soja	YWS771	FF-0233	South Korea, Jeonbuk			
	G. soja	YWS770	FF-0232	South Korea, Jeonbuk			
	G. soja	YWS767	FF-0230	South Korea, Jeonbuk			
	G. soja	YWS766	CD-0513	South Korea, Jeonbuk			
	G. soja	YWS773	CD-0515	South Korea, Jeonbuk			
	G. soja	YWS774	CD-0516	South Korea, Jeonbuk			
	G. soja	YWS775	CD-0517	South Korea, Jeonbuk			
	G. soja	YWS781	CD-0522	South Korea, Jeonbuk			
	G. soja	YWS777	FF-0235	South Korea, Jeonbuk			
	G. soja	YWS768	FF-0231	South Korea, Jeonbuk			
2894	G. soja	YWS790	FF-0237	South Korea, Jeonnam	Purple	Black	
	G. soja	YWS787	CD-0528	South Korea, Jeonbuk			

	G. soja	YWS791	CD-0530	South Korea, Jeonnam			
2895	G. soja	YWS821	FF-0247	South Korea, Jeonnam	Purple	Black	
	G. soja	YWS822	FF-0248	South Korea, Jeonnam			
2896	G. soja	YWS834	FF-0249	South Korea, Gangwon	Purple	Black	
2897	G. soja	YWS837	FF-0250	South Korea, Gangwon	Purple	Black	
2898	G. soja	YWS852	FF-0254	South Korea, Gangwon	Purple	Black	
	G. soja	YWS851	CD-0574	South Korea, Gangwon			
	G. soja	YWS859	CD-0579	South Korea, Gyeongbuk			
	G. soja	YWS845	CD-0570	South Korea, Gangwon			
	G. soja	YWS849	CD-0573	South Korea, Gangwon			
2899	G. soja	YWS858	FF-0255	South Korea, Gyeongbuk	Purple	Black	
2900	G. soja	YWS888	FF-0265	South Korea, Gyeongnam	Purple	Black	
2901	G. soja	YWS890	FF-0266	South Korea, Gyeongnam	Purple	Black	
	G. soja	YWS893	CD-0601	South Korea, Gyeongnam			
2902	G. soja	YWS892	FF-0267	South Korea, Gyeongnam	Purple	Black	
2903	G. soja	YWS897	FF-0270	South Korea, Gyeongnam	Purple	Black	
2904	G. soja	YWS923	FF-0273	South Korea, Gyeonggi	Purple	Black	
2905	G. soja	YWS925	FF-0274	South Korea, Gyeonggi	Purple	Black	Representative
2906	G. soja	YWS940	FF-0277	South Korea, Gyeongbuk	Purple	Black	
2907	G. soja	YWS947	FF-0279	South Korea, Jeonnam	Purple	Black	
2908	G. soja	YWS980	FF-0288	South Korea, Jeonbuk	Purple	Black	
2909	G. soja	YWS992	FF-0292	South Korea, Jeonbuk	Purple	Black	
	G. soja	YWS981	CD-0660	South Korea, Jeonbuk			
2910	G. soja	YWS999	FF-0293	South Korea, Jeonbuk	Purple	Black	
2911	G. soja	YWS1011	FF-0295	South Korea, Jeonbuk	Purple	Black	
2912	G. soja	YWS1086	FF-0308	South Korea, Chungnam	Purple	Black	
2913	G. soja	YWS1106	FF-0313	South Korea, Gangwon	Purple	Black	
2914	G. soja	YWS1122	FF-0317	South Korea, Gangwon	Purple	Black	
2915	G. soja	YWS1126	FF-0318	South Korea, Gangwon	Purple	Black	
	G. soja	YWS1125	CD-0773	South Korea, Gangwon			
2916	G. soja	YWS1156	FF-0327	South Korea, Chungnam	Purple	Black	
2917	G. soja	YWS1164	FF-0331	South Korea, Chungnam	Purple	Black	
2918	G. soja	YWS1222	FF-0342	South Korea, Gyeongnam	Purple	Black	
2919	G. soja	YWS1223	FF-0343	South Korea, Gyeongnam	Purple	Black	
2920	G. soja	YWS1226	FF-0344	South Korea, Gyeongnam	Purple	Black	
2921	G. soja	YWS1235	FF-0345	South Korea, Gyeongnam	Purple	Black	
2922	G. soja	YWS1269	FF-0347	South Korea, Chungnam	Purple	Black	
2923	G. soja	YWS1278	FF-0349	South Korea, Gyeonggi	Purple	Black	
2924	G. soja	YWS1283	FF-0350	South Korea, Gyeonggi	Purple	Black	
2925	G. soja	YWS1291	FF-0352	South Korea, Gyeonggi	Purple	Black	
2926	G. soja	YWS1292	FF-0353	South Korea, Gyeonggi	Purple	Black	
2927	G. soja	YWS1301	FF-0354	South Korea, Gyeonggi	Purple	Black	
2928	G. soja	YWS1310	FF-0355	South Korea, Chungbuk	Purple	Black	
2929	G. soja	YWS1324	FF-0357	South Korea, Gyeongnam	Purple	Black	
2930	G. soja	YWS1330	FF-0359	South Korea, Gyeongnam	Purple	Black	
2931	G. soja	YWS1331	FF-0360	South Korea, Gyeongnam	Purple	Black	
2932	G. soja	YWS1340	FF-0361	South Korea, Gyeongnam	Purple	Black	
2933	G. soja	YWS1354	FF-0363	South Korea, Gyeongbuk	Purple	Black	
2934	G. soja	YWS1359	FF-0365	South Korea, Gyeongbuk	Purple	Black	
2935	G. soja	YWS1360	FF-0366	South Korea, Gyeongbuk	Purple	Black	
2936	G. soja	YWS1391	FF-0368	South Korea, Chungbuk	Purple	Black	
2937	G. soja	YWS1405	FF-0370	South Korea, Chungbuk	Purple	Black	
2938	G. soja	YWS1409	FF-0372	South Korea, Jeonnam	Purple	Black	
2939	G. soja	YWS1445	FF-0375	South Korea, Gyeongnam	Purple	Black	
2940	G. soja	YWS1448	FF-0376	South Korea, Gyeongnam	Purple	Black	
2941	G. soja	YWS1456	FF-0378	South Korea, Gyeongnam	Purple	Black	
2942	G. soja	YWS1467	FF-0379	South Korea, Chungbuk	Purple	Black	
2943	G. soja	YWS1468	FF-0380	South Korea, Gyeongbuk	Purple	Black	
2944	G. soja	YWS1477	FF-0381	South Korea, Gyeongbuk	Purple	Black	
2945	G. soja	YWS1502	FF-0382	South Korea, Gyeonggi	Purple	Black	
2946	G. soja	YWS1522	FF-0383	South Korea, Gyeongbuk	Purple	Black	
2947	G. soja	YWS1523	FF-0384	South Korea, Gyeongbuk	Purple	Black	
2948	G. soja	YWS1525	FF-0386	South Korea, Gyeongbuk	Purple	Black	
2949	G. soja	YWS1527	FF-0388	South Korea, Gyeongbuk	Purple	Black	
2950	G. soja	YWS1534	FF-0389	South Korea, Jeonnam	Purple	Black	
2951	G. soja	YWS1535	FF-0390	South Korea, Jeonnam	Purple	Black	
2952	G. soja	YWS1536	FF-0391	South Korea, Jeonnam	Purple	Black	

2953	G. soja	YWS1537		FF-0392	South Korea, Jeonnam	Purple	Black	
	G. soja	YWS1538		FF-0393	South Korea, Jeonnam			
2954	G. soja	YWS1563		FF-0396	South Korea, Chungnam	Purple	Black	
2955	G. soja	YWS1564		FF-0397	South Korea, Chungnam	Purple	Black	
2956	G. soja	YWS1572		FF-0399	South Korea, Jeonnam	Purple	Black	
2957	G. soja	YWS1575		FF-0400	South Korea, Jeonnam	Purple	Black	
2958	G. soja	YWS1576		FF-0401	South Korea, Jeonnam	Purple	Black	
2959	G. soja	YWS1577		FF-0402	South Korea, Jeonnam	Purple	Black	
2960	G. soja	YWS1583		FF-0403	South Korea, Jeonnam	Purple	Black	
2961	G. soja	YWS1588		FF-0405	South Korea, Jeonnam	Purple	Black	
2962	G. soja	YWS1589		FF-0406	South Korea, Jeonnam	Purple	Black	
2963	G. soja	YWS1595		FF-0407	South Korea, Jeonbuk	Purple	Black	
2964	G. soja	YWS1602		FF-0408	South Korea, Jeonbuk	Purple	Black	
2965	G. soja	YWS1603		FF-0409	South Korea, Jeonbuk	Purple	Black	
2966	G. soja	YWS1604		FF-0410	South Korea, Jeonbuk	Purple	Black	
2967	G. soja	B01098	JPN2	FF-0411	Japan, Hokkaido	Purple	Black	Representative
2968	G. soja	B01143	JPN6	FF-0413	Japan, Hokkaido	Purple	Black	Representative
2969	G. soja	B02086	JPN12	FF-0415	Japan, Aomori	Purple	Black	Representative
2970	G. soja	B02136	JPN14	FF-0417	Japan, Yamagata	Purple	Black	Representative
2971	G. soja	B02154	JPN15	FF-0418	Japan, Miyagi	Purple	Black	Representative
2972	G. soja	B02165	JPN16	FF-0419	Japan, Iwate	Purple	Black	Representative
2973	G. soja	B03051	JPN30	FF-0425	Japan, Fukui	Purple	Black	Representative
2974	G. soja	B04054	JPN31	FF-0426	Japan, Nagano	Purple	Black	Representative
2975	G. soja	B04148	JPN39	FF-0429	Japan, Saitama	Purple	Brown	Representative
2976	G. soja	B05055	JPN44	FF-0430	Japan, Mie	Purple	Black	Representative
2977	G. soja	B05057	JPN45	FF-0431	Japan, Mie	Purple	Black	Representative
2978	G. soja	B06046	JPN48	FF-0432	Japan, Nara	Purple	Black	Representative
2979	G. soja	B07072	JPN52	FF-0435	Japan, Kochi	Purple	Black	Representative
2980	G. soja	B08040	JPN60	FF-0436	Japan, Yamaguchi	Purple	Black	Representative
2981	G. soja	B08050	JPN62	FF-0437	Japan, Shimane	Purple	Black	Representative
2982	G. soja	PI 483462 A	CHN3	FF-0442	China, Beijing	Purple	Black	Representative
	G. soja	PI 483462 B	CHN4	FF-0443	China, Beijing			
2983	G. soja	PI 483461	CHN5	FF-0444	China, Hebei	Purple	Black	Representative
2984	G. soja	PI 464936 B	81-200004, CNH22	FF-0447	China, Jiangsu	Purple	Black	Representative
2985	G. soja	PI 464927 C	LS-005, CHN34	FF-0450	China, Liaoning	Purple	Black	Representative
2986	G. soja	PI 483464 B	CHN40	FF-0452	China, Ningxia	Purple	Black	Representative
2987	G. soja	PI 483466	CHN43	FF-0453	China, Shandong	Purple	Black	Representative
2988	G. soja	PI 483463	CHN52	FF-0457	China, Shanxi	Purple	Black	Representative
2989	G. soja	PI 407262		FF-0464	South Korea, Gyeongnam	Purple	Black	Representative
2990	G. soja	PI 407230		FF-0465	South Korea, Chungnam	Purple	Black	
2991	G. soja	PI 407255		FF-0467	South Korea, Gyeongnam	Purple	Black	
2992	G. soja	Kangwon hoengseong sujib	IT103915	FF-0472	South Korea, Gangwon	Purple	Black	
2993	G. soja	Jeonbuk jangsu-1985-5751	IT105751	FF-0474	South Korea, Jeonbuk	Purple	Black	
2994	G. soja	Chungnam yesan sujib	IT178534	FF-0478	South Korea, Chungnam	Purple	Black	
2995	G. soja	Chungnam yesan sujib	IT178541	FF-0479	South Korea, Chungnam	Purple	Black	
2996	G. soja	Chungnam jongju sujib	IT182817	FF-0481	South Korea, Chungnam	Purple	Black	
2997	G. soja	Buyeo	IT182822	FF-0482	South Korea, Chungnam	Purple	Black	
2998	G. soja	Chungnam nonsan sujib	IT182829	FF-0484	South Korea, Chungnam	Purple	Black	
2999	G. soja	Daejeon daedeok sujib	IT182831	FF-0485	South Korea, Chungnam	Purple	Black	
3000	G. soja	Jeonbuk jinan sujib	IT182838	FF-0488	South Korea, Jeonbuk	Purple	Black	
3001	G. soja	Jeonbuk sunchang sujib	IT182842	FF-0489	South Korea, Jeonbuk	Purple	Black	
3002	G. soja	IT182847	Jeonbuk Namwon sujib	FF-0490	South Korea, Jeonbuk	Purple	Black	
3003	G. soja	Gyeongbuk seongju sujib	IT182856	FF-0492	South Korea, Gyeongbuk	Purple	Black	
3004	G. soja	Gyeongbuk seongju sujib	IT182860	FF-0493	South Korea, Gyeongbuk	Purple	Black	
3005	G. soja	IT182864	Gyeongnam Geochang sujib	FF-0494	South Korea, Gyeongnam	Purple	Black	
3006	G. soja	Gyeongnam changnyeong sujib	IT182870	FF-0495	South Korea, Gyeongnam	Purple	Black	

3007	G. soja	Gyeonggi hwaseong sujib	IT182930	FF-0496	South Korea, Gyeonggi	Purple	Black	
3008	G. soja	Gyeonggi yongin sujib	IT182934	FF-0497	South Korea, Gyeonggi	Purple	Black	
3009	G. soja	Gyeonggi yongin sujib	IT182938	FF-0498	South Korea, Gyeonggi	Purple	Black	
3010	G. soja	Gyeonggi anseong sujib	IT182942	FF-0499	South Korea, Gyeonggi	Purple	Black	
3011	G. soja	IT182946	Gyeonggi Hwaseong sujib	FF-0500	South Korea, Gyeonggi	Purple	Black	
3012	G. soja	Gyeonggi hwaseong sujib	IT182950	FF-0501	South Korea, Gyeonggi	Purple	Black	
3013	G. soja	Gyeonggi hwaseong sujib	IT182965	FF-0504	South Korea, Gyeonggi	Purple	Black	
3014	G. soja	IT183036	Chungbuk Jecheon sujib	FF-0505	South Korea, Chungbuk	Purple	Black	
	G. soja	Chungbuk Jechun sujib	IT184089	FF-0546	South Korea, Chungbuk			
	G. soja	Jeonnam Haenam sujib	IT182982	FF-0508	South Korea, Jeonnam			
3015	G. soja	IT182974		FF-0506	South Korea, Jeonnam	Purple	Black	Representative
3016	G. soja	IT182976	Gyeonggi Yongin sujib	FF-0507	South Korea, Gyeonggi	Purple	Black	
3017	G. soja	Jeonnam gangjin sujib	IT182985	FF-0509	South Korea, Jeonnam	Purple	Black	
3018	G. soja	Gyeonggi yongin sujib	IT182990	FF-0511	South Korea, Gyeonggi	Purple	Black	
3019	G. soja	IT182993	Kangwon Wonju sujib	FF-0512	South Korea, Gangwon	Purple	Black	
3020	G. soja	Chungbuk cheongwon sujib	IT182998	FF-0514	South Korea, Chungbuk	Purple	Black	
3021	G. soja	Gyeongnam changnyeong sujib	IT183002	FF-0515	South Korea, Gyeongnam	Purple	Black	
3022	G. soja	Gyeongnam changnyeong sujib	IT183007	FF-0516	South Korea, Gyeongnam	Purple	Black	
3023	G. soja	Kangwon inje sujib	IT183023	FF-0518	South Korea, Gangwon	Purple	Black	
	G. soja	Gyeonggi yangpyung sujib	IT183026	FF-0519	South Korea, Gyeonggi			
	G. soja	kwangwon hongcheon sujib	IT184227	FF-0565	South Korea, Gangwon			
	G. soja	Gyungnam changnyung sujib	IT184179	FF-0555	South Korea, Gyeongnam			
	G. soja	YWS 175	IT184186	FF-0067	South Korea, Chungnam			
	G. soja	YWS569		CD-0371	South Korea, Gyeonggi			
	G. soja	YWS 131	IT184229	CD-0093	South Korea, Gangwon			
	G. soja	YWS 138	IT183024	CD-0095	South Korea, Gyeonggi			
3024	G. soja	IT183036	Kangwon Yeongwol sujib	FF-0521	South Korea, Gangwon	Purple	Black	
	G. soja	YWS 115	IT183035	CD-0086	South Korea, Gangwon			
3025	G. soja	IT183037	Chungbuk Jecheon sujib	FF-0522	South Korea, Chungbuk	Purple	Black	
3026	G. soja	Chungbuk jecheon sujib	IT183039	FF-0523	South Korea, Chungbuk	Purple	Black	
3027	G. soja	IT183045		FF-0525	South Korea, Gyeongbuk	Purple	Black	Representative
3028	G. soja	Gyeongbuk andong sujib	IT183049	FF-0526	South Korea, Gyeongbuk	Purple	Black	
3029	G. soja	Gyeongbuk sangju sujib	IT183062	FF-0529	South Korea, Gyeongbuk	Purple	Black	
3030	G. soja	Chungnam seosan sujib	IT183092	FF-0536	South Korea, Chungnam	Purple	Black	
3031	G. soja	IT183105	Chungnam Hongseong sujib	FF-0539	South Korea, Chungnam	Purple	Black	
	G. soja	YWS 180	IT183105	CD-0117	South Korea,			
	G. soja	YWS 188	IT178480	CD-0122	South Korea, Chungbuk			
3032	G. soja	IT183115	Chungnam Dangjin sujib	FF-0542	South Korea, Chungnam	Purple	Black	
3033	G. soja	IT183120	Gyeonggi Pyeongtaek sujib	FF-0543	South Korea, Gyeonggi	Purple	Black	
3034	G. soja	Gyeonggi yongin sujib	IT184053	FF-0544	South Korea, Gyeonggi	Purple	Black	
3035	G. soja	gyeonggi hwaseong sujib	IT184072	FF-0545	South Korea, Gyeonggi	Purple	Black	

	G. soja	Gyeonggi hwangseong sujib	IT182960	FF-0503	South Korea, Gyeonggi			
3036	G. soja	IT184108	Jeonnam Gangjin sujib	FF-0547	South Korea, Jeonnam	Purple	Black	
3037	G. soja	gyeonggi yongin sujib	IT184136	FF-0549	South Korea, Gyeonggi	Purple	Black	
3038	G. soja	IT184146	Kangwon Chunseong sujib	FF-0550	South Korea, Gangwon	Purple	Black	
3039	G. soja	Kangwon wonseong sujib	IT184154	FF-0551	South Korea, Gangwon	Purple	Black	
3040	G. soja	Chungbuk jungwon sujib	IT184161	FF-0552	South Korea, Chungbuk	Purple	Black	
3041	G. soja	Chungbuk jungwon sujib	IT184163	FF-0553	South Korea, Chungbuk	Purple	Black	
3042	G. soja	gyeonggi hwaseong sujib	IT184171	FF-0554	South Korea, Gyeonggi	Purple	Black	
3043	G. soja	Chungnam daedeok sujib	IT184183	FF-0556	South Korea, Chungnam	Purple	Black	
3044	G. soja	Chungnam yeongi sujib	IT184191	FF-0557	South Korea, Chungnam	Purple	Black	
3045	G. soja	IT184194	Gyeonggi gwacheon sujib	FF-0558	South Korea, Gyeonggi	Purple	Black	
3046	G. soja	Gyeonggi hwaseong sujib	IT184200	FF-0559	South Korea, Gyeonggi	Purple	Yellow	
3047	G. soja	Gyeonggi yeoncheon sujib	IT184206	FF-0560	South Korea, Gyeonggi	Purple	Black	
3048	G. soja	Gyeonggi pocheon sujib	IT184210	FF-0561	South Korea, Gyeonggi	Purple	Black	
3049	G. soja	Cheorwon	IT184216	FF-0562	South Korea, Gangwon	Purple	Black	
3050	G. soja	Kangwon yanggu sujib	IT184221	FF-0564	South Korea, Gangwon	Purple	Black	
3051	G. soja	IT184233		FF-0566	South Korea, Gyeonggi	Purple	Black	Representative
3052	G. soja	IT184238		FF-0567	South Korea, Gyeonggi	Purple	Black	Representative
3053	G. soja	IT184254	Chungbuk Yeongdong sujib	FF-0569	South Korea, Chungbuk	Purple	Black	
3054	G. soja	IT184258	Gyeongbuk Yecheon sujib	FF-0570	South Korea, Gyeongbuk	Purple	Black	
3055	G. soja	IT188353	Gyeonggi Gapyeong sujib	FF-0571	South Korea, Gyeonggi	Purple	Black	
3056	G. soja	gyeonggi gapyeong sujib	IT188356	FF-0572	South Korea, Gyeonggi	Purple	Black	
3057	G. soja	IT188367	Kangwon Yanggu sujib	FF-0575	South Korea, Gangwon	Purple	Black	
3058	G. soja	Kangwon hongcheon sujib	IT188375	FF-0577	South Korea, Gangwon	Purple	Black	
3059	G. soja	Kangwon pyeongchang sujib	IT188380	FF-0578	South Korea, Gangwon	Purple	Black	
3060	G. soja	Kangwon yeongwol sujib	IT188383	FF-0579	South Korea, Gangwon	Purple	Black	
3061	G. soja	Chungbuk goesan sujib	IT188392	FF-0582	South Korea, Chungbuk	Purple	Black	
3062	G. soja	Chungbuk goesan sujib	IT188395	FF-0583	South Korea, Chungbuk	Purple	Black	
3063	G. soja	Chungbuk goesan sujib	IT188398	FF-0584	South Korea, Chungbuk	Purple	Black	
3064	G. soja	Gyeongnam hapcheon sujib	IT188422	FF-0589	South Korea, Gyeongnam	Purple	Black	
3065	G. soja	Gyeongnam changnyeong sujib	IT188427	FF-0590	South Korea, Gyeongnam	Purple	Black	
3066	G. soja	IT195544	Chungbuk Jincheon sujib	FF-0592	South Korea, Chungbuk	Purple	Black	
3067	G. soja	Chungbuk cheongwon sujib	IT195549	FF-0593	South Korea, Chungbuk	Purple	Black	
3068	G. soja	IT195562	Gwangju Gwangsan sujib	FF-0597	South Korea, Jeonnam	Purple	Black	
3069	G. soja	Jeonnam hampyeong sujib	IT195566	FF-0598	South Korea, Jeonnam	Purple	Black	
3070	G. soja	Jangheung	IT195572	FF-0599	South Korea, Jeonnam	Purple	Black	
3071	G. soja	Jeonnam boseong sujib	IT195575	FF-0600	South Korea, Jeonnam	Purple	Black	
3072	G. soja	PI 203246		FF-0602	Japan, Fukuoka	Purple	Black	Representative
3073	G. soja	PI 378698		FF-0603	Japan, Yamanashi	Purple	Black	Representative

3074	G. soja	IT162825		IT162825	South Korea, Gyeongbuk	Purple	Black	Representative
3075	G. soja	IT178458		IT178458-1	South Korea, Chungbuk	Purple	Black	Representative
	G. soja	Chungnam gongju sujib	IT183111	FF-0541	South Korea, Chungnam			
3076	G. soja	IT178480		IT178480	South Korea, Chungbuk	Purple	Black	Representative
3077	G. soja	IT182819		IT182819-1	South Korea, Jeonnam	Purple	Black	
3078	G. soja	IT182840		IT182840	South Korea, Jeonbuk	Purple	Black	Representative
3079	G. soja	IT182848		IT182848	South Korea, Jeonnam	Purple	Black	Representative
3080	G. soja	IT182819		IT182919-1	South Korea, Chungnam	Purple	Black	DNA mislabelled
3081	G. soja	IT182932		IT182932	South Korea, Gyeonggi	Purple	Black	Representative
3082	G. soja	PI 339871 B		PI339871B-2	South Korea, Jeju	Purple	Black	Representative
3083	G. soja	PI 342620 A		PI342620A-1	Russia, Primorye	Purple	Black	Representative
3084	G. soja	PI 578346 B		PI578346B-1	Russia, Primorye	Purple	Black	Representative
3085	G. soja	PI 424002		PI404002-1	Russia, Amur	Purple	Black	Representative
3086	G. soja	PI 507800 B		PI507800B-1	Russia, Primorye	Purple	Black	Representative
	G. soja	PI 597457 A	ZYD3232, CHN44	CD-1143	China, Shandong			
