# Supplementary material for: Genetic diversity patterns and domestication origin of soybean
Source: Theor Appl Genet. 2018 Dec 26;132(4):1179–93. doi: 10.1007/s00122-018-3271-7 (PMC6449312; doi:10.1007/s00122-018-3271-7)
Supplement: Supplementary file 3 — Supplementary material 3 (PDF 396 kb) [file 122_2018_3271_MOESM3_ESM.pdf]

Supplementary Tables S2 to S6 for

Genetic diversity patterns and domestication origin of soybean

Jeong et al., 2018

Table S2. Summary of groupings and geographic origins of soybean accessions

A total of 4,234 accessions were genotyped with higher than 97% sample call rate. The 4,234 accessions were grouped into their countries of origin based mainly on data retrieved from the National Agrobiodiversity Center (http://genebank.rda.go.kr/) in Korea and the Germplasm Resource Information Network (https://www.ars-grin.gov/) in Illinois, USA. Non-redundant 3036 set was obtained from filtration of duplicated or redundant accessions. Representative population study set was obtained mainly by filtering closely related accessions collected in South Korea.

Study set	*Glycine max*						*G. soja*					Hybrid			
	Total	South Korea	North Korea	China	Japan	Improved or unknown type	Total	South Korea	China	Japan	Russia	Total	South Korea	China	Japan
Total set of 4234	2824	2224	89	56	17	438	1360	1244	57	53	6	50	37	11	2
Non-redundant 3036 set	1957	1485	83	56	16	316	1079	984	40	50	5				
Representative population study set	50	17	16	13	4	0	144	50	39	50	5				

Table S3. Pairwise comparisons of percent inconsistencies between duplicated accessions, between isogenic lines, and between pedigree-unknown samples

Percent inconsistencies between Williams isoliness									
	OT94-49	OT94-51	L67-153	Harosoy	L62-667	OT89-05	OT89-06	OT93-26	OT93-28
OT94-49									
OT94-51	0.83								
L67-153	1.48	0.55							
Harosoy	1.11	1.16	1.16						
L62-667	1.11	1.48	1.16	1.22					
OT89-05	0.78	0.85	1.23	0.68	1.03				
OT89-06	0.76	0.84	1.21	0.85	1.35	0.54			
OT93-26	0.41	0.84	1.45	0.92	1.39	0.66	0.91		
OT93-28	0.58	0.65	1.39	0.85	1.18	0.42	0.80	0.38	
OT94-37	0.71	0.99	1.10	0.62	1.28	0.75	0.66	0.75	0.91

Percent inconsistencies between Clark isoliness and between Clark duplicates					
	Clark (Nod)	Clark 63	Clark	PI547413-1	Clark
Clark (Nod)					
Clark 63	0.48				
Clark	0.94	0.98			
L62-1579	1.05	1.09	0.61		
Clark	1.00	0.81	0.99	1.10	
Clark	0.74	0.76	0.37	0.47	0.76

Percent inconsistencies between Williams isolines and between Williams isolines and landraces							
		Winchester	Williams82K	Uid	KLS 129-1	L77-1794	L29
Isoline	Winchester						
Isoline	Williams82K	1.01					
Isoline	Uid	1.50	1.06				
Landrace	KLS 129-1	0.89	0.62	1.09			

Isoline	L77-1794	0.13	0.82	1.08	0.99		
Isoline	L29	1.24	0.96	1.33	0.83	1.24	
Landrace	KLS85102	1.27	0.87	0.72	0.69	0.64	1.10

Percent inconsistencies between pedigree-unknown landraces and cultivars

	Norchief	KAS590-1	KLS904-2	PI 399045
Norchief				
KAS590-1	0.74			
KLS904-2	0.14	0.83		
PI 399045	0.07	0.76	0.16	
KAS548-10	0.76	0.06	0.84	0.74

Table S4. List of subpopulation-specific single nucleotide polymorphism (SNP) markers used as covariates in the conditional logistic regression model analysis

SNP Markers	Chromosome	Position	Minor allele frequency			Remarks
			Korean specific group[b]	The other *Glycine max* group	*G. soja*	
AX-90369371	Scaffold_1871[a]	214	0	0	0	Minor alleles are different in *G. max* and *G. soja*
AX-90513805	13	23,482,886	0.011	0.257	0	
AX-90370689	8	38,800,023	0.088	0.249	0	
AX-90485014	13	35,027,391	0.005	0.268	0.475	
AX-90470893	11	31,613,799	0.006	0.207	0.355	
AX-90316127	10	49,952,082	0.161	0.058	0.401	
AX-90471811	6	6,515,763	0.267	0.413	0.049	MAF = 0 in *G. soja* Gs-I and Gs-II groups. Minor allele in Korean specific group is different from that in the other groups
AX-90422942	9	44,053,258	0.424	0.282	0.039	MAF = 0 in *G. soja* Gs-I and Gs-II groups
AX-90449659	1	53,334,898	0.188	0.117	0.012	MAF = 0 in *G. soja* Gs-I and Gs-II groups
AX-90381233	5	39,573,688	0.019	0.127	0.003	MAF = 0 in G. *soja* Gs-III and Gs-IV groups
AX-90521508	2	10,614,251	0.411	0.470	0.001	MAF = 0 in *G. soja* Gs-I, Gs-II, and Gs-IV groups

[a] An unanchored scaffold in the soybean reference genome sequence Wm82.a2.v1 (https://phytozome.jgi.doe.gov/pz/portal.html).

[b] Minor allele frequency (MAF) in Korean specific *Glycine max* group that the majority of Korean accessions cluster together, as defined in Fig. 1A.

Table S5. Analysis of molecular variance (AMOVA) of the 3,036 non-redundant soybean set and the 194 representative soybean set between (1) two groups (a *G. max* group and a *G. soja* group containing four populations identified by PCA) and (2) all populations consisting of a *G. max* and four *G. soja* populations

	Source of variation	d.f.	Sum of squares	Variance components	Percentage of variation	Fixation indices[a]	*P* value
3036 non-redundant soybean set							
(1)	Among groups	1	22808305.226	6215.98313	28.79	$F_{CT} = 0.28791$	~ 0.2082
	Among populations within groups	3	824170.558	2159.31397	10.00	$F_{SC} = 0.14045$	< 0.0001
	Within populations	6067	80175734.437	13215.05430	61.21	$F_{ST} = 0.38792$	< 0.0001
(2)	Among populations	4	23632475.784	8099.59057	38.00	$F_{ST} = 0.38000$	< 0.0001
	Within populations	6067	80175734.437	13215.05430	62.00		
194 representative soybean set							
(1)	Among groups	1	991047.057	4623.28461	20.01	$F_{CT} = 0.20009$	~ 0.2053
	Among populations within groups	3	658718.047	3011.32268	13.03	$F_{SC} = 0.16292$	< 0.0001
	Within populations	383	5925746.388	15471.92268	66.96	$F_{ST} = 0.33041$	< 0.0001
(2)	Among populations	4	1649765.104	5303.86051	25.53	$F_{ST} = 0.25529$	< 0.0001
	Within populations	383	5925746.388	15471.92268	74.47		

[a]Fixation indexes: F_{CT}, difference among groups, F_{SC}, difference among populations within groups; F_{ST}, difference among populations

Notes: In this analysis, the *G. max* group was treated as one group or population, with an objective to find its most closely related population in the four *G. soja* populations. In both the 3,036 whole soybean set and the 194 representative set, the fixation indices among groups (F_{CT}) was not significant, whereas those among populations within groups (F_{SC}) were significant. These results confirmed that the *G. soja* group was structured. A proportion of the genetic differentiation (28.79%) among groups of the 3,036 non-redundant soybean set was higher than that (20.1%) of the 194 representative set, whereas proportions of the genetic differentiation among populations within groups and among

populations slightly increased in the 194 representative set, and the F_{ST} and F_{SC} values were highly significant. Thus, the AMOVA results indicated that the *G. soja* representative set likely well represented the whole *G. soja* population. The results of the AMOVA considering all populations as one demonstrated that the variance among populations in the 3,036 whole soybean set was higher than that in the 194 representative set and the F_{ST} values were highly significant in both the sets. The results indicated that although the diversity level of the *G. max* population in the representative set was slightly lower than that in the whole soybean set, the five populations consisting of one *G. max* and four geographically separated *G. soja* populations, which were observed in the 3,036 whole soybean set, were well represented in the resultant representative set.

Table S6. List of 62 wild soybeans (*Glycine soja* Siebold & Zucc.) whose genome resequencing data were reported by Tian et al. (2015)[a]

Individual code	Accession name	Origin	Remarks
IGDB-001	ZJ-ZY020	Zhejiang, China	
IGDB-002	ZJ-YJ086	Zhejiang, China	Heterozygous SNP calls > 20%
IGDB-003	ZJ-Y314	Zhejiang, China	
IGDB-004	ZJ-Y217	Zhejiang, China	
IGDB-005	ZJ-Y200	Zhejiang, China	
IGDB-006	ZJ-Y191	Zhejiang, China	
IGDB-007	ZJ-Y188	Zhejiang, China	
IGDB-008	ZJ-Y108	Zhejiang, China	
IGDB-009	ZJ-YJ038	Zhejiang, China	
IGDB-010	ZJ-Y282	Zhejiang, China	
IGDB-011	ZJ-Y2300-1	Zhejiang, China	
IGDB-012	ZJ-Y155	Zhejiang, China	
IGDB-013	PI 597461C	Shandong, China	
IGDB-014	PI 597461A	Shandong, China	
IGDB-015	PI 597459D	Shandong, China	Genotyped with the SNP array in this study
IGDB-016	PI 597459C	Shandong, China	
IGDB-017	PI 593983	Hokkaido, Japan	
IGDB-018	PI 578357	Amur, Russian Federation	
IGDB-019	PI 578341	Khabarovsk, Russian Federation	
IGDB-020	PI 562565	ChollaPuk, Korea	
IGDB-021	PI 562559	ChollaPuk, Korea	
IGDB-022	PI 549046	Shaanxi, China	Genotyped with the SNP array in this study
IGDB-023	PI 547831	Illinois, United States	Hybrid (*G. max* x *G. soja*); PI 65549(6) x PI 96983
IGDB-024	PI 522228	Primorye, Russian Federation	
IGDB-025	PI 522226	Primorye, Russian Federation	
IGDB-026	PI 522216	Primorye, Russian Federation	
IGDB-027	PI 522182B	Heilongjiang, China	
IGDB-028	PI 507662	Kagoshima, Japan	
IGDB-029	PI 504286	ChungchongPuk, Korea	Heterozygous SNP calls > 20%
IGDB-030	PI 483465	Shaanxi, China	Genotyped with the SNP array in this study
IGDB-031	PI 483464A	Ningxia, China	Heterozygous SNP calls > 20%
IGDB-032	PI 483460B	Liaoning, China	
IGDB-033	PI 479769	Heilongjiang, China	
IGDB-034	PI 479752	Jilin, China	
IGDB-035	PI 468916	Liaoning, China	

IGDB-036	PI 468400A	Ningxia, China	
IGDB-037	PI 464935	Jiangsu, China	Genotyped with the SNP array in this study
IGDB-038	PI 464929B	Liaoning, China	Heterozygous SNP calls > 20%
IGDB-039	PI 464929A	Liaoning, China	
IGDB-040	PI 464927A	Liaoning, China	
IGDB-041	PI 458538	Heilongjiang, China	
IGDB-042	PI 458536	Heilongjiang, China	Heterozygous SNP calls > 20%
IGDB-043	PI 458535	Heilongjiang, China	
IGDB-044	PI 447004	Jilin, China	Heterozygous SNP calls > 20%
IGDB-045	PI 424096	ChungchongNam, Korea	
IGDB-046	PI 423991	Amur, Russian Federation	Heterozygous SNP calls > 20%
IGDB-047	PI 407301	Zhejiang, China	
IGDB-048	PI 407288	Jilin, China	Genotyped with the SNP array in this study
IGDB-049	PI 407285	Kanagawa, Japan	
IGDB-050	PI 407275	Kyonggi, Korea	
IGDB-051	PI 407246	KyongsangPuk, Korea	Heterozygous SNP calls > 20%
IGDB-052	PI 407197	Kangwon, Korea	
IGDB-053	PI 407170	Kyonggi, Korea	
IGDB-054	PI 407131	Kumamoto, Japan	
IGDB-055	PI 407027	Akita, Japan	Heterozygous SNP calls > 20%
IGDB-056	PI 393551	Hsinchu Hsien, Taiwan	
IGDB-057	PI 378692	Iwate, Japan	
IGDB-058	PI 366123	Iwate, Japan	
IGDB-059	PI 366121	Fukushima, Japan	Genotyped with the SNP array in this study; homozygous SNPs 98.16% identity; Heterozygous SNP calls > 20%
IGDB-060	PI 366120	Akita, Japan	
IGDB-061	PI 339871A	Cheju, Korea	
IGDB-062	PI 326582A	Primorye, Russian Federation	Heterozygous SNP calls > 20%

[a] Notes: When we examined the extracted SNP data from the 62 accessions, we observed that the level of heterozygous SNPs (mean = 7.6%) in the 62 accessions was significantly higher than that (mean = 0.53%) in our representative set (*t*-test, $P < 0.001$). Thus, of the 62 accessions, eleven that showed > 20 % heterozygous SNPs were excluded. However, the level of heterozygous SNPs (mean = 2.96%) in the remaining 51 accessions was still significantly higher than that in our representative set (*t*-test, $P = 0.001$). PI 366121, which has 20.7% heterozygous SNPs, was detected to be a hybrid in our SNP array data analysis and record of PI 547831 in the US National Genetic Resources Program indicated that it is an isoline backcross-derived between *G. soja* and *G. max*. In addition, five accessions overlapped with accessions in our representative set. In results, 45 of the 62 accessions were incorporated into the representative set.
